# ToxiM: A Toxicity Prediction Tool for Small Molecules Developed Using Machine Learning and Chemoinformatics Approaches

**DOI:** 10.3389/fphar.2017.00880

**Published:** 2017-11-30

**Authors:** Ashok K. Sharma, Gopal N. Srivastava, Ankita Roy, Vineet K. Sharma

**Affiliations:** Metagenomics and Systems Biology Laboratory, Department of Biological Sciences, Indian Institute of Science Education and Research, Bhopal, India

**Keywords:** machine leaning, toxicity prediction, chemoinformatics, solubility, permeability, regression model, classification models

## Abstract

The experimental methods for the prediction of molecular toxicity are tedious and time-consuming tasks. Thus, the computational approaches could be used to develop alternative methods for toxicity prediction. We have developed a tool for the prediction of molecular toxicity along with the aqueous solubility and permeability of any molecule/metabolite. Using a comprehensive and curated set of toxin molecules as a training set, the different chemical and structural based features such as descriptors and fingerprints were exploited for feature selection, optimization and development of machine learning based classification and regression models. The compositional differences in the distribution of atoms were apparent between toxins and non-toxins, and hence, the molecular features were used for the classification and regression. On 10-fold cross-validation, the descriptor-based, fingerprint-based and hybrid-based classification models showed similar accuracy (93%) and Matthews's correlation coefficient (0.84). The performances of all the three models were comparable (Matthews's correlation coefficient = 0.84–0.87) on the blind dataset. In addition, the regression-based models using descriptors as input features were also compared and evaluated on the blind dataset. Random forest based regression model for the prediction of solubility performed better (*R*^2^ = 0.84) than the multi-linear regression (MLR) and partial least square regression (PLSR) models, whereas, the partial least squares based regression model for the prediction of permeability (caco-2) performed better (*R*^2^ = 0.68) in comparison to the random forest and MLR based regression models. The performance of final classification and regression models was evaluated using the two validation datasets including the known toxins and commonly used constituents of health products, which attests to its accuracy. The ToxiM web server would be a highly useful and reliable tool for the prediction of toxicity, solubility, and permeability of small molecules.

## Introduction

The human body is exposed to numerous chemical substances in our daily life including natural compounds, cosmetics, pharmaceuticals, and other chemicals. Several of these chemicals are known to cause adverse drug reactions, non-acute and sub-acute poisoning leading to allergic reactions, and sometimes disability or death due to their mutagenic, carcinogenic or toxic nature (Shonkoff et al., 2012). In addition, our body is also exposed to toxic gases and aerosols such as carbon monoxide, lead, cigarette smoke, wood smoke, workplace chemicals, and dietary exposures to pesticides and herbicides that cause chronic disease mortality (Seaton et al., 1995). Even toxins and onco-metabolites, which are produced naturally during microbial metabolic processes in the gut have been shown to cause diabetes, kidney disease and cancer (Nowicki and Gottlieb, 2015). The damaging potential of a toxin is determined by a multitude of related factors and not just by its inherent toxicity. The observed clinical consequences of any toxin are influenced by chemical, biological and exposure related factors depend upon its absorption, metabolism and elimination from the body. Dose along with duration, frequency and route of administration of toxin are the crucial exposure-related factors (Klaassen and Amdur, 1996). Overall, the toxicity can be understood as the sum of adverse effects exhibited by a substance on any organism.

The chemical ingredients commonly used in human applications usually go through clinical trials to be certified as safe for use in certain limits. The simplest experimental measure of toxicity is the use of bio-assays involving animals injected with the toxin (Borenfreund and Puerner, 1985). The experimental measures are known to be tedious, time-consuming and have their own limitations (Harry et al., 1998). Therefore, there is a need for alternate methods, which can use the inherent properties of a given molecule for the determination of its toxic nature (Hinderliter et al., 2010). In this scenario, the computational methods appear promising in determining the toxicity of a given compound using its structural and molecular properties. The most commonly used features for these properties are molecular descriptors (Dong et al., 2015) and fingerprints (Xue and Bajorath, 2000), which can extract the chemical and structural information inherent in any given molecule for prediction-based approaches. The chemical properties of a molecule also determine its solubility (Hutchinson et al., 1979), which influences its absorption; and a molecule with poor solubility will show limited absorption and hence reduced toxicity (Hutchinson et al., 1979). Thus, the aqueous solubility, and also the permeability (caco-2 permeability) are important toxicity determining factors. The caco-2 cells are human colon epithelial cancer cell lines, which are used as a model to predict the intestinal absorption of molecules using experimental methods (Van Breemen and Li, 2005).

Therefore, the chemical and structural properties of a molecule can be exploited for the prediction of toxicity of a given molecule, the determination of which is an important and challenging task. At present, feature-based methods such as ToxiPred (Mishra et al., 2014), DeepTox (Mayr et al., 2016) are available for the prediction of toxicity (Cheng et al., 2012). Another tool is admetSAR, a freely available tool which uses various classification and regression models for the prediction of ADMET properties. Also, a large number of machine learning tools are available for the toxicological QSAR problems, but are molecule-specific due to their training on highly similar molecules with a similar backbone to construct such models. In summary, the available tools have their own limitations since most of the available tools are either highly specific for a particular toxicity, or are not freely available in many cases. Thus, there is a need for an accurate, efficient, comprehensive and easily accessible computational tool to predict the toxicity and toxicity-related properties of a molecule. In this work, by integrating machine-leaning and chemoinformatics approaches, we have developed a computational method “ToxiM” for the prediction of toxicity of molecules using fingerprints and descriptors as input features. It is freely available at http://metagenomics.iiserb.ac.in/ToxiM/.

## Materials and methods

### Dataset preparation

To develop the prediction modules using machine learning approaches, two distinct datasets of molecules were curated (i) positive dataset- consisting of known toxins, and (ii) negative dataset- consisting of non-toxins.

### Positive dataset

The positive dataset contained 3,519 toxins retrieved from the T3DB database (http://www.t3db.ca/) (Lim et al., 2010). All metals and small peptides (670 toxins) were removed from the dataset, and the final dataset contained 2,849 toxins. The dataset included toxic compounds with a recorded medical consequence in relatively low concentrations.

### Negative dataset

The negative dataset was constructed using the human metabolites from the RECON1 model in BIGG database (http://bigg.ucsd.edu/data_access) (King et al., 2016). The human metabolites were used since they are produced by the conserved pathways, and had no known reports of showing any human toxicity, and thus can serve as a good negative set, since the aim of the tool was to predict the molecules toxic for the human system. Using the id information available in the RECON1 model, a total of 1,263 unique molecules were retrieved from different sources (Supplementary Text S1). These molecules were considered as the negative dataset in this study.

### Validation datasets

Two validation datasets were used to optimize the performance. First validation set consisted of 41 drugs, which were withdrawn from the commercial market due to their adverse effects. Second validation set consisted of 15 compounds commonly used as additives in food, cosmetics, detergents and preservatives. Though these compounds are available in the commercial market, these are reported to be toxic and their use has been debatable (Gernhardt et al., 2004).

### Compositional analysis

To differentiate between the positive and negative datasets, a compositional analysis was performed using the atomcount function of “ChemmineR” “library” in “R”. It is a physicochemical descriptor, which calculates the elemental composition, and was used to compare the elements occurring in toxins and non-toxins (Cao et al., 2008).

### Input features

#### Descriptors and fingerprints

Descriptors depict all two and three-dimensional properties in the form of numerical values, and thus, it is a form of conceptual projection of all the molecular properties. Molecular descriptors can be categorized into one, two and three-dimensional descriptors. 1D-descriptors contain information on bulk properties such as molecular weight, molar refractivity, permeability and solubility. Physicochemical properties of a molecule such as LogP, LogD, and Topological Polar Surface Area (TPSA) can be estimated based on 2D structures described by the fragmental descriptors. The 1D and 2D descriptors were used in this study. Fingerprints are a complex form of descriptors. They typically encode the information of molecular structures into a bit string, which produces a pattern characteristic of a given molecule. Bit strings account for different structural fragments, and their presence or absence was indicated by 0 or 1. Fingerprint overlap was determined as a measure of similarity and was calculated by Tanimoto coefficient (Dong et al., 2015).

#### Development of classification models for the prediction of toxicity

Fingerprints and descriptors were used as the input feature for the development of various classification models. In total, 10,208 bits belonging to 10 different fingerprints were calculated for 2,843 molecules in positive dataset, and 1,262 molecules in negative dataset using “PaDEL” (Supplementary Text S2; Yap, 2011). The fingerprint dataset (10,208 bits) from 4,105 molecules was divided randomly into 80:20 ratios, from which the 80% part (3,282 molecules) was used for training and the remaining 20% (823 molecules) was used for testing.

In total, 196 descriptors belonging to six different descriptor families (Supplementary Text S3) were calculated using “RDKit” for 2,835 molecules in the positive dataset, and 1,247 molecules in the negative dataset. Descriptors such as MinPartialCharge, MaxPartialCharge, MaxAbsPartialCharge, MaxAbsPartialCharge, and Ipc, were excluded because the values for these descriptors for all the molecules present in the dataset could not be calculated using RDKit. The final dataset consisting of 191 descriptors from 4,082 molecules was divided randomly into 80:20 ratio, from which 80% part (3,263 molecules) was used for training, and the remaining 20% (819 molecules) was used for testing.

#### Principal component analysis (PCA)

We have high-dimensional data represented by several input features. Thus, PCA was carried out to compress the data dimensions by identifying those components, which are distinctly different in the two datasets (Wold et al., 1987).

#### Selection of appropriate machine learning model for classification

10-fold cross validation was performed on training data with descriptor and fingerprints as feature inputs for various machine learning algorithms including SVM, CART, Random Forest and KNN, to compare their performance (Hanley and McNeil, 1982) using “caret” package in R (Liaw and Wiener, 2002).

#### Optimization of various parameters for the development of classification models

The mtry (number of randomly selected variables), ntree (number of trees generated by random forest algorithm) and input feature parameters were optimized using the random forest package in R (version 3.3.2) (Ihaka and Gentleman, 1996). The importance of each fingerprint and descriptor was calculated using the mean decrease in accuracy values at the best mtry value obtained using tuneRF function, which calculates optimal value for mtry for random forest using out of bag error estimate. The Out-of-Bag (OOB) error, which represents the prediction error of random forest algorithm, of fingerprint-based classification models was calculated using top 0.5, 1, 5, 10, and 20% fingerprints from the total 3,282 molecules using various mtry values at ntree = 200–1,000. Similarly, the OOB error performance of descriptor-based classification models was evaluated using top 20, 40, 60, 80, and 100 descriptors derived from the total 3,263 molecules using various mtry values at ntree = 200–1,000. The best model with minimum OOB error from fingerprint-based and descriptor-based classification models was selected. On the basis of performance of these two models, the top 10% (1,021 bits) fingerprints and the top 100 descriptors were combined together to develop a hybrid model with 1,121 input features. Performances of various classification models were optimized using 10-fold cross validation.

#### Performance validation of final classification models

Performances of fingerprint, descriptor and hybrid-based models were evaluated on 20% of the test dataset, and also on two separate validation datasets using the following performance measures.

$$\begin{array}{l} Sensitivity=\frac{TP}{TP+FN}\;Specificity=\frac{TN}{TN+FP}\\ Precision=\frac{TP}{TP+FP}\\ Accurancy=\frac{TP+TN}{TP+FN+FP+TN}\\ MCC=\frac{\left(TP\times TN\right)-\left(FP\times FN\right)}{\sqrt{\left(TP+FP\right)\left(TP+FN\right)\left(TN+FP\right)\left(TN+FN\right)}} \end{array}$$

Where, *TP* = True Positive, *TN* = True Negative, *FP* = False Positive, *FN* = False Negative and *MCC* = Matthews correlation coefficient.

#### Development of regression models for the prediction of solubility and permeability

To examine the toxicity determining factors such as aqueous solubility and caco-2 permeability, additional regression models were developed to calculate the numerical values of LogS and LogP. These values are expected to be high in the case of a toxin and low in the case of a non-toxin (Artursson and Karlsson, 1991).

#### Construction of datasets

To prepare the solubility dataset, a total 452 molecules were considered from the positive and negative dataset for which the experimental values for aqueous solubility were available at the admetSAR dataset (Cheng et al., 2012) and from the work by Palmer et al. (2007). All these molecules are structurally diverse and solid at room temperature (Palmer et al., 2007). To prepare the permeability dataset, 133 molecules from the positive and negative dataset for toxicity classification model were considered for which the experimental values for caco-2 cell permeability were available at admetSAR dataset (Cheng et al., 2012), and from the work by Wang et al. (2016). Both the datasets were divided into two groups in the ratio of 80:20, where the 80% part was used as training dataset and the 20% part was used as testing dataset. These training and testing datasets were used for the construction and validation of the regression models. Final solubility dataset contained 362 molecules in the training set and 90 molecules in the testing set, whereas the final dataset for permeability consisted of 115 molecules in the training dataset, and 18 molecules in the testing dataset.

#### Selection of appropriate machine learning based models

Molecular descriptors have been used as an input feature for the development of various regression models because descriptors have all the molecular information including electronic, topological, thermodynamics and structural properties. Multi-linear regression (MLR), Random Forest Regression (RFR) and Partial Least Square Regression (PLSR) were optimized to calculate the aqueous solubility and caco-2 cell permeability (Schneider et al., 2010).

Multi-linear regression (MLR) fits two or more variables on the experimentally known data by using a linear equation. In general, the linear equation for MLR regression having n observations is *Y* = a_1_X_1_+a_2_X_2_+a_3_X_3_+…….+a_n_X_n_+C. In this case, every independent variable X_i_ (*i* = 1,2,3…,n) is associated with the dependent variable Y according to the above equation. R^2^ was used for LogS and LogP estimation using MLR algorithm (Kujawski et al., 2012). For the development of final models, the best descriptors were selected based on their minimum *p*-values.

The RFR models for both datasets were trained using randomForest package in R. Models were constructed using mtry = 14, step = 0.5, improvement factor = 10^−5^ and ntree = 100. Variable importance information for each descriptor was obtained from the random forest models. For the development of final models, the best descriptors were selected based on their mean decrease in accuracy values.

Partial Least Square regression (PLSR) is a widely used predictive modeling method to deal with highly collinear predictor variables (Van Huffel, 1997). In this model, it is considered that all the independent variables (*X*_*i*__=_ descriptors) are linearly related to the dependent variable (*Y* = LogS/LogP) (Geladi and Kowalski, 1986). Variable selection was performed with the help of plsgenomics library and variable. selection function (Kotsiantis et al., 2007).

## Results

### Compositional analysis reveals the difference between toxin and non-toxins

The compositional analysis revealed that non-toxic molecules were rich in Carbon, Oxygen, Nitrogen, and Phosphorus, which are the building blocks of living matter (Supplementary Figure S1). In contrast, the toxins were rich in Chlorine, Bromine, Arsenic, Lead, Cobalt and Fluorine in addition to Carbon and Oxygen (Supplementary Figure S2; Data Sheet 2). The atom frequency of compositional elements showed clear differences between toxins and non-toxins, which suggests that composition and related properties are important determinants of the toxicity of a compound.

### Principal component analysis

To compute variance between toxins and non-toxins, PCA was performed on training data using fingerprints and descriptors as input features (Supplementary Figure S3 and Supplementary Text S4).

### Selection of appropriate machine learning method for classification

**Figure 1:**
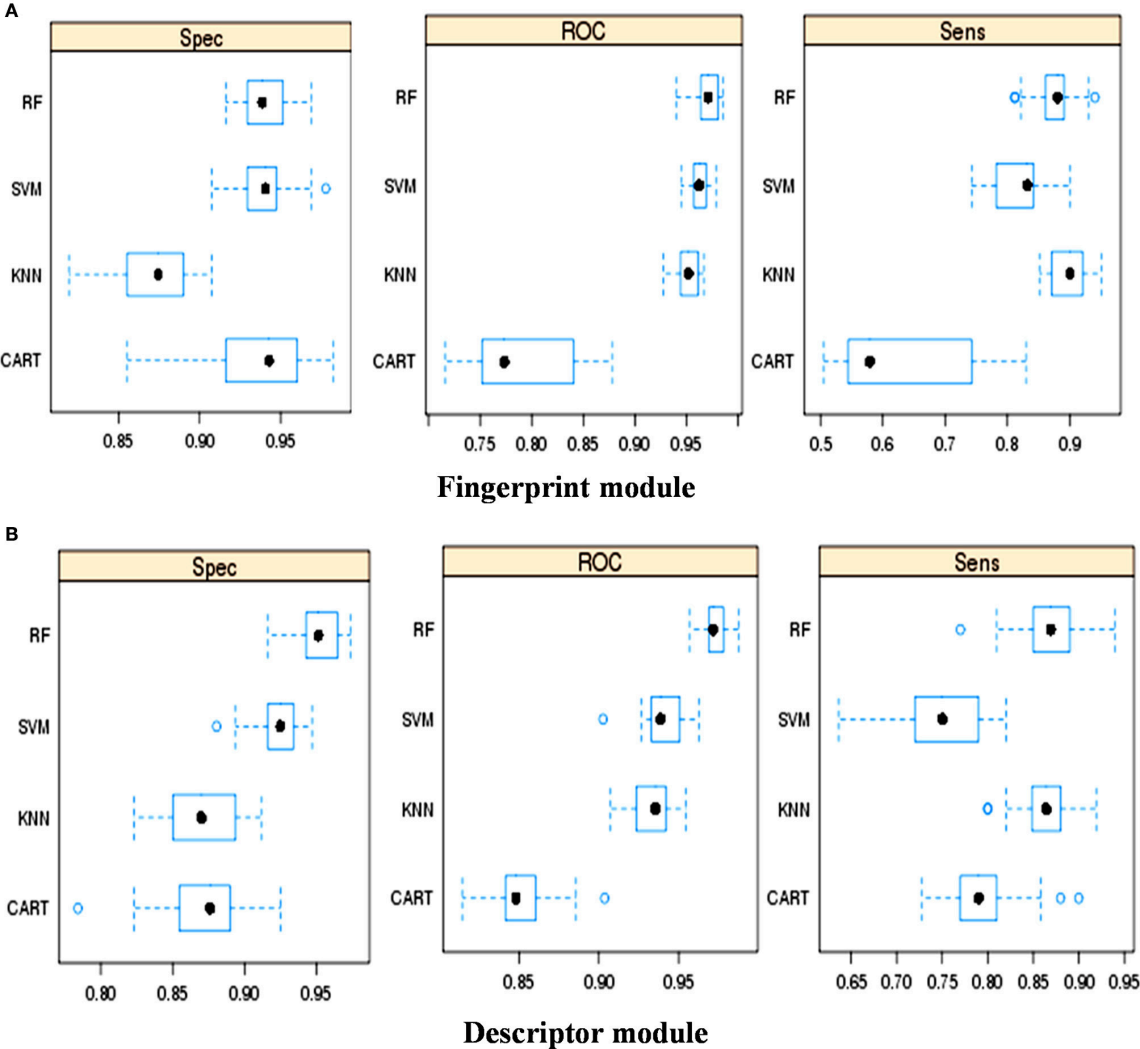
Performance of different machine learning methods for the classification of toxins and no-toxins, **(A)** Using fingerprints as input feature, and **(B)** Using descriptors as input features.

The classification performance of various machine learning methods was evaluated using “caret” package in R script (Kotsiantis et al., 2007). Using fingerprints as input features (Figure 1A) ROC (Receiver Operating Characteristic) values displayed by RF, SVM, KNN, and CART were 0.97, 0.96, 0.95, and 0.77, respectively. Similarly, using descriptors as the input features, the ROC values displayed by RF, SVM, KNN, and CART were 0.97, 0.94, 0.94, and 0.85, respectively (Figure 1B). Thus, it was apparent that the RF-based model outperformed the other machine learning based models.

### Optimization of RF parameters and development of classification models

Mean decrease in accuracy values for each variable in descriptors and fingerprints were calculated at the best mtry (optimized by tuneRF function) and ntree = 500. The mean decrease in accuracy values for the top 30 fingerprints and descriptors are shown in Supplementary Figures S4A,B, and the complete list of fingerprints and descriptors with the mean decrease in accuracy values are provided in Supplementary Tables S1, S2.

**Figure 2:**
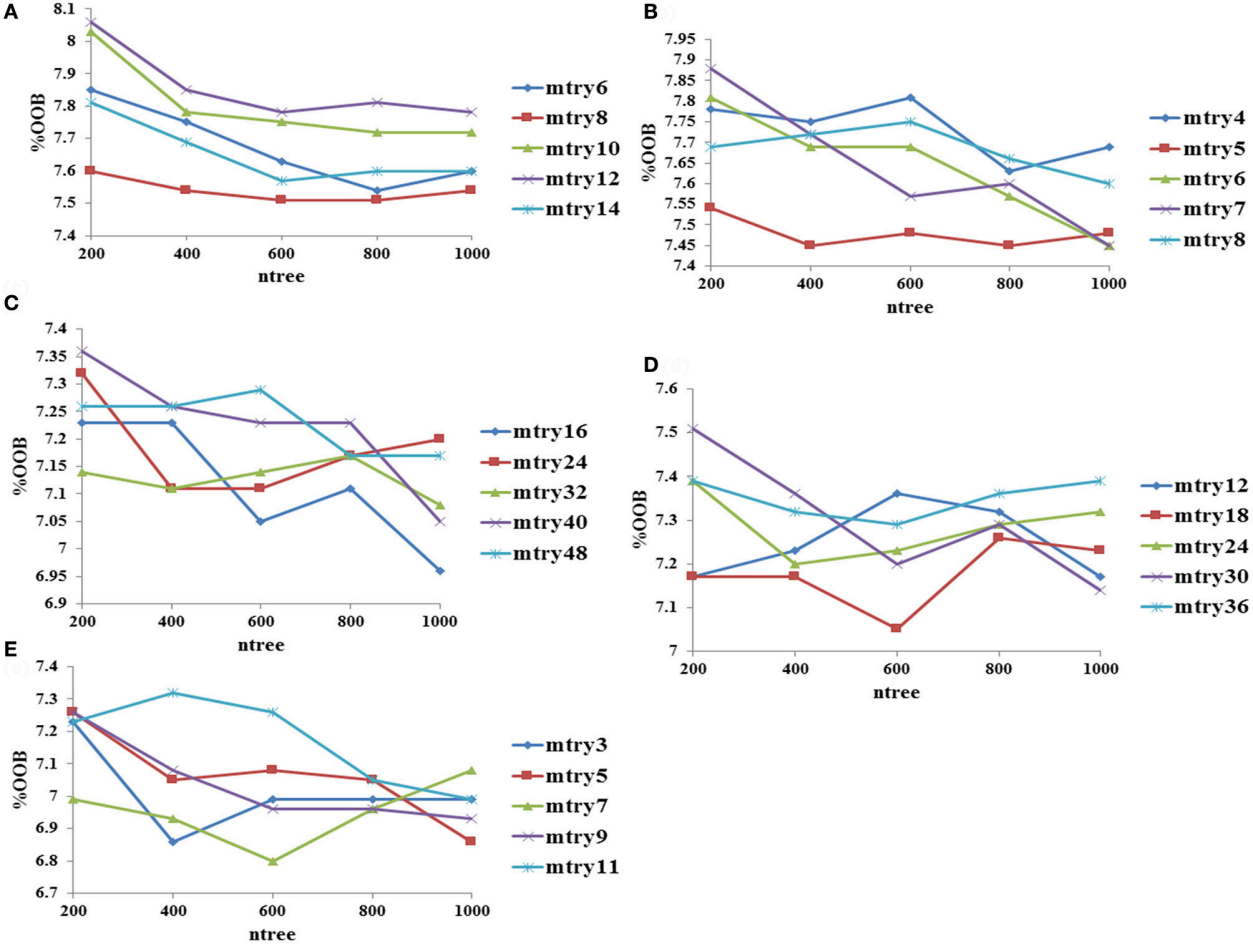
Optimization of random forest at various mtry and ntree values using descriptors as input features, **(A)** performance using top 20 descriptors, **(B)** performance using top 40 descriptors, **(C)** performance using top 60 descriptors, **(D)** performance using top 80 descriptors, and **(E)** performance using top 100 descriptors.

**Figure 3:**
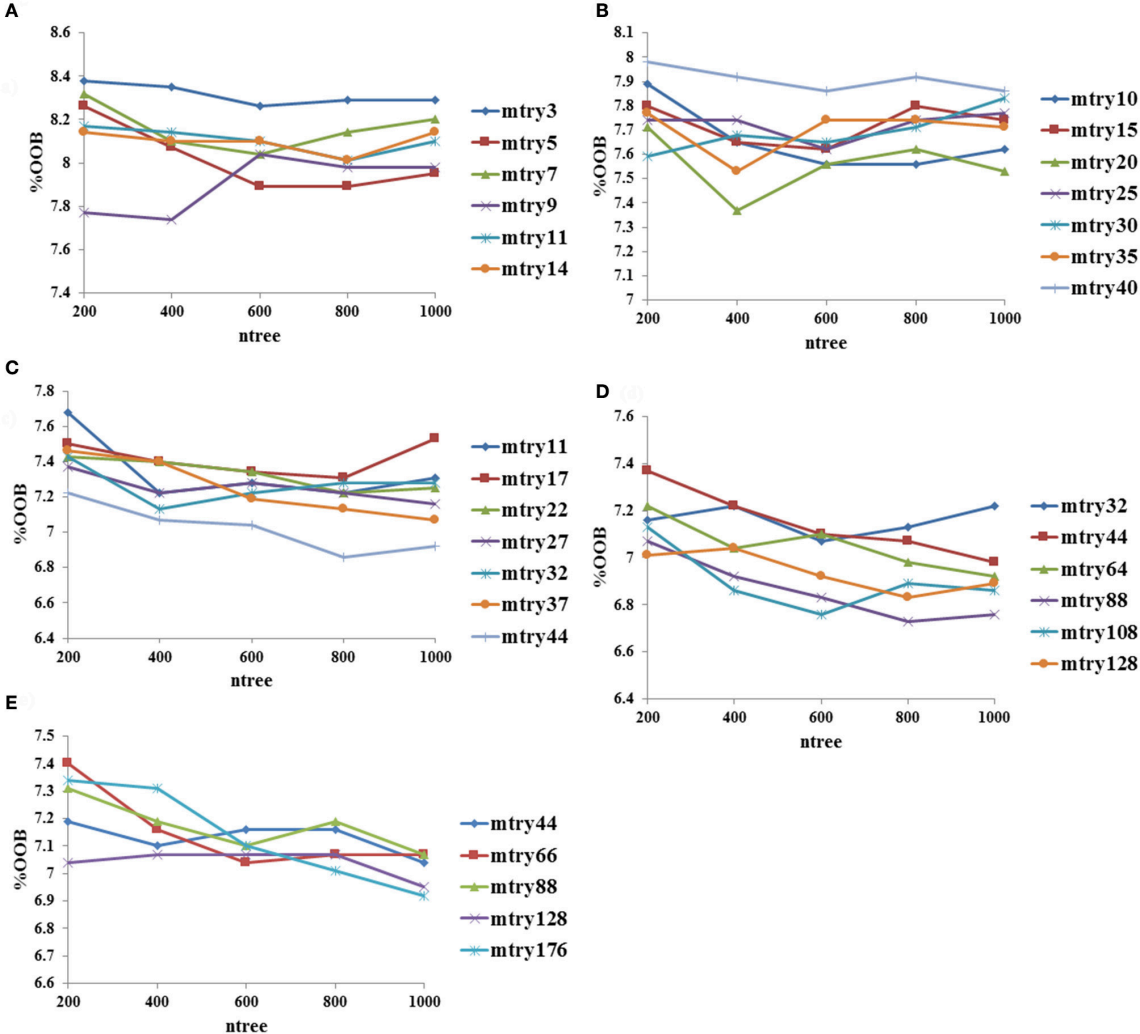
Optimization of random forest at various mtry and ntree values using fingerprints as input features, **(A)** performance using top 0.5% fingerprints, **(B)** performance using top 1% fingerprints, **(C)** performance using top 5% fingerprints, **(D)** performance using top 10% fingerprints, and **(E)** performance using top 20% fingerprints.

The performance of descriptor-based and fingerprint-based classification models was examined at various mtry values and ntree = 200–1,000 using different combinations of descriptors and fingerprints. From Table 1 and Figure 2, it is apparent that the model developed using the top 100 descriptors at mtry = 5 and ntree = 1,000 performed better than the other classification models and showed an accuracy of 0.93, MCC of 0.84, and the lowest OOB error of 6.9%. From Table 2 and Figure 3, it is apparent that the model developed using the top 10% (1,021 bits) of fingerprints at mtry = 88 and ntree = 800, performed better than other classification models as it displayed an accuracy of 0.93, MCC of 0.84 and the lowest OOB error of 6.7%. A hybrid set was constructed using the top 100 descriptors and 1,021 fingerprints, and the performance of RF-based classification model was evaluated using the optimized parameters for fingerprint-based model (mtry = 88 and ntree = 800). The performance of the hybrid model was almost similar to the performance of fingerprint-based model, and showed the Sensitivity, Specificity, Precision, Accuracy and MCC of 0.95, 0.89, 0.95, 0.93, and 0.84, respectively. From above, the best performing fingerprint-based, descriptor-based and hybrid models were selected as the final models for the classification of molecules as toxic or non-toxic.

**Table 1:** Performance of random forest models at difference mtry values using variable number of important descriptors.

**Descriptors**	**mtry**	**Sensitivity**	**Specificity**	**Precision**	**Accuracy**	**MCC**
Top20	6	0.9429	0.8802	0.9479	0.9240	0.8203
	8	0.9434	0.8812	0.9484	0.9246	0.8218
	10	0.9421	0.8782	0.9470	0.9228	0.8174
	12	0.9418	0.8927	0.9542	0.9272	0.8272
	14	0.9436	0.8734	0.9444	0.9222	0.8165
Top40	4	0.9421	0.8791	0.9475	0.9231	0.8181
	5	0.9434	0.8830	0.9492	0.9252	0.8231
	6	0.9431	0.8847	0.9501	0.9255	0.8237
	7	0.9454	0.8800	0.9475	0.9255	0.8243
	8	0.9433	0.8794	0.9475	0.9240	0.8204
Top60	16	0.9477	0.8905	0.9523	0.9304	0.8356
	24	0.9468	0.8848	0.9497	0.9280	0.8300
	32	0.9480	0.8861	0.9501	0.9292	0.8330
	40	0.9477	0.8878	0.9510	0.9295	0.8335
	48	0.9472	0.8850	0.9497	0.9283	0.8307
Top80	12	0.9468	0.8857	0.9501	0.9283	0.8306
	18	0.9448	0.8879	0.9515	0.9277	0.8289
	24	0.9455	0.8836	0.9492	0.9268	0.8270
	30	0.9476	0.8851	0.9497	0.9286	0.8315
	36	0.9439	0.8849	0.9501	0.9261	0.8253
Top100	3	0.9473	0.8904	0.9523	0.9301	0.8348
	5	0.9470	0.8948	0.9545	0.9314	0.8375
	7	0.9469	0.8884	0.9515	0.9292	0.8327
	9	0.9474	0.8922	0.9532	0.9307	0.8362
	11	0.9473	0.8904	0.9523	0.9301	0.8348

*Top100 at mtry 5 was selected to be the best performing model to build the descriptor-based module for ToxiM as the model showed the best performance among all the models developed during the variable optimization step for descriptors*.

**Table 2:** Performance of random forest models at difference mtry values using variable number of important fingerprints.

	**mtry**	**Sensitivity**	**Specificity**	**Precision**	**Accuracy**	**MCC**
Train0.5per	3	0.9296	0.8867	0.9525	0.9171	0.803
	5	0.9359	0.884	0.9503	0.9205	0.8117
	7	0.9376	0.873	0.9446	0.918	0.8067
	9	0.9428	0.8694	0.9419	0.9202	0.8126
	11	0.9423	0.8667	0.9406	0.919	0.8099
	14	0.9422	0.8659	0.9402	0.9186	0.8092
Train1per	10	0.9454	0.8754	0.9446	0.9238	0.8212
	15	0.9457	0.8712	0.9424	0.9226	0.8187
	20	0.949	0.8713	0.9419	0.9247	0.8242
	25	0.9476	0.8667	0.9397	0.9223	0.8186
	30	0.9472	0.8658	0.9393	0.9217	0.8171
	35	0.9469	0.8699	0.9415	0.9229	0.8197
	40	0.9472	0.8649	0.9388	0.9214	0.8165
Train5per	11	0.9504	0.8751	0.9437	0.9267	0.8291
	17	0.9486	0.8721	0.9424	0.9247	0.824
	22	0.9496	0.8783	0.9454	0.9275	0.8302
	27	0.9493	0.8817	0.9472	0.9284	0.8321
	32	0.9484	0.8797	0.9463	0.9272	0.8292
	37	0.9494	0.8843	0.9485	0.9293	0.8341
	44	0.9519	0.8841	0.9481	0.9308	0.838
Train10per	32	0.9508	0.877	0.9446	0.9278	0.8312
	44	0.9514	0.8831	0.9476	0.9302	0.8366
	64	0.9511	0.8856	0.949	0.9308	0.8378
	88	0.9524	0.8877	0.9498	0.9324	0.8414
	108	0.9515	0.8866	0.9494	0.9314	0.8392
	128	0.9515	0.8857	0.949	0.9311	0.8386
Train20per	44	0.9518	0.8806	0.9463	0.9296	0.8353
	66	0.9506	0.882	0.9472	0.9293	0.8344
	88	0.9502	0.8828	0.9476	0.9293	0.8343
	128	0.9506	0.8855	0.949	0.9305	0.837
	176	0.9507	0.8864	0.9494	0.9308	0.8377

*Top 10% fingerprints at mtry 88 were selected to be the best performing model to build the fingerprint-based module for ToxiM, as this model showed the best performance among all the models developed during the variable optimization step for fingerprints*.

### Identification of statistical significant descriptors and fingerprints

**Figure 4:**
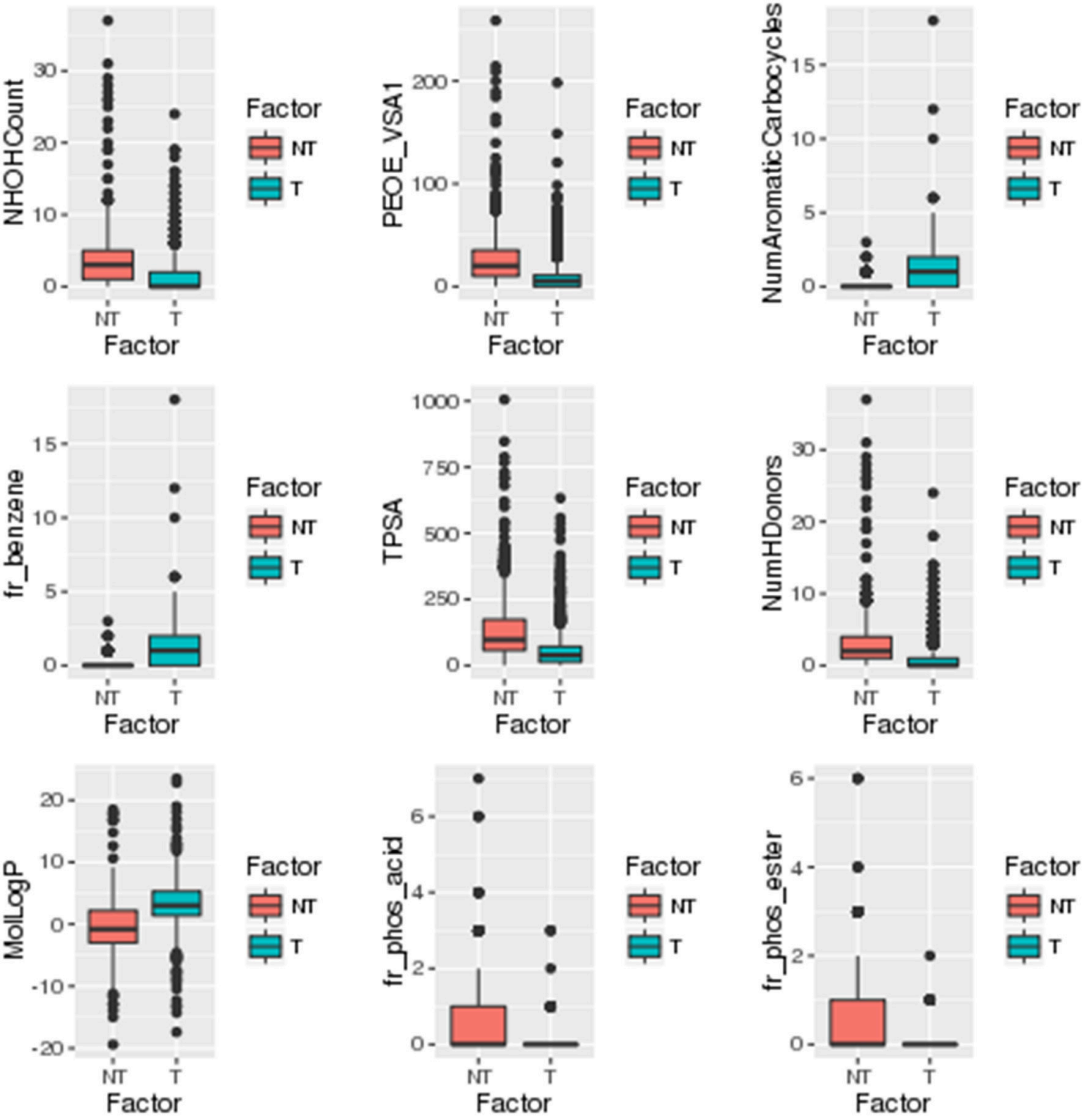
Distribution of significantly discriminating descriptors (Wilcoxon test, *p* < 0.05) among toxins (T) and non-toxins (NT).

**Figure 5:**
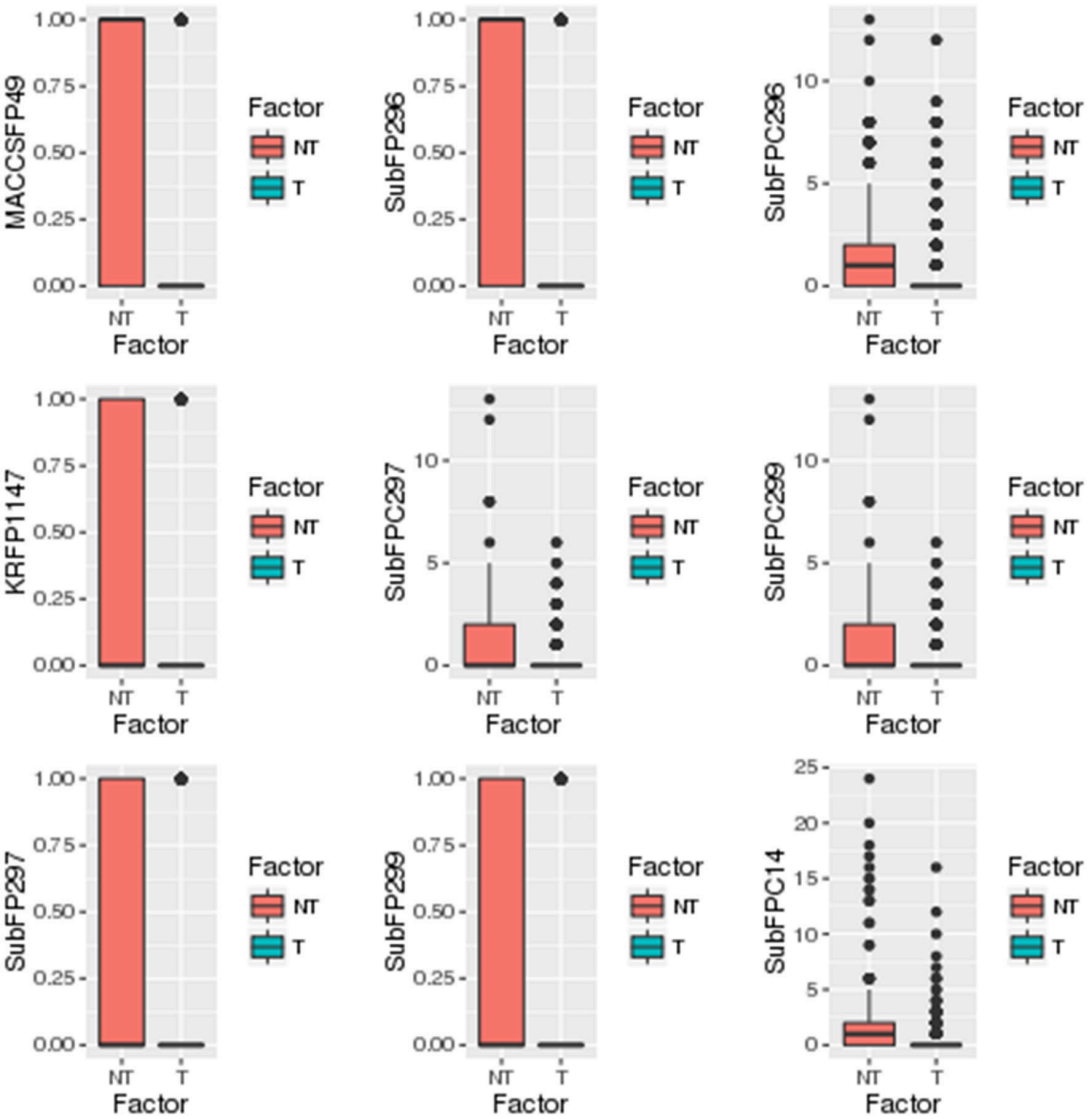
Distribution of significantly discriminating fingerprints (Wilcoxon test, *p* < 0.05) among toxins (T) and non-toxins (NT).

A separate statistical analysis (Wilcoxon rank sum test) was performed on the training datasets of top 100 descriptors and top 1,021 fingerprints to find out the significantly discriminating (*P* ≤ 0.05) descriptors and fingerprints among toxins and non-toxins. The distribution of top descriptors and fingerprints in toxins and non-toxins are shown in Figures 4, 5, respectively. From the results, it is apparent that the proportion of these descriptors and fingerprints varied considerably from toxins to non-toxins. Detailed characterization of the discriminating features for toxin-like property in any molecule will be of great use for the researchers working in the toxicology field.

### Performance evaluation of RF classification models using blind set

**Figure 6:**
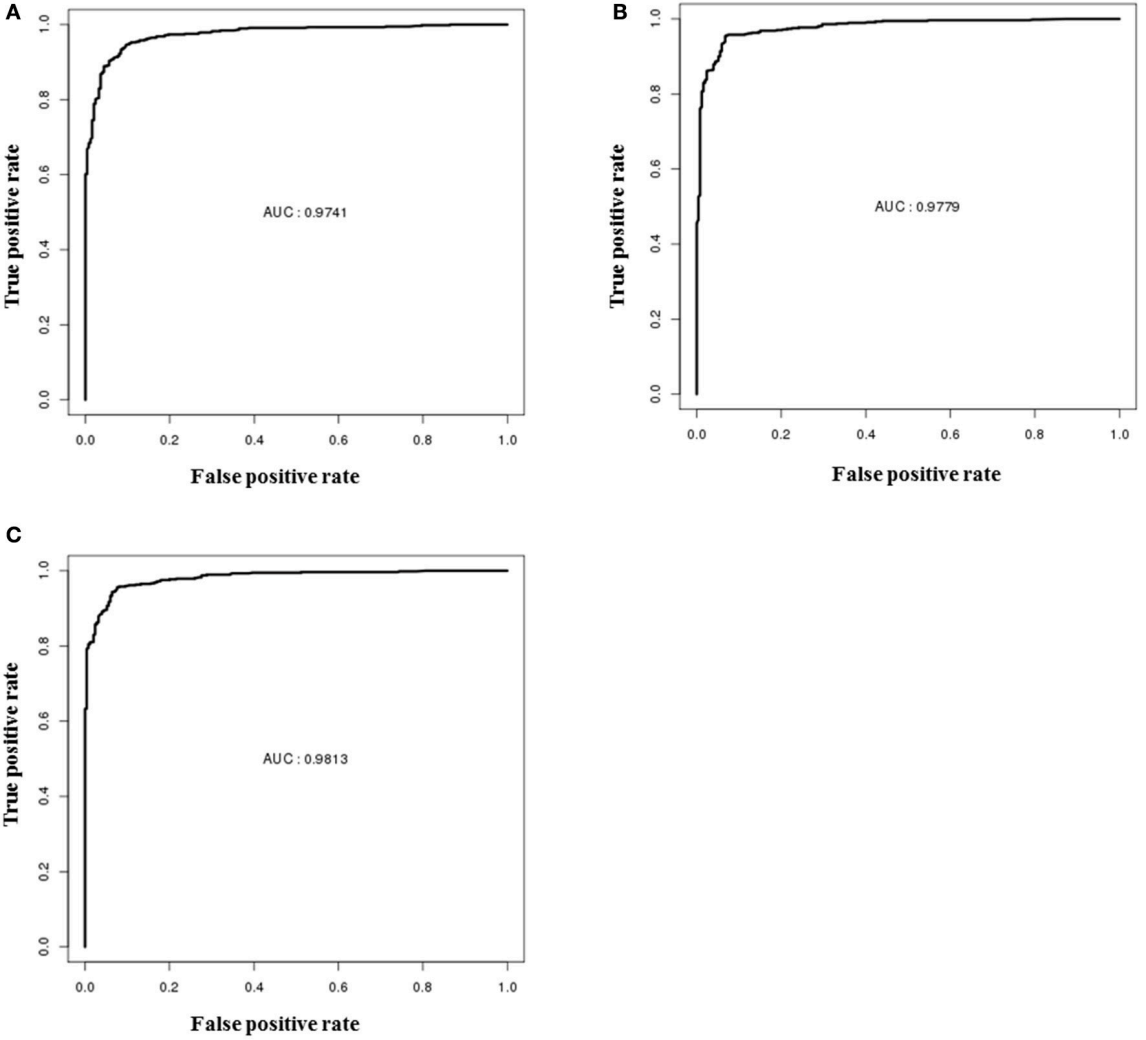
ROC performances of random forest models on validation dataset, **(A)** performance of the descriptor-based model, **(B)** performance of the fingerprint-based model, and **(C)** performance of the hybrid-based model.

The performances of descriptor-based, fingerprint-based and hybrid models were evaluated using the blind data set (methods section). From Figure 6, it is apparent that all the models showed very high true positive rates as compared to the false positive rates. The ROC values shown by the hybrid model (Figure 6C) was slightly higher (0.98) than the other two fingerprints (Figure 6B) and descriptors (Figure 6A) models (0.97). The detailed performances of each model are provided in Table 3.

**Table 3:** Performance of all three models on blind dataset.

**Model**	**Sensitivity**	**Specificity**	**Precision**	**Accuracy**	**MCC**
Descriptors	0.95	0.88	0.95	0.93	0.84
Fingerprints	0.96	0.92	0.96	0.95	0.87
Hybrid	0.96	0.88	0.95	0.94	0.85

### Development and validation of regression models

In addition to the prediction of toxicity of compounds, MLR, RF, and PLS based regression models were also developed to calculate the solubility and permeability values using descriptors as the input feature. To construct MLR-based regression models, top 15 descriptors were selected (based on their importance) to calculate LogS and top 11 descriptors were selected to calculate LogP (Supplementary Tables S3, S4). Similarly, to construct RF-based regression models, top 40 most important descriptors were selected to calculate LogS, and top 10 descriptors were selected to calculate LogP (Supplementary Tables S5, S6). To construct PLS-based regression model, top five descriptors were used to calculate LogS and LogP (Supplementary Tables S7, S8). The performance of each model was evaluated using R^2^ values, which is a statistical measure of how close the data points are fitted on the regression line.

**Figure 7:**
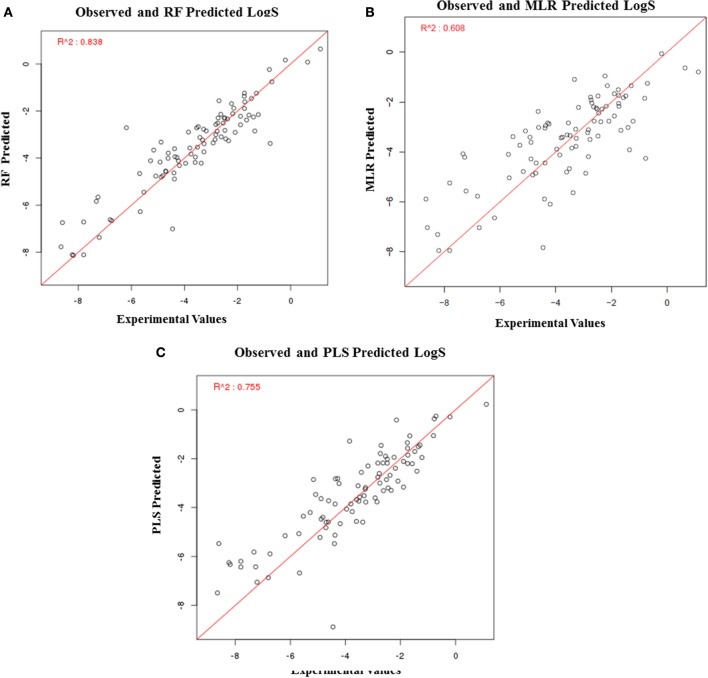
Performance of regression-based models for prediction of LogS, **(A)** RF-based regression model, **(B)** MLR-based regression model, and **(C)** PLS-based regression.

**Figure 8:**
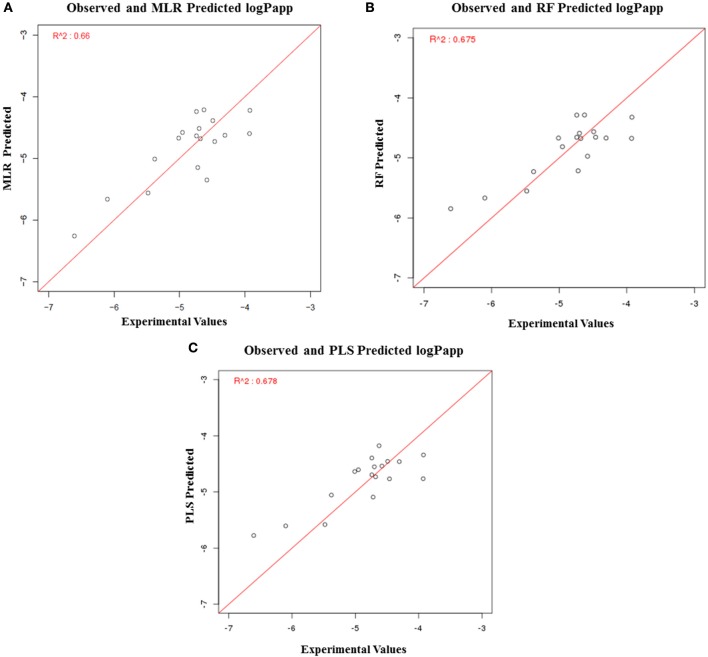
Performance of regression-based models for prediction of LogP, **(A)** MLR-based regression model, **(B)** RF-based regression model, and **(C)** PLS-based regression model.

The solubility predictive ability of RF-based regression model was higher (*R*^2^ = 0.84) as compared to the ML-based regression model (*R*^2^ = 0.61) and PLS regression model (*R*^2^ = 0.76) (Figure 7). Therefore, the RF-based model was selected for validation, which displayed a correlation accuracy of 0.92 on the blind dataset (Table 4). The permeability predictive ability of PLS-based regression model was marginally higher (*R*^2^ = 0.678) as compared to the RF-based regression model (*R*^2^ = 0.675) and ML regression model (*R*^2^ = 0.66) (Figure 8). Therefore, the PLS-based regression model was selected for validation, which performed with a correlation accuracy of 0.82 on the blind dataset (Table 5).

**Table 4:** Experimental and predicted values of LogS on the blind set for solubility.

**Molecule Name**	**Observed LogS**	**Predicted LogS**
Dicofol	−0.2	0.16
1,1,2,2-Tetrachloroethane	−2.45	−3.18
1,1-Dichloroethane	−4.883	−3.32
1,1,2-Trichloroethane	−5.08	−4.77
1,2-Diphenylhydrazine	−5.16	−3.66
1-Bromopropane	−7.21	−7.38
Tefluthrin	−2.38	−2.34
1,2,4-Trichlorobenzene	−1.219	−2.15
Benzo[e]pyrene	0.64	0.08
2,4-Dichlorophenoxyacetic acid	−2.478	−2.82
Dapsone	−2.507	−2.29
Pyrene	−4.71	−4.55
Styrene	−2.92	−3.36
Hexachlorobutadiene	−3.26	−3.39
Alprazolam	−3.48	−2.67
Prochlorperazine	−4.19	−4.32
Meperidine	−3.27	−3.74
Imipramine	−2.77	−2.86
Metharbital	−4.92	−4.17
Cimetidine	−1.75	−1.38
Procaine	−3.18	−2.85
Diazepam	−3.752	−3.83
Nitrazepam	−2.635	−2.14
Strychnine	−4.4	−4.63
Bromadiolone	−3.54	−2.72
Pentane	−3.96	−4.22
2,3-Dimethylpentane	−2.144	−1.88
3,3-Dimethylpentane	−5.666	−6.28
2,2,3-Trimethylbutane	−1.89	−2.59
2-Methylheptane	−4.61	−3.79
2-Pentene	−2.617	−3.10
2-Methyl-1-butene	−2.73	−2.30
Isopropylbenzene	−5.28	−4.12
2-Nitrophenol	−4.23	−4.12
4-Aminophenol	−8.23	−8.11
Benfluralin	−7.8	−8.11
Chlorpyrifos-methyl	−0.77	−3.38
Iprodione	−6.74	−6.66
Niclosamide	−8.65	−7.77
Oxadiazon	−2.7	−1.57
Prometon	−3.28	−2.78
4-Phenylphenol	−6.8	−6.62
Propylparaben	−2.09	−2.91
Adenine	−2.338	−3.27
Cholic acid	−1.74	−1.90
D-Fructose	−1.29	−1.25
L-Tryptophan	−4.7	−4.58
Succinic acid	−3.42	−3.12
Atrazine	−4.376	−3.61
Clomazone	−4.38	−4.89
Diphenylamine	−2.75	−2.52
Ethofumesate	−2.48	−2.31
Ethylenethiourea	−8.19	−8.14
Fenarimol	−4.28	−3.98
3-Chlorobiphenyl	−2.82	−2.57
Oryzalin	−1.73	−1.61
Picloram	−4.88	−4.81
Terbacil	−1.57	−2.18
Dodecanoic acid	−4.36	−3.95
Equilin	−1.74	−1.24
Griseofulvin	−7.321	−5.84
Estrone	−1.35	−2.85
Hydroxyurea	−3.59	−3.96
1-Hexadecanol	−8.6	−6.74
Coumarin	−6.19	−2.71
Benzoin	−3.51	−3.54
1-Nitronaphthalene	1.12	0.63
2,3′,4,4′-Tetrachlorobiphenyl	−2.82	−3.01
2,2′,3,4,5-Pentachlorobiphenyl	−3.32	−3.24
1,2-Dibromo-3-chloropropane	−1.89	−2.23
2,2′,3,3′,6,6′-Hexachlorobiphenyl	−0.8	−0.24
Vinyl chloride	−5.53	−5.45
Tetracene	−3.6	−4.18
Triphenylene	−2.23	−1.69
Dichlorprop	−5.69	−4.66
2-Methyl-4-chlorophenoxyacetic acid	−3.85	−2.90
Alachlor	−3.8	−3.58
Amygdalin	−1.4	−2.25
Mirex	−2.183	−2.11
Asulam	−7.8	−6.72
Benfuracarb	−4.62	−4.01
Benomyl	−4.45	−7.01
Carbaril	−2.54	−2.51
Chlorbufam	−7.26	−5.66
2,4-Dinitrophenol	−2.85	−3.21
Dioxacarb	−3.37	−4.22
Benzo[a]pyrene	−4.82	−4.75
Pyrolan	−1.48	−1.47
Ethylbenzene	−0.71	−0.76
Benzo[b]fluoranthene	−1.66	−2.38

**Table 5:** Experimental and predicted values of LogP of blind set for permeability.

**Molecule Name**	**Observed LogP**	**Predicted LogP**
Glipizide	−4.58	−4.54
Ceftriaxone	−4.46	−4.77
Dextromethorphan	−4.74	−4.40
Olopatadine	−4.72	−5.09
Sulfasalazine	−4.49	−4.46
Diazepam	−4.74	−4.70
Oxazepam	−4.95	−4.60
Diflunisal	−5.48	−5.58
Thalidomide	−4.70	−4.55
Verapamil	−4.31	−4.46
Daidzein	−6.60	−5.78
Vinblastine	−3.92	−4.34
Griseofulvin	−4.68	−4.73
Diclofenac	−5.01	−4.64
Cytarabine	−4.63	−4.18
Methanol	−3.93	−4.76
choline	−5.38	−5.06
progesterone	−6.10	−5.60

### Development of toxim webserver for the prediction of toxicity

**Figure 9:**
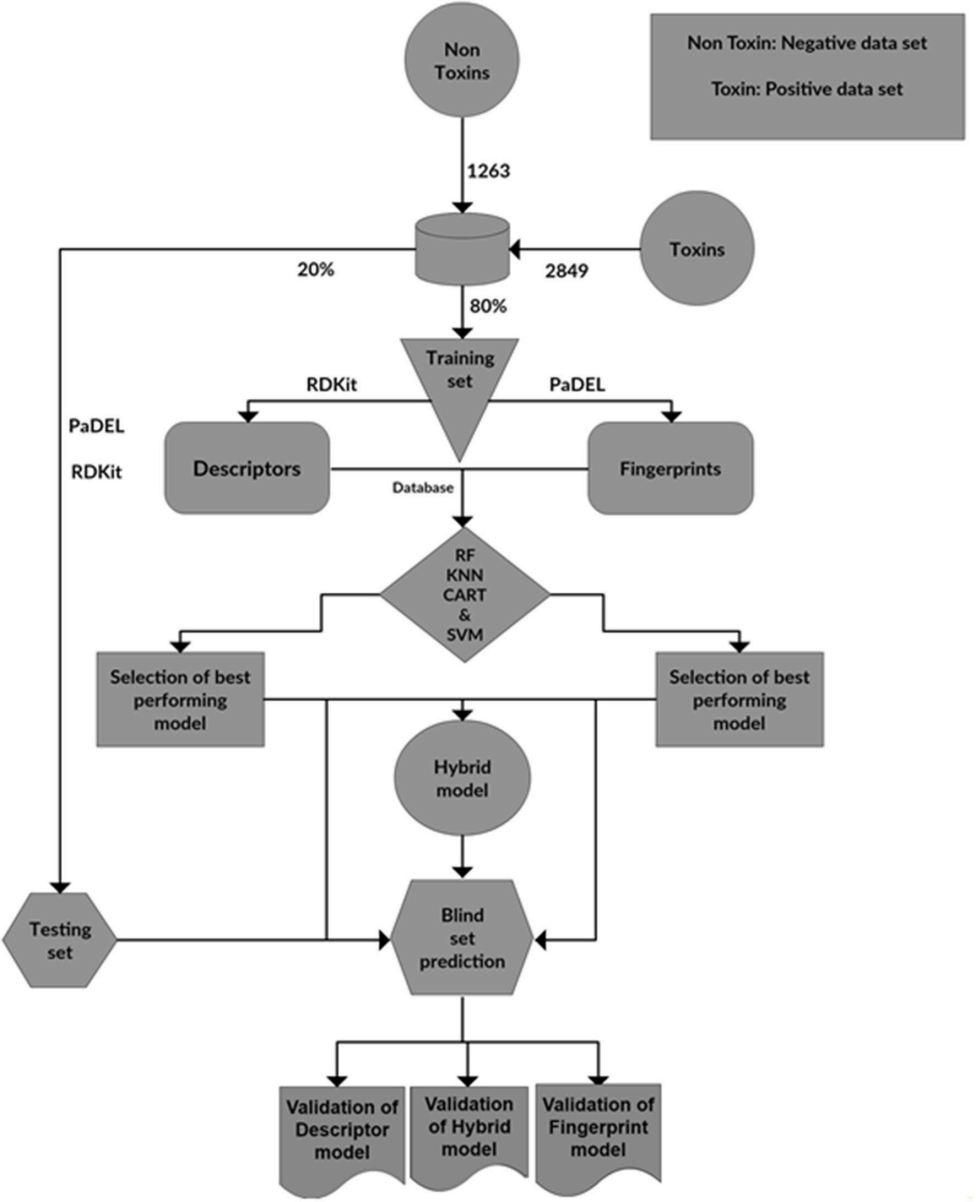
The steps involved in the construction of ToxiM classification models for the prediction of toxicity of molecules.

The steps involved in the construction of ToxiM classification models for the prediction of toxicity of molecules is represented in Figure 9. Using the final classification and regression models, a web server ToxiM was developed to facilitate the online submission of a query by the user, and to display the prediction results. The toxicity prediction page enables the user to submit the query molecule either by the PubChem CID or by uploading its SDF file. The query SDF file is processed through the models available at the web server for the prediction of toxicity, permeability and solubility properties. All the three best performing models, i.e., fingerprint-based, descriptor-based and hybrid-based are available at the webserver for selection. The query is analyzed through the selected model, and the results are displayed in the form of prediction probabilities for the classification of a query as toxic or non-toxic. To examine the solubility and permeability of a given query molecule, descriptor-based best performing models, the RF-based regression model for the calculation of LogS, and the ML-based regression model for the calculation of LogP, are available as the default options. A tutorial page is provided to explain the navigation through the website. The webserver can be accessed at http://metagenomics.iiserb.ac.in/ToxiM/.

### Performance validation of toxim using validation datasets

The performance of ToxiM was evaluated on two validation datasets. The first dataset consisted of 41 drugs, which were withdrawn due to their potentially harmful effects. The second dataset consisted of 15 commonly used molecules but their toxicity to humans have been debatable (Wishart et al., 2006). ToxiM predicted all the withdrawn drugs to be toxic with probability score >0.5, and could successfully validate the toxicity of these drugs (Supplementary Table S9). For withdrawn drugs, toxicity prediction probability scores of ToxiM were higher in comparison to the admetSAR (Table 6). The predictions of ToxiM on the second validation set are provided in Supplementary Table S10. In addition to the toxicity prediction of withdrawn drugs and other molecules, LogP and LogS values were also calculated using the regression analysis on both the sets. The results for LogP and LogS for FDA withdrawn molecules are given in Supplementary Tables S11, S12, respectively. Similarly, the values of LogP and LogS calculated for the second dataset are given in Supplementary Tables S13, S14, respectively.

**Table 6:** Toxicity prediction performance of admetSAR and ToxiM.

**Smiles**	**admetSAR scores**	**ToxiM Scores**	**Names**	**Type of Toxicity**
OC(= O)CCCCCCNC1c2c(CCc3c1cccc3)cccc2	0.4559	0.831	Amineptine	hepatotoxicity
Clc1cn2c(CC(= O)N(CCC)CCC)c(nc2cc1)c1ccc(Cl)cc1	0.4837	0.997	Alpidem	hepatotoxicity
S = C(N(CC(= O)O)C)c1c2c(c(c(OC)cc2)C(F)(F)F)ccc1	0.5253	0.947	Tolrestat	Failed phase III
O = C1c2c(C(= O)c3c1c(O)ccc3)cccc2O	0.5401	0.943	Danthron	human carcinogen
Clc1ccc(CC(NC(= O)OCC)(C)C)cc1	0.5445	0.995	clophorex	
P(= O)(O)(O)O.N(C(C)C)c1ncccn1	0.5563	0.684	IsaxonineÊPhosphate	hepatotoxicity
FC(F)(F)c1cc(N2CCN(CC2)CCOC(= O)c2c(Nc3c4c(ncc3)cc(cc4)C(F)(F)F)cccc2)ccc1	0.5568	0.953	Antrafenine	
S(= O)(= O)(O)C.Fc1c(N2C[C@H]3[C@H](C3NC(= O)[C@@H](NC(= O)[C@@H](N)C)C)C2)nc2n(cc(c(= O)c2c1)C(= O)O)c1c(F)cc(F)cc1	0.5706	0.785	Trovafloxacin	hepatotoxicity
Clc1c(ccc(OCC(= O)O)c1Cl)C(= O)c1sccc1	0.5742	0.934	Ticrynafen	hepatotoxicity
Clc1c(onc1C)NS(= O)(= O)c1c(scc1)C(= O)Cc1c(cc2OCOc2c1)C	0.5746	0.979	sitaxsentanÊ	hepatotoxicity
O1C(CC(= CC1 = O)OC)/C = C/C1 = CCCC = C1	0.5873	0.95	Kava	hepatotoxicity
O = C(N1[C@@H](CC1)C(= O)NCc1ccc(cc1)/C(= N/O)/N)[C@H](NCC(= O)OCC)C1CCCCC1	0.603	0.886	Ximelagatran	hepatotoxicity
O(CC(NN)C)c1ccccc1	0.6151	0.738	Fenoxypropazine	hepatotoxicity
Brc1ccc(S(= O)(= O)N/C = N/CCSCc2nc(sc2)N = C(N)N)cc1	0.6167	0.896	Ebrotidine	ToxicityÊÊon reproduction
OC(CC(= O)Nc1ccc(OCC)cc1)C	0.6174	0.883	Bucetin	carcinogenesis
Ic1c(O)c2ncccc2c(Cl)c1	0.6223	0.99	Clioquinol	emaciation
O(c1ccc(C(= C2CCCCC2)c2ccc(OC(= O)C)cc2)cc1)C(= O)C	0.6307	0.961	cyclofenil	underlying heartÊconditions
O(C(Nc1ccc(cc1)C(= O)O)C(= O)c1ccc(cc1)c1ccccc1)CC	0.6483	0.918	XenazoicÊAcid	hepatotoxicity
O = C1N(N(C(= O)C1CCCC)c1ccccc1)c1ccccc1	0.6583	0.99	phenylbutazone	hepatotoxicity
O(CCCC)C(= O)c1ccc(N)cc1	0.6807	0.964	Butamben	CNS and cardiac effects
O1CCN(CC(OC(= O)c2cc(OC)c(OC)c(OC)c2)COCCC(C)C)CC1	0.71	0.932	Amoproxan	Dermatologic and ophthalmic toxicity
Clc1ccc(CC(N)(C)C)cc1	0.7205	0.954	Chlorphentermine	Cardiovascular Toxicity
N(= C(\N = C(N)N)/N)/CCCC	0.7374	0.581	Buformin	SevereÊlactic acidosis
Clc1c(N2CC = CC2)ccc(C(C)C(= O)O)c1	0.7379	0.959	Pirprofen	Liver Toxicity
O1C(c2ccccc2)C(= O)N = C1N	0.7407	0.743	Pemoline	Hepatotoxicity
O = C1Nc2c(C1(c1ccc(O)cc1)c1ccc(O)cc1)cccc2	0.741	0.957	Oxyphenisatin	Hepatotoxicity
O = C1N(c2c(C1(c1ccc(OC(= O)C)cc1)c1ccc(OC(= O)C)cc1)cccc2)C(= O)C	0.7436	0.971	Phenisatin	Hepatotoxicity
Clc1ccc(CCC2N(CCc3c2cc(OC)c(OC)c3)C)cc1	0.7469	0.998	Metofoline	Unspecific experimental toxicity
O = C1N(Cc2c1cccc2)c1ccc(C(C)C(= O)O)cc1	0.7493	0.903	Indoprofen	Animal carcinogenicity, gastrointestinal toxicity
[O-][N+](= O)c1nc(n(c2ccc([N+](= O)[O-])cc2)c1)C	0.7522	0.959	Nitrefazole	Hepatic and hematologic toxicity
Fc1c2n(C3CC3)cc(c(= O)c2c(N)c(F)c1N1C[C@@H](N[C@@H](C1)C)C)C(= O)O	0.7719	0.895	Sparfloxacin	QT prolongation and phototoxicity
O = C(NCc1ccccc1)CCNNC(= O)c1ccncc1	0.7767	0.896	Nialamide	Hepatotoxicity
Clc1ccc(OCC(= O)N2CCN(CC2)Cc2cc3OCOc3cc2)cc1	0.7791	0.997	Fipexide	Hepatotoxicity
S(c1ccc(C(O)C(NCCCCCCCC)C)cc1)C(C)C	0.7826	0.807	Suloctidil	OralÊtoxicityÊis very low but is much higher with intravenous
O(c1nn(c2c1cccc2)Cc1ccccc1)CC(= O)O	0.7859	0.938	Bendazac	Hepatotoxicity
Brc1ccc(/C(= C/CN(C)C)/c2cccnc2)cc1	0.7868	0.997	Zimeldine	
O = C1N(N(C(= O)C1CC = C(C)C)c1ccccc1)c1ccccc1	0.8006	0.984	Feprazone	Cutaneous reaction, multiorgan toxicity
O[C@]1(n2c3[C@H]4N(CCC[C@]4(C1)CC)CCc3c1c2cccc1)C(= O)OC	0.8101	0.979	Vincamine	Hematologic toxicity
Clc1c(O)c(Sc2c(O)c(Cl)cc(Cl)c2)cc(Cl)c1	0.8154	0.985	Bithionol	Dermatologic toxicity
OC(= O)Cc1ccc(CC(C)C)cc1	0.8507	0.931	Ibufenac	Hepatotoxicity, jaundice
Oc1c(C(= O)c2cc(O)c(O)c(O)c2)ccc(O)c1O	0.8572	0.858	EXIFONE	Hepatotoxicity

### Analysis and discussion of toxim prediction results on second validation set

Food additives such as aspartame, saccharin and MSG, were predicted to be toxic by all three modules of ToxiM (Maher and Wurtman, 1987; Ellwein and Cohen, 1990; Freeman, 2006). Aspartame and saccharin are artificial non-carbohydrate calorie-free sweeteners, which are commonly available in the commercial market. These were predicted to be toxic (descriptor model score: 0.54 and 0.74 respectively) and were also found to be soluble and permeable. Similarly, MSG, which is used as a flavor enhancer, was predicted to have a toxicity score of 0.57, and was found to be soluble and permeable. Thus, the ToxiM predictions also point toward their potentially harmful effects.

Pesticides are being widely used to control insects, rodents and other pests in the agricultural fields. The continued usage of pesticides has highly detrimental environmental impacts on air, water, soil and food, and could be toxic to humans, lower animals and the food chain. Their exposure has been linked to hormone disruption, cancer, neurological effects like loss of memory, and affect neurological and reproductive development. The ToxiM tool predicted the highly used pesticides DCPA and EDTA (Fountain and Reith, 2014) as toxic with prediction values >0.9, and both were also found to be soluble but, EDTA was predicted to be non-permeable. Another commonly used class of compounds comprised of the beauty and cosmetic products. Molecules such as Butylhydroxybutylnitrosamine (Parkinson and Lotzová, 1989) and Sodium tetradecanesulfonate, which are present in cosmetics, were predicted to be toxic (0.89 and 0.88, respectively) by ToxiM. Imidazolidinyl urea is used in cosmetics as an antimicrobial preservative due to its high solubility in water, and its use has been debatable (https://ntp.niehs.nih.gov/ntp/htdocs/chem_background/exsumpdf/imidazolidinylurea_508.pdf). Similarly, mixed results were also obtained in the case of Imidazolidinyl urea, where the ToxiM descriptor model predicted it to be non-toxic, whereas, both fingerprint and hybrid models predicted it to be toxic. The Imidazolidinyl urea is not known to induce any toxicity in human but in patients with contact dermatitis, it can show positive reaction from the exposure to Imidazolidinyl urea.

Benzethonium chloride (National Toxicology Program, 1995) is commonly used in cosmetics, medicaments, deodorants, and mouthwash because of its antiseptic and antimicrobial properties. It was predicted to be highly toxic by all the three models of ToxiM, and was also predicted to be soluble and permeable. Another commonly used synthetic product, Polysorbate-80 (Roberts et al., 2010), which is used as an emulsifier in vitamins, vaccines, medicines, surfactant in soaps and cosmetics, defoamer in the fermentation of wine, and as a binding agent in ice cream, was also predicted to be highly toxic by all the three models. Mixed results were obtained for Sodium hypochlorite, which is commonly known as bleaching powder, and is used on a large scale for bleaching, surface purification, and disinfection of water. Sodium hypochlorite was predicted to be toxic by fingerprint model and non-toxic by descriptor and hybrid models. The reason for the inconsistency in predictions in the case of sodium hypochlorite can be explained using compositional analysis, which revealed that toxins had atoms such as Cl, Br etc., and sodium hypochlorite also contained Cl atom in its structure. Also, it is predicted to be toxic only by fingerprint model that uses the structural properties of a molecule to make the prediction. On the other hand, the descriptor module predicted bleaching powder to be non-toxic, as it takes into consideration physical and chemical properties of the molecule. It is well-established that sodium hypochlorite acts as an irritant for the human skin but it is not toxic at lower concentrations. The hybrid model uses the feature information from both fingerprint and descriptor modules for making the prediction, which was inconsistent in this case due to the above reasons. Sodium hypochlorite was also predicted to be highly water soluble (Budavari, 1996) and permeable, which is in accordance with experimentally known facts. Asbestos, which is commonly used in construction works because of its thermal insulation and fire protection, was predicted to be toxic (0.54–0.84) (Kanarek, 2011). Ethylene glycol, an antifreeze agent whose toxic effects have been long debated, was predicted to be toxic using the hybrid and fingerprint-based ToxiM models (0.68 and 0.54, respectively) (Jacobsen and McMartin, 1986), whereas it was classified as a non-toxin by the descriptor model. Mixed results obtained for ethylene glycol can be explained by the fact that it does not have any inherent toxicity before it is metabolized (http://emedicine.medscape.com/article/814701-overview#a5).

Methyl methacrylate, which is a polymer used in the manufacture of PMMA and MBS and also in hip and knee replacements, was predicted to be toxic, soluble and permeable. Polyacrylamide-butylamine, a polymer used in the manufacture of pesticides, emulsifiers and pharmaceuticals, was predicted to be toxic by descriptor model but was shown to be non-toxic by fingerprint and hybrid models. Polyacrylamide-butylamine was also predicted to be soluble and permeable with the help of regression models used in ToxiM. A total of 12 out of 15 cases were predicted to be toxic, of which 9 compounds were predicted to be permeable, 11 compounds were predicted to be soluble, and 9 compounds were shown to be water soluble and permeable.

We also attempted to derive the relation between classification and regression predictions scores of molecules present in the second validation dataset. Out of 15 compounds present in the second validation set, three compounds: ethylene glycol, Sodium hypochlorite and Imidazolidinyl urea, were predicted to be non-toxic by descriptor module (Supplementary Table S15). Among 12 compounds that were predicted to be toxic, two terminal cases [Polysorbate 80(glycol) and Asbestos] were found that did not show the linear relation of permeability and solubility with the toxicity prediction done by descriptor module. Some of these cases are discussed in the Supplementary Text S5.

Dimethyl tetrachloroterepthalate (DCPA) which is a pre-emergent-herbicide was shown to be permeable but insoluble in water, which is supported by the report published in the Merck Index where DCPA was suggested to be <5% soluble in water (Budavari, 1989). Human toxicity excerpts have shown that this compound does not show any detectable health effects, which explains the continuous usage of DCPA (Gleason et al., 1957; Hamilton and Hardy, 1974).

Thus, for most of the discussed cases and those presented in Supplementary Tables S10, S15, the toxicity prediction values corroborated well with their known toxic nature, which attests the usability of the tool for the prediction of toxicity of a given compound. However, in a few cases such as Benzethonium Chloride (National Toxicology Program, 1995), the compound was predicted to be toxic, permeable and non-soluble in water but experimentally, it is known to be highly water soluble which is in contrast with the prediction results obtained for Benzethonium Chloride. The plausible explanation for the contrasting result in the case of solubility prediction could be inferred from the results of performance of models for predicting the solubility and permeability, which showed the *R*^2^-values of 0.84 and 0.678 respectively. Polysorbate 80 (glycol), which is known to be highly water soluble was predicted to be neither caco-2 cell permeable nor water soluble. Polysorbate 80 is still used in cosmetics and as food additives, because it does not show any adverse effects at lower concentration (Gosselin et al., 1976; Supplementary Text S5). Solubility and permeability are known to be complex properties, the prediction of which has been a challenging task since the available datasets compile the data generated in different laboratories using different techniques, which reduces the quality of the data and its usability as a good training set (Bergström, 2005). Thus, it is suggested that an independent validation of the predictions made on solubility and permeability properties of the query molecule should be carried out by the user.

## Discussion

The prediction of toxicity of any molecule and/or their metabolites is important while deciding its commercial application in products related to human health and for assessing their potential detrimental effect on the environment. Additionally, the prediction of aqueous solubility and permeability along with its toxicity provides useful information for a given molecule while determining its actual toxic potential. Hence, in this study machine learning-based classification and regression models were developed to predict these properties using the chemical and structural information present in a molecule. The 10-fold cross validation for all steps from algorithm selection to the model optimization helped in avoiding the overfitting of data, which is usually a significant concern while training. The reported accuracies in assigning a molecule as toxic or non-toxic, and coefficient of determination values to predict the aqueous solubility and permeability attest the performance of the tool.

The performances of developed models were measured using the validation sets, and a higher performance was also observed for ToxiM on comparison with admetSAR. Only for a few cases, some discrepancy in predictions by the different models were observed which justifies the inclusion of the different prediction models in ToxiM, and the known literature also supported the variable toxicity predictions observed for such molecules. Thus, it is recommended that the user should examine the query molecule using all the three models available at the web server for comprehensive results.

Several factors need to be considered while determining the toxicity of a given molecule such as its compositional, structural and molecular properties, concentration, aqueous solubility and permeability, target organism/system, which together provides useful information on the toxic potential of a given molecule. In this work, we have focused on the structural and molecular properties of molecules for developing the tool to predict the molecules which could be toxic for humans. Though, the current tool is limited for the prediction of toxicity based on the structural and molecular properties, the achieved accuracy of results on the validation datasets attests the importance of these properties and also justifies the application of machine learning for toxicity prediction. It would be a challenging task to include the effect of concentration, and binding with the downstream targets or any other factor that leads to physiological toxicity. The accuracy and applicability of the tool may further be improved in the future by including the other properties, but it would require a lot of experimental data to generate such models. The predictions made using this server will provide valuable information to the scientific community to examine the environmental and physiological toxicity of a given molecule, and especially working in the field of xenobiotics metabolism and toxicity.

## Author contributions

VS and AS conceived the work, participated in the design of the study. AS, GS, and AR developed classification and regression based models. AS and GS were involved in the development of the web server. VS, AS, GS, and AR drafted the manuscript. All the authors read and approved the final manuscript.

### Conflict of interest statement

The authors declare that the research was conducted in the absence of any commercial or financial relationships that could be construed as a potential conflict of interest.

## Supplementary material

Supplementary Figure 1 Compositional analysis using non-toxic molecules.

Supplementary Figure 2 Compositional analysis using toxin molecules.

Supplementary Figure 3 **(A)** PCA plot using fingerprints as input features, **(B)** observation of explained variance from PC-1 to PC-10, **(C)** PCA plot using descriptors as input features, and **(D)** observation of explained variance from PC-1 to PC-10.

Supplementary Figure 4 Variable importance calculation, **(A)** mean decrease in accuracy for top 30 fingerprints, and **(B)** mean decrease in accuracy for top 30 descriptors.

## References

[B1] Artursson P. Karlsson J. (1991). Correlation between oral drug absorption in humans and apparent drug permeability coefficients in human intestinal epithelial (Caco-2) cells. Biochem. Biophys. Res. Commun. 175, 880–885. 10.1016/0006-291X(91)91647-U 1673839

[B2] Bergström C. A. (2005). *In silico* predictions of drug solubility and permeability: two rate-limiting barriers to oral drug Absorption. Basic Clin. Pharmacol. Toxicol. 96, 156–161. 10.1111/j.1742-7843.2005.pto960303.x 15733209

[B3] Borenfreund E. Puerner J. A. (1985). Toxicity determined *in vitro* by morphological alterations and neutral red absorption. Toxicol. Lett. 24, 119–124. 10.1016/0378-4274(85)90046-3 3983963

[B4] Budavari S. (ed.). (1996). The Merck Index-An Encyclopedia of Chemicals, Drugs, and Biologicals. Whitehouse Station, NJ: Merck and Co., Inc., 1478.

[B5] Budavari S. (ed.). (1989). The Merck Index-Encyclopedia of Chemicals, Drugs and Biologicals. Rahway, NJ: Merck and Co., Inc., 446.

[B6] Cao Y. Charisi A. Cheng L. C. Jiang T. Girke T. (2008). ChemmineR: a compound mining framework for R. Bioinformatics 24, 1733–1734. 10.1093/bioinformatics/btn307 18596077 PMC2638865

[B7] Cheng F. Li W. Zhou Y. Shen J. Wu Z. Liu G.. (2012). admetSAR: a comprehensive source and free tool for assessment of chemical ADMET properties. J. Chem. Inf. Model. 2012, 52, 3099–3105. 10.1021/ci300367a 23092397

[B8] Dong J. Cao D. S. Miao H. Y. Liu S. Deng B. C. Yun Y. H.. (2015). ChemDes: an integrated web-based platform for molecular descriptor and fingerprint computation. J. Cheminform. 7:60. 10.1186/s13321-015-0109-z 26664458 PMC4674923

[B9] Ellwein L. B. Cohen S. M. (1990). The health risks of saccharin revisited. Crit. Rev. Toxicol. 20, 311–326. 10.3109/10408449009089867 2202324

[B10] Fountain J. S. Reith D. M. (2014). Dangers of “EDTA.” N. Z. Med. J. 127, 126–127. 25146869

[B11] Freeman M. (2006). Reconsidering the effects of monosodium glutamate: a literature review. J. Am. Assoc. Nurse Pract. 18, 482–486. 10.1111/j.1745-7599.2006.00160.x 16999713

[B12] Geladi P. Kowalski B. R. (1986). Partial least-squares regression: a tutorial. Anal. Chim. Acta 185, 1–17. 10.1016/0003-2670(86)80028-9

[B13] Gernhardt C. Eppendorf K. Kozlowski A. Brandt M. (2004). Toxicity of concentrated sodium hypochlorite used as an endodontic irrigant. Int. Endod. J. 37, 272–280. 10.1111/j.0143-2885.2004.00804.x 15056354

[B14] Gleason M. N. Gosselin R. E. Hodge H. C. (1957). Clinical Toxicology of Commercial Products. Baltimore, MD: Williams and Wilkins Co., 154.

[B15] Gosselin R. E. Hodge H. C. Smith R. P. Gleason M. N. (1976). Clinical Toxicology of Commercial Products. 4th Edn. Baltimore: Williams and Wilkins, II–181.

[B16] Hamilton A. Hardy H. L. (1974). Industrial Toxicology. Acton, MA: Publishing Sciences Group. Inc.

[B17] Hanley J. A. McNeil B. J. (1982). The meaning and use of the area under a receiver operating characteristic (ROC) curve. Radiology 143, 29–36. 10.1148/radiology.143.1.7063747 7063747

[B18] Harry G. J. Billingsley M. Bruinink A. Campbell I. L. Classen W. Dorman D. C.. (1998). *In vitro* techniques for the assessment of neurotoxicity. Environ. Health Perspect. 106:131. 10.1289/ehp.98106s1131 9539010 PMC1533280

[B19] Hinderliter P. M. Minard K. R. Orr G. Chrisler W. B. Thrall B. D. Pounds J. G.. (2010). ISDD: a computational model of particle sedimentation, diffusion and target cell dosimetry for *in vitro* toxicity studies. Part. Fibre Toxicol. 7:36. 10.1186/1743-8977-7-36 21118529 PMC3012653

[B20] Hutchinson T. C. Hellebust J. A. Mackay D. Tarn D. Kauss P. (1979). Relationship of hydrocarbon solubility to toxicity in algae and cellular membrane effects, in International Oil Spill Conference: American Petroleum Institute (Washington, DC), 541–547.

[B21] Ihaka R. Gentleman R. (1996). R: a language for data analysis and graphics. J. Comput. Graph. Stat. 5, 299–314.

[B22] Jacobsen D. McMartin K. E. (1986). Methanol and ethylene glycol poisonings. Med. Toxicol. 1, 309–334. 10.1007/BF03259846 3537623

[B23] Kanarek M. S. (2011). Mesothelioma from chrysotile asbestos: update. Ann. Epidemiol. 21, 688–697. 10.1016/j.annepidem.2011.05.010 21820631

[B24] King Z. A. Lu J. Dräger A. Miller P. Federowicz S. Lerman J. A.. (2016). BiGG Models: a platform for integrating, standardizing and sharing genome-scale models. Nucleic Acids Res. 44, D515–D522. 10.1093/nar/gkv1049 26476456 PMC4702785

[B25] Klaassen C. D. Amdur M. O. (1996). Casarett and Doull's Toxicology: The Basic Science of Poisons. New York, NY: McGraw-Hill.

[B26] Kotsiantis S. B. Zaharakis I. Pintelas P. (2007). Supervised machine learning: a review of classification techniques. Informatica 31, 249–268.

[B27] Kujawski J. Popielarska H. Myka A. Drabinska B. Bernard M. K. (2012). The log P parameter as a molecular descriptor in the computer-aided drug design–an overview. Comput. Methods Sci. Technol. 18, 81–88. 10.12921/cmst.2012.18.02.81-88

[B28] Liaw A. Wiener M. (2002). Classification and regression by randomForest. R news 2, 18–22.

[B29] Lim E. Pon A. Djoumbou Y. Knox C. Shrivastava S. Guo A. C.. (2010). T3DB: a comprehensively annotated database of common toxins and their targets. Nucleic Acids Res. 38, D781–D786. 10.1093/nar/gkp934 19897546 PMC2808899

[B30] Maher T. J. Wurtman R. J. (1987). Possible neurologic effects of aspartame, a widely used food additive. Environ. Health Perspect. 75:53. 10.1289/ehp.877553 3319565 PMC1474447

[B31] Mayr A. Klambauer G. Unterthiner T. Hochreiter S. (2016). DeepTox: toxicity prediction using deep learning. Front. Environ. Sci. 3:80 10.3389/fenvs.2015.00080

[B32] Mishra N. K. Singla D. Agarwal S. Raghava G. P. (2014). ToxiPred: a server for prediction of aqueous toxicity of small chemical molecules in T. Pyriformis. J. Trans. Toxicol. 1, 21–27. 10.1166/jtt.2014.1005

[B33] National Toxicology Program, (1995). NTP Toxicology and Carcinogenesis Studies of Benzethonium Chloride (CAS No. 121-54-0) in F344/N Rats and B6C3F1 Mice (Dermal Studies). Natl. Toxicol. Program Tech. Rep. Ser. 438:1. 12595925

[B34] Nowicki S. Gottlieb E. (2015). Oncometabolites: tailoring our genes. Febs J. 282, 2796–2805. 10.1111/febs.13295 25864878 PMC4676302

[B35] Palmer D. S. O'boyle N. M. Glen R. C. Mitchell J. B. (2007). Random forest models to predict aqueous solubility. J. Chem. Inf. Model. 47, 150–158. 10.1021/ci060164k 17238260

[B36] Parkinson D. Lotzová E. (1989). Interleukin-2, killer cells and cancer therapy: an overview. Nat. Immun. Cell Growth Regul. 9, 237–241. 2215512

[B37] Roberts C. L. Keita Å. V. Duncan S. H. O'kennedy N. Söderholm J. D. Rhodes J. M. (2010). Translocation of Crohn's disease *Escherichia coli* across M-cells: contrasting effects of soluble plant fibres and emulsifiers. Gut 2009:195370 10.1136/gut.2009.195370 PMC2976079 20813719

[B38] Schneider A. Hommel G. Blettner M. (2010). Linear regression analysis. Dtsch Ä Rztebl Int 107, 776–782. 10.3238/arztebl.2010.0776 21116397 PMC2992018

[B39] Seaton A. Godden D. MacNee W. Donaldson K. (1995). Particulate air pollution and acute health effects. Lancet 345, 176–178. 10.1016/S0140-6736(95)90173-6 7741860

[B40] Shonkoff J. P. Garner A. S. Siegel B. S. Dobbins M. I. Earls M. F. Mcguinn L.. (2012). The lifelong effects of early childhood adversity and toxic stress. Pediatrics 129, e232–e246. 10.1542/peds.2011-2663 22201156

[B41] Van Breemen R. B. Li Y. (2005). Caco-2 cell permeability assays to measure drug absorption. Exp. Opin. Drug Metab. Toxicol. 1, 175–185. 10.1517/17425255.1.2.175 16922635

[B42] Van Huffel S. (1997). Recent advances in total least squares techniques and errors-in-variables modeling, in Proceeding of 2nd International Workshop on Total Least Squares and Errors-in-Variables Modeling, ed Siam (Leuven), 21–24.

[B43] Wang N. N. Dong J. Deng Y. H. Zhu M. F. Wen M. Yao Z. J.. (2016). ADME properties evaluation in drug discovery: prediction of Caco-2 Cell permeability using a combination of NSGA-II and boosting. J. Chem. Inf. Model. 56, 763–773. 10.1021/acs.jcim.5b00642 27018227

[B44] Wishart D. S. Knox C. Guo A. C. Shrivastava S. Hassanali M. Stothard P.. (2006). DrugBank: a comprehensive resource for in silico drug discovery and exploration. Nucleic Acids Res. 34, D668–D672. 10.1093/nar/gkj067 16381955 PMC1347430

[B45] Wold S. Esbensen K. Geladi P. (1987). Principal component analysis. Chemometr. Intell. Lab. Syst. 2, 37–52. 10.1016/0169-7439(87)80084-9

[B46] Xue L. Bajorath J. (2000). Molecular descriptors in chemoinformatics, computational combinatorial chemistry, and virtual screening. Comb. Chem. High Throughput Screen. 3, 363–372. 10.2174/1386207003331454 11032954

[B47] Yap C. W. (2011). PaDEL-descriptor: an open source software to calculate molecular descriptors and fingerprints. J. Comput. Chem. 32, 1466–1474. 10.1002/jcc.21707 21425294

