# Supplementary figures and images for: ToxiM: A Toxicity Prediction Tool for Small Molecules Developed Using Machine Learning and Chemoinformatics Approaches
Source: Front Pharmacol. 2017 Nov 30;8:880. doi: 10.3389/fphar.2017.00880 (PMC5714866; doi:10.3389/fphar.2017.00880)

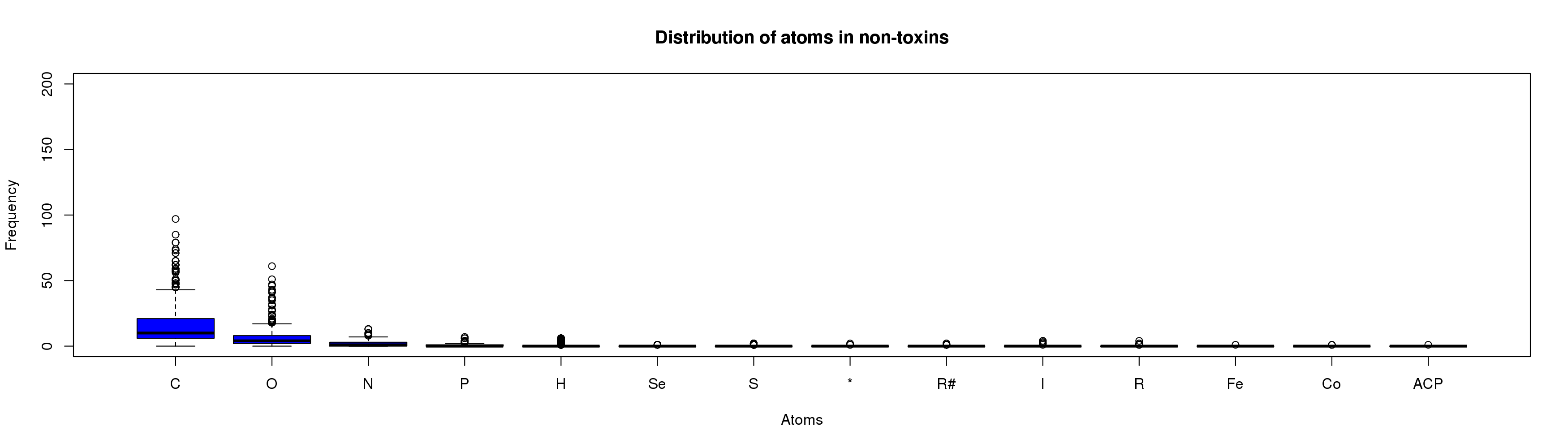

Supplement: Supplementary Figure 1 — Compositional analysis using non-toxic molecules. [file Image1.PNG]

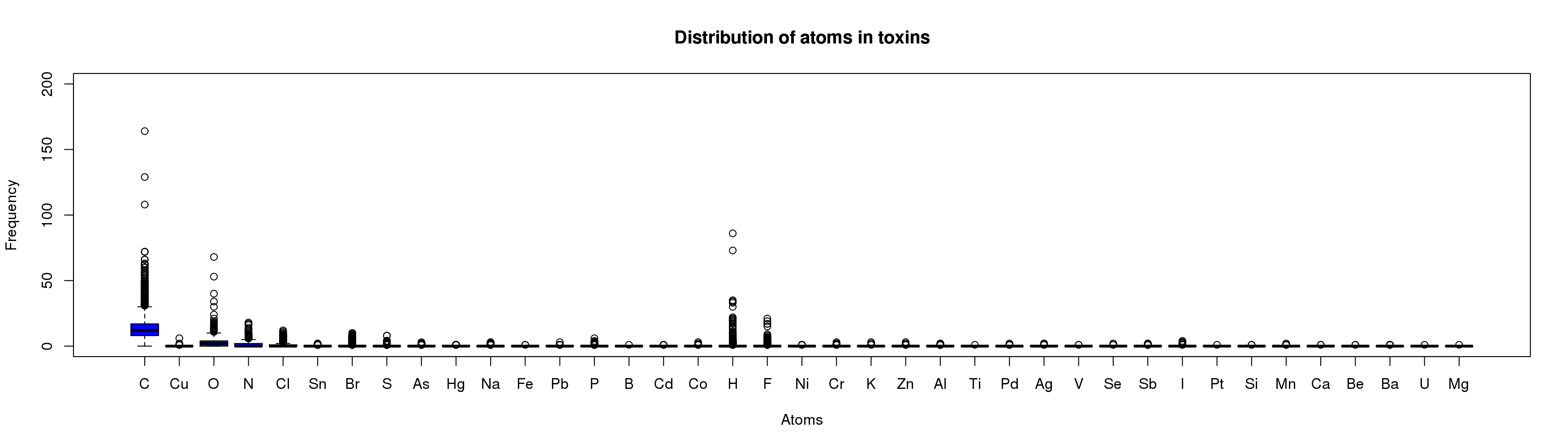

Supplement: Supplementary Figure 2 — Compositional analysis using toxin molecules. [file Image2.PNG]

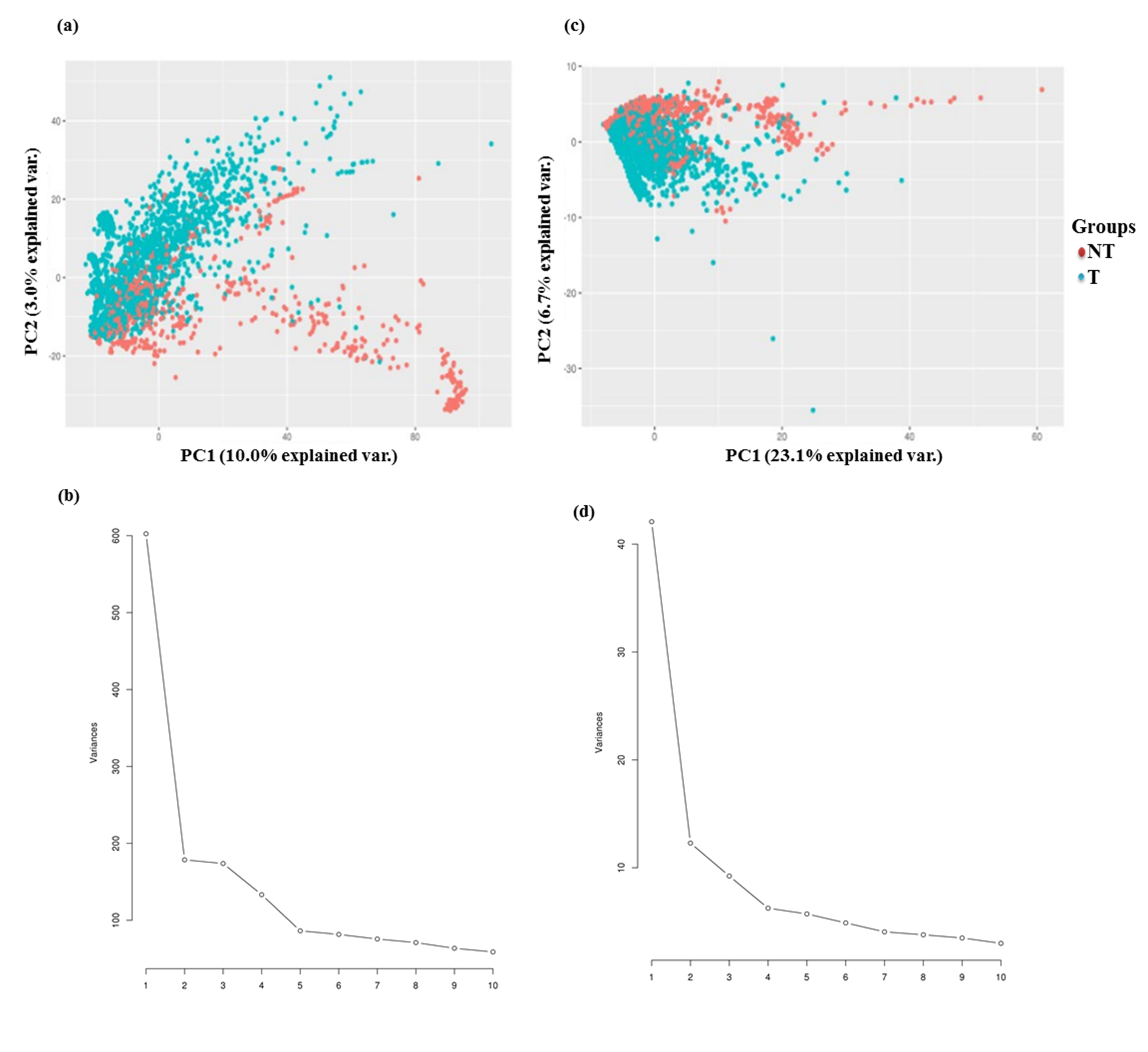

Supplement: Supplementary Figure 3 — (A) PCA plot using fingerprints as input features, (B) observation of explained variance from PC-1 to PC-10, (C) PCA plot using descriptors as input features, and (D) observation of explained variance from PC-1 to PC-10. [file Image3.TIF]

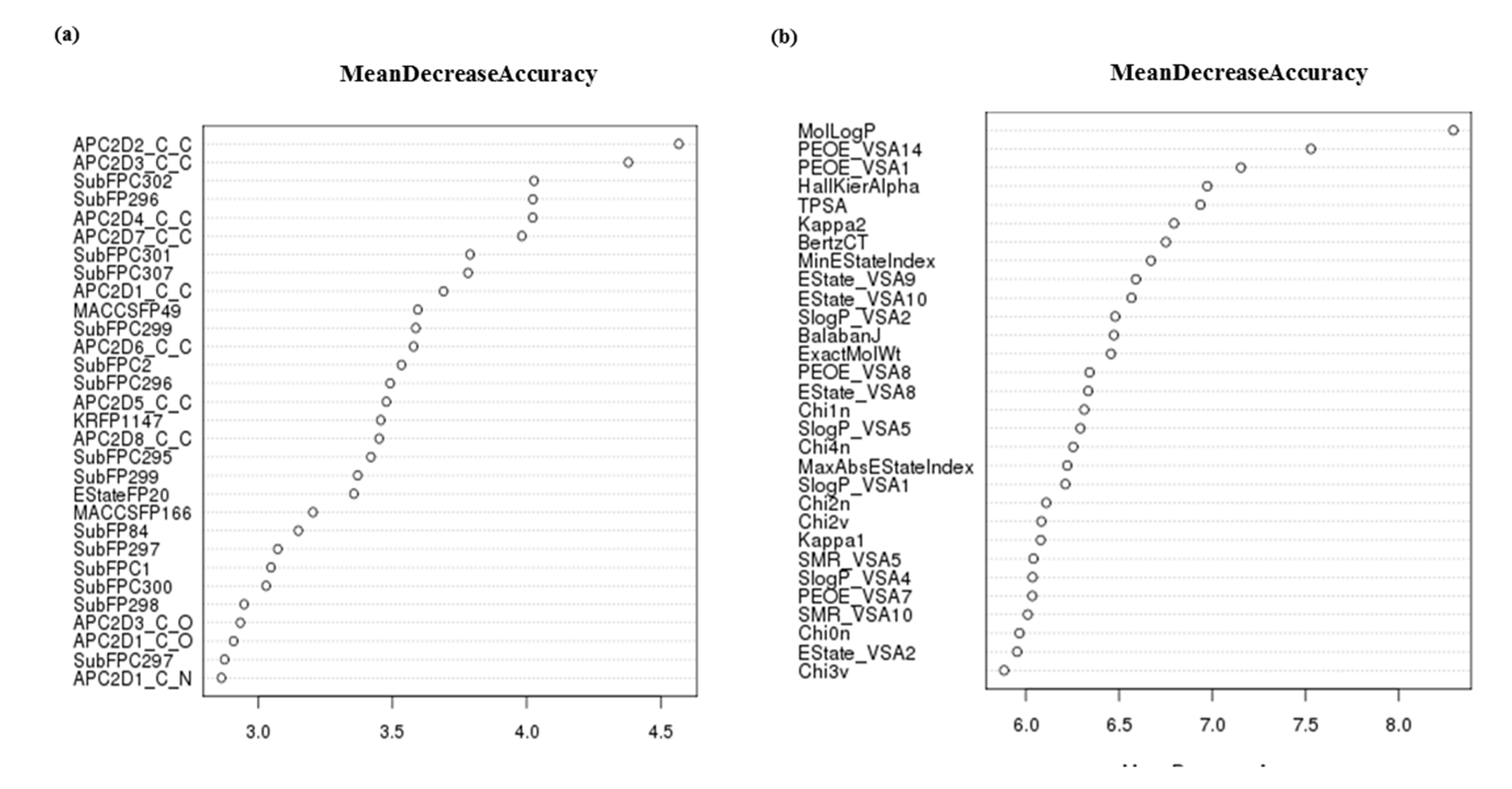

Supplement: Supplementary Figure 4 — Variable importance calculation, (A) mean decrease in accuracy for top 30 fingerprints, and (B) mean decrease in accuracy for top 30 descriptors. [file Image4.TIF]
