# Supplementary material for: ToxiM: A Toxicity Prediction Tool for Small Molecules Developed Using Machine Learning and Chemoinformatics Approaches
Source: Front Pharmacol. 2017 Nov 30;8:880. doi: 10.3389/fphar.2017.00880 (PMC5714866; doi:10.3389/fphar.2017.00880)
Supplement: Supplementary file 5 [file Table1.DOCX]

**Supplementary  Table S1**. List of the fingerprints to select most important ones for the construction of RF based models

Fingerprint MeanDecreaseAccuracy

APC2D2_C_C 4.56727188438074

APC2D3_C_C 4.37926622760802

SubFPC302 4.02800210393458

SubFP296 4.0234287857152

APC2D4_C_C 4.02298544790154

APC2D7_C_C 3.98250370425183

SubFPC301 3.78936407002127

SubFPC307 3.7818493481104

APC2D1_C_C 3.69069846879526

MACCSFP49 3.59464885212824

SubFPC299 3.58699932396006

APC2D6_C_C 3.57884389786126

SubFPC2 3.53382572462454

SubFPC296 3.49165691394652

APC2D5_C_C 3.47733045632363

KRFP1147 3.45643232517024

APC2D8_C_C 3.45074164528135

SubFPC295 3.41983617768423

SubFP299 3.37062537511903

EStateFP20 3.35630133058993

MACCSFP166 3.20397374522798

SubFP84 3.14951948244192

SubFP297 3.07266601303226

SubFPC1 3.04762174151106

SubFPC300 3.02921662275696

SubFP298 2.94736004949891

APC2D3_C_O 2.93332695780113

APC2D1_C_O 2.90809191817476

SubFPC297 2.87448478732648

APC2D1_C_N 2.86279769350198

SubFPC84 2.80833684969117

APC2D5_C_O 2.78844464966165

SubFPC298 2.78213003059813

APC2D4_C_O 2.76712743016992

APC2D2_C_O 2.75749296980718

MACCSFP82 2.67975465885329

APC2D9_C_C 2.66064719713492

APC2D10_C_C 2.65116312317588

APC2D2_O_O 2.63308584519006

APC2D6_C_O 2.56380376872612

MACCSFP149 2.56079229451572

SubFPC14 2.55369803380789

APC2D9_C_O 2.53043493728272

SubFPC12 2.51531700757204

GraphFP306 2.49362327157221

MACCSFP104 2.48074087077755

SubFPC5 2.45354926532097

FP921 2.44683329342531

AD2D547 2.44284610647031

KRFP669 2.42325925487507

PubchemFP9 2.41172770971227

PubchemFP608 2.40456481207067

KRFP2975 2.40263764276673

EStateFP7 2.39655746566749

KRFP466 2.37970090492055

MACCSFP114 2.36187276114819

PubchemFP308 2.35568351028585

FP129 2.33708963000417

PubchemFP346 2.31187187046669

APC2D5_O_O 2.2922109936746

MACCSFP12 2.28748037517036

SubFPC88 2.28336513492789

EStateFP30 2.28234632735658

PubchemFP571 2.27659358492873

APC2D2_C_N 2.24980394598646

KRFP3654 2.24904119391439

SubFP88 2.24806625222456

PubchemFP374 2.24580895761272

FP672 2.24275671303846

PubchemFP617 2.24047136490956

GraphFP156 2.23502407287416

MACCSFP122 2.22862301039729

APC2D7_C_O 2.22286025496135

SubFPC275 2.22025570271997

KRFP2547 2.21121348457229

KRFP3744 2.1904115563041

KRFP3737 2.17687776560839

APC2D1_C_X 2.17411128505989

SubFPC3 2.17343967507319

GraphFP786 2.17151715509096

KRFP4080 2.16977257931683

EStateFP36 2.16760173957882

SubFP14 2.16746835718088

APC2D8_C_O 2.16668549762679

MACCSFP160 2.14623015597127

PubchemFP420 2.14615767443546

FP392 2.14491689229556

APC2D2_N_O 2.14095339759378

KRFP3788 2.13986615066615

KRFP3959 2.13750412579515

AD2D469 2.13739563210853

PubchemFP452 2.13641236327612

PubchemFP14 2.12654330228479

MACCSFP159 2.12337818318629

PubchemFP366 2.11839397004628

PubchemFP440 2.11834289263296

PubchemFP528 2.11089883770542

MACCSFP156 2.10739151723794

KRFP2949 2.09814571888743

FP802 2.07570279511245

PubchemFP710 2.07555957649095

FP394 2.07255731317414

MACCSFP134 2.07081755779712

PubchemFP432 2.06921677070305

MACCSFP94 2.06382029767385

PubchemFP283 2.0635212232539

PubchemFP570 2.06218359647626

KRFP1406 2.06211637931594

PubchemFP470 2.0613867659192

AD2D90 2.05959485935637

MACCSFP153 2.05469761627802

SubFP41 2.05272332053107

MACCSFP154 2.04808865006807

PubchemFP578 2.04686919445208

KRFP3455 2.04663862782453

PubchemFP776 2.04544992981872

SubFP12 2.04477885760669

FP34 2.04233322882543

KRFP3414 2.01968087457742

PubchemFP2 2.019125520779

MACCSFP123 2.01628954178335

PubchemFP594 2.01478772930409

MACCSFP72 2.01442920207202

FP118 2.01400434314073

KRFP3790 2.01332738689861

AD2D391 2.00979614789408

PubchemFP535 2.00678314163152

FP638 2.00665282266997

GraphFP263 2.00560474868372

PubchemFP595 2.00444040673462

KRFP1148 1.9988963628466

GraphFP726 1.99416500198737

PubchemFP696 1.99409126317609

AD2D703 1.99398507894467

PubchemFP637 1.99320577623972

FP96 1.99226008922496

APC2D4_C_N 1.98775215268171

PubchemFP634 1.98763965468752

APC2D5_C_N 1.98532883680065

GraphFP453 1.97968380321506

PubchemFP592 1.97913333664054

MACCSFP53 1.97616858317945

PubchemFP537 1.97550831383332

AD2D12 1.97543109553494

FP85 1.96716687753725

SubFPC49 1.96542169800027

FP76 1.96259663144911

SubFPC4 1.96064084277777

MACCSFP54 1.96025182064827

PubchemFP677 1.95986409036262

GraphFP332 1.95960240602017

APC2D2_C_X 1.9568639934734

APC2D3_O_O 1.94808910721487

MACCSFP138 1.94697628884702

FP645 1.94445283085942

MACCSFP107 1.94234284574148

PubchemFP178 1.94064722578067

MACCSFP22 1.93999171125544

PubchemFP393 1.93878362574288

KRFP3268 1.93256637557766

KRFP1 1.93128992631632

PubchemFP406 1.92932281290186

FP549 1.92910952371973

KRFP189 1.91121812027875

PubchemFP684 1.9107813355416

KRFP17 1.91029214289189

GraphFP730 1.90703548467524

EStateFP35 1.90641089051871

FP746 1.9053690510052

MACCSFP16 1.90493908172467

SubFP49 1.90444101740692

PubchemFP664 1.90364203451059

PubchemFP603 1.9006400431585

PubchemFP365 1.89833168877895

KRFP341 1.89725107871014

FP128 1.89654708049146

APC2D1_O_P 1.89341258629155

KRFP4237 1.89235909368585

FP57 1.89096738030461

FP146 1.88883018384396

GraphFP878 1.88791231950664

PubchemFP301 1.88488495086862

GraphFP770 1.88242448762838

FP688 1.8811362907742

GraphFP815 1.87617617192046

GraphFP245 1.87585633932421

AD2D1 1.87465897462708

FP345 1.86817550704157

KRFP3971 1.86646020491041

FP455 1.86363119929638

KRFP2262 1.85489661663086

SubFP2 1.8538185450856

AD2D313 1.853437589909

PubchemFP411 1.85253462933193

MACCSFP63 1.85220798163102

GraphFP499 1.84993324365283

MACCSFP155 1.84890968301923

FP181 1.84749003888542

PubchemFP668 1.84474758565799

GraphFP926 1.8437300901729

SubFP302 1.84212674218007

PubchemFP425 1.84182966369922

FP721 1.83795585602329

AD2D170 1.83716499337094

FP932 1.8332512917896

APC2D3_C_N 1.83298753220965

PubchemFP443 1.82837834708277

AD2D26 1.82433204129014

APC2D4_O_O 1.82344028907828

AD2D246 1.82335712938346

GraphFP157 1.81854117425266

SubFP1 1.81751274983856

MACCSFP108 1.81557006525479

PubchemFP185 1.81550448972649

SubFPC287 1.81486605210942

EStateFP16 1.81054673467877

PubchemFP341 1.8079557023303

FP743 1.80789214026838

MACCSFP120 1.80724759414247

MACCSFP137 1.80650667695138

MACCSFP102 1.80522689695891

MACCSFP100 1.80310941370228

FP77 1.80167824134389

PubchemFP520 1.80142268648937

MACCSFP151 1.79769345330035

GraphFP542 1.79719641647134

KRFP3738 1.79712704255993

AD2D258 1.79701904352544

APC2D9_O_O 1.79680165774146

MACCSFP86 1.79339232525845

PubchemFP371 1.79295921540611

FP301 1.78983925813806

KRFP3328 1.78784793039327

FP920 1.78489692555819

KRFP4695 1.78413155806899

FP832 1.78311463349085

AD2D236 1.78254526788535

GraphFP929 1.78116666167536

FP668 1.78114876608498

GraphFP544 1.78017842243426

GraphFP506 1.77990865664191

FP644 1.77687739183018

FP302 1.77599633487278

GraphFP859 1.77477069928138

APC2D3_C_X 1.77288469622083

SubFPC36 1.77223335864721

PubchemFP536 1.77188220441405

GraphFP892 1.76876441940194

MACCSFP71 1.76597663431994

GraphFP362 1.76590185415691

KRFP3750 1.7651160260409

PubchemFP680 1.76456586316071

FP75 1.76373108391486

KRFP2673 1.7632887710283

PubchemFP618 1.76326836080569

MACCSFP148 1.76056248417497

FP87 1.75982982140471

PubchemFP340 1.75866814898529

PubchemFP284 1.75849510623837

FP499 1.75845837371749

MACCSFP126 1.75811726912698

MACCSFP84 1.75645606777674

GraphFP155 1.75581927134

PubchemFP384 1.75352968086099

PubchemFP345 1.75299601528553

MACCSFP146 1.7519000931973

FP70 1.74917869642987

PubchemFP818 1.74748346842874

SubFPC282 1.7449389281397

GraphFP323 1.74464970596092

EStateFP34 1.74161334179608

FP477 1.74086053524118

PubchemFP380 1.73915337367025

FP744 1.73832403915237

SubFPC41 1.7371786147955

GraphFP504 1.73535044250125

FP180 1.73187623276227

KRFP298 1.7310894621862

FP546 1.73038474013964

KRFP3741 1.72933426993014

KRFP4811 1.7285262704204

AD2D180 1.72830982952418

EStateFP21 1.72817654209095

PubchemFP192 1.72794945554251

FP800 1.72430814661929

APC2D1_N_O 1.72251017469811

FP482 1.72104749027196

KRFP4117 1.71524968955483

GraphFP790 1.71451013463672

PubchemFP11 1.71408965460348

MACCSFP141 1.71190545439235

KRFP3394 1.70807954920786

FP661 1.70596835100154

PubchemFP462 1.70503124848005

APC2D5_C_P 1.7031815816314

FP354 1.70031340582771

PubchemFP143 1.70027906560738

SubFPC274 1.69880725819093

AD2D315 1.69802532597926

AD2D338 1.69739089541944

GraphFP563 1.69517205133821

FP252 1.69464183376285

PubchemFP405 1.69460277471529

PubchemFP456 1.69333736620158

APC2D4_C_X 1.68790308268758

GraphFP205 1.68725164166614

FP397 1.68697261104983

GraphFP531 1.68667129451264

PubchemFP391 1.68529133195652

MACCSFP164 1.68220503976726

FP534 1.68201540598039

GraphFP197 1.68146174869459

FP73 1.68087294024878

GraphFP316 1.68008435637868

FP193 1.68007080122991

GraphFP1010 1.68003823072514

PubchemFP339 1.67982705954789

GraphFP743 1.67931614326362

PubchemFP666 1.67928425777204

APC2D5_C_X 1.67915658878909

FP603 1.67904086831778

MACCSFP139 1.67849947952701

SubFP48 1.67843567337228

SubFP16 1.67837748709351

PubchemFP641 1.67648227470477

GraphFP486 1.67539977957019

APC2D6_O_O 1.673131916971

GraphFP784 1.67225289257071

GraphFP190 1.67200505893198

KRFP2380 1.67189902669425

EStateFP15 1.67092101558233

FP81 1.67028965952464

MACCSFP70 1.66987771857913

SubFPC16 1.66744887006191

MACCSFP83 1.66616037991285

APC2D3_C_P 1.66610906738196

KRFP3360 1.66574366615426

KRFP3697 1.66495521429718

AD2D92 1.6608333620608

APC2D6_C_N 1.66009772285877

KRFP4283 1.65853072411612

GraphFP294 1.6584873553795

GraphFP319 1.65766588654283

FP956 1.65747100298371

KRFP3701 1.65616981359382

GraphFP744 1.65599314052338

MACCSFP124 1.6546022913067

FP544 1.65443497652691

EStateFP31 1.65402488999753

GraphFP1005 1.65232214715255

AD2D2 1.65045218230633

FP471 1.64712015812342

GraphFP334 1.64615398427412

FP225 1.64597142567611

MACCSFP90 1.64368241193427

GraphFP307 1.64321624467256

GraphFP689 1.64120517755103

AD2D414 1.63990303619805

KRFP3671 1.63827534839548

FP766 1.63760480060108

MACCSFP69 1.63627273220278

APC2D9_N_O 1.63571787467386

AD2D260 1.63558335711327

GraphFP889 1.63547155087019

AD2D79 1.63468496652031

FP495 1.6343514104185

GraphFP104 1.63413582534829

FP771 1.63160610405667

MACCSFP93 1.63098402152469

PubchemFP13 1.62972674893565

PubchemFP390 1.62971711973309

PubchemFP286 1.62938205172883

FP818 1.62861005070003

KRFP3730 1.62847471586677

KRFP548 1.62821577417304

FP633 1.62815110990541

FP1016 1.62733042146528

PubchemFP15 1.62707173884135

GraphFP73 1.62694781401253

GraphFP60 1.62688853298251

FP451 1.62606701262075

FP239 1.6253267171674

GraphFP1003 1.62353746045471

KRFP3706 1.62344934931562

MACCSFP29 1.62295623190508

FP307 1.61960831379646

FP447 1.61850692758865

FP671 1.61825296357974

FP691 1.6173974235924

GraphFP96 1.61623405952754

SubFPC35 1.61507978887186

SubFP275 1.61450448040883

MACCSFP158 1.61438029418027

KRFP3593 1.61341587681263

FP873 1.61239276604434

FP17 1.61162912047717

MACCSFP150 1.61079210962457

SubFP300 1.61060181749822

FP166 1.60925750202413

KRFP3692 1.60905038403304

KRFP647 1.60879535644633

KRFP3668 1.60844204268595

FP212 1.60719222084575

SubFPC47 1.60691856318902

GraphFP122 1.60609885670264

EStateFP17 1.60542208962021

PubchemFP643 1.60296642081833

APC2D7_C_N 1.60118334030475

GraphFP183 1.60092010184033

APC2D7_O_O 1.60059019905095

GraphFP59 1.59890237339482

PubchemFP699 1.59835339750474

FP874 1.5976167080285

MACCSFP121 1.5968082710944

FP708 1.5966048888651

KRFP3369 1.59649761095954

SubFP3 1.59643534274397

FP494 1.59618820671729

GraphFP428 1.59610545178277

SubFPC283 1.59454731070258

MACCSFP142 1.59329816431131

PubchemFP436 1.59147000042812

PubchemFP646 1.59024745517506

FP1007 1.58931840809176

FP944 1.5887256487043

AD2D14 1.58659989225012

FP994 1.58621473382266

SubFP35 1.58602361531863

GraphFP543 1.58540912876782

GraphFP308 1.58535190682508

KRFP300 1.58345249125757

FP374 1.58255204877976

FP326 1.58115588513719

FP830 1.58040879815234

FP283 1.57892574095062

MACCSFP89 1.5785941324888

FP953 1.57809951235785

KRFP3025 1.57522091004119

FP819 1.57500926229622

FP498 1.5728555309267

GraphFP965 1.57197298019248

GraphFP228 1.57195143681648

MACCSFP157 1.57152087209252

KRFP382 1.57110563419743

FP335 1.57093052075837

PubchemFP552 1.57071920497446

KRFP3751 1.56930219707869

MACCSFP128 1.56625646151192

MACCSFP105 1.56501430647122

PubchemFP688 1.56381063212267

FP772 1.56297675026497

FP405 1.56220726339792

FP409 1.56040705747358

GraphFP311 1.55942139892224

AD2D169 1.55916922955343

FP572 1.55858477087276

KRFP3058 1.55760228701781

GraphFP303 1.557139725751

FP670 1.55623822340848

FP798 1.55583317281275

PubchemFP697 1.55568339253329

PubchemFP314 1.55520248805524

MACCSFP132 1.55380285600496

PubchemFP777 1.55303175711432

PubchemFP1 1.55254634957455

FP37 1.55227523536943

FP423 1.55088170761305

AD2D159 1.54939152069048

FP59 1.54800326496553

FP107 1.54522600889517

MACCSFP119 1.54491105922995

GraphFP723 1.54341070303685

SubFPC48 1.5432429598427

FP503 1.54255664012507

AD2D158 1.54191755584332

PubchemFP182 1.54147386098155

FP481 1.54124235818708

AD2D393 1.5412004216037

KRFP3884 1.54095401600058

KRFP401 1.53815707724553

GraphFP243 1.53763857943335

FP390 1.53704982315659

GraphFP725 1.53670593043343

FP596 1.53668945354677

FP171 1.53632229430424

GraphFP63 1.53630875256372

GraphFP475 1.53485393813505

FP692 1.53468916195118

PubchemFP370 1.53419534127918

PubchemFP455 1.53404730821498

KRFP3746 1.53401010138039

FP305 1.53399837639291

FP457 1.53289115235343

GraphFP917 1.53197182045394

PubchemFP451 1.53065389413904

KRFP409 1.53018929724508

AD2D336 1.53013996286582

PubchemFP498 1.5284152185598

SubFPC100 1.5277070366771

FP253 1.52764717652235

AD2D189 1.52468895855083

FP913 1.52447418515484

PubchemFP540 1.52400587373778

GraphFP884 1.52390425027699

FP150 1.52350205067591

PubchemFP352 1.52292687789538

KRFP3752 1.52267199381635

AD2D161 1.52228794012835

FP622 1.52167291281098

FP1001 1.52068155571387

FP461 1.52026400151556

APC2D7_C_X 1.5201998613554

FP114 1.5196837444876

FP207 1.51946014498374

PubchemFP491 1.51929186998354

GraphFP637 1.51709869540705

FP32 1.51706490412154

KRFP2259 1.51444962967571

GraphFP186 1.51443448782726

PubchemFP708 1.51336089975089

AD2D168 1.51334955418963

MACCSFP152 1.51261187167817

GraphFP684 1.51259824811613

GraphFP676 1.51057105332227

KRFP3399 1.51012180816197

FP44 1.50911673183176

MACCSFP109 1.50684263089173

KRFP346 1.50534087110226

KRFP3428 1.50322695200327

PubchemFP12 1.50206628197111

GraphFP728 1.50090655014593

FP995 1.50071906225822

FP444 1.50066470454228

GraphFP390 1.49968755004634

AD2D402 1.49844543213382

KRFP14 1.49840594932743

KRFP3806 1.49717053306766

FP311 1.49664076698302

EStateFP33 1.49501655954499

AD2D102 1.49277955938721

FP537 1.48856208274873

PubchemFP430 1.48736815117996

GraphFP955 1.48595751816664

MACCSFP96 1.48528727614475

GraphFP898 1.48479050459496

PubchemFP698 1.48385491240036

APC2D10_C_O 1.48309950986916

EStateFP24 1.48255581399147

APC2D6_N_N 1.48239239974875

MACCSFP24 1.48078692806316

MACCSFP161 1.47935895506152

KRFP3789 1.47808561715249

GraphFP237 1.47807801426842

GraphFP754 1.47659924105283

GraphFP522 1.47628771926485

GraphFP404 1.4759675491495

FP936 1.47517678232729

PubchemFP573 1.47469903934903

APC2D4_O_P 1.47365074190696

AD2D314 1.47271573946913

GraphFP255 1.47086642298611

AD2D3 1.4703835692601

MACCSFP87 1.46993769790786

FP484 1.46950689406464

KRFP397 1.46893580292377

SubFPC137 1.46853817699259

MACCSFP111 1.46789947447157

KRFP3408 1.46731184848764

FP571 1.4669752198362

APC2D3_N_N 1.46548504540453

GraphFP994 1.46303675247261

GraphFP410 1.46286080970538

SubFPC23 1.46285996314051

KRFP4858 1.46213362971191

FP265 1.46049862907123

FP264 1.46030895883182

MACCSFP50 1.46016867091363

AD2D324 1.45987807203477

GraphFP928 1.45932791845507

KRFP493 1.45833546542764

GraphFP1009 1.45794912729228

FP470 1.45736286270881

GraphFP816 1.45713588399493

KRFP3729 1.45660295843971

FP880 1.45641606404868

FP109 1.45639265323729

FP27 1.45619074223401

PubchemFP584 1.45559044767181

FP851 1.4548476460833

FP6 1.45397727475383

GraphFP287 1.45339043577376

KRFP3728 1.45315628785063

GraphFP733 1.45208442663813

GraphFP1008 1.45123541971952

PubchemFP10 1.4497694971143

FP784 1.44961856774631

EStateFP13 1.4493270772157

APC2D10_O_O 1.44919233583191

KRFP4836 1.44889796359805

APC2D4_C_S 1.44857735397194

KRFP3224 1.4464047005241

GraphFP67 1.44568961159617

FP46 1.44512503271284

FP1008 1.44391793371249

KRFP4019 1.44309773186972

AD2D392 1.44271432769278

GraphFP947 1.44219394020704

FP785 1.44101954019841

GraphFP745 1.43920857567224

FP352 1.43846504766119

FP84 1.43815034066247

KRFP3708 1.43776512579792

KRFP3773 1.43628637544356

KRFP4610 1.43544210645325

GraphFP970 1.43480270223445

MACCSFP76 1.43380075572465

GraphFP128 1.43362927854476

FP619 1.43354375720407

FP740 1.43282452856043

FP617 1.4327923670308

FP22 1.43271557784248

KRFP3882 1.43082262355215

KRFP3731 1.43051026263363

AD2D156 1.42996605099747

FP678 1.42991299755986

KRFP505 1.4298781023996

GraphFP382 1.42955976536829

FP11 1.42859042734773

KRFP3956 1.42823759053547

FP123 1.42696227580368

FP52 1.42649864756545

FP839 1.42627501415731

KRFP1149 1.42559823408186

FP327 1.42434436565634

FP231 1.42292903880816

GraphFP518 1.42250812447221

KRFP1146 1.42139053494134

GraphFP787 1.42136446375178

FP195 1.42092795514985

KRFP2265 1.42013705060477

FP167 1.41990554032592

MACCSFP79 1.41919283265134

FP183 1.41863042432531

APC2D8_C_N 1.41824091534605

FP930 1.41786080255395

FP143 1.41698158254958

PubchemFP619 1.41692889537588

GraphFP534 1.41682324573972

FP353 1.41564612527493

EStateFP46 1.41425443443259

PubchemFP674 1.41270723279676

GraphFP943 1.41263428589629

GraphFP919 1.41207797628033

KRFP3336 1.41191801991399

GraphFP487 1.41186434246655

FP1000 1.41155945440755

PubchemFP30 1.41109036045559

AD2D627 1.41098115015213

FP393 1.4102272845851

FP39 1.40985260528691

FP480 1.40858609722041

FP148 1.40826742276834

PubchemFP355 1.40769552065592

FP649 1.40738630714966

PubchemFP423 1.40660315759358

AD2D182 1.40621343718774

FP433 1.40570086450034

AD2D625 1.40458919490088

GraphFP328 1.40375679419748

PubchemFP524 1.40309552938231

GraphFP363 1.4028774119214

APC2D2_C_P 1.40287518041255

SubFP301 1.40273460674781

GraphFP242 1.40230490780066

FP689 1.40169175642414

FP935 1.40148383937701

FP334 1.40119624530256

AD2D492 1.4009319717831

MACCSFP97 1.40036614092165

GraphFP830 1.39983381424449

FP1017 1.39898087747255

KRFP3149 1.39817308062775

SubFP36 1.39801516334829

GraphFP367 1.39758591962438

GraphFP847 1.39721199052895

FP133 1.39702228407032

APC2D6_N_O 1.39700032816196

FP213 1.39620915954692

KRFP4331 1.39334933940609

GraphFP596 1.39293204910722

MACCSFP130 1.39159224319604

MACCSFP112 1.39145693946941

MACCSFP147 1.39047099785914

MACCSFP55 1.39032732717467

PubchemFP564 1.38901908142574

FP198 1.38879236850838

FP767 1.38713659944933

FP125 1.38597770484802

GraphFP747 1.38582190931784

GraphFP760 1.38579485429837

FP656 1.38516888493772

FP543 1.38376333870936

KRFP3782 1.38329442795355

FP954 1.38322905189151

FP889 1.38206455625889

FP759 1.38115671340003

KRFP3640 1.38099824899861

KRFP3660 1.38053615487909

MACCSFP9 1.37996789359586

SubFP100 1.37988063779919

KRFP3821 1.37958708252487

FP1013 1.37914898986551

FP728 1.37907740308532

FP48 1.37815243233564

PubchemFP521 1.37811912367703

FP23 1.37644204428621

MACCSFP143 1.37564581597455

PubchemFP672 1.3756089034491

MACCSFP116 1.37521545038425

FP174 1.37520577423676

FP888 1.37315130601834

FP300 1.37302628003336

AD2D326 1.37256360642148

FP162 1.37146486507237

FP55 1.37136596882937

KRFP3395 1.37100044525456

FP762 1.37035268769202

PubchemFP3 1.3701184626447

MACCSFP66 1.36972275902683

FP1005 1.3695729529731

KRFP3389 1.36937936434383

FP1006 1.36858457061215

PubchemFP645 1.36849294028869

EStateFP12 1.36819305555429

FP647 1.36718731435567

FP765 1.36505337179088

SubFP85 1.3638948190564

GraphFP230 1.36125941047152

MACCSFP110 1.36123045697196

KRFP3295 1.35954366744964

KRFP629 1.35873265053664

GraphFP839 1.35809608349779

GraphFP752 1.35800085278888

FP426 1.35672068546824

GraphFP251 1.35651613148913

FP600 1.35627609995059

KRFP3749 1.35497769811812

FP618 1.35471917966225

FP523 1.35460662955957

MACCSFP92 1.35370044795997

KRFP3693 1.35364997519037

FP702 1.35357021458163

FP514 1.3532626162092

FP486 1.35278660288532

GraphFP842 1.35236480982466

GraphFP348 1.35190978667655

FP196 1.35164690604379

KRFP1193 1.35088109313067

PubchemFP516 1.35086311748363

GraphFP941 1.34990452707754

GraphFP920 1.34977825859053

PubchemFP144 1.34968742422072

PubchemFP442 1.349554979342

FP847 1.3490204277378

GraphFP708 1.3485388637656

FP406 1.34826958649517

GraphFP150 1.34781242130553

PubchemFP660 1.3457357329824

GraphFP530 1.34437741013033

KRFP672 1.34336375530589

FP419 1.34208922639833

FP18 1.34151578862243

FP761 1.34098682030696

FP5 1.34054892570937

MACCSFP136 1.34023987117932

GraphFP922 1.34004042612276

PubchemFP179 1.33951019483872

GraphFP578 1.33886295856986

KRFP4295 1.3385393744607

GraphFP114 1.33784588827386

FP972 1.33774370647643

AD2D85 1.33760988044896

FP156 1.33754954761796

GraphFP887 1.3362712777045

MACCSFP67 1.3362633903787

GraphFP795 1.33617998774965

PubchemFP695 1.33615238680148

MACCSFP19 1.33612866846721

GraphFP320 1.33612814701779

GraphFP856 1.33587354369235

AD2D549 1.33572833924438

GraphFP269 1.33553833984799

GraphFP388 1.33461421838902

GraphFP318 1.33407560020993

APC2D3_O_X 1.33361419222179

FP502 1.33282228524007

PubchemFP299 1.33277508343713

PubchemFP472 1.3326674645904

FP229 1.33250781463533

APC2D4_C_P 1.33198771311998

GraphFP286 1.33148239477133

FP72 1.33131841975156

FP883 1.33103713911623

MACCSFP103 1.33072705296312

GraphFP526 1.33024107781503

FP149 1.33017017654981

FP939 1.329438689355

FP577 1.32926754575282

APC2D3_O_P 1.32821741937773

FP26 1.32803926034094

FP881 1.32793230410566

FP866 1.32750048553134

GraphFP366 1.3272206242183

MACCSFP165 1.32703102738072

PubchemFP860 1.32690965712004

FP992 1.32635561311195

GraphFP264 1.32634359973123

PubchemFP19 1.32607678138291

PubchemFP294 1.32555804722379

GraphFP498 1.32549457718758

PubchemFP38 1.32518968214647

FP136 1.32380492742883

KRFP115 1.32301296467931

KRFP2 1.32257161255046

GraphFP858 1.32175876349212

GraphFP912 1.32164830849091

FP604 1.32123990228753

FP748 1.31919028618841

SubFP283 1.31752285559475

FP958 1.3170862684484

FP176 1.31704994042798

MACCSFP81 1.31629964295686

PubchemFP337 1.31593037260541

FP550 1.31543131889674

PubchemFP681 1.31526252399941

FP805 1.31506866354709

FP989 1.3148363098488

PubchemFP560 1.31431397548796

FP581 1.31422866628869

KRFP3667 1.31402405314805

FP963 1.3139304477985

FP170 1.31386741185864

FP860 1.31374631353258

FP928 1.31365208895983

FP559 1.31331037441595

FP933 1.31323393136276

KRFP111 1.31219282649496

GraphFP298 1.3118521619489

FP806 1.3116939969984

MACCSFP163 1.31129627405629

FP904 1.31126517049811

FP623 1.31046789404428

KRFP839 1.30988550395595

PubchemFP447 1.30898894211109

SubFP287 1.30893043072286

FP652 1.30884925654637

FP179 1.3084490043072

APC2D3_N_O 1.30813750400354

MACCSFP131 1.30795932605958

AD2D237 1.30792906591852

KRFP338 1.30778256501369

FP160 1.30626832522338

FP724 1.30626149158971

GraphFP660 1.30619836980074

FP235 1.30528888904972

FP966 1.30447574102912

PubchemFP712 1.30426578641934

GraphFP606 1.30425600548508

PubchemFP0 1.30418179196117

GraphFP485 1.30415273738074

FP574 1.30379474347871

EStateFP19 1.302572616143

FP791 1.30211462077835

KRFP3293 1.30193400229705

GraphFP573 1.30136981654843

GraphFP115 1.30091582613863

FP236 1.30081997653453

APC2D2_N_N 1.29971141295538

MACCSFP99 1.29967128948602

GraphFP798 1.29963160557898

FP519 1.29887314741643

KRFP362 1.29806888128256

PubchemFP663 1.29722271935442

FP159 1.29651424522633

FP227 1.29610997556731

APC2D8_N_N 1.29602959800938

PubchemFP689 1.29522963946362

FP947 1.29364024627647

APC2D5_O_P 1.29323394830812

PubchemFP582 1.29094300155307

FP764 1.29016121213366

GraphFP797 1.29004112013868

GraphFP140 1.28931126059113

MACCSFP48 1.2890776231583

PubchemFP373 1.28891845723477

FP483 1.28888534351625

FP4 1.28867995951206

PubchemFP464 1.28823120017783

FP593 1.28818500999036

GraphFP170 1.28714163037318

FP821 1.28680986109499

KRFP3926 1.28625809199467

GraphFP285 1.28605987659334

FP867 1.28520111698678

PubchemFP579 1.28488785817691

GraphFP964 1.28463201036717

KRFP504 1.28394482633156

GraphFP911 1.28234583942012

APC2D6_C_X 1.28144503162196

FP42 1.28089583274407

FP726 1.28017879172417

FP240 1.27980423907598

APC2D6_N_P 1.27820604712131

FP210 1.27675774560951

FP788 1.27578312711192

PubchemFP613 1.27574846626788

FP665 1.27563575252383

PubchemFP506 1.27470745101269

PubchemFP692 1.27454949477202

FP131 1.27431287300428

KRFP344 1.27295089813786

FP249 1.27210617938758

EStateFP54 1.27090144296798

FP587 1.27081848970827

FP449 1.26965534582293

FP206 1.2692015562035

FP862 1.26889478333488

KRFP3156 1.26856559925111

KRFP3928 1.26815295844139

FP450 1.26756461571916

MACCSFP91 1.26745984641997

FP959 1.26741061875612

PubchemFP678 1.26710634166228

FP737 1.26626258671372

KRFP343 1.26607164811771

GraphFP500 1.26501840941064

FP391 1.26460688938369

KRFP368 1.26426510357161

PubchemFP553 1.26420471590246

FP610 1.26407203031808

PubchemFP482 1.26398297379836

MACCSFP57 1.26349370976035

PubchemFP344 1.26260015296956

FP703 1.26228648980035

SubFPC224 1.26225732328136

AD2D81 1.26072403517826

KRFP3938 1.25957225363281

PubchemFP797 1.25950423213865

FP126 1.25914102836023

FP7 1.25867046750418

FP441 1.25860935989165

FP513 1.25847046223142

FP630 1.2583163632815

FP934 1.25731608483725

PubchemFP547 1.25726943941754

FP745 1.25726308224371

FP949 1.25702259250727

PubchemFP614 1.25588769341393

GraphFP967 1.25514262531151

PubchemFP639 1.25454534629709

MACCSFP73 1.25281348745123

FP364 1.25220047396954

PubchemFP332 1.25199329796147

PubchemFP416 1.25128053833479

FP53 1.248785741299

GraphFP413 1.24823421973561

PubchemFP679 1.24736740866354

FP741 1.24727943682611

FP245 1.24712094032128

FP153 1.24652374106054

FP850 1.24570521506372

GraphFP175 1.24541085655638

APC2D7_C_P 1.24530667537299

GraphFP667 1.24494882279129

GraphFP756 1.2446732305881

FP681 1.24425924547771

FP795 1.24421609333321

KRFP4821 1.24407337759479

KRFP3643 1.24392543745722

FP793 1.24379032581146

FP700 1.24329442556588

AD2D235 1.24310289284258

FP538 1.24295145048568

FP768 1.24190668700326

PubchemFP490 1.24174474726472

AD2D705 1.24107748395779

FP332 1.2407959527248

AD2D239 1.23972993761113

KRFP4852 1.23855373943416

APC2D8_O_O 1.23845387608382

KRFP3647 1.23817609073608

FP893 1.23710616951071

APC2D4_N_N 1.23634755375628

KRFP4122 1.23619235636558

SubFPC285 1.23611764539127

PubchemFP399 1.23559061713256

GraphFP385 1.23547621729989

FP140 1.23427346738919

KRFP3768 1.23368227858411

APC2D5_N_O 1.23347084378426

GraphFP387 1.23300216188548

PubchemFP488 1.23199025379262

FP173 1.23128940176903

AD2D480 1.22989040985865

FP991 1.22968048185481

APC2D2_X_X 1.22933837949936

SubFPC279 1.22929963028096

FP108 1.22887361719617

GraphFP671 1.22872347091105

PubchemFP392 1.22809707211483

GraphFP948 1.22744733681373

PubchemFP599 1.22730230624929

GraphFP394 1.22724344620919

GraphFP864 1.22632798637054

FP203 1.22619936663399

MACCSFP85 1.22541439739757

MACCSFP51 1.22531237101184

GraphFP97 1.22342174940365

FP366 1.22323759748847

FP685 1.22298842265152

FP1 1.22286274488745

KRFP3410 1.22190580886135

GraphFP163 1.22165613585027

SubFPC281 1.22033180053621

FP548 1.22013940474384

FP121 1.2201134398805

KRFP467 1.22007989872025

FP641 1.22001083576489

SubFP179 1.21940000040602

KRFP3948 1.21938958488315

PubchemFP659 1.21894418576183

GraphFP455 1.2185129348669

KRFP3795 1.21843376025951

FP957 1.21828827499448

AD2D157 1.21810767603909

PubchemFP401 1.21807115460276

GraphFP100 1.21689029840345

FP147 1.21560169420597

GraphFP446 1.21488788428713

FP753 1.21462213201324

MACCSFP125 1.21414373073699

PubchemFP556 1.21334317865399

SubFP4 1.2124431822588

AD2D548 1.21218845055038

FP506 1.2118351615459

FP594 1.21165013000029

GraphFP588 1.21125468444326

GraphFP641 1.2106811511196

PubchemFP356 1.20936628643994

GraphFP553 1.20875088781812

FP367 1.2082723712188

PubchemFP499 1.20810692881019

GraphFP996 1.207637239652

KRFP3733 1.20678805829727

FP340 1.20599666507554

PubchemFP115 1.20507827329205

GraphFP662 1.20501051323573

PubchemFP667 1.20479542518809

FP309 1.20398084088

KRFP3655 1.2036774236259

EStateFP11 1.20361868667587

GraphFP617 1.20263739734234

FP417 1.20212253299917

FP566 1.20208532884614

FP144 1.2018046134076

MACCSFP75 1.20166471356682

PubchemFP434 1.20110561990194

FP1022 1.20078436086497

FP134 1.19957012980974

PubchemFP559 1.1993312963594

FP945 1.19843772241465

GraphFP794 1.1984127172065

FP62 1.19759537800561

FP214 1.19753585433194

GraphFP548 1.19689219373466

PubchemFP376 1.196579501881

FP905 1.19614845939008

PubchemFP566 1.19577645736121

PubchemFP690 1.19478917921173

PubchemFP580 1.19433571084102

FP161 1.19343353289483

FP63 1.19304460727787

PubchemFP519 1.1929168715186

FP782 1.19264871814058

FP631 1.19258886710268

PubchemFP709 1.19242371216188

PubchemFP353 1.19095895253567

FP465 1.18934459725138

AD2D317 1.18904975293434

APC2D6_O_P 1.18895304230379

AD2D718 1.18846865648245

PubchemFP18 1.18719475977768

GraphFP817 1.18719093559568

GraphFP905 1.18699079860007

FP531 1.18690145137397

FP599 1.18670175594928

AD2D80 1.18623256983182

PubchemFP287 1.18517616341721

FP510 1.18508096645592

FP564 1.18475792788076

KRFP3436 1.18473783347626

FP923 1.18463033749117

PubchemFP407 1.18452497916104

MACCSFP118 1.18407973553488

KRFP4408 1.18394572691305

FP682 1.18352368379345

AD2D551 1.18332727952907

FP836 1.18327230631332

SubFP274 1.18321968285452

GraphFP737 1.18268256724875

GraphFP354 1.18261184714796

AD2D83 1.17994560791071

GraphFP397 1.17975179937662

FP907 1.1795774112047

FP312 1.17946056175786

GraphFP31 1.17942604687165

FP803 1.17930028040326

FP627 1.17879102948258

AD2D470 1.17830018733953

KRFP4817 1.17807931909234

MACCSFP78 1.17752627898398

PubchemFP691 1.17575919674619

AD2D704 1.1754951252281

FP385 1.17524829246992

PubchemFP567 1.17443331224276

KRFP302 1.17418987911358

FP496 1.17390979633959

FP597 1.17339678406971

PubchemFP26 1.17278600978071

GraphFP949 1.17207593858685

FP611 1.17106370779871

FP396 1.16919409348391

PubchemFP685 1.16914351694606

FP320 1.16847794271476

APC2D9_C_N 1.16831706556945

FP219 1.16784438915378

MACCSFP115 1.16692550824555

MACCSFP98 1.16642293762818

GraphFP381 1.16625408120662

FP978 1.16613687525989

FP431 1.16449269493784

GraphFP99 1.16442396953067

PubchemFP368 1.16376364092213

KRFP3709 1.16354469195572

FP223 1.16347149105474

GraphFP443 1.16344276910779

FP185 1.16285516746076

FP601 1.16283532663676

GraphFP277 1.16211542182255

FP720 1.16175751158843

MACCSFP3 1.16146819053096

FP475 1.16134426666459

FP209 1.16100769084391

FP407 1.16046654913492

GraphFP256 1.16031207835987

GraphFP359 1.16013000519807

KRFP3775 1.1598570290866

AD2D312 1.15812516188225

GraphFP72 1.15621756990796

KRFP347 1.15557753516878

FP757 1.15552472773256

KRFP4671 1.15552354461631

GraphFP874 1.1548992888535

KRFP3740 1.15447858673996

KRFP297 1.1536103961677

FP864 1.15297940775202

PubchemFP335 1.15249770863932

SubFPC86 1.15156974845921

PubchemFP624 1.15139652863713

FP339 1.15138012199747

PubchemFP334 1.14969434530579

KRFP8 1.14943546669827

FP51 1.14865160504043

FP925 1.14861772082649

PubchemFP65 1.14841413128184

FP497 1.14829378312571

FP612 1.14700535416025

GraphFP840 1.14659868855271

APC2D7_O_X 1.14473668794482

FP569 1.14471697126298

GraphFP195 1.14320980962524

GraphFP329 1.14249660152319

MACCSFP40 1.14160697861037

KRFP3662 1.14097364505272

FP533 1.14092958542831

GraphFP791 1.14071582789687

GraphFP338 1.14060103733328

GraphFP894 1.14051645119179

FP576 1.13793075156468

FP204 1.13772231353119

FP962 1.13768073287349

PubchemFP640 1.13758935467768

FP379 1.13707208152542

FP777 1.13697551095499

FP487 1.13697333935633

GraphFP885 1.13682267803881

AD2D319 1.13656639474784

PubchemFP756 1.13629162914984

GraphFP412 1.13593372931068

FP773 1.1356687884512

FP817 1.13549904545485

FP1021 1.1349143498672

GraphFP456 1.13439639141151

GraphFP549 1.13385144195562

PubchemFP700 1.13345836295484

GraphFP775 1.13334254541276

GraphFP283 1.13296274018689

GraphFP120 1.13226068336136

SubFP13 1.13184164011435

FP598 1.13073191126673

FP243 1.13040331603341

FP388 1.13014975601611

PubchemFP458 1.12991344494144

PubchemFP453 1.12927919951956

FP916 1.12867942277748

GraphFP1019 1.12811988577316

FP951 1.12802937968776

FP878 1.12745907148992

KRFP3400 1.12745689586148

FP106 1.12703738788316

KRFP3661 1.12693458042507

FP929 1.12643042788461

GraphFP942 1.1256757704103

FP657 1.12328030786624

FP369 1.12294897405678

KRFP4499 1.12221333043757

FP342 1.12203529271384

GraphFP92 1.12174332114444

SubFP23 1.1216406785784

FP43 1.12144163547304

GraphFP698 1.12105985254148

GraphFP867 1.12102297818907

FP436 1.12075091121142

MACCSFP95 1.12069304957338

FP429 1.12048173223801

PubchemFP593 1.12036603988087

GraphFP153 1.12029546676364

FP273 1.12018449116472

FP139 1.11975802144917

KRFP3388 1.11965342839884

KRFP3150 1.11865788381582

FP8 1.11805317181944

FP952 1.11747256499992

FP260 1.11718083030497

PubchemFP31 1.11658710486621

FP488 1.11629554522382

FP71 1.11595369294434

PubchemFP449 1.11586740658545

FP583 1.11580236785237

FP190 1.11537523788843

FP717 1.11518703148657

FP435 1.1146253950718

KRFP3635 1.1146199526027

FP24 1.11442500025014

FP977 1.11437250450321

FP915 1.11374968243035

MACCSFP106 1.11280927795625

FP824 1.11272587000986

FP628 1.11211807105775

GraphFP357 1.11176244703654

PubchemFP819 1.11145580095984

FP575 1.11074120937491

FP659 1.11029978163326

GraphFP823 1.10911501876625

SubFP280 1.10910575844858

FP247 1.10799628844595

FP890 1.10767449263018

PubchemFP704 1.10711396792414

FP626 1.10696032185992

AD2D248 1.10682496681383

KRFP4495 1.10671352223796

AD2D395 1.10585443909031

FP673 1.10569495417212

FP884 1.10568828309571

FP756 1.10499681440975

FP202 1.10482912409805

FP456 1.10479733906021

FP557 1.10475923784889

AD2D136 1.10466671232972

GraphFP281 1.10461542898487

FP197 1.10435363848034

FP16 1.10382466822433

FP975 1.10359456875701

PubchemFP21 1.10321361807778

FP298 1.10319067011203

PubchemFP349 1.10264472007245

APC2D3_C_Cl 1.10233065368419

FP192 1.10157386256357

APC2D3_C_S 1.10096797676274

KRFP1154 1.10074448767426

GraphFP44 1.10040160697688

FP438 1.10027442932174

GraphFP422 1.10004649648147

FP103 1.0991937218411

KRFP3725 1.09906516607942

FP452 1.09790385589161

FP651 1.09783201737932

GraphFP396 1.09770858138715

FP113 1.09718925774854

KRFP4843 1.09596919277645

GraphFP309 1.09496538864486

FP178 1.09486274141443

PubchemFP682 1.09371175511973

FP584 1.09358022549949

GraphFP288 1.09281324032833

MACCSFP80 1.09231895423843

FP845 1.09136069321306

KRFP342 1.09094648932603

SubFPC15 1.09053682537709

FP837 1.08986072320923

KRFP494 1.08976227410117

PubchemFP476 1.08736875604256

FP763 1.08706457340513

PubchemFP545 1.08635564778493

FP292 1.08626994209057

GraphFP683 1.08583661847848

GraphFP211 1.08564649556897

APC2D6_C_F 1.08553114662523

GraphFP384 1.08537044187562

SubFPC13 1.08516078755599

KRFP1405 1.08452737187825

KRFP3786 1.08407412414427

MACCSFP127 1.08390685195765

SubFPC280 1.08352786254951

GraphFP474 1.08348537969988

GraphFP634 1.08264272793795

PubchemFP604 1.08251306275034

KRFP126 1.08242633692169

FP145 1.08182507096311

FP141 1.08063321347628

FP993 1.08021119888061

FP926 1.07961192880984

FP660 1.07952824333711

FP835 1.07934752222684

KRFP3957 1.07908916193804

FP912 1.07886837255325

FP842 1.07849362203356

PubchemFP803 1.07769661418606

FP250 1.07742641676642

AD2D7 1.07663012874344

KRFP3659 1.0761993245062

FP500 1.07543562315256

MACCSFP117 1.07538896147946

GraphFP575 1.07504037950142

FP56 1.0749585862088

PubchemFP633 1.07438897892353

GraphFP358 1.07292757660673

PubchemFP550 1.07263253385381

GraphFP940 1.07258688995026

FP277 1.07223221286451

FP505 1.07193019394382

PubchemFP395 1.07176464141591

AD2D626 1.07114866223138

FP445 1.07097508655686

GraphFP473 1.0708801119202

FP238 1.06992191794479

KRFP3757 1.06744197194565

FP41 1.06718921162703

GraphFP945 1.06710065693645

AD2D163 1.06627654467492

GraphFP631 1.06510150994238

GraphFP966 1.06345131744665

PubchemFP546 1.06322848552475

KRFP678 1.0618208283168

GraphFP476 1.06169678538464

PubchemFP596 1.06135877684868

FP337 1.06118722043697

PubchemFP413 1.06067676454157

FP375 1.06037255896519

GraphFP232 1.06036987878415

GraphFP478 1.06034821670357

FP194 1.06028567613944

APC2D5_C_Cl 1.05992286112047

FP967 1.05980888181365

FP634 1.05953571727088

SubFPC237 1.05921407055585

SubFP224 1.05901488464401

PubchemFP439 1.0590000610525

GraphFP289 1.05860552092692

PubchemFP118 1.05836274896153

PubchemFP431 1.05762728516454

KRFP3889 1.05760549456992

PubchemFP642 1.05734731593611

PubchemFP37 1.05729116228907

KRFP4818 1.05701378360925

GraphFP699 1.05679359196997

EStateFP26 1.05665962857252

KRFP3719 1.05658807625455

AD2D91 1.05612254979884

FP341 1.05595815971059

PubchemFP487 1.05588020987368

KRFP138 1.05586034690815

FP365 1.05584296042211

GraphFP678 1.05455296435563

KRFP890 1.05430240882051

FP328 1.05366708630959

KRFP1448 1.05363144229754

GraphFP436 1.05358751257587

GraphFP89 1.05340012299442

GraphFP117 1.05333211500318

FP256 1.05309956094277

FP982 1.05198015060541

PubchemFP527 1.05197803152888

GraphFP520 1.05127422176872

FP467 1.05094414674219

GraphFP174 1.05051135222179

APC2D2_N_X 1.05050836693383

FP579 1.04968957354886

SubFPC286 1.04956781197779

KRFP2260 1.04913852971201

FP976 1.04878748546316

GraphFP668 1.04817520714566

GraphFP188 1.0481350958017

FP453 1.0480329658189

GraphFP772 1.0475388279563

KRFP4286 1.04713109141833

AD2D93 1.04677969887572

KRFP3716 1.04641234319957

FP980 1.0456728230835

FP120 1.04534615893276

FP258 1.04526561051644

KRFP654 1.04475350186865

FP642 1.04451368113449

SubFP291 1.04411315704615

KRFP287 1.04409632685733

PubchemFP620 1.04405541208971

PubchemFP607 1.04261241529255

EStateFP9 1.04206704236098

PubchemFP46 1.04196398576149

FP512 1.04161496578673

KRFP1418 1.04149808849026

GraphFP123 1.0411756446528

KRFP20 1.04044243451643

FP1015 1.04029276388729

KRFP4032 1.03959747881202

SubFP307 1.03942352981742

PubchemFP840 1.03916074626788

FP414 1.03883073741301

FP344 1.03831211708936

FP897 1.03764677162316

GraphFP914 1.03761736505725

AD2D482 1.03746062452949

GraphFP514 1.03741054668161

APC2D5_N_F 1.03702373729714

GraphFP564 1.03609592007894

SubFP5 1.03579903208625

FP1014 1.03542309698154

PubchemFP333 1.03532190079813

FP112 1.03530548512098

FP362 1.03472696570741

FP714 1.03457801990134

GraphFP881 1.03456867989711

GraphFP339 1.03357062499916

KRFP605 1.03296236755408

KRFP4810 1.03247580833648

FP58 1.03226449482194

KRFP398 1.03212238616232

SubFP246 1.03185520692443

FP831 1.03179337894733

MACCSFP38 1.03162168840461

FP378 1.03160949076953

SubFP96 1.03152958339663

FP968 1.03146094357127

KRFP3722 1.03103919101573

GraphFP131 1.02994234490344

MACCSFP47 1.02914264337065

GraphFP386 1.02897666484103

FP931 1.02884224385925

FP943 1.02810554848849

AD2D501 1.02705700456424

FP415 1.02601302618653

KRFP3396 1.02492953946526

FP88 1.02374803617457

KRFP3010 1.023714681474

SubFPC246 1.02330984142426

APC2D9_C_P 1.0232273954705

FP812 1.02304043266789

GraphFP18 1.02259649696425

GraphFP925 1.02225998738468

FP80 1.0221125710639

FP89 1.02202517852923

FP733 1.02199196509732

PubchemFP611 1.02188764542665

FP553 1.02186916562474

FP704 1.02130925752364

FP511 1.0208037346163

KRFP3937 1.02044064247489

GraphFP711 1.02016536501682

FP330 1.0195042954693

FP485 1.01915890166274

FP276 1.01892200430824

APC2D2_C_F 1.01881566530067

MACCSFP77 1.01847021539173

FP620 1.01817294652385

FP879 1.01771156360236

GraphFP465 1.01767706849819

KRFP677 1.01722106454309

FP28 1.01660579531578

GraphFP822 1.01603367281473

PubchemFP199 1.01508740900405

GraphFP909 1.01502880808695

FP295 1.01476588084702

PubchemFP418 1.01467211612928

FP1009 1.01407090356875

MACCSFP43 1.01386970363755

PubchemFP701 1.01376269318119

SubFPC303 1.01365862187085

FP69 1.01309429763895

GraphFP768 1.01304833521779

KRFP1160 1.0121465997651

FP632 1.01141758756404

PubchemFP285 1.01131026578188

GraphFP137 1.01112136715104

GraphFP736 1.01107633274771

FP259 1.01052022855759

KRFP3942 1.01051285844515

KRFP2986 1.00907117617256

AD2D495 1.00704070690833

FP373 1.00671982890788

FP979 1.00652424643971

FP317 1.00595318745665

AD2D325 1.00554455910503

FP169 1.00509370516199

KRFP3946 1.0043544012346

FP580 1.00412973736576

SubFP282 1.00364587018393

FP40 1.00340555558982

KRFP299 1.00283292683795

FP350 1.00244894407761

SubFP303 1.00235357763237

GraphFP279 1.00213091433974

PubchemFP502 1.00201823608627

APC2D8_O_X 1.00191704056967

FP12 1.00154950220611

FP614 1.0011587688047

FP636 1.00075846375088

FP296 0.999391278404905

FP846 0.999384720543533

FP286 0.999140304463542

GraphFP554 0.999136125105505

GraphFP890 0.998667884160124

KRFP4678 0.997749510460471

KRFP3781 0.996855559599296

GraphFP151 0.996465885345487

PubchemFP437 0.996292251828066

FP346 0.995959115661661

FP675 0.995656588636937

FP154 0.994775403106143

FP490 0.994095669656219

FP29 0.991854028243802

FP257 0.991759909302816

FP13 0.991468391218927

KRFP671 0.991324704487045

GraphFP813 0.991258508230383

GraphFP154 0.991238582117566

GraphFP785 0.991091111400083

FP92 0.99091039989581

FP268 0.990154197873307

GraphFP353 0.990012095864541

GraphFP203 0.989457665202349

GraphFP149 0.988584595724986

FP78 0.988449713233566

APC2D10_C_N 0.988391272446972

FP313 0.98824798598724

FP74 0.987988735052986

GraphFP716 0.987429106517501

PubchemFP446 0.987362610357221

APC2D7_N_O 0.987108497525009

GraphFP340 0.986270273951333

FP221 0.986095252970294

AD2D558 0.985899047075472

KRFP3785 0.985866460718395

GraphFP932 0.985710116555443

KRFP3809 0.985420428800864

MACCSFP60 0.985207858622374

KRFP2548 0.985203512865822

KRFP2308 0.984949722826434

APC2D4_N_S 0.983879621645634

FP91 0.983637774284655

FP635 0.983022736995151

APC2D3_C_F 0.982823299070273

PubchemFP372 0.982527353301604

GraphFP164 0.981904951335792

PubchemFP300 0.981674790654691

FP310 0.981643596661871

PubchemFP347 0.981464935573305

AD2D13 0.980832482102402

GraphFP608 0.980375306355491

FP621 0.979816312959776

EStateFP48 0.979308467317836

GraphFP712 0.978701373434141

GraphFP674 0.977977506480559

GraphFP734 0.977171331777949

FP464 0.976544968514689

FP658 0.976296468012588

GraphFP94 0.976107208356672

MACCSFP58 0.975977263524301

GraphFP908 0.975960912217882

SubFP285 0.975492835726751

SubFPC291 0.975284279838364

FP573 0.975098426080372

FP50 0.974786798705911

SubFP286 0.974518369055541

FP285 0.974219310449822

GraphFP441 0.9736858217867

KRFP4015 0.973540538051108

FP749 0.973178174890591

PubchemFP656 0.973160186599105

KRFP3718 0.973145680474866

FP360 0.973127077757656

FP902 0.972803810008511

GraphFP913 0.971571393412744

FP358 0.971532340541589

KRFP3120 0.971107389728979

FP177 0.97054548786522

FP568 0.970376655682986

FP403 0.969470320386686

FP855 0.968720806306378

PubchemFP539 0.968074155189732

FP629 0.967331385245473

SubFP295 0.967064593548537

PubchemFP671 0.966923423420133

GraphFP779 0.96678299056109

FP799 0.966756801361057

GraphFP102 0.966484965483204

SubFPC171 0.966454879683349

PubchemFP702 0.96502197326779

AD2D4 0.964876306387459

FP841 0.964774457854873

SubFPC135 0.96461550026504

GraphFP491 0.964615455163102

SubFPC27 0.96457127192242

FP875 0.96448220844082

FP662 0.964097051990392

FP602 0.964040528540464

APC2D6_C_P 0.963985745553853

PubchemFP549 0.96390737297885

FP372 0.963754524401244

FP38 0.963396917879819

FP815 0.963128762103029

FP418 0.9631196014297

GraphFP540 0.962622620781253

KRFP3609 0.962592706866027

AD2D397 0.962378826180468

MACCSFP145 0.962369623407333

APC2D9_C_S 0.96222958896973

GraphFP672 0.961903005914871

FP420 0.961479021545476

GraphFP695 0.961092680567694

GraphFP206 0.961035711324277

KRFP3894 0.960602739036315

FP1004 0.960256879659528

KRFP3699 0.960091458707534

FP908 0.959083136788796

GraphFP145 0.958844731921195

SubFPC43 0.958350462672082

GraphFP776 0.958253252868569

AD2D629 0.958098997934196

FP918 0.957843596091981

GraphFP321 0.957505394881738

FP530 0.957354201705432

MACCSFP113 0.957072898820466

KRFP326 0.956958776299626

PubchemFP386 0.956248432166701

GraphFP879 0.956192572633536

FP289 0.955694516484492

FP524 0.955566765284684

GraphFP893 0.955541655532628

PubchemFP183 0.955319650015242

GraphFP50 0.95515213948387

GraphFP225 0.955080941485753

GraphFP536 0.954957060955238

GraphFP576 0.954891863499917

FP15 0.954704307291321

FP60 0.954084251422931

MACCSFP140 0.953459620455867

GraphFP112 0.953077704174511

PubchemFP351 0.952968422487672

KRFP2598 0.952954097007544

FP297 0.952950553266763

KRFP438 0.952882082382932

MACCSFP52 0.952812013307361

FP998 0.952237769130201

PubchemFP839 0.951767845948158

GraphFP873 0.95169918188734

GraphFP105 0.951681232759985

AD2D121 0.951562059677608

FP974 0.951550888720348

FP463 0.951042390406716

SubFP276 0.950684000301444

FP79 0.950205768238015

FP725 0.949725238343196

PubchemFP734 0.949352818922305

GraphFP179 0.949226242546011

AD2D162 0.948541577147059

FP734 0.948363578972145

PubchemFP400 0.948006752393504

GraphFP958 0.946604273947408

FP137 0.946527849743083

FP787 0.946191080214629

PubchemFP33 0.945442770530215

GraphFP450 0.945323343981039

FP491 0.945189667791544

FP940 0.94422850358119

FP1023 0.943626780909116

GraphFP489 0.943494217703158

KRFP2306 0.942870320897914

FP961 0.942594836079614

PubchemFP721 0.942578659215475

FP823 0.942374323193523

FP707 0.942321363264766

PubchemFP798 0.942245856336354

KRFP608 0.942015365696912

FP97 0.9417521266922

APC2D2_O_S 0.940662772106379

GraphFP484 0.940399762002164

APC2D5_C_S 0.93898314440412

FP877 0.938946502135392

KRFP4531 0.938934396256701

MACCSFP135 0.938222397613115

KRFP383 0.937882578797252

EStateFP28 0.937468657779719

MACCSFP42 0.936905061746674

KRFP2855 0.936667367190795

KRFP3657 0.936662970060611

PubchemFP186 0.935914843349475

FP9 0.935779286049851

GraphFP27 0.935712356533147

GraphFP888 0.935184336904268

FP838 0.933447982472113

GraphFP1000 0.933444735031075

FP996 0.933180902020828

FP205 0.932414144423532

KRFP3713 0.932095779429286

PubchemFP323 0.931960637474085

GraphFP751 0.931813289034676

FP124 0.931653567949463

APC2D5_O_X 0.931579043871236

PubchemFP20 0.93103846478778

SubFPC85 0.930864174536727

KRFP2595 0.930069724294492

PubchemFP438 0.929142802412162

FP291 0.927915471514897

KRFP348 0.927848576580722

SubFP281 0.927625482850988

GraphFP659 0.927526825192163

PubchemFP548 0.927297970646881

GraphFP646 0.926700405939564

AD2D335 0.92637602413612

SubFPC99 0.925918637676187

AD2D345 0.925014249891045

SubFP279 0.923851463705629

KRFP1156 0.923520632568198

AD2D103 0.923372860030099

GraphFP849 0.923316299875998

GraphFP87 0.922569333929068

APC2D8_C_P 0.922257985159506

GraphFP610 0.921649172276076

KRFP4826 0.921547268496186

FP705 0.920920292957601

FP834 0.920689445189979

FP157 0.920459119070501

GraphFP761 0.919592786026272

FP731 0.918544553542243

FP899 0.918338220025257

GraphFP21 0.9179817098593

GraphFP254 0.916924273467558

FP472 0.916748759095879

PubchemFP523 0.916629679961769

FP677 0.916322146136961

GraphFP15 0.916275701590253

AD2D6 0.916123653065487

FP359 0.915892276820291

EStateFP25 0.915568352178133

FP508 0.915450104387569

FP527 0.914796418317947

PubchemFP589 0.914496053887961

PubchemFP293 0.914017592619616

KRFP480 0.914013852542673

FP539 0.913913155400025

FP381 0.91390418936669

GraphFP221 0.913290215661746

GraphFP295 0.912481793841844

GraphFP333 0.912127411652257

GraphFP609 0.910960210111529

GraphFP23 0.910562121823125

FP970 0.910229114406492

GraphFP701 0.909508449367557

FP684 0.909460302094157

APC2D1_C_Br 0.909222997154556

GraphFP324 0.908413803929935

PubchemFP555 0.908210703170834

FP476 0.907525922545265

FP68 0.907453758942176

GraphFP841 0.907000759068148

GraphFP746 0.905871977004818

AD2D33 0.905723226523079

FP254 0.905163829860097

FP217 0.903935929459932

FP226 0.903848113120559

GraphFP652 0.903661826984864

SubFP174 0.90310514310951

PubchemFP387 0.903064761000106

KRFP3648 0.902431642616688

APC2D1_C_Cl 0.902152632741748

FP86 0.901223759152434

GraphFP116 0.901157698433261

SubFP190 0.901156930780384

PubchemFP336 0.901147273944971

AD2D628 0.901132284526598

FP694 0.900220469869499

KRFP3351 0.899945583602356

KRFP3682 0.899509256750851

MACCSFP7 0.898927478166047

SubFP43 0.898908219331129

KRFP2651 0.89889935292908

FP329 0.897255079726401

AD2D423 0.897171444097336

FP90 0.896700527609616

KRFP3404 0.896662918455859

FP184 0.896467565458773

PubchemFP256 0.896442663296537

FP794 0.896104657401488

FP356 0.896052266274127

KRFP2882 0.895954154163749

APC2D6_C_Cl 0.895024998146201

GraphFP740 0.894829024489686

GraphFP780 0.89436184088947

GraphFP702 0.893247231172843

PubchemFP638 0.89322758599782

SubFP181 0.893209116857082

AD2D560 0.893119268274153

GraphFP838 0.892962263175128

KRFP4666 0.892878910661909

GraphFP886 0.892635743665265

GraphFP152 0.892555585041612

PubchemFP255 0.892476057454691

PubchemFP569 0.891480977404749

FP654 0.891358985554126

FP422 0.891303923188051

FP687 0.891202764852687

AD2D403 0.890968956915437

GraphFP275 0.890883356043202

GraphFP90 0.890865641162075

GraphFP957 0.890342475204602

GraphFP773 0.889868308759377

GraphFP458 0.889696917859806

FP270 0.888745958560509

FP639 0.888724375759839

APC2D4_C_F 0.888005077480355

AD2D416 0.887804542791523

SubFPC109 0.887282278449834

FP49 0.886771432220657

GraphFP270 0.886562554821487

GraphFP835 0.886466366879268

GraphFP550 0.886458398942509

GraphFP229 0.886017461413683

PubchemFP148 0.88601647541369

AD2D473 0.885829981279634

GraphFP20 0.885740929037252

GraphFP411 0.885154054699902

PubchemFP403 0.8845985654319

FP780 0.884475761765851

GraphFP600 0.883943546832417

SubFP143 0.883257944715613

KRFP4829 0.882443820124424

PubchemFP257 0.881559139184532

APC2D2_N_S 0.881018345234241

PubchemFP338 0.88047797059858

PubchemFP358 0.880313521072335

FP965 0.880219834987681

FP886 0.880028901435874

AD2D707 0.879946351973238

FP616 0.879766834081086

FP911 0.879465780603116

FP261 0.878452622532012

FP754 0.87833935876319

FP729 0.87745551981068

FP807 0.876568115478441

PubchemFP16 0.876104081652152

KRFP3742 0.875272209933645

FP61 0.874284033516936

GraphFP825 0.874207173806701

GraphFP803 0.874090272195885

PubchemFP568 0.873415036556055

FP401 0.87325661687578

GraphFP173 0.873227508678432

PubchemFP683 0.873138206147244

APC2D2_Cl_Cl 0.873052040452948

FP10 0.872790863661211

GraphFP682 0.872661813042263

FP710 0.872484319707527

FP188 0.872084421947099

GraphFP846 0.872046038569302

MACCSFP65 0.871749313592634

FP885 0.871586826839888

FP988 0.870668321029645

PubchemFP542 0.869748713992804

GraphFP902 0.869681502675194

FP663 0.869455275699316

EStateFP32 0.868815048187596

FP370 0.868735773798407

KRFP3419 0.86851829267027

FP119 0.867658535516993

SubFPC26 0.867459470388258

FP158 0.867330273931316

AD2D706 0.866801836933035

SubFP47 0.866710910073053

FP186 0.865509883515701

KRFP4296 0.864614655485829

GraphFP517 0.86377858560769

GraphFP48 0.863744355519368

MACCSFP74 0.863458964034112

FP697 0.862730047716447

FP552 0.862147475399356

FP605 0.862026915244054

FP272 0.861135942259462

GraphFP710 0.861075631760618

KRFP4294 0.860493865370253

PubchemFP389 0.86020230093643

KRFP4293 0.859976528177253

KRFP290 0.859846319801616

KRFP506 0.859632760113515

PubchemFP357 0.858964860344881

APC2D4_C_Cl 0.858894288896831

FP434 0.858748188302149

FP906 0.85862123243626

KRFP161 0.858465908630824

GraphFP521 0.858339047398955

KRFP2264 0.85814330933914

FP917 0.858129542879377

GraphFP714 0.857310821930842

APC2D3_N_P 0.856668993842497

GraphFP28 0.856533757740929

KRFP3914 0.856393637483641

GraphFP681 0.856316665658731

PubchemFP501 0.856159124098143

FP319 0.856106190222875

APC2D1_N_N 0.855994033336152

FP263 0.855805205329321

GraphFP435 0.855662606750286

FP492 0.855557633269542

FP172 0.855553597882762

GraphFP508 0.855124934215884

MACCSFP35 0.854985514167445

GraphFP866 0.854858285557328

APC2D7_O_I 0.854800963328003

MACCSFP162 0.854314027401333

SubFP6 0.85335332442772

KRFP3707 0.853345035975489

APC2D10_N_P 0.853140637773782

KRFP3820 0.852985209205083

GraphFP418 0.852966497898856

APC2D8_C_X 0.852810904820343

PubchemFP585 0.852561810420577

FP130 0.852387197554029

AD2D657 0.852178658540496

SubFP26 0.852131364915063

PubchemFP51 0.852003459389828

PubchemFP206 0.851113331308266

KRFP4667 0.850951898645396

FP896 0.850901802926658

GraphFP355 0.850750401987886

GraphFP946 0.850706168476781

PubchemFP572 0.850360099741448

SubFPC180 0.849697862899161

PubchemFP435 0.849370647291072

FP752 0.848835386060685

GraphFP231 0.848807743458435

GraphFP472 0.848686295747184

KRFP4522 0.847750149153785

FP3 0.847598778997643

GraphFP677 0.847519191488573

PubchemFP181 0.847194872103392

PubchemFP189 0.847111986106931

FP30 0.846995240594489

FP474 0.846567003260173

APC2D4_N_P 0.846549203606058

AD2D160 0.846415921361859

FP686 0.846264202640117

PubchemFP457 0.846114339183867

GraphFP14 0.846105107177482

FP318 0.845815196843655

GraphFP598 0.845551881366172

FP588 0.845333131840465

FP411 0.845318738946944

FP986 0.845229554080082

FP676 0.845216023485487

FP82 0.845038442519537

PubchemFP651 0.844611409026084

GraphFP424 0.843644055000414

APC2D10_N_N 0.843089186703782

KRFP393 0.842696791131238

FP797 0.842647061656641

MACCSFP56 0.842465799685693

GraphFP302 0.842274269351203

KRFP3402 0.842014425723495

FP248 0.841788249970629

FP377 0.841742468165548

FP607 0.840481215339975

FP99 0.840463910235909

GraphFP828 0.839647132000679

GraphFP180 0.839407180337676

GraphFP433 0.839246494095359

FP384 0.838884862890908

FP428 0.837909044576285

KRFP3398 0.83706920951569

FP868 0.836476451092389

FP376 0.835329808357214

GraphFP268 0.834528630616934

GraphFP618 0.834478584466423

GraphFP852 0.833886936218065

FP969 0.832428185595137

GraphFP587 0.832191789724788

FP615 0.831981015864731

PubchemFP43 0.831914887936327

FP555 0.830280380719377

AD2D340 0.83016114727703

FP325 0.829740932536523

PubchemFP146 0.828781451414493

FP439 0.827805459211049

KRFP3392 0.827185025997711

FP843 0.827166299123528

GraphFP987 0.826470240980948

FP421 0.826430910588089

GraphFP944 0.825290567097424

SubFPC143 0.824669483026427

KRFP3735 0.824273546728779

AD2D472 0.824271662561477

GraphFP1002 0.823666124992973

MACCSFP61 0.823236782947735

GraphFP252 0.823066450057942

GraphFP706 0.823013581895255

FP228 0.822954630625258

KRFP3739 0.822500712429622

GraphFP493 0.821417242182384

FP960 0.821235289004898

FP701 0.820878895870316

GraphFP335 0.819687844614277

FP220 0.818921589751299

PubchemFP381 0.81883697266358

APC2D1_C_I 0.818301686108435

FP383 0.817781948122843

PubchemFP484 0.817427578717795

FP990 0.817015603370674

GraphFP880 0.817007062340831

GraphFP129 0.816907056134126

SubFPC284 0.81665381837682

PubchemFP711 0.816506880377401

AD2D8 0.81638619190817

EStateFP38 0.816384557700438

GraphFP368 0.816366207121231

GraphFP973 0.816183192470906

APC2D3_X_X 0.816167063549989

AD2D183 0.816098979127051

SubFPC209 0.815886139250825

GraphFP136 0.815455729603322

GraphFP1007 0.815202739803196

GraphFP118 0.814755237780943

FP747 0.814530844318286

FP558 0.813675669186997

AD2D84 0.813033942244797

PubchemFP495 0.812611900946369

FP501 0.812610467727831

FP515 0.812365283257093

PubchemFP861 0.811084735540503

FP275 0.810704614399886

AD2D267 0.810405440744594

SubFPC188 0.810393436640083

MACCSFP46 0.810320389901779

GraphFP555 0.809087465145794

AD2D726 0.80893491402937

GraphFP796 0.808626012539206

FP709 0.80776709584118

FP801 0.807506390721386

PubchemFP445 0.806924878742259

FP582 0.806579188324534

FP321 0.806475505390416

FP142 0.805747090304615

GraphFP296 0.805384635515724

GraphFP693 0.802698019026912

FP738 0.802484416817174

GraphFP167 0.801946230615381

GraphFP356 0.801939485278935

FP165 0.801735560715145

GraphFP483 0.801009538921274

GraphFP731 0.800299432432646

APC2D1_C_F 0.800230398919966

KRFP350 0.799467986976323

FP427 0.79916881912306

APC2D7_O_P 0.799156628652635

FP861 0.798650083451277

PubchemFP654 0.798550462004845

FP468 0.798529711356128

GraphFP34 0.79801541910572

GraphFP481 0.79732553172971

PubchemFP600 0.796971264380326

KRFP557 0.796853854521341

EStateFP75 0.795953400658086

GraphFP200 0.795214418981107

KRFP410 0.795004471967039

KRFP3649 0.794655664407851

KRFP18 0.794628514056433

GraphFP224 0.794297790541891

FP323 0.793824616144464

GraphFP480 0.793480952355093

FP54 0.793427617691407

APC2D10_C_P 0.792908459315607

KRFP354 0.791884671964434

GraphFP891 0.791664135455185

SubFP237 0.790165339793911

FP518 0.788917911640886

FP175 0.788720672052326

FP674 0.78858778751932

PubchemFP441 0.787814193326997

GraphFP875 0.787396052365072

MACCSFP133 0.786619059831865

KRFP983 0.786447883523573

GraphFP979 0.786407932714569

GraphFP244 0.786285429868805

FP545 0.785813661540652

FP215 0.785285353815597

GraphFP642 0.78484787096146

FP680 0.784816246928703

KRFP4292 0.784072539519113

GraphFP757 0.783751499092332

FP1002 0.783282706486429

GraphFP715 0.783154203602637

FP950 0.783018017598024

GraphFP512 0.782867128824115

PubchemFP375 0.782367627228575

KRFP3229 0.782114464855828

KRFP444 0.781853697627849

FP606 0.781808355075121

FP517 0.781772013364957

MACCSFP88 0.781672242784707

GraphFP566 0.781135765703027

KRFP310 0.781123861376702

PubchemFP209 0.781123091005092

KRFP3442 0.781110217441352

SubFPC6 0.781107605218975

GraphFP373 0.781104686986941

SubFP215 0.781102576240068

PubchemFP213 0.781063184088016

FP789 0.781052063791888

APC2D7_I_I 0.781032508610412

FP355 0.780642205139235

FP67 0.780553175041622

APC2D4_N_O 0.78026911210745

KRFP4834 0.780242073175695

GraphFP495 0.779603390107406

FP466 0.779300981458971

PubchemFP297 0.779025674996391

FP93 0.778984888575484

FP942 0.778950020378171

GraphFP247 0.778938758330642

KRFP4077 0.778486153952912

KRFP4175 0.778482683745907

GraphFP623 0.778468200207534

PubchemFP47 0.778450047228048

KRFP3228 0.778390379199907

SubFP32 0.7783536026421

FP732 0.778306644256661

KRFP3915 0.778297474134051

FP779 0.778110759529128

FP331 0.777979875241068

GraphFP762 0.777963363926385

FP363 0.777839437738269

MACCSFP18 0.777359309442939

PubchemFP427 0.777218882035454

GraphFP217 0.77662625278072

KRFP2773 0.776326677884385

FP284 0.775379652741917

EStateFP50 0.775317332458344

SubFP27 0.775182046156206

GraphFP431 0.774830215961889

GraphFP370 0.773688895756501

GraphFP523 0.773590351357837

KRFP3721 0.773434669471817

KRFP4205 0.772486243170169

FP408 0.771638163031448

GraphFP250 0.769614196648467

GraphFP700 0.769334681560042

KRFP840 0.769311618168594

FP266 0.768499876765281

AD2D714 0.767526162245539

GraphFP591 0.767410902414449

AD2D247 0.767370904845612

FP711 0.767156743038943

APC2D8_N_P 0.766526079405823

AD2D318 0.766216442109693

KRFP4665 0.766067551331575

GraphFP939 0.765741562770815

GraphFP722 0.765704497149958

PubchemFP362 0.76556803531715

SubFPC9 0.764845768736395

FP646 0.764569620175758

FP324 0.764522700658862

GraphFP57 0.763341160613975

FP589 0.763207598335136

KRFP363 0.762620339650637

FP822 0.762303746236107

KRFP3157 0.762249996669753

GraphFP169 0.7617029378627

KRFP476 0.761652548258533

FP690 0.759346869020762

AD2D241 0.759232997990447

GraphFP952 0.759221197154486

APC2D8_N_O 0.758715251884121

PubchemFP605 0.758459747344097

FP182 0.75794281994233

SubFP188 0.756427677280275

FP389 0.756072520814018

GraphFP559 0.755963228317673

GraphFP1013 0.755567301943983

PubchemFP652 0.755021222356701

KRFP3474 0.753483642922296

GraphFP464 0.752691482812756

FP400 0.752577493074393

SubFPC153 0.752439389467599

SubFP9 0.752376446366231

GraphFP315 0.751999504467934

GraphFP837 0.751838814146352

KRFP408 0.750929835784027

KRFP4757 0.750805686896257

PubchemFP191 0.750687245210669

GraphFP1018 0.750668179291019

APC2D4_I_X 0.75064946409373

FP719 0.75029209523617

PubchemFP454 0.749819369758988

PubchemFP621 0.749254627868231

FP100 0.74910614922001

PubchemFP465 0.748753136741449

KRFP3382 0.747658296679621

KRFP3815 0.747381557656676

GraphFP876 0.747033152503743

FP891 0.746848045670478

APC2D10_O_P 0.746791439179389

SubFPC181 0.746319424143996

GraphFP399 0.746259725789444

FP304 0.746117818128677

FP430 0.745846793039862

PubchemFP591 0.745666451467481

FP529 0.745492257810377

GraphFP29 0.744902744650574

PubchemFP66 0.74426812042154

GraphFP915 0.744258994477616

FP234 0.743962858319707

GraphFP462 0.743054175082837

GraphFP636 0.742670552045825

GraphFP582 0.742136005143067

FP230 0.740182873481626

FP1003 0.740066370063615

KRFP3641 0.739735343361222

FP827 0.739657036244517

GraphFP10 0.739639586232147

FP395 0.738657617538806

PubchemFP22 0.738638330865375

FP306 0.73789125967599

GraphFP6 0.737807961944633

GraphFP26 0.737739014073514

FP371 0.737426095807953

GraphFP261 0.737365442329943

GraphFP134 0.737306090471445

APC2D7_O_F 0.737198332995644

KRFP104 0.737178542128931

GraphFP8 0.737125523310648

PubchemFP329 0.737099880915021

FP347 0.736681876266284

GraphFP529 0.736282771076693

KRFP3550 0.736160405508089

APC2D7_C_S 0.736145201902341

MACCSFP39 0.735976762678639

FP45 0.73589945695121

PubchemFP203 0.735827462091696

APC2D4_O_S 0.735736325102034

FP591 0.735734683063533

FP964 0.735595021421704

FP14 0.735573635187783

FP648 0.735117426770287

AD2D728 0.734989666792126

FP735 0.734543930668672

GraphFP138 0.734494111880283

FP727 0.734348910930179

KRFP620 0.733924630601003

AD2D638 0.733819844833608

FP987 0.733681179016199

SubFPC8 0.733248931674242

KRFP3777 0.733061953038274

FP563 0.731890092686563

GraphFP477 0.731367413415135

AD2D240 0.730908464247736

KRFP390 0.730331756478071

FP997 0.729802423374893

FP262 0.729259594344449

FP168 0.729104827886033

GraphFP938 0.728740756546627

MACCSFP26 0.728450366016605

APC2D5_N_X 0.727592942777908

FP255 0.727105415842852

PubchemFP821 0.726893143313718

KRFP1150 0.726693988747877

SubFPC101 0.726528578610292

FP117 0.726084073573783

AD2D474 0.725065317618807

PubchemFP295 0.725000570201191

GraphFP639 0.724835353484975

GraphFP903 0.724552696961781

AD2D471 0.724139247261386

APC2D7_C_F 0.723998400593644

FP927 0.723983437482374

AD2D406 0.723346677669097

AD2D396 0.723280353322416

GraphFP732 0.722989135012991

AD2D552 0.722931875032146

KRFP4243 0.722744103423733

GraphFP950 0.722647683842057

KRFP1245 0.722627546671748

FP64 0.72239535562113

GraphFP951 0.722218795371907

GraphFP238 0.722041714748809

GraphFP619 0.721983456812769

PubchemFP686 0.721521793841401

FP919 0.721413757681574

APC2D8_O_P 0.721114597578341

GraphFP68 0.721027365460969

FP299 0.720411443606362

APC2D5_C_F 0.720018140672343

AD2D327 0.718413554494399

GraphFP552 0.71834405626023

GraphFP953 0.717998997930401

FP232 0.716679369628753

SubFP137 0.714791617296648

KRFP3434 0.714429502697848

FP643 0.713165041430091

KRFP1241 0.71309654223926

GraphFP717 0.712844202957079

KRFP2950 0.711852996178024

GraphFP83 0.710233574666817

FP412 0.710139569062713

AD2D242 0.71001837506642

FP565 0.70984517657062

GraphFP834 0.709398258927123

KRFP16 0.709268282649802

GraphFP809 0.708890003652259

AD2D475 0.708673943318031

FP448 0.707473310455021

SubFP169 0.707265240171273

FP609 0.706689431696955

PubchemFP612 0.705743635182546

FP416 0.705703661605821

AD2D433 0.705127215147148

GraphFP372 0.705013281423069

PubchemFP557 0.705012159701744

GraphFP519 0.704607977084704

PubchemFP397 0.70402202966051

GraphFP620 0.703816619587997

GraphFP273 0.702718110017096

GraphFP613 0.702383207975263

GraphFP895 0.702305690055032

MACCSFP129 0.701747228040279

GraphFP910 0.701147979331416

GraphFP133 0.701131794077175

FP909 0.70057268907357

FP462 0.698304451194533

FP679 0.698086648653565

GraphFP614 0.696647247524912

GraphFP833 0.696370649479051

FP640 0.693969282967455

FP810 0.693001457100148

GraphFP278 0.692476057978697

FP132 0.691732668275749

GraphFP793 0.691492286071799

PubchemFP665 0.690398678908875

GraphFP758 0.690158859186601

AD2D572 0.689649224775013

APC2D4_X_X 0.689587938382783

FP102 0.689321713097776

FP521 0.688890212963464

KRFP3881 0.688832296874437

AD2D494 0.688722138114282

GraphFP79 0.687478488249248

FP776 0.687468827853238

AD2D537 0.687415414358579

GraphFP144 0.687272921030141

GraphFP778 0.686848197674119

KRFP2781 0.686651749104388

FP613 0.685946999174613

KRFP4163 0.685803436826324

KRFP352 0.684679716207157

PubchemFP193 0.684590627572413

KRFP2055 0.683544047958333

FP522 0.683258189023063

FP937 0.683199532424675

GraphFP781 0.683155728924782

FP315 0.683066356420737

FP585 0.681960194301144

GraphFP496 0.681856478611428

FP946 0.68161982436867

AD2D316 0.681518747000935

GraphFP675 0.681107843813993

GraphFP960 0.681002399355643

PubchemFP184 0.680077813582953

FP781 0.68003714440238

KRFP487 0.67930929759866

FP425 0.679184866754364

PubchemFP713 0.678970457916441

SubFP239 0.678851465915168

AD2D234 0.678562751223064

AD2D5 0.678022830664505

GraphFP986 0.677791001370352

KRFP4285 0.677774676127581

FP525 0.677415143764471

FP368 0.677306598096578

FP758 0.677202303867923

PubchemFP361 0.677111245076846

FP282 0.677021500933937

FP723 0.676683846308654

GraphFP46 0.676366095712276

AD2D184 0.676307170618166

KRFP4823 0.675627315059731

GraphFP556 0.675075068926236

APC2D8_C_S 0.674640268808263

FP892 0.67422905357163

KRFP2564 0.674222085591273

FP526 0.673098673439923

GraphFP848 0.672924138490584

FP35 0.672525479638116

GraphFP673 0.671132476471398

PubchemFP655 0.67083159485007

FP941 0.669660464077773

FP535 0.669377206586825

GraphFP509 0.668524162217494

FP241 0.668013248182721

FP608 0.667495414605433

PubchemFP538 0.66600165535975

SubFP184 0.665933717207166

PubchemFP367 0.665647705154365

KRFP3869 0.665607684168618

KRFP1153 0.664868963386256

AD2D25 0.664180192202117

GraphFP423 0.663823785208623

FP561 0.663807008218686

APC2D5_N_N 0.661562110067273

FP25 0.661256564583912

PubchemFP541 0.660895155267832

GraphFP166 0.659591871109882

SubFPC169 0.659537665339265

FP898 0.659475189032133

AD2D82 0.659261106677303

GraphFP528 0.659073416678436

FP532 0.658988805260332

FP887 0.658962949801907

KRFP349 0.658463468423966

FP870 0.657598964881825

GraphFP482 0.657264371155937

FP473 0.656834053053794

GraphFP831 0.656422349662657

GraphFP191 0.655673313766638

GraphFP101 0.654724041693871

KRFP3727 0.654508939531697

PubchemFP602 0.654356054942792

GraphFP545 0.654216541904259

GraphFP505 0.653693017033551

KRFP1452 0.653207494877658

FP540 0.652527866327188

PubchemFP530 0.651889612989898

APC2D2_P_P 0.651690702339482

FP570 0.651159711252197

FP973 0.650486584230798

PubchemFP23 0.650192049901779

GraphFP832 0.649980377515334

PubchemFP577 0.648804225080686

KRFP25 0.648294464234458

FP562 0.647762862418912

FP509 0.647707074838687

FP595 0.647637598263062

FP454 0.647397633356583

GraphFP963 0.647318501888869

KRFP3674 0.646306756473128

MACCSFP25 0.644667918713844

FP828 0.644638440106662

GraphFP601 0.644596133044521

FP111 0.64436132552707

FP520 0.641164966885607

FP983 0.64098208614333

FP349 0.640902660921289

FP278 0.640741768837036

KRFP3034 0.640734830990782

FP578 0.640028957447538

FP83 0.639721274844259

APC2D2_Cl_X 0.63887656499994

GraphFP718 0.638876511787836

APC2D3_C_Br 0.638871063081749

GraphFP64 0.638866929403252

GraphFP562 0.6388660252547

KRFP769 0.638854412808477

GraphFP240 0.638853967945614

GraphFP593 0.638851796447841

APC2D9_N_N 0.638842095432179

AD2D279 0.638811266060569

KRFP133 0.638625920611598

SubFPC179 0.638469420698119

KRFP3013 0.638394670107471

GraphFP98 0.63789083873524

APC2D1_C_P 0.637882567428316

KRFP665 0.636985557786348

FP211 0.636604570019781

GraphFP401 0.635641726163728

APC2D3_O_Cl 0.635641673431108

KRFP3759 0.635641672649251

KRFP821 0.635641515747892

GraphFP612 0.635641501729935

KRFP76 0.635641254648628

GraphFP389 0.63564083443486

KRFP3452 0.635640352467069

APC2D1_O_X 0.635640340971787

PubchemFP44 0.635639749687334

GraphFP962 0.635639060395117

AD2D292 0.635638235690849

KRFP3074 0.635637377270485

PubchemFP348 0.635637290186834

GraphFP811 0.635636208512255

GraphFP665 0.635635044256693

AD2D328 0.635635010707953

GraphFP383 0.6356339757586

GraphFP75 0.635632315350311

GraphFP688 0.635631365515605

SubFPC215 0.635629526079235

GraphFP1006 0.635627947861721

PubchemFP826 0.635627482819558

PubchemFP845 0.635627159104344

GraphFP440 0.635626030039684

GraphFP976 0.635621454941019

KRFP367 0.635620197960574

APC2D7_P_P 0.63562016248121

GraphFP999 0.63562001968307

GraphFP590 0.63561832006325

FP216 0.635617932733685

FP693 0.635617814658991

AD2D329 0.635616837590091

GraphFP661 0.63561118041652

GraphFP995 0.635605104060087

APC2D4_P_X 0.635604553637705

KRFP2587 0.635600998319546

GraphFP872 0.635595901724096

GraphFP572 0.63559458380683

SubFPC190 0.635581092216924

KRFP4086 0.635575311979376

PubchemFP543 0.63555163963523

KRFP468 0.635543927605389

AD2D257 0.635540320269972

KRFP4188 0.635528982747802

APC2D3_O_F 0.635527866783072

KRFP1155 0.635519773125544

KRFP3453 0.635499799943085

KRFP4524 0.635458942053994

GraphFP904 0.635371414840853

KRFP4830 0.635274723084318

PubchemFP151 0.634958003986683

SubFP151 0.634941924442722

GraphFP162 0.634174186433705

FP590 0.633872754492284

FP1018 0.632623252778944

KRFP3137 0.631522724400021

PubchemFP180 0.630990077712883

PubchemFP615 0.630766791714977

GraphFP524 0.630667762831382

FP308 0.630654147039093

GraphFP980 0.629672090058379

GraphFP380 0.627631183110733

APC2D10_C_S 0.627454388970237

FP404 0.627443964594432

PubchemFP155 0.626954967910041

PubchemFP597 0.626622531984979

GraphFP649 0.626451349653563

GraphFP194 0.626067727605206

GraphFP515 0.62440609264135

KRFP3375 0.624343501082464

FP750 0.623941100577918

APC2D6_O_X 0.623627764853349

PubchemFP450 0.622890690493624

GraphFP774 0.622811814848418

PubchemFP459 0.622264719989968

GraphFP451 0.621690344210981

FP984 0.621372814215568

KRFP4301 0.621300543120377

FP722 0.620459870853516

FP914 0.620123627755557

GraphFP421 0.619882182355858

FP287 0.619059441882563

PubchemFP493 0.618230347262966

FP1010 0.6180281821507

FP458 0.616788977656628

PubchemFP598 0.616185323353706

GraphFP434 0.616063140527654

KRFP3440 0.615936103838784

GraphFP777 0.615920171275374

KRFP4472 0.615306202315287

GraphFP77 0.614526551542288

PubchemFP59 0.614394076681917

GraphFP447 0.612715297171742

FP854 0.612061809972026

APC2D2_C_Cl 0.611822025022419

GraphFP516 0.611810610492522

FP825 0.611758151713873

GraphFP836 0.611329836864781

SubFPC184 0.611190264305926

FP742 0.611182893870656

GraphFP280 0.610613375648257

KRFP3704 0.610060672754671

GraphFP975 0.609872995781103

GraphFP764 0.609772089369906

FP242 0.609679541284095

GraphFP857 0.609646966365302

PubchemFP485 0.609479926913721

GraphFP317 0.609462585634252

GraphFP325 0.608776833148424

GraphFP272 0.608741108571814

PubchemFP169 0.607328811834796

FP432 0.606854585835451

PubchemFP145 0.6065065094207

GraphFP651 0.60641495333283

FP547 0.606300117667713

PubchemFP342 0.6062446218549

GraphFP502 0.606155682902995

KRFP3223 0.605967238083011

FP706 0.605759538402234

EStateFP23 0.605608528052733

PubchemFP385 0.605483010937455

FP224 0.605460847826133

FP199 0.605159600891994

GraphFP342 0.605147322708541

GraphFP479 0.60510425216248

GraphFP71 0.605063385435214

APC2D2_C_Br 0.60489144938663

SubFP171 0.604856853658864

PubchemFP779 0.604661057340302

GraphFP663 0.604611664000408

GraphFP739 0.604541477371683

GraphFP603 0.604518145256078

APC2D8_O_F 0.604067823466235

APC2D1_O_S 0.604064965040356

APC2D9_O_X 0.604039518204496

KRFP1173 0.604007150123797

GraphFP1014 0.603886193466868

GraphFP304 0.603884769159314

APC2D6_C_S 0.603849322670988

PubchemFP812 0.603820963168697

PubchemFP259 0.603818330135825

APC2D3_N_S 0.603684422538362

APC2D2_F_X 0.603657287979706

SubFP153 0.603287563607273

FP560 0.603282445461899

KRFP3936 0.603220336183156

GraphFP130 0.603184942274946

MACCSFP64 0.603184750327028

GraphFP557 0.603156182310954

KRFP4231 0.603088442346187

MACCSFP23 0.603022689155527

FP382 0.602966681251562

AD2D96 0.602824750181096

KRFP1165 0.602759246669152

APC2D4_I_I 0.602757070917741

PubchemFP56 0.602748924660443

KRFP4191 0.602559621016634

PubchemFP378 0.602526599482674

PubchemFP195 0.602475554758425

PubchemFP782 0.602356569687614

PubchemFP575 0.602356569687614

GraphFP185 0.602348753168411

FP251 0.602284328256563

PubchemFP737 0.602146296938745

APC2D1_O_Cl 0.602019391394583

APC2D8_P_P 0.602018556121475

APC2D8_C_F 0.602017719456525

GraphFP471 0.601965876752721

GraphFP301 0.601958885990371

AD2D394 0.60190870043367

GraphFP405 0.601883175608579

GraphFP168 0.601773311492854

GraphFP54 0.601641369845362

KRFP1299 0.601631125283301

GraphFP599 0.601624210745491

GraphFP274 0.601559089445031

FP138 0.601510464732796

KRFP1633 0.601232236036531

KRFP1021 0.60094827646037

GraphFP632 0.600451126474571

AD2D640 0.600377282317157

AD2D171 0.599798567483226

FP948 0.599756948739487

KRFP1184 0.59963688519551

KRFP670 0.598255814219159

GraphFP178 0.597958496939197

GraphFP417 0.596052226630123

KRFP325 0.594147245159301

FP955 0.593622648679986

FP493 0.593585996321513

FP820 0.593213231174564

FP910 0.593108154484468

GraphFP293 0.593037135968377

FP938 0.591807925658753

KRFP4813 0.589816381225946

GraphFP1024 0.589680941613773

FP683 0.58915768739328

KRFP3054 0.589057775852091

APC2D4_O_X 0.588994964768447

KRFP307 0.58749780350348

GraphFP40 0.586788680173225

KRFP2984 0.586641188214143

KRFP3427 0.586306329961286

GraphFP997 0.585129661254613

KRFP91 0.584980372079905

GraphFP799 0.584898364498075

FP833 0.582961623255022

GraphFP213 0.582130508413768

APC2D2_C_S 0.581860136828083

GraphFP604 0.58169851439181

FP413 0.580322766429545

GraphFP202 0.57957913632013

PubchemFP650 0.578243703650292

GraphFP826 0.578148503661218

MACCSFP41 0.576698013543919

FP101 0.576247880425414

KRFP3390 0.575443289810861

FP424 0.574780665388674

FP189 0.574492273329796

GraphFP721 0.57436866554704

MACCSFP34 0.574148183460405

GraphFP1017 0.573627871582172

FP31 0.573413374976734

KRFP4803 0.571486783232567

KRFP3945 0.57134009892443

GraphFP954 0.570484332810653

GraphFP615 0.570395398972468

GraphFP409 0.57004564971924

AD2D251 0.569942456865085

AD2D35 0.569796334207345

GraphFP1022 0.569667193085643

SubFPC239 0.56916715277563

KRFP4856 0.568891467863923

KRFP4197 0.568747524269895

KRFP2976 0.568605294772157

AD2D711 0.568270296910667

KRFP3393 0.568051770534482

FP478 0.568014670623563

SubFP10 0.567907879716013

GraphFP705 0.567831036306497

AD2D213 0.567635967837502

GraphFP820 0.567465023275316

GraphFP437 0.567343230028452

GraphFP565 0.566923349334241

SubFP8 0.566720886406475

KRFP2870 0.566694674425297

GraphFP226 0.566045229984602

GraphFP533 0.566008533708799

SubFP17 0.565316390386385

FP770 0.56524104677793

GraphFP901 0.56519378125288

GraphFP579 0.564066469504767

AD2D498 0.563855260103868

FP542 0.563449063951648

GraphFP819 0.56330760151833

FP116 0.563112110169113

GraphFP377 0.562974514245744

GraphFP694 0.562394858081037

EStateFP29 0.562255817995343

FP33 0.562125452085294

AD2D715 0.561952042763296

PubchemFP574 0.561201613654589

FP718 0.560602124908508

FP882 0.560440071351552

FP489 0.560300821218913

FP730 0.558006016552296

FP712 0.556030694345414

FP294 0.555978183495818

GraphFP62 0.555789681122647

KRFP758 0.554967029681083

FP716 0.554532945978861

APC2D7_N_N 0.554112186312912

GraphFP981 0.55370681939317

GraphFP214 0.553417648605165

PubchemFP412 0.552336480990019

GraphFP9 0.552177772470356

FP786 0.551111988852796

FP163 0.550981875092315

GraphFP58 0.549621999418393

FP792 0.549173100119836

FP437 0.548835577069253

KRFP2772 0.548818960934988

GraphFP51 0.547740404178719

FP104 0.547453887990184

GraphFP982 0.547307488064779

GraphFP691 0.54702656955238

AD2D562 0.546907153551955

GraphFP408 0.546646300754475

AD2D636 0.546206205152927

APC2D3_Cl_X 0.545077103842302

GraphFP501 0.545072896116935

GraphFP227 0.544691165267892

SubFP86 0.543712656474116

PubchemFP625 0.543077275404728

KRFP3646 0.542892876993171

PubchemFP824 0.541658364768508

FP19 0.539984315001513

GraphFP800 0.539899817598654

FP696 0.539444051735664

GraphFP755 0.538762675442017

MACCSFP62 0.538604180473036

FP246 0.538517792968397

AD2D337 0.538354466582311

KRFP3380 0.538165428755756

FP695 0.538018072864693

FP271 0.537510015802872

GraphFP607 0.535285725768713

FP281 0.53483428543828

FP164 0.533950679683911

FP336 0.533381913712955

GraphFP900 0.532581002699061

FP348 0.532105553308057

FP218 0.531781913780065

GraphFP297 0.531714393293552

KRFP303 0.531607567092098

GraphFP616 0.531542478674724

GraphFP741 0.531300776628039

KRFP67 0.530829320369467

GraphFP750 0.530410396369206

GraphFP808 0.530353186378968

PubchemFP196 0.530087737827495

GraphFP666 0.529993412789255

KRFP1161 0.529627541778139

GraphFP299 0.528551565664784

GraphFP551 0.528409135339954

FP739 0.526818205037734

KRFP3598 0.526377265748347

PubchemFP705 0.526135478703398

PubchemFP647 0.526109039655233

GraphFP41 0.521610261599129

FP187 0.521547022243415

AD2D141 0.521181296495941

FP900 0.521056102660401

GraphFP812 0.520953896478734

KRFP3887 0.520709662302595

GraphFP492 0.519372377883689

EStateFP18 0.519326303014651

FP1011 0.518978831074309

KRFP1408 0.518093795373388

KRFP1645 0.517605635159753

PubchemFP800 0.51744138143812

GraphFP690 0.517234167730927

GraphFP3 0.516744393417822

PubchemFP414 0.516265787291978

KRFP3225 0.515996215179599

FP399 0.515722547795356

GraphFP212 0.515043751782177

APC2D10_C_X 0.514859895989179

SubFPC74 0.514858041386316

KRFP1432 0.514764243686246

GraphFP497 0.514305721003353

GraphFP696 0.514263691133512

FP47 0.512530766729871

FP152 0.512150053916915

GraphFP670 0.5117947080481

FP981 0.511726028144903

GraphFP921 0.51060013902662

PubchemFP563 0.510359703799194

GraphFP645 0.509882969305118

KRFP4018 0.5098425664684

FP191 0.509819235107634

FP267 0.509359222230158

GraphFP210 0.509080839964788

GraphFP570 0.508574185788717

GraphFP656 0.506979256546244

SubFPC18 0.506650221963642

KRFP2977 0.506623835355767

GraphFP546 0.506384874086696

KRFP3441 0.5059365497212

FP122 0.505130252000069

GraphFP581 0.504732160331882

GraphFP253 0.50415627161486

GraphFP863 0.503179256374432

GraphFP219 0.503077903773205

FP65 0.502635311823618

AD2D86 0.502568589737955

FP443 0.501528103173054

GraphFP783 0.501264849898609

PubchemFP673 0.501141795418527

AD2D648 0.500726921999314

FP314 0.500515451683688

FP924 0.499144097086129

FP387 0.497835319885772

GraphFP853 0.496100435964685

KRFP3443 0.492556594243154

KRFP4284 0.490028699592856

GraphFP198 0.489356548513043

FP999 0.488400091998117

PubchemFP662 0.487847729587548

KRFP10 0.486954001778474

FP809 0.486723626131317

PubchemFP693 0.486674733617075

FP516 0.486562507110153

GraphFP647 0.486310830167445

FP402 0.485650656020864

PubchemFP784 0.485608259604074

FP338 0.485568768905136

FP333 0.480510961394273

FP790 0.480506837875565

FP903 0.480283627064795

PubchemFP379 0.480221896894439

GraphFP291 0.47958681498223

SubFP74 0.479535059814101

GraphFP25 0.479399551093406

FP94 0.479372884584379

FP2 0.478394794770659

GraphFP971 0.477287110179547

GraphFP605 0.475444492817621

PubchemFP448 0.474499481753739

GraphFP804 0.474338896808146

KRFP4291 0.473117847155993

EStateFP8 0.471603216701541

GraphFP827 0.469383434693591

FP872 0.468762760182898

GraphFP738 0.467844813791073

KRFP1407 0.467591857464328

PubchemFP504 0.465734205770462

KRFP3779 0.462419816995135

GraphFP282 0.462368066833628

FP858 0.461054550063504

KRFP19 0.46061058315508

FP386 0.459441292305316

GraphFP871 0.458284247870317

PubchemFP758 0.458187124376526

AD2D321 0.45791334518163

KRFP4516 0.457582528715217

GraphFP220 0.455781340489933

GraphFP931 0.455244533717

KRFP3160 0.455031793669804

GraphFP703 0.45499714881316

GraphFP235 0.454724670181622

GraphFP193 0.454217632257216

KRFP3435 0.453925454254568

KRFP1922 0.453752814382467

KRFP3151 0.453511108629981

GraphFP13 0.452628603038803

GraphFP896 0.451840837102601

FP808 0.451772891360095

SubFP109 0.45147114749609

PubchemFP258 0.451036150441223

APC2D9_C_X 0.45101153580132

FP852 0.45053614348322

GraphFP850 0.449549053261561

SubFPC53 0.449466574975495

SubFPC294 0.449466574975495

SubFPC288 0.449466574975495

SubFPC19 0.449466574975495

SubFPC183 0.449466574975495

SubFPC163 0.449466574975495

SubFPC136 0.449466574975495

SubFPC134 0.449466574975495

SubFPC128 0.449466574975495

SubFPC11 0.449466574975495

SubFP72 0.449466574975495

SubFP68 0.449466574975495

SubFP22 0.449466574975495

SubFP210 0.449466574975495

SubFP204 0.449466574975495

SubFP18 0.449466574975495

SubFP180 0.449466574975495

SubFP136 0.449466574975495

SubFP134 0.449466574975495

SubFP128 0.449466574975495

SubFP125 0.449466574975495

SubFP101 0.449466574975495

PubchemFP97 0.449466574975495

PubchemFP95 0.449466574975495

PubchemFP878 0.449466574975495

PubchemFP864 0.449466574975495

PubchemFP857 0.449466574975495

PubchemFP833 0.449466574975495

PubchemFP815 0.449466574975495

PubchemFP80 0.449466574975495

PubchemFP792 0.449466574975495

PubchemFP78 0.449466574975495

PubchemFP773 0.449466574975495

PubchemFP738 0.449466574975495

PubchemFP675 0.449466574975495

PubchemFP669 0.449466574975495

PubchemFP658 0.449466574975495

PubchemFP62 0.449466574975495

PubchemFP61 0.449466574975495

PubchemFP551 0.449466574975495

PubchemFP532 0.449466574975495

PubchemFP517 0.449466574975495

PubchemFP514 0.449466574975495

PubchemFP497 0.449466574975495

PubchemFP48 0.449466574975495

PubchemFP474 0.449466574975495

PubchemFP467 0.449466574975495

PubchemFP466 0.449466574975495

PubchemFP460 0.449466574975495

PubchemFP428 0.449466574975495

PubchemFP39 0.449466574975495

PubchemFP363 0.449466574975495

PubchemFP360 0.449466574975495

PubchemFP359 0.449466574975495

PubchemFP35 0.449466574975495

PubchemFP328 0.449466574975495

PubchemFP305 0.449466574975495

PubchemFP245 0.449466574975495

PubchemFP232 0.449466574975495

PubchemFP219 0.449466574975495

PubchemFP202 0.449466574975495

PubchemFP200 0.449466574975495

PubchemFP188 0.449466574975495

PubchemFP17 0.449466574975495

PubchemFP160 0.449466574975495

PubchemFP132 0.449466574975495

MACCSFP28 0.449466574975495

MACCSFP21 0.449466574975495

KRFP973 0.449466574975495

KRFP936 0.449466574975495

KRFP797 0.449466574975495

KRFP787 0.449466574975495

KRFP778 0.449466574975495

KRFP682 0.449466574975495

KRFP662 0.449466574975495

KRFP658 0.449466574975495

KRFP653 0.449466574975495

KRFP630 0.449466574975495

KRFP607 0.449466574975495

KRFP562 0.449466574975495

KRFP554 0.449466574975495

KRFP483 0.449466574975495

KRFP4827 0.449466574975495

KRFP4774 0.449466574975495

KRFP4767 0.449466574975495

KRFP4763 0.449466574975495

KRFP4689 0.449466574975495

KRFP4657 0.449466574975495

KRFP453 0.449466574975495

KRFP45 0.449466574975495

KRFP4413 0.449466574975495

KRFP4359 0.449466574975495

KRFP4317 0.449466574975495

KRFP4315 0.449466574975495

KRFP4281 0.449466574975495

KRFP4264 0.449466574975495

KRFP4254 0.449466574975495

KRFP4238 0.449466574975495

KRFP4192 0.449466574975495

KRFP4116 0.449466574975495

KRFP4053 0.449466574975495

KRFP4023 0.449466574975495

KRFP3967 0.449466574975495

KRFP3950 0.449466574975495

KRFP3949 0.449466574975495

KRFP3934 0.449466574975495

KRFP3899 0.449466574975495

KRFP3895 0.449466574975495

KRFP3813 0.449466574975495

KRFP3803 0.449466574975495

KRFP3794 0.449466574975495

KRFP3793 0.449466574975495

KRFP3778 0.449466574975495

KRFP3767 0.449466574975495

KRFP3656 0.449466574975495

KRFP3617 0.449466574975495

KRFP359 0.449466574975495

KRFP3529 0.449466574975495

KRFP3418 0.449466574975495

KRFP3403 0.449466574975495

KRFP3385 0.449466574975495

KRFP3373 0.449466574975495

KRFP3368 0.449466574975495

KRFP3179 0.449466574975495

KRFP3159 0.449466574975495

KRFP315 0.449466574975495

KRFP3104 0.449466574975495

KRFP3015 0.449466574975495

KRFP3012 0.449466574975495

KRFP2886 0.449466574975495

KRFP2816 0.449466574975495

KRFP2683 0.449466574975495

KRFP2668 0.449466574975495

KRFP2263 0.449466574975495

KRFP1933 0.449466574975495

KRFP1920 0.449466574975495

KRFP190 0.449466574975495

KRFP1824 0.449466574975495

KRFP1592 0.449466574975495

KRFP1536 0.449466574975495

KRFP1500 0.449466574975495

KRFP1240 0.449466574975495

KRFP1013 0.449466574975495

GraphFP992 0.449466574975495

GraphFP989 0.449466574975495

GraphFP969 0.449466574975495

GraphFP956 0.449466574975495

GraphFP927 0.449466574975495

GraphFP924 0.449466574975495

GraphFP907 0.449466574975495

GraphFP88 0.449466574975495

GraphFP865 0.449466574975495

GraphFP844 0.449466574975495

GraphFP810 0.449466574975495

GraphFP806 0.449466574975495

GraphFP805 0.449466574975495

GraphFP80 0.449466574975495

GraphFP782 0.449466574975495

GraphFP766 0.449466574975495

GraphFP759 0.449466574975495

GraphFP753 0.449466574975495

GraphFP749 0.449466574975495

GraphFP724 0.449466574975495

GraphFP719 0.449466574975495

GraphFP692 0.449466574975495

GraphFP69 0.449466574975495

GraphFP687 0.449466574975495

GraphFP664 0.449466574975495

GraphFP653 0.449466574975495

GraphFP644 0.449466574975495

GraphFP622 0.449466574975495

GraphFP611 0.449466574975495

GraphFP595 0.449466574975495

GraphFP589 0.449466574975495

GraphFP584 0.449466574975495

GraphFP560 0.449466574975495

GraphFP539 0.449466574975495

GraphFP525 0.449466574975495

GraphFP490 0.449466574975495

GraphFP488 0.449466574975495

GraphFP463 0.449466574975495

GraphFP461 0.449466574975495

GraphFP460 0.449466574975495

GraphFP457 0.449466574975495

GraphFP454 0.449466574975495

GraphFP438 0.449466574975495

GraphFP430 0.449466574975495

GraphFP415 0.449466574975495

GraphFP398 0.449466574975495

GraphFP375 0.449466574975495

GraphFP361 0.449466574975495

GraphFP352 0.449466574975495

GraphFP33 0.449466574975495

GraphFP322 0.449466574975495

GraphFP313 0.449466574975495

GraphFP266 0.449466574975495

GraphFP260 0.449466574975495

GraphFP258 0.449466574975495

GraphFP257 0.449466574975495

GraphFP246 0.449466574975495

GraphFP22 0.449466574975495

GraphFP172 0.449466574975495

GraphFP142 0.449466574975495

GraphFP139 0.449466574975495

GraphFP113 0.449466574975495

GraphFP110 0.449466574975495

GraphFP1 0.449466574975495

GraphFP1020 0.449466574975495

GraphFP1015 0.449466574975495

GraphFP1011 0.449466574975495

EStateFP64 0.449466574975495

EStateFP62 0.449466574975495

EStateFP37 0.449466574975495

APC2D9_N_S 0.449466574975495

APC2D9_N_P 0.449466574975495

APC2D9_C_I 0.449466574975495

APC2D8_O_Cl 0.449466574975495

APC2D8_Cl_X 0.449466574975495

APC2D8_C_I 0.449466574975495

APC2D7_S_P 0.449466574975495

APC2D7_N_P 0.449466574975495

APC2D7_I_X 0.449466574975495

APC2D6_N_X 0.449466574975495

APC2D5_O_Cl 0.449466574975495

APC2D5_O_Br 0.449466574975495

APC2D5_N_P 0.449466574975495

APC2D5_N_Cl 0.449466574975495

APC2D4_O_Br 0.449466574975495

APC2D4_N_F 0.449466574975495

APC2D4_Cl_Cl 0.449466574975495

APC2D4_C_Br 0.449466574975495

APC2D4_Br_X 0.449466574975495

APC2D3_O_S 0.449466574975495

APC2D3_O_Br 0.449466574975495

APC2D3_N_X 0.449466574975495

APC2D2_O_X 0.449466574975495

APC2D2_N_Cl 0.449466574975495

APC2D2_F_F 0.449466574975495

APC2D1_S_P 0.449466574975495

APC2D1_N_P 0.449466574975495

APC2D10_N_O 0.449466574975495

AD2D87 0.449466574975495

AD2D702 0.449466574975495

AD2D649 0.449466574975495

AD2D624 0.449466574975495

AD2D573 0.449466574975495

AD2D571 0.449466574975495

AD2D531 0.449466574975495

AD2D503 0.449466574975495

AD2D491 0.449466574975495

AD2D483 0.449466574975495

AD2D481 0.449466574975495

AD2D468 0.449466574975495

AD2D418 0.449466574975495

AD2D413 0.449466574975495

AD2D390 0.449466574975495

AD2D330 0.449466574975495

AD2D297 0.449466574975495

AD2D284 0.449466574975495

AD2D28 0.449466574975495

AD2D268 0.449466574975495

AD2D264 0.449466574975495

AD2D174 0.449466574975495

AD2D146 0.449466574975495

AD2D111 0.449466574975495

AD2D104 0.449466574975495

PubchemFP503 0.449156315628442

KRFP1427 0.448780892337292

GraphFP789 0.448703598574311

APC2D6_P_P 0.448372013463036

GraphFP45 0.447885318806772

GraphFP592 0.44700989422282

GraphFP680 0.44688954977657

FP233 0.446653347170894

PubchemFP805 0.446067753967569

APC2D7_X_X 0.445390607569829

GraphFP935 0.44483103131335

GraphFP330 0.444550671441824

KRFP3430 0.44450468777183

KRFP435 0.444047293244415

PubchemFP382 0.443901613788045

FP410 0.443696231289873

SubFP292 0.441953515709978

GraphFP103 0.441123608253791

FP840 0.440412410261579

KRFP4139 0.440091699688713

GraphFP265 0.439405208668081

MACCSFP17 0.439340923296503

FP826 0.438789859887212

PubchemFP162 0.437497310928685

FP698 0.436595265697701

PubchemFP606 0.436394277626471

PubchemFP419 0.436078052378409

PubchemFP565 0.43586139740901

GraphFP124 0.435614099911579

FP844 0.434597972449885

GraphFP985 0.432317718636425

KRFP3784 0.431364835764412

SubFP52 0.431138826931396

KRFP4496 0.429077771314137

GraphFP223 0.428561898030773

GraphFP78 0.422756540827869

KRFP3703 0.422225177328393

GraphFP233 0.419200896931325

KRFP3933 0.417679527127587

GraphFP788 0.417624681284924

PubchemFP164 0.416599855563975

GraphFP345 0.415883702463859

GraphFP36 0.41566497628813

GraphFP934 0.415271541640108

AD2D493 0.414019966022384

PubchemFP661 0.413811311998025

PubchemFP657 0.413694763706885

FP876 0.413600661633242

GraphFP148 0.413433882698308

PubchemFP623 0.41154270978134

GraphFP290 0.410751162899119

GraphFP249 0.41068603922084

GraphFP860 0.41058844671175

PubchemFP170 0.41058165933928

KRFP3940 0.410096010724284

GraphFP801 0.409028294951477

GraphFP978 0.408884513409611

PubchemFP628 0.407867769910559

AD2D637 0.404839103629539

GraphFP292 0.404064401970485

PubchemFP150 0.403941579720542

FP288 0.403591505591153

KRFP387 0.403494559924033

FP343 0.403188467342314

FP894 0.403073332025438

GraphFP583 0.402983800686977

APC2D1_O_O 0.400888318904435

AD2D550 0.399202143209518

KRFP413 0.397468365328038

PubchemFP626 0.397230240641119

AD2D716 0.394631669472316

FP36 0.394482627785005

PubchemFP632 0.390969234835827

FP1012 0.389599533144052

FP985 0.388749722295099

GraphFP818 0.382027390616771

FP811 0.381152293606199

FP736 0.380245612913017

GraphFP400 0.379287038311065

FP244 0.37885333507215

AD2D250 0.378020787360175

GraphFP52 0.377748166169166

GraphFP160 0.376966769871656

KRFP3907 0.376446973611297

GraphFP420 0.374815263158859

GraphFP558 0.373763222128551

AD2D249 0.373741974064403

SubFPC276 0.373405798311541

FP625 0.372553160260139

PubchemFP588 0.372281842626606

GraphFP42 0.371971951857566

PubchemFP34 0.371837017929045

GraphFP336 0.371809461631231

PubchemFP706 0.371772990452744

KRFP2675 0.371741796288791

PubchemFP707 0.371728311901316

KRFP370 0.371254284489389

SubFPC37 0.370624066017574

GraphFP449 0.370623501265303

KRFP2135 0.370577148220768

APC2D4_Cl_X 0.368656713378912

PubchemFP157 0.36841534764769

GraphFP729 0.367813487545054

FP774 0.366303906771767

KRFP403 0.366141106575515

FP290 0.366128692582634

KRFP3180 0.36599108005834

GraphFP1001 0.365258899026471

AD2D101 0.364310547312918

GraphFP239 0.364116655118746

FP460 0.362863060660315

KRFP3712 0.362461667738873

PubchemFP292 0.36208953626396

FP551 0.361376157166933

GraphFP630 0.360059923959072

AD2D484 0.357434538087083

KRFP4512 0.357313539178187

GraphFP686 0.355388823916703

FP922 0.35250340066012

GraphFP845 0.351838287935998

GraphFP109 0.34761079110848

FP849 0.345860844371018

FP504 0.345320028477272

FP895 0.345245560762631

KRFP1566 0.343471472481295

FP816 0.342316495120314

AD2D570 0.337445895084161

SubFPC96 0.335197500920497

GraphFP763 0.33304094211795

PubchemFP149 0.32876875311588

FP848 0.328683780656126

KRFP358 0.328571110111413

FP127 0.326053487232939

GraphFP704 0.326021560146346

SubFPC151 0.325887413654609

KRFP3560 0.323267813026405

GraphFP767 0.32196770190259

FP380 0.320767254308055

AD2D277 0.319357013319147

GraphFP503 0.319272268537675

FP863 0.319131734098451

FP871 0.317285323373949

FP592 0.316605709230041

AD2D238 0.31528188083941

KRFP4820 0.31363408128764

GraphFP541 0.313618220654928

KRFP3291 0.312489831373721

SubFP15 0.30916826883078

KRFP4000 0.308674154997698

GraphFP882 0.308082645604917

AD2D559 0.306697923870167

FP554 0.305595824813146

GraphFP284 0.30511288593684

FP775 0.301437677079056

FP303 0.298637380969623

GraphFP305 0.295710821650276

FP541 0.294351194571072

FP98 0.293732336941817

GraphFP374 0.292931697448153

FP813 0.290504636022097

GraphFP769 0.287784621349502

APC2D7_C_Cl 0.286933639151062

GraphFP571 0.286919438535112

GraphFP568 0.285940186081063

FP208 0.28588094857899

FP293 0.285473653443363

MACCSFP37 0.285257974419152

GraphFP327 0.285085361313882

EStateFP70 0.283909426513153

FP650 0.279677427048084

GraphFP586 0.27868997026611

APC2D3_O_I 0.277919550632975

GraphFP91 0.273355226304772

GraphFP824 0.270923326257249

SubFP133 0.270900528366601

FP110 0.270848296073264

GraphFP974 0.27082278072014

GraphFP209 0.270605233166379

KRFP4754 0.269948549207577

FP669 0.269508671933882

FP1019 0.269476183692093

KRFP790 0.268259348015845

GraphFP199 0.267743035496949

EStateFP10 0.267509584852531

EStateFP49 0.266945395296613

GraphFP748 0.266285601573339

KRFP3745 0.26584431401989

FP666 0.264556323531767

GraphFP7 0.2640133137058

KRFP447 0.263385117015754

FP1024 0.263019329161675

GraphFP196 0.262956527901337

PubchemFP703 0.26249074996721

KRFP4022 0.262355708913712

KRFP3353 0.262161583189842

GraphFP547 0.261946204786332

GraphFP43 0.260748937222462

GraphFP76 0.260642415800973

GraphFP427 0.260493025623486

FP280 0.260253519661415

GraphFP234 0.259503806346036

AD2D303 0.259211074745177

FP637 0.259140070625239

KRFP374 0.258641394649372

PubchemFP533 0.258036715654116

GraphFP988 0.258007076236334

FP715 0.257438850171184

GraphFP262 0.257363839955532

AD2D553 0.257090707155228

FP269 0.256969865443774

KRFP3284 0.256069148294108

FP829 0.255932140178973

FP769 0.255100263030795

AD2D404 0.253881873670484

FP869 0.253193281825263

GraphFP897 0.253027188311087

MACCSFP14 0.251060751093784

GraphFP561 0.250221343640008

KRFP3816 0.249796908837367

GraphFP11 0.249340817642486

FP135 0.248979504755204

KRFP33 0.248935989532104

FP586 0.248220639721187

SubFP293 0.247339554183892

GraphFP12 0.244115503542253

KRFP442 0.243833855719525

GraphFP38 0.243232621631343

PubchemFP581 0.243221015761783

GraphFP276 0.241624011801745

APC2D5_N_S 0.240594043714121

SubFP63 0.239417671043557

PubchemFP461 0.237921311407149

KRFP3078 0.237091318945959

KRFP2667 0.235872014855873

FP398 0.235798292075665

KRFP72 0.234507263687696

GraphFP977 0.232965835763769

KRFP1250 0.232889992255793

FP155 0.232849484500508

FP1020 0.231083992746858

KRFP3675 0.228783018747265

GraphFP93 0.221143375298147

KRFP3893 0.220099977884389

GraphFP567 0.219757153442862

FP778 0.218963450687006

KRFP4519 0.218719658723378

GraphFP432 0.216810714824235

KRFP4521 0.21561218557156

AD2D135 0.212367809697589

PubchemFP755 0.211998575387482

FP853 0.210170767652441

KRFP458 0.208176384906525

KRFP92 0.207947249754395

FP279 0.207849485007913

FP151 0.20623099261347

PubchemFP429 0.205956705097051

GraphFP61 0.204778030682512

AD2D410 0.203757174897107

SubFP75 0.20374740641528

GraphFP343 0.203575133291376

KRFP3056 0.203427953698743

APC2D6_N_S 0.202003196218376

GraphFP991 0.201996663925355

KRFP2137 0.20180925704453

KRFP547 0.20071244819921

PubchemFP309 0.200164886005867

APC2D6_C_Br 0.197974798679317

KRFP312 0.197818419995879

FP95 0.196482462819308

FP751 0.196318167941497

PubchemFP350 0.195264369648158

AD2D579 0.194481589120553

PubchemFP156 0.191000212577862

FP528 0.189548032109357

GraphFP147 0.188526700937089

FP713 0.187879611562441

KRFP4842 0.184977049187786

FP971 0.183903794109931

FP274 0.181646144382903

GraphFP259 0.181430361437551

APC2D1_C_S 0.180100622236101

FP667 0.179026621658301

GraphFP883 0.178905510196199

GraphFP439 0.178687397671509

FP783 0.177906827375563

GraphFP108 0.175472519137815

MACCSFP144 0.173494978877141

GraphFP727 0.173160205362363

FP760 0.169487907446897

PubchemFP377 0.167618045288247

FP901 0.166859797913679

FP351 0.164902365861758

GraphFP1023 0.164767940805682

KRFP4193 0.164301034874875

APC2D6_O_F 0.161222507885466

GraphFP983 0.159741291876185

FP442 0.149231534624146

SubFP288 0.148029680470793

FP469 0.146439575905967

KRFP4320 0.139075968737923

FP857 0.139039172204762

FP804 0.132619881208099

GraphFP207 0.130111522598516

KRFP3710 0.125022310578596

GraphFP851 0.122200146764263

KRFP4399 0.121969407013932

EStateFP53 0.121573305640791

KRFP291 0.121397779029746

KRFP98 0.120993338199448

GraphFP650 0.119603185113487

MACCSFP30 0.115532746718186

MACCSFP31 0.111885439021936

KRFP3596 0.110589004189883

GraphFP222 0.109885413619885

FP796 0.109581535857619

FP479 0.105733882996986

MACCSFP45 0.104779596521743

PubchemFP129 0.0975386843280367

FP440 0.0965710760306352

FP21 0.0906505500122334

KRFP3645 0.0900512053456633

FP624 0.0868584386129659

SubFPC293 0.0866954671433652

GraphFP189 0.0852489734435099

FP653 0.0852432565477803

GraphFP125 0.0843325409513861

PubchemFP396 0.0776074402497378

FP105 0.0766544686567369

GraphFP961 0.0648163988039224

KRFP4663 0.055805856809411

FP237 0.0553668318800321

SubFPC292 0.021851902078359

AD2D453 0.0212389575673557

GraphFP177 0.0166766387004087

KRFP4668 0.016023903661972

PubchemFP601 0.0160054961864048

FP201 0.0142619830341937

SubFP39 0.0134425212052551

PubchemFP421 0.0133239420700301

KRFP4328 0.00947000736107146

KRFP4074 0.00945246672471915

GraphFP640 0.00920361117927131

GraphFP930 0.00883866370570168

GraphFP187 0.00862905896403921

KRFP3681 0.00823012255178687

GraphFP1012 0.00821310902936486

SubFP170 0.00735364691553033

AD2D165 0.00731718734582679

KRFP4185 0.00676730067035658

FP755 0.00664046826946863

KRFP3606 0.005749813257837

GraphFP1004 0.00568097567666516

FP115 0.00550951028723279

AD2D415 0.00501737289244584

KRFP3943 0.0048314024259628

AD2D259 0.00475924723826041

GraphFP146 0.00470797786039094

KRFP366 0.00467898644152039

GraphFP843 0.00420249807444586

GraphFP538 0.00407124313265399

GraphFP802 0.00393822506864459

APC2D1_S_S 0.00374214884337753

PubchemFP298 0.0037397762871527

KRFP4591 0.00373586501555154

PubchemFP116 0.00344157489353087

GraphFP16 0.00343871057881606

GraphFP625 0.00330083641774634

KRFP375 0.00317288090121036

KRFP1263 0.00286772892499088

MACCSFP8 0.00261148874931526

GraphFP208 0.0021450421477788

GraphFP24 0.00214448478663796

KRFP3591 0.0021187673500827

FP459 0.00162202972367568

KRFP3756 0.00159042469510728

GraphFP629 0.00157326769744273

KRFP1910 0.00156049053356184

GraphFP402 0.00132923533319372

PubchemFP483 0.0010515150643918

GraphFP469 0.000704868955131092

GraphFP765 0.000532363068032819

PubchemFP417 0.000522731505941474

GraphFP369 0.000159936495246224

SubFPC98 0

SubFPC97 0

SubFPC95 0

SubFPC94 0

SubFPC93 0

SubFPC92 0

SubFPC91 0

SubFPC90 0

SubFPC89 0

SubFPC87 0

SubFPC83 0

SubFPC82 0

SubFPC81 0

SubFPC80 0

SubFPC79 0

SubFPC78 0

SubFPC77 0

SubFPC76 0

SubFPC73 0

SubFPC72 0

SubFPC71 0

SubFPC70 0

SubFPC7 0

SubFPC69 0

SubFPC68 0

SubFPC67 0

SubFPC66 0

SubFPC65 0

SubFPC64 0

SubFPC62 0

SubFPC61 0

SubFPC60 0

SubFPC59 0

SubFPC58 0

SubFPC56 0

SubFPC55 0

SubFPC54 0

SubFPC51 0

SubFPC50 0

SubFPC46 0

SubFPC45 0

SubFPC44 0

SubFPC42 0

SubFPC38 0

SubFPC34 0

SubFPC33 0

SubFPC31 0

SubFPC306 0

SubFPC305 0

SubFPC304 0

SubFPC30 0

SubFPC290 0

SubFPC29 0

SubFPC289 0

SubFPC28 0

SubFPC278 0

SubFPC277 0

SubFPC273 0

SubFPC272 0

SubFPC271 0

SubFPC270 0

SubFPC269 0

SubFPC268 0

SubFPC267 0

SubFPC266 0

SubFPC265 0

SubFPC264 0

SubFPC263 0

SubFPC262 0

SubFPC261 0

SubFPC260 0

SubFPC259 0

SubFPC258 0

SubFPC257 0

SubFPC256 0

SubFPC255 0

SubFPC254 0

SubFPC253 0

SubFPC252 0

SubFPC251 0

SubFPC250 0

SubFPC25 0

SubFPC249 0

SubFPC248 0

SubFPC247 0

SubFPC245 0

SubFPC244 0

SubFPC243 0

SubFPC242 0

SubFPC241 0

SubFPC240 0

SubFPC24 0

SubFPC238 0

SubFPC236 0

SubFPC235 0

SubFPC234 0

SubFPC233 0

SubFPC232 0

SubFPC231 0

SubFPC230 0

SubFPC229 0

SubFPC228 0

SubFPC227 0

SubFPC226 0

SubFPC225 0

SubFPC223 0

SubFPC222 0

SubFPC221 0

SubFPC220 0

SubFPC22 0

SubFPC219 0

SubFPC218 0

SubFPC217 0

SubFPC216 0

SubFPC214 0

SubFPC213 0

SubFPC212 0

SubFPC211 0

SubFPC210 0

SubFPC21 0

SubFPC208 0

SubFPC207 0

SubFPC206 0

SubFPC205 0

SubFPC204 0

SubFPC203 0

SubFPC200 0

SubFPC20 0

SubFPC199 0

SubFPC198 0

SubFPC197 0

SubFPC196 0

SubFPC195 0

SubFPC194 0

SubFPC193 0

SubFPC192 0

SubFPC191 0

SubFPC189 0

SubFPC187 0

SubFPC186 0

SubFPC185 0

SubFPC182 0

SubFPC178 0

SubFPC177 0

SubFPC176 0

SubFPC175 0

SubFPC174 0

SubFPC173 0

SubFPC172 0

SubFPC168 0

SubFPC167 0

SubFPC166 0

SubFPC164 0

SubFPC162 0

SubFPC161 0

SubFPC160 0

SubFPC159 0

SubFPC158 0

SubFPC157 0

SubFPC156 0

SubFPC155 0

SubFPC154 0

SubFPC152 0

SubFPC150 0

SubFPC149 0

SubFPC148 0

SubFPC147 0

SubFPC146 0

SubFPC145 0

SubFPC144 0

SubFPC142 0

SubFPC141 0

SubFPC140 0

SubFPC139 0

SubFPC138 0

SubFPC132 0

SubFPC131 0

SubFPC130 0

SubFPC129 0

SubFPC127 0

SubFPC125 0

SubFPC124 0

SubFPC123 0

SubFPC122 0

SubFPC121 0

SubFPC120 0

SubFPC119 0

SubFPC118 0

SubFPC117 0

SubFPC116 0

SubFPC115 0

SubFPC114 0

SubFPC113 0

SubFPC112 0

SubFPC111 0

SubFPC110 0

SubFPC108 0

SubFPC107 0

SubFPC106 0

SubFPC105 0

SubFPC104 0

SubFPC103 0

SubFPC102 0

SubFP98 0

SubFP97 0

SubFP95 0

SubFP94 0

SubFP93 0

SubFP92 0

SubFP91 0

SubFP90 0

SubFP89 0

SubFP87 0

SubFP83 0

SubFP82 0

SubFP81 0

SubFP80 0

SubFP79 0

SubFP78 0

SubFP77 0

SubFP76 0

SubFP73 0

SubFP70 0

SubFP7 0

SubFP69 0

SubFP67 0

SubFP66 0

SubFP65 0

SubFP64 0

SubFP62 0

SubFP61 0

SubFP60 0

SubFP59 0

SubFP58 0

SubFP56 0

SubFP55 0

SubFP54 0

SubFP53 0

SubFP51 0

SubFP50 0

SubFP46 0

SubFP45 0

SubFP44 0

SubFP42 0

SubFP38 0

SubFP34 0

SubFP33 0

SubFP31 0

SubFP306 0

SubFP305 0

SubFP304 0

SubFP30 0

SubFP294 0

SubFP290 0

SubFP29 0

SubFP289 0

SubFP28 0

SubFP278 0

SubFP277 0

SubFP273 0

SubFP272 0

SubFP271 0

SubFP270 0

SubFP269 0

SubFP268 0

SubFP267 0

SubFP266 0

SubFP265 0

SubFP264 0

SubFP263 0

SubFP262 0

SubFP261 0

SubFP260 0

SubFP259 0

SubFP258 0

SubFP257 0

SubFP256 0

SubFP255 0

SubFP254 0

SubFP253 0

SubFP252 0

SubFP251 0

SubFP250 0

SubFP25 0

SubFP249 0

SubFP248 0

SubFP247 0

SubFP245 0

SubFP244 0

SubFP243 0

SubFP242 0

SubFP241 0

SubFP240 0

SubFP24 0

SubFP238 0

SubFP236 0

SubFP235 0

SubFP234 0

SubFP233 0

SubFP232 0

SubFP231 0

SubFP230 0

SubFP229 0

SubFP228 0

SubFP227 0

SubFP226 0

SubFP225 0

SubFP223 0

SubFP222 0

SubFP221 0

SubFP220 0

SubFP219 0

SubFP218 0

SubFP217 0

SubFP216 0

SubFP214 0

SubFP213 0

SubFP212 0

SubFP211 0

SubFP21 0

SubFP208 0

SubFP207 0

SubFP206 0

SubFP205 0

SubFP203 0

SubFP201 0

SubFP200 0

SubFP20 0

SubFP199 0

SubFP198 0

SubFP197 0

SubFP196 0

SubFP195 0

SubFP194 0

SubFP193 0

SubFP192 0

SubFP191 0

SubFP19 0

SubFP189 0

SubFP187 0

SubFP186 0

SubFP185 0

SubFP183 0

SubFP182 0

SubFP178 0

SubFP177 0

SubFP176 0

SubFP175 0

SubFP173 0

SubFP172 0

SubFP168 0

SubFP167 0

SubFP166 0

SubFP164 0

SubFP162 0

SubFP161 0

SubFP160 0

SubFP159 0

SubFP158 0

SubFP157 0

SubFP156 0

SubFP155 0

SubFP154 0

SubFP152 0

SubFP150 0

SubFP149 0

SubFP148 0

SubFP147 0

SubFP146 0

SubFP145 0

SubFP144 0

SubFP142 0

SubFP141 0

SubFP140 0

SubFP139 0

SubFP138 0

SubFP132 0

SubFP131 0

SubFP130 0

SubFP129 0

SubFP127 0

SubFP124 0

SubFP123 0

SubFP122 0

SubFP121 0

SubFP120 0

SubFP119 0

SubFP118 0

SubFP117 0

SubFP116 0

SubFP115 0

SubFP114 0

SubFP113 0

SubFP112 0

SubFP111 0

SubFP110 0

SubFP11 0

SubFP108 0

SubFP107 0

SubFP106 0

SubFP105 0

SubFP104 0

SubFP103 0

SubFP102 0

PubchemFP99 0

PubchemFP98 0

PubchemFP96 0

PubchemFP94 0

PubchemFP93 0

PubchemFP92 0

PubchemFP91 0

PubchemFP90 0

PubchemFP89 0

PubchemFP880 0

PubchemFP88 0

PubchemFP879 0

PubchemFP877 0

PubchemFP876 0

PubchemFP875 0

PubchemFP874 0

PubchemFP873 0

PubchemFP872 0

PubchemFP871 0

PubchemFP870 0

PubchemFP87 0

PubchemFP869 0

PubchemFP868 0

PubchemFP867 0

PubchemFP866 0

PubchemFP865 0

PubchemFP863 0

PubchemFP862 0

PubchemFP86 0

PubchemFP859 0

PubchemFP858 0

PubchemFP856 0

PubchemFP855 0

PubchemFP854 0

PubchemFP853 0

PubchemFP852 0

PubchemFP851 0

PubchemFP850 0

PubchemFP85 0

PubchemFP849 0

PubchemFP848 0

PubchemFP847 0

PubchemFP846 0

PubchemFP844 0

PubchemFP843 0

PubchemFP842 0

PubchemFP841 0

PubchemFP84 0

PubchemFP838 0

PubchemFP837 0

PubchemFP836 0

PubchemFP835 0

PubchemFP834 0

PubchemFP832 0

PubchemFP831 0

PubchemFP830 0

PubchemFP83 0

PubchemFP829 0

PubchemFP828 0

PubchemFP827 0

PubchemFP825 0

PubchemFP823 0

PubchemFP822 0

PubchemFP820 0

PubchemFP82 0

PubchemFP817 0

PubchemFP816 0

PubchemFP814 0

PubchemFP813 0

PubchemFP811 0

PubchemFP810 0

PubchemFP81 0

PubchemFP809 0

PubchemFP808 0

PubchemFP807 0

PubchemFP804 0

PubchemFP802 0

PubchemFP801 0

PubchemFP8 0

PubchemFP799 0

PubchemFP796 0

PubchemFP795 0

PubchemFP794 0

PubchemFP793 0

PubchemFP791 0

PubchemFP790 0

PubchemFP79 0

PubchemFP789 0

PubchemFP788 0

PubchemFP787 0

PubchemFP786 0

PubchemFP785 0

PubchemFP783 0

PubchemFP781 0

PubchemFP780 0

PubchemFP778 0

PubchemFP775 0

PubchemFP774 0

PubchemFP772 0

PubchemFP771 0

PubchemFP77 0

PubchemFP769 0

PubchemFP768 0

PubchemFP767 0

PubchemFP766 0

PubchemFP765 0

PubchemFP764 0

PubchemFP763 0

PubchemFP762 0

PubchemFP760 0

PubchemFP76 0

PubchemFP759 0

PubchemFP757 0

PubchemFP754 0

PubchemFP753 0

PubchemFP752 0

PubchemFP751 0

PubchemFP750 0

PubchemFP75 0

PubchemFP749 0

PubchemFP748 0

PubchemFP747 0

PubchemFP746 0

PubchemFP745 0

PubchemFP744 0

PubchemFP743 0

PubchemFP742 0

PubchemFP741 0

PubchemFP74 0

PubchemFP739 0

PubchemFP736 0

PubchemFP733 0

PubchemFP732 0

PubchemFP731 0

PubchemFP730 0

PubchemFP73 0

PubchemFP729 0

PubchemFP728 0

PubchemFP727 0

PubchemFP726 0

PubchemFP725 0

PubchemFP724 0

PubchemFP723 0

PubchemFP722 0

PubchemFP720 0

PubchemFP72 0

PubchemFP718 0

PubchemFP717 0

PubchemFP716 0

PubchemFP71 0

PubchemFP70 0

PubchemFP7 0

PubchemFP69 0

PubchemFP68 0

PubchemFP676 0

PubchemFP67 0

PubchemFP649 0

PubchemFP648 0

PubchemFP644 0

PubchemFP64 0

PubchemFP635 0

PubchemFP63 0

PubchemFP629 0

PubchemFP627 0

PubchemFP616 0

PubchemFP609 0

PubchemFP60 0

PubchemFP6 0

PubchemFP587 0

PubchemFP583 0

PubchemFP57 0

PubchemFP562 0

PubchemFP561 0

PubchemFP558 0

PubchemFP554 0

PubchemFP55 0

PubchemFP54 0

PubchemFP534 0

PubchemFP531 0

PubchemFP53 0

PubchemFP526 0

PubchemFP525 0

PubchemFP522 0

PubchemFP52 0

PubchemFP518 0

PubchemFP515 0

PubchemFP513 0

PubchemFP512 0

PubchemFP511 0

PubchemFP510 0

PubchemFP509 0

PubchemFP508 0

PubchemFP505 0

PubchemFP500 0

PubchemFP50 0

PubchemFP5 0

PubchemFP496 0

PubchemFP492 0

PubchemFP49 0

PubchemFP489 0

PubchemFP486 0

PubchemFP481 0

PubchemFP480 0

PubchemFP479 0

PubchemFP478 0

PubchemFP477 0

PubchemFP473 0

PubchemFP471 0

PubchemFP469 0

PubchemFP468 0

PubchemFP463 0

PubchemFP45 0

PubchemFP444 0

PubchemFP433 0

PubchemFP426 0

PubchemFP424 0

PubchemFP422 0

PubchemFP42 0

PubchemFP415 0

PubchemFP410 0

PubchemFP41 0

PubchemFP409 0

PubchemFP404 0

PubchemFP402 0

PubchemFP40 0

PubchemFP4 0

PubchemFP398 0

PubchemFP383 0

PubchemFP369 0

PubchemFP364 0

PubchemFP36 0

PubchemFP354 0

PubchemFP343 0

PubchemFP331 0

PubchemFP330 0

PubchemFP327 0

PubchemFP326 0

PubchemFP325 0

PubchemFP324 0

PubchemFP322 0

PubchemFP321 0

PubchemFP320 0

PubchemFP32 0

PubchemFP319 0

PubchemFP318 0

PubchemFP317 0

PubchemFP316 0

PubchemFP315 0

PubchemFP313 0

PubchemFP312 0

PubchemFP311 0

PubchemFP310 0

PubchemFP307 0

PubchemFP306 0

PubchemFP304 0

PubchemFP303 0

PubchemFP302 0

PubchemFP296 0

PubchemFP291 0

PubchemFP290 0

PubchemFP29 0

PubchemFP289 0

PubchemFP288 0

PubchemFP282 0

PubchemFP281 0

PubchemFP280 0

PubchemFP28 0

PubchemFP279 0

PubchemFP278 0

PubchemFP277 0

PubchemFP276 0

PubchemFP275 0

PubchemFP274 0

PubchemFP273 0

PubchemFP272 0

PubchemFP271 0

PubchemFP270 0

PubchemFP27 0

PubchemFP269 0

PubchemFP268 0

PubchemFP267 0

PubchemFP266 0

PubchemFP265 0

PubchemFP264 0

PubchemFP263 0

PubchemFP262 0

PubchemFP261 0

PubchemFP260 0

PubchemFP254 0

PubchemFP253 0

PubchemFP252 0

PubchemFP251 0

PubchemFP250 0

PubchemFP25 0

PubchemFP249 0

PubchemFP248 0

PubchemFP247 0

PubchemFP246 0

PubchemFP244 0

PubchemFP243 0

PubchemFP242 0

PubchemFP241 0

PubchemFP240 0

PubchemFP24 0

PubchemFP239 0

PubchemFP238 0

PubchemFP237 0

PubchemFP236 0

PubchemFP235 0

PubchemFP234 0

PubchemFP233 0

PubchemFP231 0

PubchemFP230 0

PubchemFP229 0

PubchemFP227 0

PubchemFP226 0

PubchemFP225 0

PubchemFP224 0

PubchemFP223 0

PubchemFP222 0

PubchemFP221 0

PubchemFP220 0

PubchemFP218 0

PubchemFP217 0

PubchemFP216 0

PubchemFP215 0

PubchemFP214 0

PubchemFP212 0

PubchemFP211 0

PubchemFP210 0

PubchemFP208 0

PubchemFP207 0

PubchemFP205 0

PubchemFP204 0

PubchemFP201 0

PubchemFP198 0

PubchemFP197 0

PubchemFP194 0

PubchemFP187 0

PubchemFP177 0

PubchemFP176 0

PubchemFP175 0

PubchemFP174 0

PubchemFP173 0

PubchemFP172 0

PubchemFP171 0

PubchemFP168 0

PubchemFP167 0

PubchemFP166 0

PubchemFP165 0

PubchemFP161 0

PubchemFP159 0

PubchemFP158 0

PubchemFP154 0

PubchemFP152 0

PubchemFP142 0

PubchemFP141 0

PubchemFP140 0

PubchemFP139 0

PubchemFP138 0

PubchemFP137 0

PubchemFP136 0

PubchemFP135 0

PubchemFP134 0

PubchemFP133 0

PubchemFP131 0

PubchemFP128 0

PubchemFP127 0

PubchemFP126 0

PubchemFP125 0

PubchemFP124 0

PubchemFP123 0

PubchemFP122 0

PubchemFP121 0

PubchemFP120 0

PubchemFP119 0

PubchemFP117 0

PubchemFP114 0

PubchemFP113 0

PubchemFP112 0

PubchemFP111 0

PubchemFP110 0

PubchemFP109 0

PubchemFP108 0

PubchemFP107 0

PubchemFP106 0

PubchemFP105 0

PubchemFP104 0

PubchemFP103 0

PubchemFP102 0

PubchemFP101 0

PubchemFP100 0

MACCSFP6 0

MACCSFP59 0

MACCSFP5 0

MACCSFP44 0

MACCSFP4 0

MACCSFP36 0

MACCSFP33 0

MACCSFP32 0

MACCSFP20 0

MACCSFP2 0

MACCSFP13 0

MACCSFP101 0

MACCSFP10 0

MACCSFP1 0

KRFP999 0

KRFP998 0

KRFP997 0

KRFP996 0

KRFP995 0

KRFP994 0

KRFP993 0

KRFP992 0

KRFP991 0

KRFP990 0

KRFP989 0

KRFP988 0

KRFP987 0

KRFP986 0

KRFP985 0

KRFP984 0

KRFP982 0

KRFP981 0

KRFP980 0

KRFP979 0

KRFP978 0

KRFP977 0

KRFP976 0

KRFP975 0

KRFP974 0

KRFP972 0

KRFP971 0

KRFP970 0

KRFP97 0

KRFP969 0

KRFP968 0

KRFP967 0

KRFP966 0

KRFP965 0

KRFP964 0

KRFP963 0

KRFP962 0

KRFP961 0

KRFP960 0

KRFP96 0

KRFP959 0

KRFP958 0

KRFP957 0

KRFP956 0

KRFP955 0

KRFP954 0

KRFP953 0

KRFP952 0

KRFP951 0

KRFP950 0

KRFP95 0

KRFP949 0

KRFP948 0

KRFP947 0

KRFP946 0

KRFP945 0

KRFP944 0

KRFP943 0

KRFP942 0

KRFP941 0

KRFP940 0

KRFP94 0

KRFP939 0

KRFP938 0

KRFP937 0

KRFP935 0

KRFP934 0

KRFP933 0

KRFP932 0

KRFP931 0

KRFP930 0

KRFP93 0

KRFP929 0

KRFP928 0

KRFP927 0

KRFP926 0

KRFP925 0

KRFP924 0

KRFP923 0

KRFP922 0

KRFP921 0

KRFP920 0

KRFP919 0

KRFP918 0

KRFP917 0

KRFP916 0

KRFP915 0

KRFP914 0

KRFP913 0

KRFP912 0

KRFP911 0

KRFP910 0

KRFP909 0

KRFP908 0

KRFP907 0

KRFP906 0

KRFP905 0

KRFP904 0

KRFP903 0

KRFP902 0

KRFP901 0

KRFP900 0

KRFP90 0

KRFP9 0

KRFP899 0

KRFP898 0

KRFP897 0

KRFP896 0

KRFP895 0

KRFP894 0

KRFP893 0

KRFP892 0

KRFP891 0

KRFP89 0

KRFP889 0

KRFP888 0

KRFP887 0

KRFP886 0

KRFP885 0

KRFP884 0

KRFP883 0

KRFP882 0

KRFP881 0

KRFP880 0

KRFP88 0

KRFP879 0

KRFP878 0

KRFP877 0

KRFP876 0

KRFP875 0

KRFP874 0

KRFP873 0

KRFP872 0

KRFP871 0

KRFP870 0

KRFP87 0

KRFP869 0

KRFP868 0

KRFP867 0

KRFP866 0

KRFP865 0

KRFP864 0

KRFP863 0

KRFP862 0

KRFP861 0

KRFP860 0

KRFP86 0

KRFP859 0

KRFP858 0

KRFP857 0

KRFP856 0

KRFP855 0

KRFP854 0

KRFP853 0

KRFP852 0

KRFP851 0

KRFP850 0

KRFP85 0

KRFP849 0

KRFP848 0

KRFP847 0

KRFP846 0

KRFP845 0

KRFP844 0

KRFP843 0

KRFP842 0

KRFP841 0

KRFP84 0

KRFP838 0

KRFP837 0

KRFP836 0

KRFP835 0

KRFP834 0

KRFP833 0

KRFP832 0

KRFP831 0

KRFP830 0

KRFP83 0

KRFP829 0

KRFP828 0

KRFP827 0

KRFP826 0

KRFP825 0

KRFP824 0

KRFP823 0

KRFP822 0

KRFP820 0

KRFP82 0

KRFP819 0

KRFP818 0

KRFP817 0

KRFP816 0

KRFP815 0

KRFP814 0

KRFP813 0

KRFP812 0

KRFP811 0

KRFP81 0

KRFP809 0

KRFP808 0

KRFP807 0

KRFP806 0

KRFP805 0

KRFP804 0

KRFP803 0

KRFP802 0

KRFP801 0

KRFP800 0

KRFP80 0

KRFP799 0

KRFP798 0

KRFP796 0

KRFP795 0

KRFP794 0

KRFP793 0

KRFP792 0

KRFP791 0

KRFP79 0

KRFP789 0

KRFP788 0

KRFP786 0

KRFP785 0

KRFP784 0

KRFP783 0

KRFP782 0

KRFP781 0

KRFP780 0

KRFP78 0

KRFP779 0

KRFP777 0

KRFP776 0

KRFP775 0

KRFP774 0

KRFP773 0

KRFP772 0

KRFP771 0

KRFP770 0

KRFP77 0

KRFP767 0

KRFP766 0

KRFP765 0

KRFP764 0

KRFP763 0

KRFP762 0

KRFP761 0

KRFP760 0

KRFP759 0

KRFP757 0

KRFP756 0

KRFP755 0

KRFP754 0

KRFP753 0

KRFP752 0

KRFP751 0

KRFP750 0

KRFP75 0

KRFP749 0

KRFP748 0

KRFP747 0

KRFP746 0

KRFP745 0

KRFP744 0

KRFP743 0

KRFP742 0

KRFP741 0

KRFP740 0

KRFP74 0

KRFP739 0

KRFP738 0

KRFP737 0

KRFP736 0

KRFP735 0

KRFP734 0

KRFP733 0

KRFP732 0

KRFP731 0

KRFP730 0

KRFP73 0

KRFP729 0

KRFP728 0

KRFP727 0

KRFP726 0

KRFP725 0

KRFP724 0

KRFP723 0

KRFP722 0

KRFP721 0

KRFP720 0

KRFP719 0

KRFP718 0

KRFP717 0

KRFP716 0

KRFP715 0

KRFP714 0

KRFP713 0

KRFP712 0

KRFP711 0

KRFP710 0

KRFP71 0

KRFP709 0

KRFP708 0

KRFP707 0

KRFP706 0

KRFP705 0

KRFP704 0

KRFP703 0

KRFP702 0

KRFP701 0

KRFP700 0

KRFP70 0

KRFP7 0

KRFP699 0

KRFP698 0

KRFP697 0

KRFP696 0

KRFP695 0

KRFP694 0

KRFP693 0

KRFP692 0

KRFP691 0

KRFP690 0

KRFP69 0

KRFP689 0

KRFP688 0

KRFP687 0

KRFP686 0

KRFP685 0

KRFP684 0

KRFP681 0

KRFP680 0

KRFP68 0

KRFP679 0

KRFP676 0

KRFP675 0

KRFP674 0

KRFP673 0

KRFP668 0

KRFP667 0

KRFP666 0

KRFP664 0

KRFP663 0

KRFP661 0

KRFP660 0

KRFP66 0

KRFP659 0

KRFP657 0

KRFP656 0

KRFP655 0

KRFP652 0

KRFP651 0

KRFP650 0

KRFP65 0

KRFP649 0

KRFP648 0

KRFP646 0

KRFP645 0

KRFP644 0

KRFP643 0

KRFP642 0

KRFP641 0

KRFP640 0

KRFP64 0

KRFP639 0

KRFP638 0

KRFP637 0

KRFP635 0

KRFP634 0

KRFP633 0

KRFP632 0

KRFP631 0

KRFP63 0

KRFP628 0

KRFP627 0

KRFP626 0

KRFP625 0

KRFP624 0

KRFP623 0

KRFP622 0

KRFP621 0

KRFP62 0

KRFP619 0

KRFP618 0

KRFP617 0

KRFP616 0

KRFP615 0

KRFP614 0

KRFP613 0

KRFP612 0

KRFP611 0

KRFP610 0

KRFP61 0

KRFP609 0

KRFP606 0

KRFP604 0

KRFP603 0

KRFP602 0

KRFP601 0

KRFP600 0

KRFP60 0

KRFP6 0

KRFP599 0

KRFP598 0

KRFP597 0

KRFP596 0

KRFP595 0

KRFP594 0

KRFP593 0

KRFP592 0

KRFP591 0

KRFP590 0

KRFP59 0

KRFP589 0

KRFP588 0

KRFP587 0

KRFP586 0

KRFP585 0

KRFP584 0

KRFP583 0

KRFP581 0

KRFP580 0

KRFP58 0

KRFP579 0

KRFP578 0

KRFP577 0

KRFP576 0

KRFP575 0

KRFP574 0

KRFP573 0

KRFP572 0

KRFP571 0

KRFP570 0

KRFP57 0

KRFP569 0

KRFP568 0

KRFP567 0

KRFP565 0

KRFP564 0

KRFP563 0

KRFP561 0

KRFP560 0

KRFP56 0

KRFP559 0

KRFP558 0

KRFP556 0

KRFP555 0

KRFP553 0

KRFP552 0

KRFP551 0

KRFP550 0

KRFP55 0

KRFP549 0

KRFP546 0

KRFP545 0

KRFP544 0

KRFP543 0

KRFP542 0

KRFP541 0

KRFP540 0

KRFP54 0

KRFP539 0

KRFP538 0

KRFP537 0

KRFP536 0

KRFP535 0

KRFP534 0

KRFP533 0

KRFP532 0

KRFP531 0

KRFP530 0

KRFP53 0

KRFP529 0

KRFP528 0

KRFP527 0

KRFP526 0

KRFP525 0

KRFP524 0

KRFP523 0

KRFP522 0

KRFP521 0

KRFP520 0

KRFP52 0

KRFP519 0

KRFP518 0

KRFP517 0

KRFP516 0

KRFP515 0

KRFP514 0

KRFP513 0

KRFP512 0

KRFP511 0

KRFP510 0

KRFP51 0

KRFP509 0

KRFP508 0

KRFP507 0

KRFP503 0

KRFP502 0

KRFP501 0

KRFP500 0

KRFP50 0

KRFP5 0

KRFP499 0

KRFP498 0

KRFP497 0

KRFP496 0

KRFP495 0

KRFP492 0

KRFP491 0

KRFP490 0

KRFP49 0

KRFP489 0

KRFP488 0

KRFP4860 0

KRFP486 0

KRFP4859 0

KRFP4857 0

KRFP4855 0

KRFP4854 0

KRFP4851 0

KRFP4850 0

KRFP485 0

KRFP4849 0

KRFP4848 0

KRFP4847 0

KRFP4846 0

KRFP4845 0

KRFP4844 0

KRFP4841 0

KRFP4840 0

KRFP484 0

KRFP4839 0

KRFP4838 0

KRFP4837 0

KRFP4835 0

KRFP4833 0

KRFP4832 0

KRFP4831 0

KRFP4828 0

KRFP4825 0

KRFP4822 0

KRFP482 0

KRFP4819 0

KRFP4816 0

KRFP4815 0

KRFP4814 0

KRFP4812 0

KRFP481 0

KRFP4809 0

KRFP4808 0

KRFP4807 0

KRFP4806 0

KRFP4805 0

KRFP4804 0

KRFP4802 0

KRFP4801 0

KRFP4800 0

KRFP48 0

KRFP4799 0

KRFP4798 0

KRFP4797 0

KRFP4796 0

KRFP4795 0

KRFP4794 0

KRFP4793 0

KRFP4792 0

KRFP4791 0

KRFP4790 0

KRFP479 0

KRFP4789 0

KRFP4788 0

KRFP4787 0

KRFP4786 0

KRFP4785 0

KRFP4784 0

KRFP4783 0

KRFP4782 0

KRFP4781 0

KRFP4780 0

KRFP478 0

KRFP4779 0

KRFP4778 0

KRFP4777 0

KRFP4776 0

KRFP4775 0

KRFP4773 0

KRFP4772 0

KRFP4771 0

KRFP477 0

KRFP4769 0

KRFP4768 0

KRFP4766 0

KRFP4765 0

KRFP4764 0

KRFP4762 0

KRFP4761 0

KRFP4760 0

KRFP4759 0

KRFP4758 0

KRFP4756 0

KRFP4755 0

KRFP4753 0

KRFP4751 0

KRFP4750 0

KRFP475 0

KRFP4748 0

KRFP4746 0

KRFP4745 0

KRFP4744 0

KRFP4743 0

KRFP4742 0

KRFP4741 0

KRFP4740 0

KRFP474 0

KRFP4739 0

KRFP4738 0

KRFP4737 0

KRFP4736 0

KRFP4735 0

KRFP4734 0

KRFP4733 0

KRFP4732 0

KRFP4731 0

KRFP4730 0

KRFP473 0

KRFP4729 0

KRFP4727 0

KRFP4726 0

KRFP4725 0

KRFP4724 0

KRFP4723 0

KRFP4722 0

KRFP4721 0

KRFP4720 0

KRFP472 0

KRFP4719 0

KRFP4718 0

KRFP4717 0

KRFP4716 0

KRFP4715 0

KRFP4714 0

KRFP4713 0

KRFP4712 0

KRFP4711 0

KRFP4710 0

KRFP471 0

KRFP4709 0

KRFP4707 0

KRFP4706 0

KRFP4705 0

KRFP4704 0

KRFP4703 0

KRFP4702 0

KRFP4701 0

KRFP4700 0

KRFP470 0

KRFP47 0

KRFP4699 0

KRFP4698 0

KRFP4697 0

KRFP4696 0

KRFP4694 0

KRFP4693 0

KRFP4692 0

KRFP4691 0

KRFP4690 0

KRFP469 0

KRFP4688 0

KRFP4687 0

KRFP4686 0

KRFP4685 0

KRFP4684 0

KRFP4683 0

KRFP4682 0

KRFP4681 0

KRFP4680 0

KRFP4679 0

KRFP4677 0

KRFP4676 0

KRFP4675 0

KRFP4674 0

KRFP4673 0

KRFP4672 0

KRFP4670 0

KRFP4669 0

KRFP4662 0

KRFP4660 0

KRFP4658 0

KRFP4656 0

KRFP4655 0

KRFP4654 0

KRFP4653 0

KRFP4652 0

KRFP4651 0

KRFP4650 0

KRFP465 0

KRFP4649 0

KRFP4648 0

KRFP4647 0

KRFP4646 0

KRFP4644 0

KRFP4643 0

KRFP4642 0

KRFP4641 0

KRFP4640 0

KRFP464 0

KRFP4639 0

KRFP4638 0

KRFP4637 0

KRFP4636 0

KRFP4635 0

KRFP4634 0

KRFP4633 0

KRFP4632 0

KRFP4631 0

KRFP4630 0

KRFP463 0

KRFP4629 0

KRFP4628 0

KRFP4627 0

KRFP4626 0

KRFP4625 0

KRFP4624 0

KRFP4623 0

KRFP4622 0

KRFP4621 0

KRFP4620 0

KRFP462 0

KRFP4619 0

KRFP4618 0

KRFP4617 0

KRFP4616 0

KRFP4615 0

KRFP4614 0

KRFP4613 0

KRFP4611 0

KRFP461 0

KRFP4609 0

KRFP4608 0

KRFP4607 0

KRFP4606 0

KRFP4605 0

KRFP4604 0

KRFP4603 0

KRFP4601 0

KRFP4600 0

KRFP460 0

KRFP46 0

KRFP4599 0

KRFP4598 0

KRFP4597 0

KRFP4596 0

KRFP4595 0

KRFP4594 0

KRFP4593 0

KRFP4592 0

KRFP4590 0

KRFP459 0

KRFP4589 0

KRFP4588 0

KRFP4587 0

KRFP4586 0

KRFP4585 0

KRFP4584 0

KRFP4582 0

KRFP4581 0

KRFP4580 0

KRFP4579 0

KRFP4578 0

KRFP4577 0

KRFP4576 0

KRFP4575 0

KRFP4574 0

KRFP4573 0

KRFP4572 0

KRFP4571 0

KRFP4570 0

KRFP457 0

KRFP4569 0

KRFP4568 0

KRFP4567 0

KRFP4566 0

KRFP4565 0

KRFP4564 0

KRFP4563 0

KRFP4562 0

KRFP4561 0

KRFP4560 0

KRFP456 0

KRFP4559 0

KRFP4558 0

KRFP4557 0

KRFP4556 0

KRFP4555 0

KRFP4554 0

KRFP4553 0

KRFP4552 0

KRFP4551 0

KRFP4550 0

KRFP455 0

KRFP4549 0

KRFP4548 0

KRFP4547 0

KRFP4546 0

KRFP4545 0

KRFP4544 0

KRFP4543 0

KRFP4542 0

KRFP4541 0

KRFP4540 0

KRFP454 0

KRFP4539 0

KRFP4538 0

KRFP4537 0

KRFP4536 0

KRFP4535 0

KRFP4534 0

KRFP4533 0

KRFP4532 0

KRFP4530 0

KRFP4529 0

KRFP4528 0

KRFP4527 0

KRFP4526 0

KRFP4520 0

KRFP452 0

KRFP4518 0

KRFP4517 0

KRFP4515 0

KRFP4514 0

KRFP4513 0

KRFP4511 0

KRFP4510 0

KRFP451 0

KRFP4509 0

KRFP4508 0

KRFP4507 0

KRFP4506 0

KRFP4505 0

KRFP4504 0

KRFP4503 0

KRFP4502 0

KRFP4501 0

KRFP4500 0

KRFP450 0

KRFP4497 0

KRFP4494 0

KRFP4493 0

KRFP4491 0

KRFP4490 0

KRFP449 0

KRFP4489 0

KRFP4488 0

KRFP4487 0

KRFP4486 0

KRFP4485 0

KRFP4484 0

KRFP4483 0

KRFP4482 0

KRFP4481 0

KRFP4480 0

KRFP448 0

KRFP4479 0

KRFP4478 0

KRFP4477 0

KRFP4476 0

KRFP4475 0

KRFP4474 0

KRFP4473 0

KRFP4471 0

KRFP4470 0

KRFP4469 0

KRFP4468 0

KRFP4467 0

KRFP4466 0

KRFP4465 0

KRFP4464 0

KRFP4463 0

KRFP4462 0

KRFP4461 0

KRFP4460 0

KRFP446 0

KRFP4459 0

KRFP4458 0

KRFP4457 0

KRFP4456 0

KRFP4455 0

KRFP4454 0

KRFP4453 0

KRFP4452 0

KRFP4451 0

KRFP4450 0

KRFP445 0

KRFP4449 0

KRFP4448 0

KRFP4447 0

KRFP4446 0

KRFP4445 0

KRFP4444 0

KRFP4443 0

KRFP4442 0

KRFP4441 0

KRFP4440 0

KRFP4439 0

KRFP4438 0

KRFP4437 0

KRFP4436 0

KRFP4435 0

KRFP4434 0

KRFP4433 0

KRFP4432 0

KRFP4431 0

KRFP4430 0

KRFP443 0

KRFP4429 0

KRFP4428 0

KRFP4427 0

KRFP4426 0

KRFP4425 0

KRFP4424 0

KRFP4423 0

KRFP4422 0

KRFP4421 0

KRFP4420 0

KRFP4419 0

KRFP4418 0

KRFP4417 0

KRFP4416 0

KRFP4415 0

KRFP4414 0

KRFP4412 0

KRFP4411 0

KRFP4410 0

KRFP441 0

KRFP4409 0

KRFP4407 0

KRFP4406 0

KRFP4405 0

KRFP4404 0

KRFP4403 0

KRFP4402 0

KRFP4401 0

KRFP4400 0

KRFP440 0

KRFP44 0

KRFP4397 0

KRFP4396 0

KRFP4395 0

KRFP4394 0

KRFP4393 0

KRFP4392 0

KRFP4391 0

KRFP4390 0

KRFP439 0

KRFP4389 0

KRFP4388 0

KRFP4387 0

KRFP4386 0

KRFP4385 0

KRFP4384 0

KRFP4383 0

KRFP4382 0

KRFP4381 0

KRFP4380 0

KRFP4379 0

KRFP4378 0

KRFP4377 0

KRFP4376 0

KRFP4375 0

KRFP4374 0

KRFP4373 0

KRFP4372 0

KRFP4371 0

KRFP4370 0

KRFP437 0

KRFP4369 0

KRFP4368 0

KRFP4367 0

KRFP4366 0

KRFP4365 0

KRFP4364 0

KRFP4363 0

KRFP4362 0

KRFP4361 0

KRFP436 0

KRFP4358 0

KRFP4357 0

KRFP4356 0

KRFP4355 0

KRFP4354 0

KRFP4353 0

KRFP4352 0

KRFP4351 0

KRFP4350 0

KRFP4349 0

KRFP4348 0

KRFP4347 0

KRFP4346 0

KRFP4345 0

KRFP4344 0

KRFP4343 0

KRFP4342 0

KRFP4341 0

KRFP4340 0

KRFP434 0

KRFP4339 0

KRFP4338 0

KRFP4337 0

KRFP4336 0

KRFP4335 0

KRFP4334 0

KRFP4333 0

KRFP4332 0

KRFP4330 0

KRFP433 0

KRFP4329 0

KRFP4327 0

KRFP4326 0

KRFP4325 0

KRFP4324 0

KRFP4323 0

KRFP4322 0

KRFP4321 0

KRFP432 0

KRFP4319 0

KRFP4318 0

KRFP4316 0

KRFP4314 0

KRFP4313 0

KRFP4312 0

KRFP4311 0

KRFP4310 0

KRFP431 0

KRFP4309 0

KRFP4308 0

KRFP4307 0

KRFP4306 0

KRFP4305 0

KRFP4304 0

KRFP4303 0

KRFP4302 0

KRFP4300 0

KRFP430 0

KRFP43 0

KRFP4299 0

KRFP4298 0

KRFP4297 0

KRFP4290 0

KRFP429 0

KRFP4289 0

KRFP4288 0

KRFP4282 0

KRFP4280 0

KRFP428 0

KRFP4279 0

KRFP4278 0

KRFP4277 0

KRFP4276 0

KRFP4275 0

KRFP4274 0

KRFP4273 0

KRFP4272 0

KRFP4271 0

KRFP4270 0

KRFP427 0

KRFP4269 0

KRFP4268 0

KRFP4267 0

KRFP4266 0

KRFP4265 0

KRFP4263 0

KRFP4262 0

KRFP4261 0

KRFP4260 0

KRFP426 0

KRFP4259 0

KRFP4258 0

KRFP4257 0

KRFP4256 0

KRFP4255 0

KRFP4253 0

KRFP4252 0

KRFP4251 0

KRFP4250 0

KRFP425 0

KRFP4249 0

KRFP4248 0

KRFP4247 0

KRFP4246 0

KRFP4245 0

KRFP4244 0

KRFP4242 0

KRFP4241 0

KRFP4240 0

KRFP424 0

KRFP4239 0

KRFP4236 0

KRFP4235 0

KRFP4234 0

KRFP4233 0

KRFP4232 0

KRFP4230 0

KRFP423 0

KRFP4229 0

KRFP4228 0

KRFP4227 0

KRFP4226 0

KRFP4224 0

KRFP4223 0

KRFP4222 0

KRFP4221 0

KRFP4220 0

KRFP422 0

KRFP4219 0

KRFP4218 0

KRFP4217 0

KRFP4216 0

KRFP4215 0

KRFP4214 0

KRFP4213 0

KRFP4212 0

KRFP4211 0

KRFP4210 0

KRFP421 0

KRFP4209 0

KRFP4208 0

KRFP4207 0

KRFP4206 0

KRFP4204 0

KRFP4203 0

KRFP4202 0

KRFP4201 0

KRFP4200 0

KRFP420 0

KRFP42 0

KRFP4199 0

KRFP4198 0

KRFP4196 0

KRFP4195 0

KRFP4194 0

KRFP4190 0

KRFP419 0

KRFP4189 0

KRFP4187 0

KRFP4186 0

KRFP4184 0

KRFP4182 0

KRFP4181 0

KRFP4180 0

KRFP418 0

KRFP4179 0

KRFP4178 0

KRFP4177 0

KRFP4176 0

KRFP4174 0

KRFP4173 0

KRFP4172 0

KRFP4171 0

KRFP4170 0

KRFP417 0

KRFP4169 0

KRFP4168 0

KRFP4167 0

KRFP4166 0

KRFP4165 0

KRFP4164 0

KRFP4162 0

KRFP4161 0

KRFP4160 0

KRFP416 0

KRFP4159 0

KRFP4158 0

KRFP4157 0

KRFP4156 0

KRFP4155 0

KRFP4154 0

KRFP4153 0

KRFP4152 0

KRFP4151 0

KRFP4150 0

KRFP415 0

KRFP4149 0

KRFP4148 0

KRFP4147 0

KRFP4146 0

KRFP4145 0

KRFP4144 0

KRFP4143 0

KRFP4142 0

KRFP4141 0

KRFP4140 0

KRFP414 0

KRFP4138 0

KRFP4137 0

KRFP4136 0

KRFP4135 0

KRFP4134 0

KRFP4133 0

KRFP4132 0

KRFP4131 0

KRFP4130 0

KRFP4129 0

KRFP4128 0

KRFP4127 0

KRFP4126 0

KRFP4125 0

KRFP4124 0

KRFP4123 0

KRFP4121 0

KRFP4120 0

KRFP412 0

KRFP4119 0

KRFP4118 0

KRFP4115 0

KRFP4114 0

KRFP4112 0

KRFP4111 0

KRFP4110 0

KRFP411 0

KRFP4109 0

KRFP4108 0

KRFP4107 0

KRFP4106 0

KRFP4105 0

KRFP4104 0

KRFP4103 0

KRFP4102 0

KRFP4101 0

KRFP4100 0

KRFP41 0

KRFP4099 0

KRFP4098 0

KRFP4097 0

KRFP4096 0

KRFP4095 0

KRFP4094 0

KRFP4093 0

KRFP4092 0

KRFP4091 0

KRFP4090 0

KRFP4089 0

KRFP4088 0

KRFP4087 0

KRFP4085 0

KRFP4084 0

KRFP4083 0

KRFP4082 0

KRFP4081 0

KRFP4079 0

KRFP4078 0

KRFP4076 0

KRFP4075 0

KRFP4073 0

KRFP4072 0

KRFP4071 0

KRFP4070 0

KRFP407 0

KRFP4069 0

KRFP4068 0

KRFP4067 0

KRFP4066 0

KRFP4065 0

KRFP4064 0

KRFP4063 0

KRFP4062 0

KRFP4061 0

KRFP4060 0

KRFP406 0

KRFP4059 0

KRFP4058 0

KRFP4057 0

KRFP4056 0

KRFP4055 0

KRFP4054 0

KRFP4052 0

KRFP4051 0

KRFP4050 0

KRFP405 0

KRFP4049 0

KRFP4048 0

KRFP4047 0

KRFP4046 0

KRFP4045 0

KRFP4044 0

KRFP4043 0

KRFP4042 0

KRFP4041 0

KRFP4040 0

KRFP404 0

KRFP4039 0

KRFP4038 0

KRFP4037 0

KRFP4036 0

KRFP4035 0

KRFP4034 0

KRFP4033 0

KRFP4031 0

KRFP4030 0

KRFP4029 0

KRFP4028 0

KRFP4027 0

KRFP4026 0

KRFP4025 0

KRFP4024 0

KRFP4021 0

KRFP4020 0

KRFP402 0

KRFP4017 0

KRFP4016 0

KRFP4014 0

KRFP4013 0

KRFP4012 0

KRFP4011 0

KRFP4010 0

KRFP4009 0

KRFP4008 0

KRFP4007 0

KRFP4006 0

KRFP4004 0

KRFP4003 0

KRFP4002 0

KRFP4001 0

KRFP400 0

KRFP40 0

KRFP4 0

KRFP3999 0

KRFP3998 0

KRFP3997 0

KRFP3996 0

KRFP3995 0

KRFP3994 0

KRFP3993 0

KRFP3992 0

KRFP3991 0

KRFP399 0

KRFP3989 0

KRFP3988 0

KRFP3987 0

KRFP3986 0

KRFP3985 0

KRFP3984 0

KRFP3983 0

KRFP3982 0

KRFP3981 0

KRFP3980 0

KRFP3979 0

KRFP3978 0

KRFP3977 0

KRFP3976 0

KRFP3975 0

KRFP3974 0

KRFP3973 0

KRFP3972 0

KRFP3970 0

KRFP3969 0

KRFP3968 0

KRFP3966 0

KRFP3965 0

KRFP3963 0

KRFP3962 0

KRFP3961 0

KRFP3960 0

KRFP396 0

KRFP3958 0

KRFP3955 0

KRFP3954 0

KRFP3953 0

KRFP3952 0

KRFP3951 0

KRFP395 0

KRFP3944 0

KRFP3941 0

KRFP394 0

KRFP3939 0

KRFP3935 0

KRFP3932 0

KRFP3931 0

KRFP3930 0

KRFP3929 0

KRFP3927 0

KRFP3925 0

KRFP3924 0

KRFP3923 0

KRFP3922 0

KRFP3921 0

KRFP3920 0

KRFP392 0

KRFP3919 0

KRFP3918 0

KRFP3917 0

KRFP3916 0

KRFP3913 0

KRFP3912 0

KRFP3911 0

KRFP3910 0

KRFP391 0

KRFP3909 0

KRFP3908 0

KRFP3906 0

KRFP3905 0

KRFP3904 0

KRFP3903 0

KRFP3902 0

KRFP3901 0

KRFP3900 0

KRFP39 0

KRFP3898 0

KRFP3897 0

KRFP3896 0

KRFP3892 0

KRFP3891 0

KRFP3890 0

KRFP389 0

KRFP3886 0

KRFP3885 0

KRFP3883 0

KRFP3880 0

KRFP388 0

KRFP3879 0

KRFP3878 0

KRFP3877 0

KRFP3876 0

KRFP3875 0

KRFP3874 0

KRFP3873 0

KRFP3872 0

KRFP3871 0

KRFP3870 0

KRFP3868 0

KRFP3867 0

KRFP3866 0

KRFP3865 0

KRFP3864 0

KRFP3863 0

KRFP3862 0

KRFP3861 0

KRFP3860 0

KRFP386 0

KRFP3859 0

KRFP3858 0

KRFP3857 0

KRFP3856 0

KRFP3855 0

KRFP3854 0

KRFP3853 0

KRFP3852 0

KRFP3851 0

KRFP3850 0

KRFP385 0

KRFP3849 0

KRFP3848 0

KRFP3847 0

KRFP3846 0

KRFP3845 0

KRFP3844 0

KRFP3843 0

KRFP3842 0

KRFP3841 0

KRFP3840 0

KRFP384 0

KRFP3839 0

KRFP3838 0

KRFP3837 0

KRFP3836 0

KRFP3835 0

KRFP3834 0

KRFP3833 0

KRFP3832 0

KRFP3831 0

KRFP3830 0

KRFP3829 0

KRFP3828 0

KRFP3827 0

KRFP3826 0

KRFP3825 0

KRFP3824 0

KRFP3823 0

KRFP3822 0

KRFP3819 0

KRFP3818 0

KRFP3817 0

KRFP3812 0

KRFP3811 0

KRFP3810 0

KRFP381 0

KRFP3808 0

KRFP3807 0

KRFP3805 0

KRFP3804 0

KRFP3802 0

KRFP3801 0

KRFP3800 0

KRFP380 0

KRFP38 0

KRFP3799 0

KRFP3798 0

KRFP3797 0

KRFP3796 0

KRFP3792 0

KRFP3791 0

KRFP379 0

KRFP3787 0

KRFP3783 0

KRFP3780 0

KRFP378 0

KRFP3776 0

KRFP3774 0

KRFP3772 0

KRFP3771 0

KRFP3770 0

KRFP377 0

KRFP3769 0

KRFP3766 0

KRFP3765 0

KRFP3764 0

KRFP3763 0

KRFP3762 0

KRFP3761 0

KRFP3760 0

KRFP376 0

KRFP3758 0

KRFP3755 0

KRFP3754 0

KRFP3753 0

KRFP3748 0

KRFP3743 0

KRFP3736 0

KRFP3734 0

KRFP3732 0

KRFP373 0

KRFP3724 0

KRFP3723 0

KRFP3720 0

KRFP372 0

KRFP3717 0

KRFP3715 0

KRFP3714 0

KRFP3711 0

KRFP371 0

KRFP3705 0

KRFP3702 0

KRFP3700 0

KRFP37 0

KRFP3698 0

KRFP3696 0

KRFP3695 0

KRFP3694 0

KRFP3691 0

KRFP3690 0

KRFP369 0

KRFP3689 0

KRFP3688 0

KRFP3687 0

KRFP3686 0

KRFP3685 0

KRFP3684 0

KRFP3679 0

KRFP3678 0

KRFP3677 0

KRFP3676 0

KRFP3673 0

KRFP3672 0

KRFP3670 0

KRFP3669 0

KRFP3666 0

KRFP3664 0

KRFP3653 0

KRFP3652 0

KRFP3651 0

KRFP3650 0

KRFP365 0

KRFP3644 0

KRFP3642 0

KRFP364 0

KRFP3639 0

KRFP3638 0

KRFP3637 0

KRFP3636 0

KRFP3634 0

KRFP3633 0

KRFP3632 0

KRFP3631 0

KRFP3630 0

KRFP3629 0

KRFP3628 0

KRFP3627 0

KRFP3626 0

KRFP3625 0

KRFP3624 0

KRFP3623 0

KRFP3622 0

KRFP3621 0

KRFP3620 0

KRFP3619 0

KRFP3618 0

KRFP3616 0

KRFP3615 0

KRFP3614 0

KRFP3613 0

KRFP3612 0

KRFP3611 0

KRFP3610 0

KRFP361 0

KRFP3607 0

KRFP3605 0

KRFP3604 0

KRFP3603 0

KRFP3601 0

KRFP3600 0

KRFP360 0

KRFP36 0

KRFP3599 0

KRFP3597 0

KRFP3595 0

KRFP3592 0

KRFP3590 0

KRFP3589 0

KRFP3588 0

KRFP3587 0

KRFP3586 0

KRFP3585 0

KRFP3584 0

KRFP3583 0

KRFP3582 0

KRFP3581 0

KRFP3580 0

KRFP3579 0

KRFP3578 0

KRFP3577 0

KRFP3576 0

KRFP3575 0

KRFP3573 0

KRFP3572 0

KRFP3571 0

KRFP3570 0

KRFP357 0

KRFP3569 0

KRFP3568 0

KRFP3567 0

KRFP3566 0

KRFP3565 0

KRFP3564 0

KRFP3563 0

KRFP3562 0

KRFP356 0

KRFP3558 0

KRFP3557 0

KRFP3556 0

KRFP3555 0

KRFP3553 0

KRFP3552 0

KRFP3551 0

KRFP355 0

KRFP3549 0

KRFP3548 0

KRFP3547 0

KRFP3546 0

KRFP3545 0

KRFP3544 0

KRFP3543 0

KRFP3542 0

KRFP3541 0

KRFP3540 0

KRFP3539 0

KRFP3538 0

KRFP3537 0

KRFP3536 0

KRFP3535 0

KRFP3534 0

KRFP3533 0

KRFP3532 0

KRFP3531 0

KRFP3530 0

KRFP353 0

KRFP3528 0

KRFP3527 0

KRFP3526 0

KRFP3525 0

KRFP3524 0

KRFP3523 0

KRFP3522 0

KRFP3521 0

KRFP3520 0

KRFP3519 0

KRFP3518 0

KRFP3517 0

KRFP3516 0

KRFP3515 0

KRFP3514 0

KRFP3513 0

KRFP3512 0

KRFP3511 0

KRFP3510 0

KRFP351 0

KRFP3509 0

KRFP3508 0

KRFP3507 0

KRFP3506 0

KRFP3505 0

KRFP3504 0

KRFP3503 0

KRFP3502 0

KRFP3501 0

KRFP3500 0

KRFP35 0

KRFP3499 0

KRFP3498 0

KRFP3497 0

KRFP3496 0

KRFP3495 0

KRFP3494 0

KRFP3493 0

KRFP3492 0

KRFP3491 0

KRFP3489 0

KRFP3488 0

KRFP3487 0

KRFP3486 0

KRFP3485 0

KRFP3484 0

KRFP3483 0

KRFP3482 0

KRFP3481 0

KRFP3480 0

KRFP3479 0

KRFP3478 0

KRFP3477 0

KRFP3476 0

KRFP3475 0

KRFP3473 0

KRFP3472 0

KRFP3471 0

KRFP3470 0

KRFP3469 0

KRFP3468 0

KRFP3467 0

KRFP3466 0

KRFP3465 0

KRFP3464 0

KRFP3463 0

KRFP3462 0

KRFP3461 0

KRFP3460 0

KRFP3459 0

KRFP3458 0

KRFP3457 0

KRFP3456 0

KRFP3454 0

KRFP3451 0

KRFP3450 0

KRFP345 0

KRFP3449 0

KRFP3448 0

KRFP3447 0

KRFP3446 0

KRFP3445 0

KRFP3444 0

KRFP3439 0

KRFP3438 0

KRFP3437 0

KRFP3433 0

KRFP3432 0

KRFP3431 0

KRFP3429 0

KRFP3424 0

KRFP3423 0

KRFP3422 0

KRFP3421 0

KRFP3420 0

KRFP3417 0

KRFP3416 0

KRFP3413 0

KRFP3412 0

KRFP3411 0

KRFP3409 0

KRFP3407 0

KRFP3406 0

KRFP3405 0

KRFP3401 0

KRFP340 0

KRFP3397 0

KRFP3391 0

KRFP339 0

KRFP3386 0

KRFP3384 0

KRFP3383 0

KRFP3379 0

KRFP3378 0

KRFP3377 0

KRFP3376 0

KRFP3374 0

KRFP3372 0

KRFP3370 0

KRFP337 0

KRFP3367 0

KRFP3366 0

KRFP3365 0

KRFP3364 0

KRFP3363 0

KRFP3362 0

KRFP336 0

KRFP3359 0

KRFP3358 0

KRFP3357 0

KRFP3356 0

KRFP3355 0

KRFP3354 0

KRFP3352 0

KRFP3350 0

KRFP3349 0

KRFP3348 0

KRFP3347 0

KRFP3346 0

KRFP3345 0

KRFP3344 0

KRFP3343 0

KRFP3342 0

KRFP3341 0

KRFP3340 0

KRFP334 0

KRFP3338 0

KRFP3337 0

KRFP3335 0

KRFP3334 0

KRFP3333 0

KRFP3332 0

KRFP3330 0

KRFP333 0

KRFP3329 0

KRFP3327 0

KRFP3326 0

KRFP3325 0

KRFP3324 0

KRFP3323 0

KRFP3322 0

KRFP3321 0

KRFP3320 0

KRFP3319 0

KRFP3318 0

KRFP3317 0

KRFP3316 0

KRFP3315 0

KRFP3314 0

KRFP3313 0

KRFP3312 0

KRFP3311 0

KRFP3310 0

KRFP3309 0

KRFP3308 0

KRFP3307 0

KRFP3306 0

KRFP3305 0

KRFP3304 0

KRFP3303 0

KRFP3302 0

KRFP3301 0

KRFP3300 0

KRFP330 0

KRFP3299 0

KRFP3298 0

KRFP3297 0

KRFP3296 0

KRFP3294 0

KRFP3292 0

KRFP3290 0

KRFP329 0

KRFP3289 0

KRFP3288 0

KRFP3287 0

KRFP3286 0

KRFP3285 0

KRFP3282 0

KRFP3281 0

KRFP3280 0

KRFP328 0

KRFP3279 0

KRFP3278 0

KRFP3277 0

KRFP3276 0

KRFP3275 0

KRFP3274 0

KRFP3273 0

KRFP3272 0

KRFP3271 0

KRFP3270 0

KRFP327 0

KRFP3269 0

KRFP3267 0

KRFP3266 0

KRFP3265 0

KRFP3264 0

KRFP3263 0

KRFP3262 0

KRFP3261 0

KRFP3260 0

KRFP3259 0

KRFP3258 0

KRFP3257 0

KRFP3256 0

KRFP3255 0

KRFP3254 0

KRFP3253 0

KRFP3252 0

KRFP3251 0

KRFP3250 0

KRFP3249 0

KRFP3248 0

KRFP3247 0

KRFP3246 0

KRFP3245 0

KRFP3244 0

KRFP3243 0

KRFP3242 0

KRFP3241 0

KRFP3240 0

KRFP324 0

KRFP3239 0

KRFP3238 0

KRFP3237 0

KRFP3236 0

KRFP3235 0

KRFP3234 0

KRFP3233 0

KRFP3232 0

KRFP3231 0

KRFP3230 0

KRFP323 0

KRFP3227 0

KRFP3226 0

KRFP3222 0

KRFP3221 0

KRFP3220 0

KRFP322 0

KRFP3219 0

KRFP3218 0

KRFP3217 0

KRFP3216 0

KRFP3215 0

KRFP3214 0

KRFP3213 0

KRFP3212 0

KRFP3211 0

KRFP3210 0

KRFP321 0

KRFP3209 0

KRFP3208 0

KRFP3207 0

KRFP3205 0

KRFP3204 0

KRFP3203 0

KRFP3202 0

KRFP3201 0

KRFP320 0

KRFP32 0

KRFP3199 0

KRFP3198 0

KRFP3197 0

KRFP3196 0

KRFP3195 0

KRFP3194 0

KRFP3193 0

KRFP3192 0

KRFP3191 0

KRFP3190 0

KRFP319 0

KRFP3189 0

KRFP3188 0

KRFP3187 0

KRFP3186 0

KRFP3185 0

KRFP3184 0

KRFP3183 0

KRFP3182 0

KRFP3181 0

KRFP318 0

KRFP3178 0

KRFP3177 0

KRFP3176 0

KRFP3175 0

KRFP3174 0

KRFP3173 0

KRFP3172 0

KRFP3171 0

KRFP3170 0

KRFP317 0

KRFP3169 0

KRFP3168 0

KRFP3167 0

KRFP3166 0

KRFP3165 0

KRFP3164 0

KRFP3163 0

KRFP3162 0

KRFP3161 0

KRFP316 0

KRFP3158 0

KRFP3155 0

KRFP3154 0

KRFP3153 0

KRFP3148 0

KRFP3147 0

KRFP3146 0

KRFP3145 0

KRFP3144 0

KRFP3143 0

KRFP3142 0

KRFP3141 0

KRFP3140 0

KRFP314 0

KRFP3138 0

KRFP3136 0

KRFP3135 0

KRFP3134 0

KRFP3133 0

KRFP3132 0

KRFP3131 0

KRFP3130 0

KRFP313 0

KRFP3129 0

KRFP3128 0

KRFP3127 0

KRFP3126 0

KRFP3125 0

KRFP3124 0

KRFP3123 0

KRFP3122 0

KRFP3119 0

KRFP3118 0

KRFP3117 0

KRFP3116 0

KRFP3115 0

KRFP3114 0

KRFP3113 0

KRFP3112 0

KRFP3111 0

KRFP3110 0

KRFP3109 0

KRFP3108 0

KRFP3107 0

KRFP3106 0

KRFP3105 0

KRFP3103 0

KRFP3102 0

KRFP3101 0

KRFP3100 0

KRFP31 0

KRFP3099 0

KRFP3098 0

KRFP3097 0

KRFP3096 0

KRFP3095 0

KRFP3094 0

KRFP3093 0

KRFP3092 0

KRFP3091 0

KRFP3090 0

KRFP309 0

KRFP3089 0

KRFP3088 0

KRFP3087 0

KRFP3086 0

KRFP3085 0

KRFP3084 0

KRFP3083 0

KRFP3082 0

KRFP3080 0

KRFP308 0

KRFP3079 0

KRFP3077 0

KRFP3076 0

KRFP3075 0

KRFP3073 0

KRFP3072 0

KRFP3071 0

KRFP3070 0

KRFP3069 0

KRFP3068 0

KRFP3067 0

KRFP3066 0

KRFP3065 0

KRFP3064 0

KRFP3063 0

KRFP3062 0

KRFP3061 0

KRFP3060 0

KRFP306 0

KRFP3059 0

KRFP3057 0

KRFP3055 0

KRFP3053 0

KRFP3052 0

KRFP3051 0

KRFP3050 0

KRFP305 0

KRFP3049 0

KRFP3048 0

KRFP3047 0

KRFP3046 0

KRFP3045 0

KRFP3044 0

KRFP3043 0

KRFP3042 0

KRFP3041 0

KRFP3040 0

KRFP3039 0

KRFP3038 0

KRFP3037 0

KRFP3036 0

KRFP3035 0

KRFP3033 0

KRFP3032 0

KRFP3031 0

KRFP3030 0

KRFP3029 0

KRFP3028 0

KRFP3027 0

KRFP3026 0

KRFP3024 0

KRFP3023 0

KRFP3022 0

KRFP3021 0

KRFP3020 0

KRFP3019 0

KRFP3018 0

KRFP3017 0

KRFP3016 0

KRFP3014 0

KRFP3011 0

KRFP301 0

KRFP3009 0

KRFP3008 0

KRFP3007 0

KRFP3006 0

KRFP3005 0

KRFP3004 0

KRFP3003 0

KRFP3002 0

KRFP3001 0

KRFP3000 0

KRFP30 0

KRFP3 0

KRFP2999 0

KRFP2998 0

KRFP2997 0

KRFP2996 0

KRFP2995 0

KRFP2994 0

KRFP2993 0

KRFP2992 0

KRFP2991 0

KRFP2990 0

KRFP2989 0

KRFP2988 0

KRFP2987 0

KRFP2985 0

KRFP2983 0

KRFP2982 0

KRFP2981 0

KRFP2980 0

KRFP2979 0

KRFP2978 0

KRFP2974 0

KRFP2973 0

KRFP2972 0

KRFP2971 0

KRFP2970 0

KRFP2969 0

KRFP2968 0

KRFP2967 0

KRFP2966 0

KRFP2965 0

KRFP2964 0

KRFP2963 0

KRFP2962 0

KRFP2961 0

KRFP2960 0

KRFP296 0

KRFP2959 0

KRFP2958 0

KRFP2957 0

KRFP2956 0

KRFP2955 0

KRFP2954 0

KRFP2953 0

KRFP2952 0

KRFP2951 0

KRFP295 0

KRFP2948 0

KRFP2947 0

KRFP2946 0

KRFP2945 0

KRFP2944 0

KRFP2943 0

KRFP2942 0

KRFP2941 0

KRFP2940 0

KRFP294 0

KRFP2939 0

KRFP2938 0

KRFP2937 0

KRFP2936 0

KRFP2935 0

KRFP2934 0

KRFP2933 0

KRFP2932 0

KRFP2931 0

KRFP2930 0

KRFP293 0

KRFP2929 0

KRFP2928 0

KRFP2927 0

KRFP2926 0

KRFP2925 0

KRFP2924 0

KRFP2923 0

KRFP2922 0

KRFP2921 0

KRFP2920 0

KRFP292 0

KRFP2919 0

KRFP2918 0

KRFP2917 0

KRFP2916 0

KRFP2915 0

KRFP2914 0

KRFP2913 0

KRFP2912 0

KRFP2911 0

KRFP2910 0

KRFP2909 0

KRFP2908 0

KRFP2907 0

KRFP2906 0

KRFP2905 0

KRFP2904 0

KRFP2903 0

KRFP2902 0

KRFP2901 0

KRFP2900 0

KRFP29 0

KRFP2899 0

KRFP2898 0

KRFP2897 0

KRFP2896 0

KRFP2895 0

KRFP2894 0

KRFP2893 0

KRFP2892 0

KRFP2891 0

KRFP2890 0

KRFP289 0

KRFP2889 0

KRFP2888 0

KRFP2887 0

KRFP2885 0

KRFP2884 0

KRFP2883 0

KRFP2881 0

KRFP2880 0

KRFP288 0

KRFP2879 0

KRFP2878 0

KRFP2877 0

KRFP2875 0

KRFP2874 0

KRFP2873 0

KRFP2872 0

KRFP2871 0

KRFP2869 0

KRFP2868 0

KRFP2867 0

KRFP2866 0

KRFP2865 0

KRFP2864 0

KRFP2863 0

KRFP2862 0

KRFP2861 0

KRFP2860 0

KRFP286 0

KRFP2859 0

KRFP2858 0

KRFP2857 0

KRFP2854 0

KRFP2853 0

KRFP2852 0

KRFP2851 0

KRFP2850 0

KRFP285 0

KRFP2849 0

KRFP2848 0

KRFP2847 0

KRFP2846 0

KRFP2845 0

KRFP2844 0

KRFP2843 0

KRFP2842 0

KRFP2841 0

KRFP2840 0

KRFP284 0

KRFP2839 0

KRFP2838 0

KRFP2837 0

KRFP2836 0

KRFP2835 0

KRFP2834 0

KRFP2833 0

KRFP2832 0

KRFP2831 0

KRFP2830 0

KRFP283 0

KRFP2829 0

KRFP2828 0

KRFP2827 0

KRFP2826 0

KRFP2825 0

KRFP2824 0

KRFP2823 0

KRFP2822 0

KRFP2821 0

KRFP2820 0

KRFP282 0

KRFP2819 0

KRFP2818 0

KRFP2817 0

KRFP2815 0

KRFP2814 0

KRFP2813 0

KRFP2812 0

KRFP2811 0

KRFP2810 0

KRFP281 0

KRFP2809 0

KRFP2808 0

KRFP2807 0

KRFP2806 0

KRFP2805 0

KRFP2804 0

KRFP2803 0

KRFP2802 0

KRFP2801 0

KRFP2800 0

KRFP280 0

KRFP28 0

KRFP2799 0

KRFP2798 0

KRFP2797 0

KRFP2796 0

KRFP2795 0

KRFP2794 0

KRFP2793 0

KRFP2792 0

KRFP2791 0

KRFP2790 0

KRFP279 0

KRFP2789 0

KRFP2788 0

KRFP2787 0

KRFP2786 0

KRFP2785 0

KRFP2784 0

KRFP2783 0

KRFP2782 0

KRFP2780 0

KRFP278 0

KRFP2779 0

KRFP2778 0

KRFP2776 0

KRFP2775 0

KRFP2774 0

KRFP2771 0

KRFP2770 0

KRFP277 0

KRFP2769 0

KRFP2768 0

KRFP2767 0

KRFP2766 0

KRFP2765 0

KRFP2764 0

KRFP2763 0

KRFP2762 0

KRFP2761 0

KRFP2760 0

KRFP276 0

KRFP2759 0

KRFP2758 0

KRFP2757 0

KRFP2756 0

KRFP2755 0

KRFP2754 0

KRFP2753 0

KRFP2752 0

KRFP2751 0

KRFP2750 0

KRFP275 0

KRFP2749 0

KRFP2748 0

KRFP2747 0

KRFP2746 0

KRFP2745 0

KRFP2744 0

KRFP2743 0

KRFP2742 0

KRFP2741 0

KRFP2740 0

KRFP274 0

KRFP2739 0

KRFP2738 0

KRFP2737 0

KRFP2736 0

KRFP2735 0

KRFP2734 0

KRFP2733 0

KRFP2732 0

KRFP2731 0

KRFP2730 0

KRFP273 0

KRFP2729 0

KRFP2728 0

KRFP2727 0

KRFP2726 0

KRFP2725 0

KRFP2724 0

KRFP2723 0

KRFP2722 0

KRFP2721 0

KRFP2720 0

KRFP272 0

KRFP2719 0

KRFP2718 0

KRFP2717 0

KRFP2716 0

KRFP2715 0

KRFP2714 0

KRFP2713 0

KRFP2712 0

KRFP2711 0

KRFP2710 0

KRFP271 0

KRFP2709 0

KRFP2708 0

KRFP2707 0

KRFP2706 0

KRFP2705 0

KRFP2704 0

KRFP2703 0

KRFP2702 0

KRFP2701 0

KRFP2700 0

KRFP270 0

KRFP27 0

KRFP2699 0

KRFP2698 0

KRFP2697 0

KRFP2696 0

KRFP2693 0

KRFP2692 0

KRFP2691 0

KRFP2690 0

KRFP269 0

KRFP2689 0

KRFP2688 0

KRFP2687 0

KRFP2686 0

KRFP2685 0

KRFP2684 0

KRFP2681 0

KRFP2680 0

KRFP268 0

KRFP2679 0

KRFP2678 0

KRFP2677 0

KRFP2676 0

KRFP2674 0

KRFP2672 0

KRFP2671 0

KRFP2670 0

KRFP267 0

KRFP2669 0

KRFP2666 0

KRFP2665 0

KRFP2664 0

KRFP2663 0

KRFP2662 0

KRFP2661 0

KRFP2660 0

KRFP266 0

KRFP2659 0

KRFP2658 0

KRFP2657 0

KRFP2656 0

KRFP2655 0

KRFP2654 0

KRFP2653 0

KRFP2652 0

KRFP2650 0

KRFP265 0

KRFP2649 0

KRFP2648 0

KRFP2647 0

KRFP2646 0

KRFP2645 0

KRFP2644 0

KRFP2643 0

KRFP2642 0

KRFP2641 0

KRFP2640 0

KRFP264 0

KRFP2639 0

KRFP2638 0

KRFP2637 0

KRFP2636 0

KRFP2635 0

KRFP2634 0

KRFP2633 0

KRFP2632 0

KRFP2631 0

KRFP2630 0

KRFP263 0

KRFP2629 0

KRFP2628 0

KRFP2627 0

KRFP2626 0

KRFP2625 0

KRFP2624 0

KRFP2623 0

KRFP2622 0

KRFP2621 0

KRFP2620 0

KRFP262 0

KRFP2619 0

KRFP2618 0

KRFP2617 0

KRFP2616 0

KRFP2615 0

KRFP2614 0

KRFP2613 0

KRFP2612 0

KRFP2611 0

KRFP2610 0

KRFP261 0

KRFP2609 0

KRFP2608 0

KRFP2607 0

KRFP2606 0

KRFP2605 0

KRFP2604 0

KRFP2603 0

KRFP2602 0

KRFP2601 0

KRFP2600 0

KRFP260 0

KRFP26 0

KRFP2599 0

KRFP2597 0

KRFP2596 0

KRFP2594 0

KRFP2593 0

KRFP2592 0

KRFP2591 0

KRFP2590 0

KRFP259 0

KRFP2589 0

KRFP2588 0

KRFP2586 0

KRFP2585 0

KRFP2584 0

KRFP2583 0

KRFP2582 0

KRFP2581 0

KRFP2580 0

KRFP258 0

KRFP2579 0

KRFP2578 0

KRFP2577 0

KRFP2576 0

KRFP2575 0

KRFP2574 0

KRFP2573 0

KRFP2572 0

KRFP2571 0

KRFP2570 0

KRFP257 0

KRFP2569 0

KRFP2568 0

KRFP2567 0

KRFP2566 0

KRFP2565 0

KRFP2563 0

KRFP2562 0

KRFP2561 0

KRFP2560 0

KRFP256 0

KRFP2559 0

KRFP2558 0

KRFP2557 0

KRFP2556 0

KRFP2555 0

KRFP2554 0

KRFP2553 0

KRFP2552 0

KRFP2551 0

KRFP2550 0

KRFP255 0

KRFP2549 0

KRFP2546 0

KRFP2545 0

KRFP2544 0

KRFP2543 0

KRFP2542 0

KRFP2541 0

KRFP2540 0

KRFP254 0

KRFP2539 0

KRFP2538 0

KRFP2537 0

KRFP2536 0

KRFP2535 0

KRFP2534 0

KRFP2533 0

KRFP2532 0

KRFP2531 0

KRFP2530 0

KRFP253 0

KRFP2529 0

KRFP2528 0

KRFP2527 0

KRFP2526 0

KRFP2525 0

KRFP2524 0

KRFP2523 0

KRFP2522 0

KRFP2521 0

KRFP2520 0

KRFP252 0

KRFP2519 0

KRFP2518 0

KRFP2517 0

KRFP2516 0

KRFP2515 0

KRFP2514 0

KRFP2513 0

KRFP2512 0

KRFP2511 0

KRFP2510 0

KRFP251 0

KRFP2509 0

KRFP2508 0

KRFP2507 0

KRFP2506 0

KRFP2505 0

KRFP2504 0

KRFP2503 0

KRFP2502 0

KRFP2501 0

KRFP2500 0

KRFP250 0

KRFP2499 0

KRFP2498 0

KRFP2497 0

KRFP2496 0

KRFP2495 0

KRFP2494 0

KRFP2493 0

KRFP2492 0

KRFP2491 0

KRFP2490 0

KRFP249 0

KRFP2489 0

KRFP2488 0

KRFP2487 0

KRFP2486 0

KRFP2485 0

KRFP2484 0

KRFP2483 0

KRFP2482 0

KRFP2481 0

KRFP2480 0

KRFP248 0

KRFP2479 0

KRFP2478 0

KRFP2477 0

KRFP2476 0

KRFP2475 0

KRFP2474 0

KRFP2473 0

KRFP2472 0

KRFP2471 0

KRFP2470 0

KRFP247 0

KRFP2469 0

KRFP2468 0

KRFP2467 0

KRFP2466 0

KRFP2465 0

KRFP2464 0

KRFP2463 0

KRFP2462 0

KRFP2461 0

KRFP2460 0

KRFP246 0

KRFP2459 0

KRFP2458 0

KRFP2457 0

KRFP2456 0

KRFP2455 0

KRFP2454 0

KRFP2453 0

KRFP2452 0

KRFP2451 0

KRFP2450 0

KRFP245 0

KRFP2449 0

KRFP2448 0

KRFP2447 0

KRFP2446 0

KRFP2445 0

KRFP2444 0

KRFP2443 0

KRFP2442 0

KRFP2441 0

KRFP2440 0

KRFP244 0

KRFP2439 0

KRFP2438 0

KRFP2437 0

KRFP2436 0

KRFP2435 0

KRFP2434 0

KRFP2433 0

KRFP2432 0

KRFP2431 0

KRFP2430 0

KRFP243 0

KRFP2429 0

KRFP2428 0

KRFP2427 0

KRFP2426 0

KRFP2425 0

KRFP2424 0

KRFP2423 0

KRFP2422 0

KRFP2421 0

KRFP2420 0

KRFP242 0

KRFP2419 0

KRFP2418 0

KRFP2417 0

KRFP2416 0

KRFP2415 0

KRFP2414 0

KRFP2413 0

KRFP2412 0

KRFP2411 0

KRFP2410 0

KRFP241 0

KRFP2409 0

KRFP2408 0

KRFP2407 0

KRFP2406 0

KRFP2405 0

KRFP2404 0

KRFP2403 0

KRFP2402 0

KRFP2401 0

KRFP2400 0

KRFP240 0

KRFP24 0

KRFP2399 0

KRFP2398 0

KRFP2397 0

KRFP2396 0

KRFP2395 0

KRFP2394 0

KRFP2393 0

KRFP2392 0

KRFP2391 0

KRFP2390 0

KRFP239 0

KRFP2389 0

KRFP2388 0

KRFP2387 0

KRFP2386 0

KRFP2385 0

KRFP2384 0

KRFP2383 0

KRFP2382 0

KRFP2381 0

KRFP238 0

KRFP2379 0

KRFP2378 0

KRFP2377 0

KRFP2376 0

KRFP2375 0

KRFP2374 0

KRFP2373 0

KRFP2372 0

KRFP2371 0

KRFP2370 0

KRFP237 0

KRFP2369 0

KRFP2368 0

KRFP2367 0

KRFP2366 0

KRFP2365 0

KRFP2364 0

KRFP2363 0

KRFP2362 0

KRFP2361 0

KRFP2360 0

KRFP236 0

KRFP2359 0

KRFP2358 0

KRFP2357 0

KRFP2356 0

KRFP2355 0

KRFP2354 0

KRFP2353 0

KRFP2352 0

KRFP2351 0

KRFP2350 0

KRFP235 0

KRFP2349 0

KRFP2348 0

KRFP2347 0

KRFP2346 0

KRFP2345 0

KRFP2344 0

KRFP2343 0

KRFP2342 0

KRFP2341 0

KRFP2340 0

KRFP234 0

KRFP2339 0

KRFP2338 0

KRFP2337 0

KRFP2336 0

KRFP2335 0

KRFP2334 0

KRFP2333 0

KRFP2332 0

KRFP2331 0

KRFP2330 0

KRFP233 0

KRFP2329 0

KRFP2328 0

KRFP2327 0

KRFP2326 0

KRFP2325 0

KRFP2324 0

KRFP2323 0

KRFP2322 0

KRFP2321 0

KRFP2320 0

KRFP232 0

KRFP2319 0

KRFP2318 0

KRFP2317 0

KRFP2316 0

KRFP2315 0

KRFP2314 0

KRFP2313 0

KRFP2312 0

KRFP2311 0

KRFP2310 0

KRFP231 0

KRFP2309 0

KRFP2307 0

KRFP2305 0

KRFP2303 0

KRFP2302 0

KRFP2301 0

KRFP2300 0

KRFP230 0

KRFP2299 0

KRFP2298 0

KRFP2297 0

KRFP2296 0

KRFP2295 0

KRFP2294 0

KRFP2293 0

KRFP2292 0

KRFP2291 0

KRFP2290 0

KRFP229 0

KRFP2289 0

KRFP2288 0

KRFP2287 0

KRFP2286 0

KRFP2285 0

KRFP2284 0

KRFP2283 0

KRFP2282 0

KRFP2281 0

KRFP2280 0

KRFP228 0

KRFP2279 0

KRFP2278 0

KRFP2277 0

KRFP2276 0

KRFP2275 0

KRFP2274 0

KRFP2273 0

KRFP2272 0

KRFP2271 0

KRFP2270 0

KRFP227 0

KRFP2269 0

KRFP2268 0

KRFP2267 0

KRFP2266 0

KRFP226 0

KRFP2258 0

KRFP2257 0

KRFP2256 0

KRFP2255 0

KRFP2254 0

KRFP2253 0

KRFP2252 0

KRFP2251 0

KRFP2250 0

KRFP225 0

KRFP2249 0

KRFP2248 0

KRFP2247 0

KRFP2246 0

KRFP2245 0

KRFP2244 0

KRFP2243 0

KRFP2242 0

KRFP2241 0

KRFP2240 0

KRFP224 0

KRFP2239 0

KRFP2238 0

KRFP2237 0

KRFP2236 0

KRFP2235 0

KRFP2234 0

KRFP2233 0

KRFP2232 0

KRFP2231 0

KRFP2230 0

KRFP223 0

KRFP2229 0

KRFP2228 0

KRFP2227 0

KRFP2226 0

KRFP2225 0

KRFP2224 0

KRFP2223 0

KRFP2222 0

KRFP2221 0

KRFP2220 0

KRFP222 0

KRFP2219 0

KRFP2218 0

KRFP2217 0

KRFP2216 0

KRFP2215 0

KRFP2214 0

KRFP2213 0

KRFP2212 0

KRFP2211 0

KRFP2210 0

KRFP221 0

KRFP2209 0

KRFP2208 0

KRFP2207 0

KRFP2206 0

KRFP2205 0

KRFP2204 0

KRFP2203 0

KRFP2202 0

KRFP2201 0

KRFP2200 0

KRFP220 0

KRFP22 0

KRFP2199 0

KRFP2198 0

KRFP2197 0

KRFP2196 0

KRFP2195 0

KRFP2194 0

KRFP2193 0

KRFP2192 0

KRFP2191 0

KRFP2190 0

KRFP219 0

KRFP2189 0

KRFP2188 0

KRFP2187 0

KRFP2186 0

KRFP2185 0

KRFP2184 0

KRFP2183 0

KRFP2182 0

KRFP2181 0

KRFP2180 0

KRFP218 0

KRFP2179 0

KRFP2178 0

KRFP2177 0

KRFP2176 0

KRFP2175 0

KRFP2174 0

KRFP2173 0

KRFP2172 0

KRFP2171 0

KRFP2170 0

KRFP217 0

KRFP2169 0

KRFP2168 0

KRFP2167 0

KRFP2166 0

KRFP2165 0

KRFP2164 0

KRFP2163 0

KRFP2162 0

KRFP2161 0

KRFP2160 0

KRFP216 0

KRFP2159 0

KRFP2158 0

KRFP2157 0

KRFP2156 0

KRFP2155 0

KRFP2154 0

KRFP2153 0

KRFP2152 0

KRFP2151 0

KRFP2150 0

KRFP215 0

KRFP2148 0

KRFP2147 0

KRFP2146 0

KRFP2145 0

KRFP2144 0

KRFP2143 0

KRFP2142 0

KRFP2141 0

KRFP2140 0

KRFP214 0

KRFP2139 0

KRFP2138 0

KRFP2136 0

KRFP2134 0

KRFP2133 0

KRFP2132 0

KRFP2131 0

KRFP2130 0

KRFP213 0

KRFP2129 0

KRFP2128 0

KRFP2127 0

KRFP2126 0

KRFP2125 0

KRFP2124 0

KRFP2123 0

KRFP2122 0

KRFP2121 0

KRFP2120 0

KRFP212 0

KRFP2119 0

KRFP2118 0

KRFP2117 0

KRFP2116 0

KRFP2115 0

KRFP2114 0

KRFP2113 0

KRFP2112 0

KRFP2111 0

KRFP2110 0

KRFP211 0

KRFP2109 0

KRFP2108 0

KRFP2107 0

KRFP2106 0

KRFP2105 0

KRFP2104 0

KRFP2103 0

KRFP2102 0

KRFP2101 0

KRFP2100 0

KRFP210 0

KRFP21 0

KRFP2099 0

KRFP2098 0

KRFP2097 0

KRFP2096 0

KRFP2095 0

KRFP2094 0

KRFP2093 0

KRFP2092 0

KRFP2091 0

KRFP2090 0

KRFP209 0

KRFP2089 0

KRFP2088 0

KRFP2087 0

KRFP2086 0

KRFP2085 0

KRFP2084 0

KRFP2083 0

KRFP2082 0

KRFP2081 0

KRFP2080 0

KRFP208 0

KRFP2079 0

KRFP2078 0

KRFP2077 0

KRFP2076 0

KRFP2075 0

KRFP2074 0

KRFP2073 0

KRFP2072 0

KRFP2071 0

KRFP2070 0

KRFP207 0

KRFP2069 0

KRFP2068 0

KRFP2067 0

KRFP2066 0

KRFP2065 0

KRFP2064 0

KRFP2063 0

KRFP2062 0

KRFP2061 0

KRFP2060 0

KRFP206 0

KRFP2059 0

KRFP2058 0

KRFP2057 0

KRFP2056 0

KRFP2054 0

KRFP2053 0

KRFP2052 0

KRFP2051 0

KRFP2050 0

KRFP205 0

KRFP2049 0

KRFP2048 0

KRFP2047 0

KRFP2046 0

KRFP2045 0

KRFP2044 0

KRFP2043 0

KRFP2042 0

KRFP2041 0

KRFP2040 0

KRFP204 0

KRFP2039 0

KRFP2038 0

KRFP2037 0

KRFP2036 0

KRFP2035 0

KRFP2034 0

KRFP2033 0

KRFP2032 0

KRFP2031 0

KRFP2030 0

KRFP203 0

KRFP2029 0

KRFP2028 0

KRFP2027 0

KRFP2026 0

KRFP2025 0

KRFP2024 0

KRFP2023 0

KRFP2022 0

KRFP2021 0

KRFP2020 0

KRFP202 0

KRFP2019 0

KRFP2018 0

KRFP2017 0

KRFP2016 0

KRFP2015 0

KRFP2014 0

KRFP2013 0

KRFP2012 0

KRFP2011 0

KRFP2010 0

KRFP201 0

KRFP2009 0

KRFP2008 0

KRFP2007 0

KRFP2006 0

KRFP2005 0

KRFP2004 0

KRFP2003 0

KRFP2002 0

KRFP2001 0

KRFP2000 0

KRFP200 0

KRFP1999 0

KRFP1998 0

KRFP1997 0

KRFP1996 0

KRFP1995 0

KRFP1994 0

KRFP1993 0

KRFP1992 0

KRFP1991 0

KRFP1990 0

KRFP199 0

KRFP1989 0

KRFP1988 0

KRFP1987 0

KRFP1986 0

KRFP1985 0

KRFP1984 0

KRFP1983 0

KRFP1982 0

KRFP1981 0

KRFP1980 0

KRFP198 0

KRFP1979 0

KRFP1978 0

KRFP1977 0

KRFP1976 0

KRFP1975 0

KRFP1974 0

KRFP1973 0

KRFP1972 0

KRFP1971 0

KRFP1970 0

KRFP197 0

KRFP1969 0

KRFP1968 0

KRFP1967 0

KRFP1966 0

KRFP1965 0

KRFP1964 0

KRFP1963 0

KRFP1962 0

KRFP1961 0

KRFP1960 0

KRFP196 0

KRFP1959 0

KRFP1958 0

KRFP1957 0

KRFP1956 0

KRFP1955 0

KRFP1954 0

KRFP1953 0

KRFP1952 0

KRFP1951 0

KRFP1950 0

KRFP195 0

KRFP1949 0

KRFP1948 0

KRFP1947 0

KRFP1946 0

KRFP1945 0

KRFP1944 0

KRFP1943 0

KRFP1942 0

KRFP1941 0

KRFP1940 0

KRFP1939 0

KRFP1938 0

KRFP1937 0

KRFP1936 0

KRFP1935 0

KRFP1934 0

KRFP1932 0

KRFP1930 0

KRFP193 0

KRFP1929 0

KRFP1928 0

KRFP1927 0

KRFP1926 0

KRFP1925 0

KRFP1924 0

KRFP1921 0

KRFP192 0

KRFP1919 0

KRFP1918 0

KRFP1917 0

KRFP1916 0

KRFP1915 0

KRFP1914 0

KRFP1913 0

KRFP1912 0

KRFP1911 0

KRFP191 0

KRFP1909 0

KRFP1908 0

KRFP1907 0

KRFP1906 0

KRFP1905 0

KRFP1904 0

KRFP1903 0

KRFP1902 0

KRFP1901 0

KRFP1900 0

KRFP1899 0

KRFP1898 0

KRFP1897 0

KRFP1896 0

KRFP1895 0

KRFP1894 0

KRFP1893 0

KRFP1892 0

KRFP1891 0

KRFP1890 0

KRFP1889 0

KRFP1888 0

KRFP1887 0

KRFP1886 0

KRFP1885 0

KRFP1884 0

KRFP1883 0

KRFP1882 0

KRFP1881 0

KRFP1880 0

KRFP188 0

KRFP1879 0

KRFP1878 0

KRFP1877 0

KRFP1876 0

KRFP1875 0

KRFP1874 0

KRFP1873 0

KRFP1872 0

KRFP1871 0

KRFP1870 0

KRFP187 0

KRFP1869 0

KRFP1868 0

KRFP1867 0

KRFP1866 0

KRFP1865 0

KRFP1864 0

KRFP1863 0

KRFP1862 0

KRFP1861 0

KRFP1860 0

KRFP186 0

KRFP1859 0

KRFP1858 0

KRFP1857 0

KRFP1856 0

KRFP1855 0

KRFP1854 0

KRFP1853 0

KRFP1852 0

KRFP1851 0

KRFP1850 0

KRFP185 0

KRFP1849 0

KRFP1848 0

KRFP1847 0

KRFP1846 0

KRFP1845 0

KRFP1844 0

KRFP1843 0

KRFP1842 0

KRFP1841 0

KRFP1840 0

KRFP184 0

KRFP1839 0

KRFP1838 0

KRFP1837 0

KRFP1836 0

KRFP1835 0

KRFP1834 0

KRFP1833 0

KRFP1832 0

KRFP1831 0

KRFP1830 0

KRFP183 0

KRFP1829 0

KRFP1828 0

KRFP1827 0

KRFP1826 0

KRFP1825 0

KRFP1823 0

KRFP1822 0

KRFP1821 0

KRFP1820 0

KRFP182 0

KRFP1819 0

KRFP1818 0

KRFP1817 0

KRFP1816 0

KRFP1815 0

KRFP1814 0

KRFP1813 0

KRFP1812 0

KRFP1811 0

KRFP1810 0

KRFP181 0

KRFP1809 0

KRFP1808 0

KRFP1807 0

KRFP1806 0

KRFP1805 0

KRFP1804 0

KRFP1803 0

KRFP1802 0

KRFP1801 0

KRFP1800 0

KRFP180 0

KRFP1799 0

KRFP1798 0

KRFP1797 0

KRFP1796 0

KRFP1795 0

KRFP1794 0

KRFP1793 0

KRFP1792 0

KRFP1791 0

KRFP1790 0

KRFP179 0

KRFP1789 0

KRFP1788 0

KRFP1787 0

KRFP1786 0

KRFP1785 0

KRFP1784 0

KRFP1783 0

KRFP1782 0

KRFP1781 0

KRFP1780 0

KRFP178 0

KRFP1779 0

KRFP1778 0

KRFP1777 0

KRFP1776 0

KRFP1775 0

KRFP1774 0

KRFP1772 0

KRFP1770 0

KRFP177 0

KRFP1768 0

KRFP1767 0

KRFP1766 0

KRFP1765 0

KRFP1764 0

KRFP1763 0

KRFP1762 0

KRFP1761 0

KRFP1760 0

KRFP176 0

KRFP1759 0

KRFP1758 0

KRFP1757 0

KRFP1756 0

KRFP1755 0

KRFP1754 0

KRFP1753 0

KRFP1752 0

KRFP1751 0

KRFP1750 0

KRFP175 0

KRFP1749 0

KRFP1748 0

KRFP1747 0

KRFP1746 0

KRFP1745 0

KRFP1744 0

KRFP1743 0

KRFP1742 0

KRFP1741 0

KRFP1740 0

KRFP174 0

KRFP1739 0

KRFP1738 0

KRFP1737 0

KRFP1736 0

KRFP1735 0

KRFP1734 0

KRFP1733 0

KRFP1732 0

KRFP1731 0

KRFP1730 0

KRFP173 0

KRFP1729 0

KRFP1728 0

KRFP1727 0

KRFP1726 0

KRFP1725 0

KRFP1723 0

KRFP1722 0

KRFP1721 0

KRFP1720 0

KRFP172 0

KRFP1719 0

KRFP1718 0

KRFP1717 0

KRFP1716 0

KRFP1715 0

KRFP1714 0

KRFP1713 0

KRFP1712 0

KRFP1711 0

KRFP1710 0

KRFP171 0

KRFP1709 0

KRFP1708 0

KRFP1707 0

KRFP1706 0

KRFP1705 0

KRFP1704 0

KRFP1703 0

KRFP1702 0

KRFP1701 0

KRFP1700 0

KRFP170 0

KRFP1699 0

KRFP1698 0

KRFP1697 0

KRFP1696 0

KRFP1695 0

KRFP1694 0

KRFP1693 0

KRFP1692 0

KRFP1691 0

KRFP1690 0

KRFP169 0

KRFP1689 0

KRFP1688 0

KRFP1687 0

KRFP1686 0

KRFP1685 0

KRFP1684 0

KRFP1683 0

KRFP1682 0

KRFP1681 0

KRFP1680 0

KRFP168 0

KRFP1679 0

KRFP1678 0

KRFP1677 0

KRFP1676 0

KRFP1675 0

KRFP1674 0

KRFP1673 0

KRFP1672 0

KRFP1671 0

KRFP1670 0

KRFP167 0

KRFP1669 0

KRFP1668 0

KRFP1667 0

KRFP1666 0

KRFP1665 0

KRFP1664 0

KRFP1663 0

KRFP1662 0

KRFP1661 0

KRFP1660 0

KRFP166 0

KRFP1659 0

KRFP1658 0

KRFP1657 0

KRFP1656 0

KRFP1655 0

KRFP1654 0

KRFP1652 0

KRFP1651 0

KRFP1650 0

KRFP165 0

KRFP1649 0

KRFP1648 0

KRFP1647 0

KRFP1646 0

KRFP1644 0

KRFP1643 0

KRFP1641 0

KRFP1640 0

KRFP164 0

KRFP1639 0

KRFP1638 0

KRFP1637 0

KRFP1636 0

KRFP1635 0

KRFP1632 0

KRFP1631 0

KRFP1630 0

KRFP163 0

KRFP1629 0

KRFP1628 0

KRFP1627 0

KRFP1626 0

KRFP1625 0

KRFP1624 0

KRFP1623 0

KRFP1622 0

KRFP1621 0

KRFP1620 0

KRFP162 0

KRFP1619 0

KRFP1618 0

KRFP1617 0

KRFP1616 0

KRFP1615 0

KRFP1614 0

KRFP1613 0

KRFP1612 0

KRFP1611 0

KRFP1610 0

KRFP1609 0

KRFP1608 0

KRFP1607 0

KRFP1606 0

KRFP1605 0

KRFP1604 0

KRFP1603 0

KRFP1602 0

KRFP1601 0

KRFP1600 0

KRFP160 0

KRFP1599 0

KRFP1598 0

KRFP1597 0

KRFP1596 0

KRFP1595 0

KRFP1594 0

KRFP1593 0

KRFP1591 0

KRFP1590 0

KRFP159 0

KRFP1589 0

KRFP1588 0

KRFP1587 0

KRFP1586 0

KRFP1585 0

KRFP1584 0

KRFP1583 0

KRFP1582 0

KRFP1581 0

KRFP1580 0

KRFP158 0

KRFP1579 0

KRFP1578 0

KRFP1577 0

KRFP1576 0

KRFP1575 0

KRFP1574 0

KRFP1573 0

KRFP1572 0

KRFP1571 0

KRFP1570 0

KRFP157 0

KRFP1569 0

KRFP1568 0

KRFP1567 0

KRFP1565 0

KRFP1563 0

KRFP1562 0

KRFP1561 0

KRFP1560 0

KRFP156 0

KRFP1559 0

KRFP1558 0

KRFP1557 0

KRFP1556 0

KRFP1555 0

KRFP1554 0

KRFP1553 0

KRFP1552 0

KRFP1551 0

KRFP1550 0

KRFP155 0

KRFP1549 0

KRFP1548 0

KRFP1547 0

KRFP1546 0

KRFP1545 0

KRFP1544 0

KRFP1543 0

KRFP1542 0

KRFP1541 0

KRFP1540 0

KRFP154 0

KRFP1539 0

KRFP1538 0

KRFP1537 0

KRFP1535 0

KRFP1534 0

KRFP1533 0

KRFP1532 0

KRFP1531 0

KRFP1530 0

KRFP153 0

KRFP1529 0

KRFP1528 0

KRFP1527 0

KRFP1526 0

KRFP1525 0

KRFP1523 0

KRFP1522 0

KRFP1521 0

KRFP1520 0

KRFP152 0

KRFP1519 0

KRFP1518 0

KRFP1517 0

KRFP1516 0

KRFP1515 0

KRFP1514 0

KRFP1513 0

KRFP1512 0

KRFP1511 0

KRFP1510 0

KRFP151 0

KRFP1509 0

KRFP1508 0

KRFP1507 0

KRFP1506 0

KRFP1505 0

KRFP1504 0

KRFP1503 0

KRFP1502 0

KRFP1501 0

KRFP150 0

KRFP15 0

KRFP1499 0

KRFP1498 0

KRFP1497 0

KRFP1496 0

KRFP1495 0

KRFP1494 0

KRFP1493 0

KRFP1492 0

KRFP1491 0

KRFP1490 0

KRFP149 0

KRFP1489 0

KRFP1488 0

KRFP1487 0

KRFP1486 0

KRFP1485 0

KRFP1484 0

KRFP1483 0

KRFP1482 0

KRFP1481 0

KRFP1480 0

KRFP148 0

KRFP1479 0

KRFP1478 0

KRFP1477 0

KRFP1476 0

KRFP1475 0

KRFP1474 0

KRFP1473 0

KRFP1472 0

KRFP1471 0

KRFP1470 0

KRFP147 0

KRFP1469 0

KRFP1468 0

KRFP1467 0

KRFP1466 0

KRFP1465 0

KRFP1464 0

KRFP1463 0

KRFP1462 0

KRFP1461 0

KRFP1460 0

KRFP146 0

KRFP1459 0

KRFP1458 0

KRFP1457 0

KRFP1456 0

KRFP1455 0

KRFP1454 0

KRFP1453 0

KRFP1451 0

KRFP1450 0

KRFP145 0

KRFP1449 0

KRFP1447 0

KRFP1446 0

KRFP1445 0

KRFP1444 0

KRFP1443 0

KRFP1442 0

KRFP1441 0

KRFP1440 0

KRFP144 0

KRFP1439 0

KRFP1438 0

KRFP1437 0

KRFP1436 0

KRFP1435 0

KRFP1434 0

KRFP1433 0

KRFP1431 0

KRFP1430 0

KRFP143 0

KRFP1429 0

KRFP1428 0

KRFP1425 0

KRFP1424 0

KRFP1423 0

KRFP1422 0

KRFP1421 0

KRFP1420 0

KRFP142 0

KRFP1419 0

KRFP1417 0

KRFP1416 0

KRFP1415 0

KRFP1414 0

KRFP1413 0

KRFP1412 0

KRFP1411 0

KRFP1410 0

KRFP141 0

KRFP1409 0

KRFP1404 0

KRFP1403 0

KRFP1402 0

KRFP1401 0

KRFP1400 0

KRFP140 0

KRFP1399 0

KRFP1398 0

KRFP1397 0

KRFP1396 0

KRFP1395 0

KRFP1394 0

KRFP1393 0

KRFP1392 0

KRFP1391 0

KRFP1390 0

KRFP139 0

KRFP1389 0

KRFP1388 0

KRFP1387 0

KRFP1386 0

KRFP1385 0

KRFP1384 0

KRFP1383 0

KRFP1382 0

KRFP1381 0

KRFP1380 0

KRFP1379 0

KRFP1378 0

KRFP1377 0

KRFP1376 0

KRFP1375 0

KRFP1374 0

KRFP1373 0

KRFP1372 0

KRFP1371 0

KRFP1370 0

KRFP137 0

KRFP1369 0

KRFP1368 0

KRFP1367 0

KRFP1366 0

KRFP1365 0

KRFP1364 0

KRFP1363 0

KRFP1362 0

KRFP1361 0

KRFP1360 0

KRFP1359 0

KRFP1358 0

KRFP1357 0

KRFP1356 0

KRFP1355 0

KRFP1354 0

KRFP1353 0

KRFP1352 0

KRFP1351 0

KRFP1350 0

KRFP135 0

KRFP1349 0

KRFP1348 0

KRFP1347 0

KRFP1346 0

KRFP1345 0

KRFP1344 0

KRFP1343 0

KRFP1342 0

KRFP1341 0

KRFP1340 0

KRFP134 0

KRFP1339 0

KRFP1338 0

KRFP1337 0

KRFP1336 0

KRFP1335 0

KRFP1334 0

KRFP1333 0

KRFP1332 0

KRFP1331 0

KRFP1330 0

KRFP1329 0

KRFP1328 0

KRFP1327 0

KRFP1326 0

KRFP1325 0

KRFP1324 0

KRFP1323 0

KRFP1322 0

KRFP1321 0

KRFP1320 0

KRFP132 0

KRFP1319 0

KRFP1318 0

KRFP1317 0

KRFP1316 0

KRFP1315 0

KRFP1314 0

KRFP1313 0

KRFP1312 0

KRFP1311 0

KRFP1310 0

KRFP131 0

KRFP1309 0

KRFP1308 0

KRFP1307 0

KRFP1306 0

KRFP1305 0

KRFP1304 0

KRFP1303 0

KRFP1302 0

KRFP1301 0

KRFP1300 0

KRFP130 0

KRFP13 0

KRFP1298 0

KRFP1297 0

KRFP1296 0

KRFP1295 0

KRFP1294 0

KRFP1293 0

KRFP1292 0

KRFP1291 0

KRFP1290 0

KRFP129 0

KRFP1289 0

KRFP1288 0

KRFP1287 0

KRFP1286 0

KRFP1285 0

KRFP1284 0

KRFP1283 0

KRFP1282 0

KRFP1281 0

KRFP1280 0

KRFP128 0

KRFP1279 0

KRFP1278 0

KRFP1277 0

KRFP1276 0

KRFP1275 0

KRFP1274 0

KRFP1273 0

KRFP1272 0

KRFP1271 0

KRFP1270 0

KRFP127 0

KRFP1269 0

KRFP1268 0

KRFP1267 0

KRFP1266 0

KRFP1265 0

KRFP1264 0

KRFP1262 0

KRFP1261 0

KRFP1260 0

KRFP1259 0

KRFP1258 0

KRFP1257 0

KRFP1256 0

KRFP1255 0

KRFP1254 0

KRFP1253 0

KRFP1252 0

KRFP1251 0

KRFP125 0

KRFP1249 0

KRFP1248 0

KRFP1247 0

KRFP1246 0

KRFP1244 0

KRFP1243 0

KRFP1242 0

KRFP124 0

KRFP1239 0

KRFP1238 0

KRFP1237 0

KRFP1236 0

KRFP1235 0

KRFP1234 0

KRFP1233 0

KRFP1232 0

KRFP1231 0

KRFP1230 0

KRFP123 0

KRFP1227 0

KRFP1226 0

KRFP1225 0

KRFP1224 0

KRFP1223 0

KRFP1221 0

KRFP1220 0

KRFP122 0

KRFP1219 0

KRFP1218 0

KRFP1217 0

KRFP1216 0

KRFP1215 0

KRFP1214 0

KRFP1213 0

KRFP1211 0

KRFP1210 0

KRFP121 0

KRFP1209 0

KRFP1208 0

KRFP1207 0

KRFP1206 0

KRFP1205 0

KRFP1204 0

KRFP1203 0

KRFP1202 0

KRFP1201 0

KRFP1200 0

KRFP120 0

KRFP12 0

KRFP1199 0

KRFP1198 0

KRFP1197 0

KRFP1196 0

KRFP1194 0

KRFP1192 0

KRFP1191 0

KRFP1190 0

KRFP119 0

KRFP1189 0

KRFP1188 0

KRFP1187 0

KRFP1186 0

KRFP1185 0

KRFP1183 0

KRFP1182 0

KRFP1181 0

KRFP1180 0

KRFP118 0

KRFP1179 0

KRFP1178 0

KRFP1177 0

KRFP1176 0

KRFP1175 0

KRFP1174 0

KRFP1172 0

KRFP1171 0

KRFP1170 0

KRFP117 0

KRFP1169 0

KRFP1168 0

KRFP1167 0

KRFP1166 0

KRFP1164 0

KRFP1163 0

KRFP1162 0

KRFP116 0

KRFP1159 0

KRFP1158 0

KRFP1152 0

KRFP1145 0

KRFP1144 0

KRFP1143 0

KRFP1142 0

KRFP1141 0

KRFP1140 0

KRFP114 0

KRFP1139 0

KRFP1138 0

KRFP1137 0

KRFP1136 0

KRFP1135 0

KRFP1134 0

KRFP1133 0

KRFP1132 0

KRFP1131 0

KRFP1130 0

KRFP113 0

KRFP1129 0

KRFP1128 0

KRFP1127 0

KRFP1126 0

KRFP1125 0

KRFP1124 0

KRFP1123 0

KRFP1122 0

KRFP1121 0

KRFP1120 0

KRFP112 0

KRFP1119 0

KRFP1118 0

KRFP1117 0

KRFP1116 0

KRFP1115 0

KRFP1114 0

KRFP1113 0

KRFP1112 0

KRFP1111 0

KRFP1110 0

KRFP1109 0

KRFP1108 0

KRFP1107 0

KRFP1106 0

KRFP1105 0

KRFP1104 0

KRFP1103 0

KRFP1102 0

KRFP1101 0

KRFP1100 0

KRFP110 0

KRFP11 0

KRFP1099 0

KRFP1098 0

KRFP1097 0

KRFP1096 0

KRFP1095 0

KRFP1094 0

KRFP1093 0

KRFP1092 0

KRFP1091 0

KRFP1090 0

KRFP109 0

KRFP1089 0

KRFP1088 0

KRFP1087 0

KRFP1086 0

KRFP1085 0

KRFP1084 0

KRFP1083 0

KRFP1082 0

KRFP1081 0

KRFP1080 0

KRFP108 0

KRFP1079 0

KRFP1078 0

KRFP1077 0

KRFP1076 0

KRFP1075 0

KRFP1074 0

KRFP1073 0

KRFP1072 0

KRFP1071 0

KRFP1070 0

KRFP107 0

KRFP1069 0

KRFP1068 0

KRFP1067 0

KRFP1066 0

KRFP1065 0

KRFP1064 0

KRFP1063 0

KRFP1062 0

KRFP1061 0

KRFP1060 0

KRFP106 0

KRFP1059 0

KRFP1058 0

KRFP1057 0

KRFP1056 0

KRFP1055 0

KRFP1054 0

KRFP1053 0

KRFP1052 0

KRFP1051 0

KRFP1050 0

KRFP105 0

KRFP1049 0

KRFP1048 0

KRFP1047 0

KRFP1046 0

KRFP1045 0

KRFP1044 0

KRFP1043 0

KRFP1042 0

KRFP1041 0

KRFP1040 0

KRFP1039 0

KRFP1038 0

KRFP1037 0

KRFP1036 0

KRFP1035 0

KRFP1034 0

KRFP1033 0

KRFP1032 0

KRFP1031 0

KRFP1030 0

KRFP103 0

KRFP1029 0

KRFP1028 0

KRFP1027 0

KRFP1026 0

KRFP1025 0

KRFP1024 0

KRFP1023 0

KRFP1022 0

KRFP1020 0

KRFP102 0

KRFP1019 0

KRFP1018 0

KRFP1017 0

KRFP1016 0

KRFP1015 0

KRFP1014 0

KRFP1012 0

KRFP1011 0

KRFP1010 0

KRFP101 0

KRFP1009 0

KRFP1008 0

KRFP1007 0

KRFP1006 0

KRFP1005 0

KRFP1004 0

KRFP1003 0

KRFP1002 0

KRFP1001 0

KRFP1000 0

KRFP100 0

GraphFP990 0

GraphFP959 0

GraphFP95 0

GraphFP936 0

GraphFP933 0

GraphFP923 0

GraphFP918 0

GraphFP916 0

GraphFP877 0

GraphFP869 0

GraphFP868 0

GraphFP862 0

GraphFP861 0

GraphFP86 0

GraphFP85 0

GraphFP84 0

GraphFP82 0

GraphFP81 0

GraphFP807 0

GraphFP735 0

GraphFP720 0

GraphFP70 0

GraphFP669 0

GraphFP658 0

GraphFP655 0

GraphFP654 0

GraphFP65 0

GraphFP648 0

GraphFP643 0

GraphFP633 0

GraphFP628 0

GraphFP627 0

GraphFP626 0

GraphFP624 0

GraphFP621 0

GraphFP597 0

GraphFP585 0

GraphFP580 0

GraphFP577 0

GraphFP56 0

GraphFP55 0

GraphFP53 0

GraphFP527 0

GraphFP510 0

GraphFP507 0

GraphFP5 0

GraphFP494 0

GraphFP470 0

GraphFP468 0

GraphFP467 0

GraphFP459 0

GraphFP452 0

GraphFP445 0

GraphFP429 0

GraphFP426 0

GraphFP425 0

GraphFP416 0

GraphFP407 0

GraphFP406 0

GraphFP403 0

GraphFP395 0

GraphFP392 0

GraphFP391 0

GraphFP378 0

GraphFP371 0

GraphFP364 0

GraphFP360 0

GraphFP351 0

GraphFP350 0

GraphFP347 0

GraphFP346 0

GraphFP314 0

GraphFP312 0

GraphFP310 0

GraphFP271 0

GraphFP241 0

GraphFP236 0

GraphFP201 0

GraphFP2 0

GraphFP176 0

GraphFP171 0

GraphFP165 0

GraphFP161 0

GraphFP159 0

GraphFP141 0

GraphFP132 0

GraphFP126 0

GraphFP119 0

GraphFP111 0

GraphFP107 0

GraphFP106 0

GraphFP1021 0

EStateFP79 0

EStateFP78 0

EStateFP77 0

EStateFP76 0

EStateFP74 0

EStateFP73 0

EStateFP72 0

EStateFP71 0

EStateFP69 0

EStateFP68 0

EStateFP67 0

EStateFP66 0

EStateFP65 0

EStateFP63 0

EStateFP61 0

EStateFP60 0

EStateFP6 0

EStateFP59 0

EStateFP58 0

EStateFP57 0

EStateFP56 0

EStateFP55 0

EStateFP5 0

EStateFP47 0

EStateFP45 0

EStateFP44 0

EStateFP43 0

EStateFP42 0

EStateFP41 0

EStateFP40 0

EStateFP4 0

EStateFP39 0

EStateFP3 0

EStateFP27 0

EStateFP22 0

EStateFP2 0

EStateFP1 0

APC2D9_X_X 0

APC2D9_S_X 0

APC2D9_S_Si 0

APC2D9_S_S 0

APC2D9_S_P 0

APC2D9_Si_X 0

APC2D9_Si_Si 0

APC2D9_S_I 0

APC2D9_S_F 0

APC2D9_S_Cl 0

APC2D9_S_Br 0

APC2D9_S_B 0

APC2D9_P_X 0

APC2D9_P_Si 0

APC2D9_P_P 0

APC2D9_P_I 0

APC2D9_P_F 0

APC2D9_P_Cl 0

APC2D9_P_Br 0

APC2D9_P_B 0

APC2D9_O_Si 0

APC2D9_O_S 0

APC2D9_O_I 0

APC2D9_O_F 0

APC2D9_O_Cl 0

APC2D9_O_Br 0

APC2D9_O_B 0

APC2D9_N_X 0

APC2D9_N_Si 0

APC2D9_N_I 0

APC2D9_N_F 0

APC2D9_N_Cl 0

APC2D9_N_Br 0

APC2D9_N_B 0

APC2D9_I_X 0

APC2D9_I_Si 0

APC2D9_I_I 0

APC2D9_I_B 0

APC2D9_F_X 0

APC2D9_F_Si 0

APC2D9_F_I 0

APC2D9_F_F 0

APC2D9_F_Cl 0

APC2D9_F_Br 0

APC2D9_F_B 0

APC2D9_C_Si 0

APC2D9_Cl_X 0

APC2D9_Cl_Si 0

APC2D9_Cl_I 0

APC2D9_Cl_Cl 0

APC2D9_Cl_Br 0

APC2D9_Cl_B 0

APC2D9_C_F 0

APC2D9_C_Cl 0

APC2D9_C_Br 0

APC2D9_C_B 0

APC2D9_B_X 0

APC2D9_B_Si 0

APC2D9_Br_X 0

APC2D9_Br_Si 0

APC2D9_Br_I 0

APC2D9_Br_Br 0

APC2D9_Br_B 0

APC2D9_B_B 0

APC2D8_X_X 0

APC2D8_S_X 0

APC2D8_S_Si 0

APC2D8_S_S 0

APC2D8_S_P 0

APC2D8_Si_X 0

APC2D8_Si_Si 0

APC2D8_S_I 0

APC2D8_S_F 0

APC2D8_S_Cl 0

APC2D8_S_Br 0

APC2D8_S_B 0

APC2D8_P_X 0

APC2D8_P_Si 0

APC2D8_P_I 0

APC2D8_P_F 0

APC2D8_P_Cl 0

APC2D8_P_Br 0

APC2D8_P_B 0

APC2D8_O_Si 0

APC2D8_O_Br 0

APC2D8_O_B 0

APC2D8_N_X 0

APC2D8_N_Si 0

APC2D8_N_I 0

APC2D8_N_F 0

APC2D8_N_Cl 0

APC2D8_N_Br 0

APC2D8_N_B 0

APC2D8_I_X 0

APC2D8_I_Si 0

APC2D8_I_I 0

APC2D8_I_B 0

APC2D8_F_X 0

APC2D8_F_Si 0

APC2D8_F_I 0

APC2D8_F_F 0

APC2D8_F_Cl 0

APC2D8_F_Br 0

APC2D8_F_B 0

APC2D8_C_Si 0

APC2D8_Cl_Si 0

APC2D8_Cl_I 0

APC2D8_Cl_Cl 0

APC2D8_Cl_Br 0

APC2D8_Cl_B 0

APC2D8_C_Br 0

APC2D8_C_B 0

APC2D8_B_X 0

APC2D8_B_Si 0

APC2D8_Br_X 0

APC2D8_Br_Si 0

APC2D8_Br_I 0

APC2D8_Br_Br 0

APC2D8_Br_B 0

APC2D8_B_B 0

APC2D7_S_X 0

APC2D7_S_Si 0

APC2D7_S_S 0

APC2D7_Si_X 0

APC2D7_Si_Si 0

APC2D7_S_I 0

APC2D7_S_F 0

APC2D7_S_Cl 0

APC2D7_S_Br 0

APC2D7_S_B 0

APC2D7_P_X 0

APC2D7_P_Si 0

APC2D7_P_I 0

APC2D7_P_F 0

APC2D7_P_Cl 0

APC2D7_P_Br 0

APC2D7_P_B 0

APC2D7_O_Si 0

APC2D7_O_Cl 0

APC2D7_O_Br 0

APC2D7_O_B 0

APC2D7_N_X 0

APC2D7_N_Si 0

APC2D7_N_I 0

APC2D7_N_F 0

APC2D7_N_Cl 0

APC2D7_N_Br 0

APC2D7_N_B 0

APC2D7_I_Si 0

APC2D7_I_B 0

APC2D7_F_X 0

APC2D7_F_Si 0

APC2D7_F_I 0

APC2D7_F_F 0

APC2D7_F_Cl 0

APC2D7_F_Br 0

APC2D7_F_B 0

APC2D7_C_Si 0

APC2D7_Cl_X 0

APC2D7_Cl_Si 0

APC2D7_Cl_I 0

APC2D7_Cl_Cl 0

APC2D7_Cl_Br 0

APC2D7_Cl_B 0

APC2D7_C_B 0

APC2D7_B_X 0

APC2D7_B_Si 0

APC2D7_Br_X 0

APC2D7_Br_Si 0

APC2D7_Br_I 0

APC2D7_Br_Br 0

APC2D7_Br_B 0

APC2D7_B_B 0

APC2D6_X_X 0

APC2D6_S_X 0

APC2D6_S_Si 0

APC2D6_S_S 0

APC2D6_S_P 0

APC2D6_Si_X 0

APC2D6_Si_Si 0

APC2D6_S_I 0

APC2D6_S_F 0

APC2D6_S_Cl 0

APC2D6_S_Br 0

APC2D6_S_B 0

APC2D6_P_X 0

APC2D6_P_Si 0

APC2D6_P_I 0

APC2D6_P_F 0

APC2D6_P_Cl 0

APC2D6_P_Br 0

APC2D6_P_B 0

APC2D6_O_Si 0

APC2D6_O_I 0

APC2D6_O_Cl 0

APC2D6_O_Br 0

APC2D6_O_B 0

APC2D6_N_Si 0

APC2D6_N_F 0

APC2D6_N_Cl 0

APC2D6_N_Br 0

APC2D6_N_B 0

APC2D6_I_X 0

APC2D6_I_Si 0

APC2D6_I_I 0

APC2D6_I_B 0

APC2D6_F_X 0

APC2D6_F_Si 0

APC2D6_F_I 0

APC2D6_F_F 0

APC2D6_F_Cl 0

APC2D6_F_Br 0

APC2D6_F_B 0

APC2D6_C_Si 0

APC2D6_Cl_X 0

APC2D6_Cl_Si 0

APC2D6_Cl_I 0

APC2D6_Cl_Cl 0

APC2D6_Cl_Br 0

APC2D6_Cl_B 0

APC2D6_C_B 0

APC2D6_B_X 0

APC2D6_B_Si 0

APC2D6_Br_X 0

APC2D6_Br_Si 0

APC2D6_Br_I 0

APC2D6_Br_Br 0

APC2D6_Br_B 0

APC2D6_B_B 0

APC2D5_X_X 0

APC2D5_S_X 0

APC2D5_S_Si 0

APC2D5_S_S 0

APC2D5_S_P 0

APC2D5_Si_X 0

APC2D5_Si_Si 0

APC2D5_S_I 0

APC2D5_S_F 0

APC2D5_S_Cl 0

APC2D5_S_Br 0

APC2D5_S_B 0

APC2D5_P_X 0

APC2D5_P_Si 0

APC2D5_P_P 0

APC2D5_P_I 0

APC2D5_P_F 0

APC2D5_P_Cl 0

APC2D5_P_Br 0

APC2D5_P_B 0

APC2D5_O_Si 0

APC2D5_O_I 0

APC2D5_O_F 0

APC2D5_O_B 0

APC2D5_N_Si 0

APC2D5_N_I 0

APC2D5_N_Br 0

APC2D5_N_B 0

APC2D5_I_X 0

APC2D5_I_Si 0

APC2D5_I_I 0

APC2D5_I_B 0

APC2D5_F_X 0

APC2D5_F_Si 0

APC2D5_F_I 0

APC2D5_F_F 0

APC2D5_F_Cl 0

APC2D5_F_Br 0

APC2D5_F_B 0

APC2D5_C_Si 0

APC2D5_Cl_X 0

APC2D5_Cl_Si 0

APC2D5_Cl_I 0

APC2D5_Cl_Cl 0

APC2D5_Cl_Br 0

APC2D5_Cl_B 0

APC2D5_C_Br 0

APC2D5_C_B 0

APC2D5_B_X 0

APC2D5_B_Si 0

APC2D5_Br_X 0

APC2D5_Br_Si 0

APC2D5_Br_I 0

APC2D5_Br_Br 0

APC2D5_Br_B 0

APC2D5_B_B 0

APC2D4_S_X 0

APC2D4_S_Si 0

APC2D4_S_S 0

APC2D4_S_P 0

APC2D4_Si_X 0

APC2D4_Si_Si 0

APC2D4_S_I 0

APC2D4_S_F 0

APC2D4_S_Cl 0

APC2D4_S_Br 0

APC2D4_S_B 0

APC2D4_P_Si 0

APC2D4_P_I 0

APC2D4_P_F 0

APC2D4_P_Cl 0

APC2D4_P_Br 0

APC2D4_P_B 0

APC2D4_O_Si 0

APC2D4_O_F 0

APC2D4_O_Cl 0

APC2D4_O_B 0

APC2D4_N_X 0

APC2D4_N_Si 0

APC2D4_N_I 0

APC2D4_N_Cl 0

APC2D4_N_Br 0

APC2D4_N_B 0

APC2D4_I_Si 0

APC2D4_I_B 0

APC2D4_F_X 0

APC2D4_F_Si 0

APC2D4_F_I 0

APC2D4_F_F 0

APC2D4_F_Cl 0

APC2D4_F_Br 0

APC2D4_F_B 0

APC2D4_C_Si 0

APC2D4_Cl_Si 0

APC2D4_Cl_I 0

APC2D4_Cl_Br 0

APC2D4_Cl_B 0

APC2D4_C_B 0

APC2D4_B_X 0

APC2D4_B_Si 0

APC2D4_Br_Si 0

APC2D4_Br_I 0

APC2D4_Br_Br 0

APC2D4_Br_B 0

APC2D4_B_B 0

APC2D3_S_X 0

APC2D3_S_Si 0

APC2D3_S_S 0

APC2D3_S_P 0

APC2D3_Si_X 0

APC2D3_Si_Si 0

APC2D3_S_I 0

APC2D3_S_F 0

APC2D3_S_Cl 0

APC2D3_S_Br 0

APC2D3_S_B 0

APC2D3_P_X 0

APC2D3_P_Si 0

APC2D3_P_P 0

APC2D3_P_I 0

APC2D3_P_F 0

APC2D3_P_Cl 0

APC2D3_P_Br 0

APC2D3_P_B 0

APC2D3_O_Si 0

APC2D3_O_B 0

APC2D3_N_Si 0

APC2D3_N_I 0

APC2D3_N_F 0

APC2D3_N_Cl 0

APC2D3_N_Br 0

APC2D3_N_B 0

APC2D3_I_X 0

APC2D3_I_Si 0

APC2D3_I_I 0

APC2D3_I_B 0

APC2D3_F_X 0

APC2D3_F_Si 0

APC2D3_F_I 0

APC2D3_F_F 0

APC2D3_F_Cl 0

APC2D3_F_Br 0

APC2D3_F_B 0

APC2D3_C_Si 0

APC2D3_Cl_Si 0

APC2D3_Cl_I 0

APC2D3_Cl_Cl 0

APC2D3_Cl_Br 0

APC2D3_Cl_B 0

APC2D3_C_I 0

APC2D3_C_B 0

APC2D3_B_X 0

APC2D3_B_Si 0

APC2D3_Br_X 0

APC2D3_Br_Si 0

APC2D3_Br_I 0

APC2D3_Br_Br 0

APC2D3_Br_B 0

APC2D3_B_B 0

APC2D2_S_X 0

APC2D2_S_Si 0

APC2D2_S_S 0

APC2D2_S_P 0

APC2D2_Si_X 0

APC2D2_Si_Si 0

APC2D2_S_I 0

APC2D2_S_F 0

APC2D2_S_Cl 0

APC2D2_S_Br 0

APC2D2_S_B 0

APC2D2_P_X 0

APC2D2_P_Si 0

APC2D2_P_I 0

APC2D2_P_F 0

APC2D2_P_Cl 0

APC2D2_P_Br 0

APC2D2_P_B 0

APC2D2_O_Si 0

APC2D2_O_P 0

APC2D2_O_I 0

APC2D2_O_F 0

APC2D2_O_Cl 0

APC2D2_O_Br 0

APC2D2_O_B 0

APC2D2_N_Si 0

APC2D2_N_P 0

APC2D2_N_I 0

APC2D2_N_F 0

APC2D2_N_Br 0

APC2D2_N_B 0

APC2D2_I_X 0

APC2D2_I_Si 0

APC2D2_I_I 0

APC2D2_I_B 0

APC2D2_F_Si 0

APC2D2_F_I 0

APC2D2_F_Cl 0

APC2D2_F_Br 0

APC2D2_F_B 0

APC2D2_C_Si 0

APC2D2_Cl_Si 0

APC2D2_Cl_I 0

APC2D2_Cl_Br 0

APC2D2_Cl_B 0

APC2D2_C_B 0

APC2D2_B_X 0

APC2D2_B_Si 0

APC2D2_Br_X 0

APC2D2_Br_Si 0

APC2D2_Br_I 0

APC2D2_Br_Br 0

APC2D2_Br_B 0

APC2D2_B_B 0

APC2D1_X_X 0

APC2D1_S_X 0

APC2D1_S_Si 0

APC2D1_Si_X 0

APC2D1_Si_Si 0

APC2D1_S_I 0

APC2D1_S_F 0

APC2D1_S_Cl 0

APC2D1_S_Br 0

APC2D1_S_B 0

APC2D1_P_X 0

APC2D1_P_Si 0

APC2D1_P_P 0

APC2D1_P_I 0

APC2D1_P_F 0

APC2D1_P_Cl 0

APC2D1_P_Br 0

APC2D1_P_B 0

APC2D1_O_Si 0

APC2D1_O_I 0

APC2D1_O_F 0

APC2D1_O_Br 0

APC2D1_O_B 0

APC2D1_N_X 0

APC2D1_N_Si 0

APC2D1_N_S 0

APC2D1_N_I 0

APC2D1_N_F 0

APC2D1_N_Cl 0

APC2D1_N_Br 0

APC2D1_N_B 0

APC2D1_I_X 0

APC2D1_I_Si 0

APC2D1_I_I 0

APC2D1_I_B 0

APC2D1_F_X 0

APC2D1_F_Si 0

APC2D1_F_I 0

APC2D1_F_F 0

APC2D1_F_Cl 0

APC2D1_F_Br 0

APC2D1_F_B 0

APC2D1_C_Si 0

APC2D1_Cl_X 0

APC2D1_Cl_Si 0

APC2D1_Cl_I 0

APC2D1_Cl_Cl 0

APC2D1_Cl_Br 0

APC2D1_Cl_B 0

APC2D1_C_B 0

APC2D1_B_X 0

APC2D1_B_Si 0

APC2D1_Br_X 0

APC2D1_Br_Si 0

APC2D1_Br_I 0

APC2D1_Br_Br 0

APC2D1_Br_B 0

APC2D1_B_B 0

APC2D10_X_X 0

APC2D10_S_X 0

APC2D10_S_Si 0

APC2D10_S_S 0

APC2D10_S_P 0

APC2D10_Si_X 0

APC2D10_Si_Si 0

APC2D10_S_I 0

APC2D10_S_F 0

APC2D10_S_Cl 0

APC2D10_S_Br 0

APC2D10_S_B 0

APC2D10_P_X 0

APC2D10_P_Si 0

APC2D10_P_P 0

APC2D10_P_I 0

APC2D10_P_F 0

APC2D10_P_Cl 0

APC2D10_P_Br 0

APC2D10_P_B 0

APC2D10_O_X 0

APC2D10_O_Si 0

APC2D10_O_S 0

APC2D10_O_I 0

APC2D10_O_F 0

APC2D10_O_Cl 0

APC2D10_O_Br 0

APC2D10_O_B 0

APC2D10_N_X 0

APC2D10_N_Si 0

APC2D10_N_S 0

APC2D10_N_I 0

APC2D10_N_F 0

APC2D10_N_Cl 0

APC2D10_N_Br 0

APC2D10_N_B 0

APC2D10_I_X 0

APC2D10_I_Si 0

APC2D10_I_I 0

APC2D10_I_B 0

APC2D10_F_X 0

APC2D10_F_Si 0

APC2D10_F_I 0

APC2D10_F_F 0

APC2D10_F_Cl 0

APC2D10_F_Br 0

APC2D10_F_B 0

APC2D10_C_Si 0

APC2D10_Cl_X 0

APC2D10_Cl_Si 0

APC2D10_Cl_I 0

APC2D10_Cl_Cl 0

APC2D10_Cl_Br 0

APC2D10_Cl_B 0

APC2D10_C_I 0

APC2D10_C_F 0

APC2D10_C_Cl 0

APC2D10_C_Br 0

APC2D10_C_B 0

APC2D10_B_X 0

APC2D10_B_Si 0

APC2D10_Br_X 0

APC2D10_Br_Si 0

APC2D10_Br_I 0

APC2D10_Br_Br 0

APC2D10_Br_B 0

APC2D10_B_B 0

AD2D99 0

AD2D98 0

AD2D97 0

AD2D95 0

AD2D94 0

AD2D89 0

AD2D88 0

AD2D780 0

AD2D78 0

AD2D779 0

AD2D778 0

AD2D777 0

AD2D776 0

AD2D775 0

AD2D774 0

AD2D773 0

AD2D772 0

AD2D771 0

AD2D770 0

AD2D77 0

AD2D769 0

AD2D768 0

AD2D767 0

AD2D766 0

AD2D765 0

AD2D764 0

AD2D763 0

AD2D762 0

AD2D761 0

AD2D760 0

AD2D76 0

AD2D759 0

AD2D758 0

AD2D757 0

AD2D756 0

AD2D755 0

AD2D754 0

AD2D753 0

AD2D752 0

AD2D751 0

AD2D750 0

AD2D75 0

AD2D749 0

AD2D748 0

AD2D747 0

AD2D746 0

AD2D745 0

AD2D744 0

AD2D743 0

AD2D742 0

AD2D741 0

AD2D740 0

AD2D74 0

AD2D739 0

AD2D738 0

AD2D737 0

AD2D736 0

AD2D735 0

AD2D734 0

AD2D733 0

AD2D732 0

AD2D731 0

AD2D730 0

AD2D73 0

AD2D729 0

AD2D727 0

AD2D725 0

AD2D724 0

AD2D723 0

AD2D722 0

AD2D721 0

AD2D720 0

AD2D72 0

AD2D719 0

AD2D717 0

AD2D713 0

AD2D712 0

AD2D710 0

AD2D71 0

AD2D709 0

AD2D708 0

AD2D701 0

AD2D700 0

AD2D70 0

AD2D699 0

AD2D698 0

AD2D697 0

AD2D696 0

AD2D695 0

AD2D694 0

AD2D693 0

AD2D692 0

AD2D691 0

AD2D690 0

AD2D69 0

AD2D689 0

AD2D688 0

AD2D687 0

AD2D686 0

AD2D685 0

AD2D684 0

AD2D683 0

AD2D682 0

AD2D681 0

AD2D680 0

AD2D68 0

AD2D679 0

AD2D678 0

AD2D677 0

AD2D676 0

AD2D675 0

AD2D674 0

AD2D673 0

AD2D672 0

AD2D671 0

AD2D670 0

AD2D67 0

AD2D669 0

AD2D668 0

AD2D667 0

AD2D666 0

AD2D665 0

AD2D664 0

AD2D663 0

AD2D662 0

AD2D661 0

AD2D660 0

AD2D66 0

AD2D659 0

AD2D658 0

AD2D656 0

AD2D655 0

AD2D654 0

AD2D653 0

AD2D652 0

AD2D651 0

AD2D650 0

AD2D65 0

AD2D647 0

AD2D646 0

AD2D645 0

AD2D644 0

AD2D643 0

AD2D642 0

AD2D641 0

AD2D64 0

AD2D639 0

AD2D635 0

AD2D634 0

AD2D632 0

AD2D631 0

AD2D630 0

AD2D63 0

AD2D623 0

AD2D622 0

AD2D621 0

AD2D620 0

AD2D62 0

AD2D619 0

AD2D618 0

AD2D617 0

AD2D616 0

AD2D615 0

AD2D614 0

AD2D613 0

AD2D612 0

AD2D611 0

AD2D610 0

AD2D61 0

AD2D609 0

AD2D608 0

AD2D607 0

AD2D606 0

AD2D605 0

AD2D604 0

AD2D603 0

AD2D602 0

AD2D601 0

AD2D600 0

AD2D60 0

AD2D599 0

AD2D598 0

AD2D597 0

AD2D596 0

AD2D595 0

AD2D594 0

AD2D593 0

AD2D592 0

AD2D591 0

AD2D590 0

AD2D59 0

AD2D589 0

AD2D588 0

AD2D587 0

AD2D586 0

AD2D585 0

AD2D584 0

AD2D583 0

AD2D582 0

AD2D581 0

AD2D580 0

AD2D58 0

AD2D578 0

AD2D577 0

AD2D575 0

AD2D57 0

AD2D569 0

AD2D568 0

AD2D567 0

AD2D566 0

AD2D565 0

AD2D564 0

AD2D563 0

AD2D561 0

AD2D56 0

AD2D557 0

AD2D556 0

AD2D554 0

AD2D55 0

AD2D546 0

AD2D545 0

AD2D544 0

AD2D543 0

AD2D542 0

AD2D541 0

AD2D540 0

AD2D54 0

AD2D539 0

AD2D538 0

AD2D536 0

AD2D535 0

AD2D534 0

AD2D533 0

AD2D532 0

AD2D530 0

AD2D53 0

AD2D529 0

AD2D528 0

AD2D527 0

AD2D526 0

AD2D525 0

AD2D524 0

AD2D523 0

AD2D522 0

AD2D521 0

AD2D520 0

AD2D52 0

AD2D519 0

AD2D518 0

AD2D517 0

AD2D516 0

AD2D515 0

AD2D514 0

AD2D513 0

AD2D512 0

AD2D511 0

AD2D510 0

AD2D51 0

AD2D509 0

AD2D508 0

AD2D507 0

AD2D506 0

AD2D505 0

AD2D504 0

AD2D502 0

AD2D500 0

AD2D50 0

AD2D499 0

AD2D497 0

AD2D496 0

AD2D490 0

AD2D49 0

AD2D489 0

AD2D488 0

AD2D487 0

AD2D486 0

AD2D485 0

AD2D48 0

AD2D479 0

AD2D478 0

AD2D476 0

AD2D47 0

AD2D467 0

AD2D466 0

AD2D465 0

AD2D464 0

AD2D463 0

AD2D462 0

AD2D461 0

AD2D460 0

AD2D46 0

AD2D459 0

AD2D458 0

AD2D457 0

AD2D456 0

AD2D455 0

AD2D454 0

AD2D452 0

AD2D451 0

AD2D450 0

AD2D45 0

AD2D449 0

AD2D448 0

AD2D447 0

AD2D446 0

AD2D445 0

AD2D444 0

AD2D443 0

AD2D442 0

AD2D441 0

AD2D440 0

AD2D44 0

AD2D439 0

AD2D438 0

AD2D437 0

AD2D436 0

AD2D435 0

AD2D434 0

AD2D432 0

AD2D431 0

AD2D430 0

AD2D43 0

AD2D429 0

AD2D428 0

AD2D427 0

AD2D426 0

AD2D425 0

AD2D424 0

AD2D422 0

AD2D421 0

AD2D420 0

AD2D42 0

AD2D419 0

AD2D417 0

AD2D412 0

AD2D411 0

AD2D41 0

AD2D409 0

AD2D408 0

AD2D407 0

AD2D405 0

AD2D401 0

AD2D400 0

AD2D40 0

AD2D398 0

AD2D39 0

AD2D389 0

AD2D388 0

AD2D387 0

AD2D386 0

AD2D385 0

AD2D384 0

AD2D383 0

AD2D382 0

AD2D381 0

AD2D380 0

AD2D38 0

AD2D379 0

AD2D378 0

AD2D377 0

AD2D376 0

AD2D375 0

AD2D374 0

AD2D373 0

AD2D372 0

AD2D371 0

AD2D370 0

AD2D37 0

AD2D369 0

AD2D368 0

AD2D367 0

AD2D366 0

AD2D365 0

AD2D364 0

AD2D363 0

AD2D362 0

AD2D361 0

AD2D360 0

AD2D36 0

AD2D359 0

AD2D358 0

AD2D357 0

AD2D356 0

AD2D355 0

AD2D354 0

AD2D353 0

AD2D352 0

AD2D351 0

AD2D350 0

AD2D349 0

AD2D348 0

AD2D347 0

AD2D346 0

AD2D344 0

AD2D343 0

AD2D342 0

AD2D341 0

AD2D339 0

AD2D334 0

AD2D333 0

AD2D332 0

AD2D331 0

AD2D323 0

AD2D322 0

AD2D32 0

AD2D311 0

AD2D310 0

AD2D31 0

AD2D309 0

AD2D308 0

AD2D307 0

AD2D305 0

AD2D304 0

AD2D302 0

AD2D301 0

AD2D300 0

AD2D30 0

AD2D299 0

AD2D298 0

AD2D296 0

AD2D295 0

AD2D294 0

AD2D293 0

AD2D291 0

AD2D290 0

AD2D29 0

AD2D289 0

AD2D288 0

AD2D287 0

AD2D286 0

AD2D285 0

AD2D283 0

AD2D282 0

AD2D281 0

AD2D280 0

AD2D278 0

AD2D276 0

AD2D275 0

AD2D274 0

AD2D273 0

AD2D272 0

AD2D271 0

AD2D270 0

AD2D27 0

AD2D269 0

AD2D266 0

AD2D265 0

AD2D263 0

AD2D261 0

AD2D256 0

AD2D255 0

AD2D254 0

AD2D253 0

AD2D252 0

AD2D245 0

AD2D244 0

AD2D233 0

AD2D232 0

AD2D231 0

AD2D230 0

AD2D23 0

AD2D229 0

AD2D228 0

AD2D227 0

AD2D226 0

AD2D225 0

AD2D224 0

AD2D223 0

AD2D222 0

AD2D221 0

AD2D220 0

AD2D22 0

AD2D219 0

AD2D218 0

AD2D217 0

AD2D216 0

AD2D215 0

AD2D214 0

AD2D212 0

AD2D211 0

AD2D210 0

AD2D21 0

AD2D209 0

AD2D208 0

AD2D207 0

AD2D206 0

AD2D205 0

AD2D204 0

AD2D203 0

AD2D202 0

AD2D201 0

AD2D200 0

AD2D20 0

AD2D199 0

AD2D198 0

AD2D197 0

AD2D196 0

AD2D195 0

AD2D194 0

AD2D193 0

AD2D192 0

AD2D191 0

AD2D190 0

AD2D19 0

AD2D188 0

AD2D187 0

AD2D185 0

AD2D181 0

AD2D18 0

AD2D179 0

AD2D178 0

AD2D177 0

AD2D176 0

AD2D175 0

AD2D173 0

AD2D172 0

AD2D17 0

AD2D167 0

AD2D166 0

AD2D164 0

AD2D16 0

AD2D155 0

AD2D154 0

AD2D153 0

AD2D152 0

AD2D151 0

AD2D150 0

AD2D15 0

AD2D149 0

AD2D148 0

AD2D147 0

AD2D145 0

AD2D144 0

AD2D143 0

AD2D142 0

AD2D140 0

AD2D139 0

AD2D138 0

AD2D137 0

AD2D134 0

AD2D133 0

AD2D132 0

AD2D131 0

AD2D130 0

AD2D128 0

AD2D127 0

AD2D126 0

AD2D125 0

AD2D124 0

AD2D123 0

AD2D122 0

AD2D120 0

AD2D119 0

AD2D118 0

AD2D117 0

AD2D116 0

AD2D115 0

AD2D114 0

AD2D113 0

AD2D112 0

AD2D110 0

AD2D11 0

AD2D109 0

AD2D108 0

AD2D107 0

AD2D106 0

AD2D105 0

AD2D100 0

AD2D10 0

GraphFP181 -0.000390523509674592

PubchemFP576 -0.000406362636248634

GraphFP870 -0.000524024328244255

FP357 -0.00104203421645554

SubFP135 -0.00108535927266961

FP222 -0.00122550041841248

MACCSFP27 -0.00142092678204394

PubchemFP636 -0.00146647259247378

PubchemFP610 -0.00159574825891776

SubFPC10 -0.00184388599975597

FP322 -0.00185112500414947

KRFP4287 -0.00194390159025572

GraphFP182 -0.00211351026580437

GraphFP685 -0.00249036816473511

KRFP1564 -0.0027826555552617

SubFP99 -0.00297876438635811

GraphFP707 -0.00330158327983631

PubchemFP58 -0.00344157489353087

GraphFP792 -0.00383315451030376

APC2D9_O_P -0.00421993505882092

GraphFP829 -0.00482751944867653

KRFP4645 -0.00565752840426994

PubchemFP190 -0.00584107479415377

GraphFP204 -0.00584995940786887

GraphFP993 -0.00601692179957046

GraphFP821 -0.00647327884082863

KRFP4747 -0.00668410972127576

FP865 -0.00739626522277148

KRFP3747 -0.00767680463296267

APC2D7_N_S -0.00828214409032846

GraphFP444 -0.00889288211169201

KRFP2261 -0.0091488484378787

GraphFP937 -0.00916660077989575

KRFP810 -0.00943140275886875

KRFP3602 -0.009789389471673

GraphFP331 -0.0101051602922737

KRFP3683 -0.0113820124766512

APC2D8_O_I -0.0114775884269961

GraphFP697 -0.0117414454474562

PubchemFP586 -0.0118475990347952

AD2D262 -0.0170584834687146

GraphFP602 -0.0462060750122403

FP536 -0.0541840752670902

KRFP3425 -0.0605722588339713

GraphFP899 -0.0634002153354954

FP316 -0.0748077697556136

FP664 -0.0929421654627462

FP507 -0.0993322169932717

APC2D5_C_I -0.104449800590503

KRFP3947 -0.104590012378415

GraphFP713 -0.107857125234018

GraphFP906 -0.129523825915099

PubchemFP622 -0.129533668959103

SubFPC133 -0.133171741116284

GraphFP984 -0.133199581578604

GraphFP594 -0.133583915653509

KRFP566 -0.13507729073978

EStateFP52 -0.139272497961735

KRFP304 -0.141013117020039

PubchemFP163 -0.143396903923243

KRFP1769 -0.153429140121444

PubchemFP694 -0.15626014867926

PubchemFP735 -0.159649200159426

SubFPC170 -0.166767820720306

KRFP3081 -0.172791021214551

SubFP209 -0.182611448930243

KRFP34 -0.188915644494308

KRFP3283 -0.189629805235318

GraphFP184 -0.192895922322266

PubchemFP740 -0.193136422646252

PubchemFP770 -0.193239101794696

GraphFP709 -0.195209832414874

GraphFP511 -0.19521214253031

GraphFP49 -0.19857235859265

PubchemFP147 -0.199814248849728

GraphFP466 -0.200066983420343

KRFP4225 -0.201605799535817

AD2D306 -0.202769093024985

KRFP3726 -0.20279900682271

PubchemFP507 -0.203260099209692

KRFP4005 -0.2036309831753

GraphFP39 -0.204708010818476

APC2D4_C_I -0.205585075312053

GraphFP532 -0.206046639746925

KRFP1157 -0.206948341404821

KRFP768 -0.206958138242458

FP361 -0.224867049024929

AD2D633 -0.249287711059892

GraphFP968 -0.251552492459101

APC2D8_N_S -0.253737668975822

KRFP136 -0.256620535729

AD2D34 -0.256808120486424

KRFP3990 -0.257390432531938

GraphFP135 -0.258914014356393

GraphFP37 -0.259348158642876

APC2D5_O_S -0.260126991114081

AD2D243 -0.262638955348282

APC2D6_N_I -0.263691584215949

PubchemFP408 -0.265662535661141

KRFP1524 -0.268988151360938

FP655 -0.273114090017789

APC2D8_O_S -0.274296043175232

KRFP3680 -0.275783357646771

GraphFP379 -0.276115481269774

KRFP2856 -0.277604583403337

APC2D7_O_S -0.281254041492753

SubFP40 -0.282984231476757

FP859 -0.285729312106606

KRFP3426 -0.286118264454472

SubFPC40 -0.286247805215313

GraphFP854 -0.287616258661637

KRFP4360 -0.291317647339676

KRFP23 -0.304051912398809

SubFPC165 -0.321297625574122

AD2D24 -0.323702002693726

SubFPC126 -0.327264485051641

KRFP3339 -0.346722077267854

KRFP4583 -0.34950199189093

KRFP582 -0.363929432433097

GraphFP535 -0.365560877067406

KRFP4398 -0.365986797882729

PubchemFP130 -0.366274136133454

FP699 -0.36631727805959

SubFPC63 -0.36757242673245

AD2D186 -0.36782538624143

APC2D2_C_I -0.372276704257453

GraphFP344 -0.375949538523156

FP66 -0.378241794359848

FP814 -0.386485190690955

PubchemFP153 -0.387445341836858

PubchemFP687 -0.399577698473414

MACCSFP68 -0.405876481931636

KRFP3561 -0.412395512860939

KRFP3490 -0.422825822498224

GraphFP855 -0.438552791789553

KRFP4659 -0.447881585050756

SubFPC32 -0.449466574975495

SubFPC202 -0.449466574975495

SubFPC201 -0.449466574975495

SubFPC17 -0.449466574975495

SubFP71 -0.449466574975495

SubFP37 -0.449466574975495

SubFP163 -0.449466574975495

PubchemFP806 -0.449466574975495

PubchemFP719 -0.449466574975495

PubchemFP715 -0.449466574975495

PubchemFP670 -0.449466574975495

PubchemFP630 -0.449466574975495

PubchemFP529 -0.449466574975495

PubchemFP475 -0.449466574975495

PubchemFP394 -0.449466574975495

PubchemFP388 -0.449466574975495

KRFP683 -0.449466574975495

KRFP636 -0.449466574975495

KRFP4853 -0.449466574975495

KRFP4824 -0.449466574975495

KRFP4664 -0.449466574975495

KRFP4612 -0.449466574975495

KRFP4602 -0.449466574975495

KRFP4525 -0.449466574975495

KRFP4498 -0.449466574975495

KRFP4183 -0.449466574975495

KRFP3964 -0.449466574975495

KRFP3665 -0.449466574975495

KRFP3663 -0.449466574975495

KRFP3594 -0.449466574975495

KRFP3574 -0.449466574975495

KRFP3415 -0.449466574975495

KRFP3381 -0.449466574975495

KRFP3331 -0.449466574975495

KRFP3206 -0.449466574975495

KRFP3200 -0.449466574975495

KRFP3152 -0.449466574975495

KRFP2777 -0.449466574975495

KRFP2304 -0.449466574975495

KRFP2149 -0.449466574975495

KRFP1923 -0.449466574975495

KRFP1724 -0.449466574975495

KRFP1634 -0.449466574975495

KRFP1229 -0.449466574975495

KRFP1222 -0.449466574975495

KRFP1151 -0.449466574975495

GraphFP972 -0.449466574975495

GraphFP814 -0.449466574975495

GraphFP74 -0.449466574975495

GraphFP679 -0.449466574975495

GraphFP638 -0.449466574975495

GraphFP635 -0.449466574975495

GraphFP574 -0.449466574975495

GraphFP569 -0.449466574975495

GraphFP537 -0.449466574975495

GraphFP513 -0.449466574975495

GraphFP47 -0.449466574975495

GraphFP448 -0.449466574975495

GraphFP442 -0.449466574975495

GraphFP414 -0.449466574975495

GraphFP4 -0.449466574975495

GraphFP365 -0.449466574975495

GraphFP35 -0.449466574975495

GraphFP341 -0.449466574975495

GraphFP337 -0.449466574975495

GraphFP326 -0.449466574975495

GraphFP267 -0.449466574975495

GraphFP248 -0.449466574975495

GraphFP218 -0.449466574975495

GraphFP215 -0.449466574975495

GraphFP192 -0.449466574975495

GraphFP19 -0.449466574975495

GraphFP17 -0.449466574975495

GraphFP158 -0.449466574975495

GraphFP121 -0.449466574975495

GraphFP1016 -0.449466574975495

FP567 -0.449466574975495

EStateFP51 -0.449466574975495

APC2D8_C_Cl -0.449466574975495

APC2D7_C_I -0.449466574975495

APC2D7_C_Br -0.449466574975495

APC2D4_O_I -0.449466574975495

AD2D574 -0.449466574975495

AD2D555 -0.449466574975495

AD2D477 -0.449466574975495

AD2D320 -0.449466574975495

AD2D129 -0.449466574975495

KRFP3371 -0.449724575148962

FP20 -0.451650672517747

GraphFP66 -0.4516737075574

PubchemFP761 -0.457535769566016

GraphFP998 -0.457762124412176

FP446 -0.495621751406753

GraphFP771 -0.506854516203298

KRFP4728 -0.506863960425896

GraphFP143 -0.511437509739073

FP856 -0.521877300141527

KRFP3658 -0.526247926980295

SubFP284 -0.552461281316966

GraphFP32 -0.569917513558415

GraphFP349 -0.570308855282078

KRFP4770 -0.58123835826329

FP200 -0.583394564041479

SubFP165 -0.600215649218918

KRFP4752 -0.601006433456098

GraphFP419 -0.60110651605881

PubchemFP631 -0.602059789036301

KRFP4523 -0.602173794242215

GraphFP127 -0.603219369226308

KRFP2876 -0.60400235241816

MACCSFP15 -0.604093039072709

AD2D399 -0.604336306815676

APC2D4_P_P -0.604339442360618

PubchemFP653 -0.604744592347554

KRFP3361 -0.605251320766813

PubchemFP228 -0.605266866742786

GraphFP742 -0.605727626153008

GraphFP376 -0.606017818123982

KRFP2695 -0.606555863282442

SubFP126 -0.615572937264447

KRFP4708 -0.617394973283038

SubFPC52 -0.619157988449197

KRFP3139 -0.622812209827682

KRFP1773 -0.622909039725242

KRFP2694 -0.635569989652407

PubchemFP494 -0.635574759997632

GraphFP300 -0.635599671495038

KRFP1931 -0.635603608295649

GraphFP657 -0.635611821128554

KRFP1212 -0.635615066514236

KRFP1195 -0.635619983761906

KRFP3121 -0.635623639102978

KRFP4113 -0.635631036772132

KRFP3814 -0.635632741492862

AD2D9 -0.635634988201563

GraphFP30 -0.635635077554659

SubFPC39 -0.63563727541887

KRFP194 -0.635639791721431

GraphFP393 -0.635641671488837

KRFP3559 -0.636129254758978

SubFPC75 -0.636318010730288

FP556 -0.63776946158062

SubFP202 -0.638870934280363

SubFP57 -0.638871099070843

KRFP1228 -0.676339813781038

KRFP3888 -0.683537772159155

PubchemFP714 -0.694478878005931

APC2D6_C_I -0.729420932852982

GraphFP216 -0.733422106932556

KRFP4749 -0.734746159703961

PubchemFP590 -0.736891456824171

KRFP3608 -0.738257743086363

KRFP99 -0.763054532034645

KRFP3554 -0.766029007400499

MACCSFP11 -0.778478820778578

KRFP331 -0.781117675462633

KRFP311 -0.78114166875684

PubchemFP544 -0.806907675839042

SubFPC57 -0.846122350173378

APC2D6_O_S -0.850160649163289

EStateFP14 -0.853394835967108

AD2D576 -0.85425161887126

KRFP1653 -0.85449042090669

KRFP1771 -0.875948651130266

KRFP4661 -0.885831187988451

KRFP332 -0.898932063453314

KRFP1642 -0.901175892107759

KRFP3387 -0.918421340771539

KRFP1426 -0.962999992581501

KRFP335 -1.00704500951626

KRFP2682 -1.10627088084511

KRFP4492 -1.36745051881651
