# Supplementary material for: ToxiM: A Toxicity Prediction Tool for Small Molecules Developed Using Machine Learning and Chemoinformatics Approaches
Source: Front Pharmacol. 2017 Nov 30;8:880. doi: 10.3389/fphar.2017.00880 (PMC5714866; doi:10.3389/fphar.2017.00880)
Supplement: Supplementary file 6 [file Table2.DOCX]

**Supplementary Table S2**. List of the descriptors to select the important ones for the construction of RF based models.

Descriptors MeanDecreaseAccuracy

MolLogP 8.2950985487

PEOE_VSA14 7.5305988112

PEOE_VSA1 7.1551729018

HallKierAlpha 6.9741938198

TPSA 6.9374253611

Kappa2 6.7956827353

BertzCT 6.7522191661

MinEStateIndex 6.6722321887

EState_VSA9 6.5910022247

EState_VSA10 6.5679145866

SlogP_VSA2 6.4799934703

BalabanJ 6.4733276206

ExactMolWt 6.4575772802

PEOE_VSA8 6.3428127973

EState_VSA8 6.3354679638

Chi1n 6.3141425386

SlogP_VSA5 6.2932805658

Chi4n 6.2546289927

MaxAbsEStateIndex 6.2231099846

SlogP_VSA1 6.2124812323

Chi2n 6.1100830955

Chi2v 6.0840003385

Kappa1 6.0805359426

SMR_VSA5 6.0410883916

SlogP_VSA4 6.036644496

PEOE_VSA7 6.0353410478

SMR_VSA10 6.0107281518

Chi0n 5.9664468364

EState_VSA2 5.9538140149

Chi3v 5.8839717488

NHOHCount 5.8533773433

MolWt 5.8356494874

Chi1v 5.8177638642

SMR_VSA4 5.8049836651

MolMR 5.794034388

NumRotatableBonds 5.7878831469

Chi1 5.7861127177

PEOE_VSA9 5.7631139078

SMR_VSA1 5.7464174641

fr_quatN 5.7328236406

PEOE_VSA10 5.7103252978

HeavyAtomMolWt 5.6888988754

NumHeteroatoms 5.55181222

MaxEStateIndex 5.5138251396

Chi3n 5.5040418381

NumHAcceptors 5.4966418447

Chi0 5.4715453247

NumHDonors 5.437462898

VSA_EState9 5.4204748899

SMR_VSA6 5.3688587374

fr_phos_acid 5.3605738616

NumValenceElectrons 5.353333513

LabuteASA 5.3478384701

Kappa3 5.3448120816

EState_VSA5 5.2808919234

fr_C_O_noCOO 5.2756788416

EState_VSA4 5.2331188165

Chi0v 5.2296786564

Chi4v 5.1764875087

fr_Al_COO 5.1073470615

fr_allylic_oxid 5.0723892093

SlogP_VSA3 5.03966163

SMR_VSA7 4.994757662

PEOE_VSA6 4.9834648116

NumAromaticCarbocycles 4.960128245

fr_COO 4.9361885927

fr_benzene 4.9207384048

fr_phos_ester 4.9122438942

SlogP_VSA6 4.9055499128

NOCount 4.8366751209

fr_Al_OH_noTert 4.6916864011

fr_NH0 4.6233899124

SMR_VSA3 4.594680812

EState_VSA7 4.5828456813

EState_VSA3 4.5702191902

fr_ether 4.5586691637

HeavyAtomCount 4.5534147295

fr_halogen 4.5157540853

MinAbsEStateIndex 4.5038941197

fr_COO2 4.4092768351

FractionCSP3 4.3811271305

EState_VSA1 4.3803075034

SlogP_VSA12 4.3694667229

NumAliphaticRings 4.2528709729

NumAliphaticCarbocycles 4.2258223397

NumAliphaticHeterocycles 4.17897997

fr_bicyclic 4.1284262286

PEOE_VSA2 4.0856516211

RingCount 3.9863498149

VSA_EState10 3.9832654138

SMR_VSA9 3.9632386403

fr_NH1 3.9310934046

NumAromaticRings 3.9130495535

VSA_EState8 3.8907181137

PEOE_VSA3 3.8787902222

EState_VSA6 3.8777009842

fr_Al_OH 3.727590259

PEOE_VSA4 3.6139116963

SlogP_VSA11 3.6129729708

SMR_VSA2 3.5863180581

fr_ester 3.532481218

fr_amide 3.5317016212

fr_Nhpyrrole 3.5237159849

fr_Ar_NH 3.3674141468

fr_ketone_Topliss 3.2590041638

PEOE_VSA12 3.2419422387

NumSaturatedCarbocycles 3.2196047565

fr_NH2 3.1254515868

SlogP_VSA7 2.9938053855

PEOE_VSA11 2.980661433

SlogP_VSA10 2.932461091

fr_Ar_N 2.9287033322

fr_C_O 2.9014436099

PEOE_VSA13 2.8294963642

NumSaturatedHeterocycles 2.820991551

fr_Imine 2.8124455485

NumSaturatedRings 2.7468749145

fr_unbrch_alkane 2.7111018203

fr_ArN 2.6636666734

PEOE_VSA5 2.6416877576

NumAromaticHeterocycles 2.595714179

fr_aldehyde 2.5119044398

SlogP_VSA8 2.4701648257

fr_ketone 2.4597852059

fr_alkyl_halide 2.4357043041

fr_imidazole 2.3579759264

VSA_EState2 2.2801535151

fr_methoxy 2.0457972738

fr_nitro 1.9942317796

fr_epoxide 1.8703832955

fr_Ndealkylation1 1.866755429

fr_aniline 1.855663002

fr_phenol 1.8424078899

fr_Ar_OH 1.7947739029

fr_SH 1.7580616609

fr_nitrile 1.6024588459

fr_amidine 1.4219545536

fr_priamide 1.3659686136

fr_sulfide 1.2513898692

fr_para_hydroxylation 1.2351000125

fr_Ndealkylation2 1.1471203407

fr_piperdine 1.1040472422

fr_phenol_noOrthoHbond 1.0875577768

fr_guanido 1.0232978103

fr_lactone 0.9266963524

fr_sulfonamd 0.8862249291

fr_N_O 0.8529348292

fr_oxime 0.7784941573

fr_aryl_methyl 0.7333344931

fr_nitro_arom_nonortho 0.6387713434

fr_dihydropyridine 0.6354596611

fr_piperzine 0.6129444097

NumRadicalElectrons 0.6045615784

fr_urea 0.5689065581

fr_nitro_arom 0.4968648951

fr_hdrzone 0.449466575

fr_lactam 0.449466575

fr_term_acetylene 0.449466575

fr_furan 0.4349971356

fr_Ar_COO 0.3682388215

fr_pyridine 0.1097251548

SMR_VSA8 0

SlogP_VSA9 0

VSA_EState1 0

VSA_EState3 0

VSA_EState4 0

VSA_EState5 0

VSA_EState6 0

VSA_EState7 0

fr_alkyl_carbamate 0

fr_azide 0

fr_azo 0

fr_barbitur 0

fr_benzodiazepine 0

fr_diazo 0

fr_hdrzine 0

fr_isocyan 0

fr_morpholine 0

fr_oxazole 0

fr_prisulfonamd 0

fr_sulfone 0

fr_tetrazole 0

fr_thiocyan 0

fr_thiophene 0

fr_HOCCN -0.0005705306

fr_imide -0.1769330539

EState_VSA11 -0.2073315161

fr_nitroso -0.449466575

fr_isothiocyan -0.7467654242

fr_thiazole -0.7582309418

fr_C_S -0.8099459531
