# Supplementary material for: ToxiM: A Toxicity Prediction Tool for Small Molecules Developed Using Machine Learning and Chemoinformatics Approaches
Source: Front Pharmacol. 2017 Nov 30;8:880. doi: 10.3389/fphar.2017.00880 (PMC5714866; doi:10.3389/fphar.2017.00880)
Supplement: Supplementary file 7 [file Table3.DOCX]

**Supplementary Table S3.**Selected descriptors for the solubility regression model with the help of multi-linear regression

| **Descriptors** | **Estimate** | **Std. Error** | **t value** | **Pr(>\|t\|)** |
| --- | --- | --- | --- | --- |
| (Intercept) | -44.404957 | 38.408779 | -1.156 | 0.24894 |
| MaxAbsEStateIndex | 0.155176 | 0.05271 | 2.944 | 0.0036 |
| Chi1n | -10.17173 | 3.165642 | -3.213 | 0.00152 |
| Chi1v | 2.345256 | 1.302452 | 1.801 | 0.07319 |
| Chi2n | -1.928726 | 0.872013 | -2.212 | 0.02805 |
| FractionCSP3 | -1.52157 | 0.636077 | -2.392 | 0.01763 |
| NumAliphaticCarbocycles | 4.489528 | 2.128 | 2.11 | 0.03606 |
| NumAliphaticHeterocycles | 4.404591 | 2.119015 | 2.079 | 0.03886 |
| NumAromaticCarbocycles | 4.43747 | 2.362685 | 1.878 | 0.06174 |
| NumAromaticHeterocycles | 4.73709 | 2.251348 | 2.104 | 0.03655 |
| fr_Al_COO | 8.120453 | 4.748751 | 1.71 | 0.08873 |
| fr_Ar_COO | 7.865361 | 4.688774 | 1.677 | 0.09493 |
| fr_Ar_NH | -1.787304 | 0.975682 | -1.832 | 0.06838 |
| fr_C_S | 7.431782 | 2.563739 | 2.899 | 0.00414 |
| fr_Imine | 3.141671 | 1.424068 | 2.206 | 0.02845 |
| fr_term_acetylene | 2.816237 | 1.53178 | 1.839 | 0.06739 |

Descriptor selection was made with the help of p value.
