# Supplementary material for: ToxiM: A Toxicity Prediction Tool for Small Molecules Developed Using Machine Learning and Chemoinformatics Approaches
Source: Front Pharmacol. 2017 Nov 30;8:880. doi: 10.3389/fphar.2017.00880 (PMC5714866; doi:10.3389/fphar.2017.00880)
Supplement: Supplementary file 8 [file Table4.DOCX]

**Supplementary TableS4.**Selection of descriptors for permeability model based on multi-linear regression approach

| **Descriptor** | **Estimate** | **Std. Error** | **t value** | **Pr(>\|t\|)** |
| --- | --- | --- | --- | --- |
| (Intercept) | -4.20E+00 | 1.43E-01 | -29.405 | < 2e-16 |
| PEOE_VSA1 | -1.99E-03 | 1.13E-02 | -0.176 | 0.860513 |
| NumHDonors | 2.59E-01 | 1.54E-01 | 1.681 | 0.095417 |
| NHOHCount | -4.56E-01 | 1.62E-01 | -2.808 | 0.005829 |
| TPSA | -8.45E-05 | 7.49E-03 | -0.011 | 0.991019 |
| MolLogP | 1.96E-02 | 6.52E-02 | 0.301 | 0.76389 |
| PEOE_VSA11 | -3.06E-02 | 9.68E-03 | -3.157 | 0.002017 |
| NumRotatableBonds | -1.02E-01 | 2.53E-02 | -4.012 | 0.000105 |
| BertzCT | -1.07E-05 | 3.68E-04 | -0.029 | 0.976727 |
| fr_NH1 | -2.62E-02 | 1.09E-01 | -0.24 | 0.810459 |
| NOCount | -8.74E-03 | 1.15E-01 | -0.076 | 0.939661 |
| MolWt | 6.31E-04 | 1.29E-03 | 0.489 | 0.625698 |

The descriptor selection was made on the basis of p value.
