# Supplementary material for: ToxiM: A Toxicity Prediction Tool for Small Molecules Developed Using Machine Learning and Chemoinformatics Approaches
Source: Front Pharmacol. 2017 Nov 30;8:880. doi: 10.3389/fphar.2017.00880 (PMC5714866; doi:10.3389/fphar.2017.00880)
Supplement: Supplementary file 9 [file Table5.DOCX]

**Supplementary TableS5.**Top 40 descriptors used for the construction of RF based regression model to calculate the solubility(LogS).

| **Descriptors List** |
| --- |
| MolLogP |
| PEOE_VSA6 |
| SlogP_VSA2 |
| MolMR |
| MaxEStateIndex |
| MaxAbsEStateIndex |
| LabuteASA |
| HallKierAlpha |
| BertzCT |
| Chi4v |
| TPSA |
| Chi1n |
| ExactMolWt |
| HeavyAtomMolWt |
| Chi4n |
| fr_halogen |
| NOCount |
| Chi1v |
| Chi2n |
| VSA_EState10 |
| MolWt |
| Chi0v |
| SMR_VSA7 |
| NumHAcceptors |
| SlogP_VSA5 |
| NumHeteroatoms |
| SMR_VSA10 |
| SMR_VSA1 |
| PEOE_VSA1 |
| NHOHCount |
| Chi3n |
| NumHDonors |
| MinEStateIndex |
| Chi3v |
| Chi0n |
| PEOE_VSA7 |
| BalabanJ |
| Chi2v |
| VSA_EState9 |
| Kappa3 |

Selection of descriptors was made with the help of %IncMSE values. After the inclusion of 40 descriptors, the R^2^ values showed a decline, and thus the top 40 descriptors were selected.

%IncMSE represents the increase in mse of predictions (approximated with OOB-CV) as a result of any variable being permuted. So, larger the %IncMSE value, important the descriptor will be.
