# Supplementary material for: ToxiM: A Toxicity Prediction Tool for Small Molecules Developed Using Machine Learning and Chemoinformatics Approaches
Source: Front Pharmacol. 2017 Nov 30;8:880. doi: 10.3389/fphar.2017.00880 (PMC5714866; doi:10.3389/fphar.2017.00880)
Supplement: Supplementary file 10 [file Table6.DOCX]

**Supplementary Table S6.**Top 10 descriptors used for the construction of RF based regression model to calculate theLogP.

| **Descriptors** |
| --- |
| PEOE_VSA1 |
| NHOHCount |
| TPSA |
| NumHDonors |
| VSA_EState9 |
| SlogP_VSA2 |
| BertzCT |
| SMR_VSA1 |
| Kappa3 |
| Chi0 |

Selection of descriptors was made with the help of %IncMSE values. After the inclusion of 10 descriptors, the R^2^ values showed a decline, and thus the top 10 descriptors were selected.
