# Supplementary material for: ToxiM: A Toxicity Prediction Tool for Small Molecules Developed Using Machine Learning and Chemoinformatics Approaches
Source: Front Pharmacol. 2017 Nov 30;8:880. doi: 10.3389/fphar.2017.00880 (PMC5714866; doi:10.3389/fphar.2017.00880)
Supplement: Supplementary file 11 [file Table7.DOCX]

**Supplementary Table S7.** Descriptor selected for the development PLS based regression model to calculate logS.

| **Descriptors** |
| --- |
| MolLogP |
| PEOE_VSA6 |
| MolMR |
| LabuteASA |
| Chi0v |
