# Supplementary material for: ToxiM: A Toxicity Prediction Tool for Small Molecules Developed Using Machine Learning and Chemoinformatics Approaches
Source: Front Pharmacol. 2017 Nov 30;8:880. doi: 10.3389/fphar.2017.00880 (PMC5714866; doi:10.3389/fphar.2017.00880)
Supplement: Supplementary file 12 [file Table8.DOCX]

**Supplementary Table S8.** Descriptors selected for the construction of PLS based model to calculate logP.

| **Descriptors** |
| --- |
| NumHDonors |
| NHOHCount |
| TPSA |
| PEOE_VSA1 |
| NOCount |
