# Supplementary material for: ToxiM: A Toxicity Prediction Tool for Small Molecules Developed Using Machine Learning and Chemoinformatics Approaches
Source: Front Pharmacol. 2017 Nov 30;8:880. doi: 10.3389/fphar.2017.00880 (PMC5714866; doi:10.3389/fphar.2017.00880)
Supplement: Supplementary file 13 [file Table9.DOCX]

**Supplementary Table S9.** Performance of RF based classification models on FDA withdrawn dataset.

| **SN** | **SDF ID** | **Name of Compounds** | **Methods** | **NT Score** | **T Score** | **NT/T** | **References** |
| --- | --- | --- | --- | --- | --- | --- | --- |
| 1 | 10007 | Chlorphentermine | D | 0.046 | 0.954 | T | Drug Information Journal. Vol. 35, pp. 293-317, 2001 |
|  |  |  | FP | 0.007 | 0.993 |  |  |
|  |  |  | H | 0.022 | 0.978 |  |  |
| 2 | 14130 | Bucetin | D | 0.117 | 0.883 | T | [[1](#_ENREF_1)] |
|  |  |  | FP | 0.135 | 0.865 |  |  |
|  |  |  | H | 0.101 | 0.899 |  |  |
| 3 | 15250 | Ibufenac | D | 0.069 | 0.931 | T | [[2](#_ENREF_2)] |
|  |  |  | FP | 0.153 | 0.847 |  |  |
|  |  |  | H | 0.072 | 0.928 |  |  |
| 4 | 15376 | Vincamine | D | 0.021 | 0.979 | T | [[3](#_ENREF_3)] |
|  |  |  | FP | 0.036 | 0.964 |  |  |
|  |  |  | H | 0.016 | 0.984 |  |  |
| 5 | 163093 | Isaxonine Phosphate | D | 0.316 | 0.684 | T | [[4](#_ENREF_4)] |
|  |  |  | FP | 0.131 | 0.869 |  |  |
|  |  |  | H | 0.162 | 0.838 |  |  |
| 6 | 16538 | Metofoline | D | 0.002 | 0.998 | T | Drug Safety Evaluation, 2nd Edition  Shayne Cox Gad  ISBN: 978-0-470-46409-0 |
|  |  |  | FP | 0.014 | 0.986 |  |  |
|  |  |  | H | 0.011 | 0.989 |  |  |
| 7 | 216235 | Sitaxsentan | D | 0.021 | 0.979 | T | [[5](#_ENREF_5)] |
|  |  |  | FP | 0.015 | 0.985 |  |  |
|  |  |  | H | 0.014 | 0.986 |  |  |
| 8 | 2313 | Bendazac | D | 0.062 | 0.938 | T | [[6](#_ENREF_6)] |
|  |  |  | FP | 0.09 | 0.91 |  |  |
|  |  |  | H | 0.076 | 0.924 |  |  |
| 9 | 239062 | Xenazoic Acid | D | 0.082 | 0.918 | T | [[7](#_ENREF_7)] |
|  |  |  | FP | 0.109 | 0.891 |  |  |
|  |  |  | H | 0.058 | 0.942 |  |  |
| 10 | 2406 | Bithionol | D | 0.015 | 0.985 | T | Dermatotoxicology, Eighth Edition  Klaus Peter Wilhelm, HongboZhai, Howard I. Maibach  ISBN 9781841848570 - CAT# HE10437 |
|  |  |  | FP | 0.022 | 0.978 |  |  |
|  |  |  | H | 0.029 | 0.971 |  |  |
| 11 | 2468 | Buformin | D | 0.419 | 0.581 | T | [[8](#_ENREF_8)] |
|  |  |  | FP | 0.36 | 0.64 |  |  |
|  |  |  | H | 0.405 | 0.595 |  |  |
| 12 | 2482 | Butamben | D | 0.036 | 0.964 | T | Drug Information Journal. Vol. 35, pp. 293-317, 2001 |
|  |  |  | FP | 0.032 | 0.968 |  |  |
|  |  |  | H | 0.038 | 0.962 |  |  |
| 13 | 26602 | Cloforex | D | 0.005 | 0.995 | T | [[9](#_ENREF_9)] |
|  |  |  | FP | 0.005 | 0.995 |  |  |
|  |  |  | H | 0.002 | 0.998 |  |  |
| 14 | 2788 | Clioquinol | D | 0.01 | 0.99 | T | [[10](#_ENREF_10)] |
|  |  |  | FP | 0.121 | 0.879 |  |  |
|  |  |  | H | 0.027 | 0.973 |  |  |
| 15 | 2898 | Cyclofenil | D | 0.039 | 0.961 | T | [[11](#_ENREF_11)] |
|  |  |  | FP | 0.028 | 0.972 |  |  |
|  |  |  | H | 0.013 | 0.987 |  |  |
| 16 | 29321 | Phenisatin | D | 0.029 | 0.971 | T | Drug Information Journal. Vol. 35, pp. 293-317, 2001 |
|  |  |  | FP | 0.017 | 0.983 |  |  |
|  |  |  | H | 0.01 | 0.99 |  |  |
| 17 | 2950 | Danthron | D | 0.057 | 0.943 | T | https://www.drugbank.ca/drugs/DB04816 |
|  |  |  | FP | 0.074 | 0.926 |  |  |
|  |  |  | H | 0.038 | 0.962 |  |  |
| 18 | 31315 | Oxyphenisatin | D | 0.043 | 0.957 | T | [[12](#_ENREF_12)] |
|  |  |  | FP | 0.1 | 0.9 |  |  |
|  |  |  | H | 0.073 | 0.927 |  |  |
| 19 | 31473 | Amoproxan | D | 0.068 | 0.932 | T | PMID: 5499223 |
|  |  |  | FP | 0.016 | 0.984 |  |  |
|  |  |  | H | 0.02 | 0.98 |  |  |
| 20 | 3351 | Fipexide | D | 0.003 | 0.997 | T | Drug Information Journal. Vol. 35, pp. 293-317, 2001 |
|  |  |  | FP | 0.009 | 0.991 |  |  |
|  |  |  | H | 0.007 | 0.993 |  |  |
| 21 | 34870 | Amineptine | D | 0.169 | 0.831 | T | [[13](#_ENREF_13)] |
|  |  |  | FP | 0.13 | 0.87 |  |  |
|  |  |  | H | 0.091 | 0.909 |  |  |
| 22 | 35455 | Feprazone | D | 0.016 | 0.984 | T | Drug Information Journal. Vol. 35, pp. 293-317, 2001 |
|  |  |  | FP | 0.012 | 0.988 |  |  |
|  |  |  | H | 0.012 | 0.988 |  |  |
| 23 | 35935 | Pirprofen | D | 0.041 | 0.959 | T | Drug Information Journal. Vol. 35, pp. 293-317, 2001 |
|  |  |  | FP | 0.037 | 0.963 |  |  |
|  |  |  | H | 0.03 | 0.97 |  |  |
| 24 | 3718 | Anolobine | D | 0.097 | 0.903 | T | [[14](#_ENREF_14)] |
|  |  |  | FP | 0.092 | 0.908 |  |  |
|  |  |  | H | 0.054 | 0.946 |  |  |
| 25 | 38409 | Ticrynafen | D | 0.066 | 0.934 | T | PMID: 7351824 |
|  |  |  | FP | 0.091 | 0.909 |  |  |
|  |  |  | H | 0.048 | 0.952 |  |  |
| 26 | 40399 | Exifone | D | 0.142 | 0.858 | T | Drug Information Journal. Vol. 35, pp. 293-317, 2001 |
|  |  |  | FP | 0.153 | 0.847 |  |  |
|  |  |  | H | 0.13 | 0.87 |  |  |
| 27 | 4472 | Nialamide | D | 0.104 | 0.896 | T | Drug Information Journal. Vol. 35, pp. 293-317, 2001 |
|  |  |  | FP | 0.114 | 0.886 |  |  |
|  |  |  | H | 0.094 | 0.906 |  |  |
| 28 | 4723 | Pemoline | D | 0.257 | 0.743 | T | [[15](#_ENREF_15)] |
|  |  |  | FP | 0.182 | 0.818 |  |  |
|  |  |  | H | 0.195 | 0.805 |  |  |
| 29 | 4781 | Phenylbutazone | D | 0.01 | 0.99 | T | [[16](#_ENREF_16)] |
|  |  |  | FP | 0.007 | 0.993 |  |  |
|  |  |  | H | 0.01 | 0.99 |  |  |
| 30 | 5281052 | Kavapyrone | D | 0.05 | 0.95 | T | [[17](#_ENREF_17)] |
|  |  |  | FP | 0.073 | 0.927 |  |  |
|  |  |  | H | 0.058 | 0.942 |  |  |
| 31 | 5282517 | AlatrofloxacinMesylate | D | 0.215 | 0.785 | T | http://www.fda.gov/ForConsumers/ConsumerUpdates/ucm053103.htm |
|  |  |  | FP | 0.202 | 0.798 |  |  |
|  |  |  | H | 0.175 | 0.825 |  |  |
| 32 | 53359 | Tolrestatin | D | 0.053 | 0.947 | T | Meyler's Side Effects of Endocrine and Metabolic Drugs, 1st Edition Authors: Jeffrey K. Aronson  Hardcover ISBN: 9780444532718  eBook ISBN: 9780080932927  Imprint: Elsevier Science  Published Date: 20th January 2009 |
|  |  |  | FP | 0.06 | 0.94 |  |  |
|  |  |  | H | 0.053 | 0.947 |  |  |
| 33 | 5354 | Suloctidyl | D | 0.193 | 0.807 | T | [[18](#_ENREF_18)] |
|  |  |  | FP | 0.08 | 0.92 |  |  |
|  |  |  | H | 0.101 | 0.899 |  |  |
| 34 | 5365247 | Zimelidine | D | 0.003 | 0.997 | T | [[19](#_ENREF_19)] |
|  |  |  | FP | 0 | 1 |  |  |
|  |  |  | H | 0 | 1 |  |  |
| 35 | 54897 | Alpidem | D | 0.003 | 0.997 | T | [[20](#_ENREF_20)] |
|  |  |  | FP | 0.008 | 0.992 |  |  |
|  |  |  | H | 0.006 | 0.994 |  |  |
| 36 | 60464 | Sparfloxacin | D | 0.105 | 0.895 | T | [[21](#_ENREF_21)] |
|  |  |  | FP | 0.112 | 0.888 |  |  |
|  |  |  | H | 0.093 | 0.907 |  |  |
| 37 | 65869 | Ebrotidine | D | 0.104 | 0.896 | T | [[22](#_ENREF_22)] |
|  |  |  | FP | 0.096 | 0.904 |  |  |
|  |  |  | H | 0.076 | 0.924 |  |  |
| 38 | 68723 | Antrafenine | D | 0.047 | 0.953 | T | Drug Information Journal. Vol. 35, pp. 293-317, 2001 |
|  |  |  | FP | 0.001 | 0.999 |  |  |
|  |  |  | H | 0.011 | 0.989 |  |  |
| 39 | 71467 | Phenoxypropazine | D | 0.262 | 0.738 | T | [[23](#_ENREF_23)] |
|  |  |  | FP | 0.06 | 0.94 |  |  |
|  |  |  | H | 0.202 | 0.798 |  |  |
| 40 | 71900 | Nitrefazole | D | 0.041 | 0.959 | T | Drug Information Journal. Vol. 35, pp. 293-317, 2001 |
|  |  |  | FP | 0.047 | 0.953 |  |  |
|  |  |  | H | 0.035 | 0.965 |  |  |
| 41 | 9574101 | Ximelagatran | D | 0.114 | 0.886 | T | [[24](#_ENREF_24)] |
|  |  |  | FP | 0.125 | 0.875 |  |  |
|  |  |  | H | 0.131 | 0.869 |  |  |

Where D: Descriptor, FP: Fingerprint and H: Hybrid model

T: Toxic, NT: Non-Toxic, MP: Mixed Prediction

1. Togei, K., et al., *Carcinogenicity of bucetin in (C57BL/6× C3H) F1 mice.* Journal of the National Cancer Institute, 1987. **79**(5): p. 1151-1158.

2. Herxheimer, A., *Ibufenac (Dytransin) withdrawn.* Drug Ther. Bull. **6**: p. 48.

3. Dany, F., et al., *Severe ventricular arrhythmia following parenteral administration of vincamine. Predisposing factors in 6 cases.* Archives des maladies du coeur et des vaisseaux, 1979. **73**(3): p. 298-306.

4. Davy, A., et al., *Hepatotoxicity of isaxonine phosphate: 4 cases of severe subacute hepatitis.* Gastroenterologie clinique et biologique, 1984. **8**(10): p. 715-719.

5. Galiè, N., et al., *Liver toxicity of sitaxentan in pulmonary arterial hypertension.* European Respiratory Journal, 2011. **37**(2): p. 475-476.

6. Hirode, M., et al., *Gene expression profiling in rat liver treated with compounds inducing elevation of bilirubin.* Human & experimental toxicology, 2009. **28**(4): p. 231-244.

7. Guengerich, F.P. and J.S. MacDonald, *Applying mechanisms of chemical toxicity to predict drug safety.* Chemical research in toxicology, 2007. **20**(3): p. 344-369.

8. DeFronzo, R., et al., *Metformin-associated lactic acidosis: Current perspectives on causes and risk.* Metabolism, 2016. **65**(2): p. 20-29.

9. MacDonald, J.S. and R.T. Robertson, *Toxicity testing in the 21st century: a view from the pharmaceutical industry.* Toxicological sciences, 2009. **110**(1): p. 40-46.

10. Franklin, R.B., J. Zou, and L.C. Costello, *The cytotoxic role of RREB1, ZIP3 zinc transporter, and zinc in human pancreatic adenocarcinoma.* Cancer biology & therapy, 2014. **15**(10): p. 1431-1437.

11. Rossi, G., E. Gabbi, and L. Serra, *Acute hepatitis induced by cyclofenil: a case report.* The Italian journal of gastroenterology, 1992. **24**(2): p. 77-78.

12. Schaffner, F., *Hepatic drug metabolism and adverse hepatic drug reactions.* Veterinary Pathology Online, 1975. **12**(2): p. 145-156.

13. Larrey, D., et al., *Genetic predisposition to drug hepatotoxicity: role in hepatitis caused by amineptine, a tricyclic antidepressant.* Hepatology, 1989. **10**(2): p. 168-173.

14. Sivelli, R., et al., *Duodenogastric reflux and gastric damage from non-steroidal antiinflammatory drugs.* International journal of tissue reactions, 1985. **8**(1): p. 61-66.

15. Berkovitch, M., et al., *Pemoline‐associated fulminant liver failure: Testing the evidence for causation.* Clinical Pharmacology & Therapeutics, 1995. **57**(6): p. 696-698.

16. Levang, J., et al. *Sweet's syndrome and phenylbutazone-induced sialadenitis*. in *Annales de Dermatologie et de Venereologie*. 2008.

17. Schulze, J., W. Raasch, and C.-P. Siegers, *Toxicity of kava pyrones, drug safety and precautions–a case study.* Phytomedicine, 2003. **10**: p. 68-73.

18. Chung, M.W., R.A. Komorowski, and R.R. Varma, *Suloctidil-induced hepatotoxicity.* Gastroenterology, 1988. **95**(2): p. 490-491.

19. Simpson, G. and N. Davidson, *Possible hepatotoxicity of zimelidine.* British medical journal (Clinical research ed.), 1983. **287**(6400): p. 1181.

20. Baty, V., et al., *Hepatitis induced by alpidem (Ananxyl). Four cases, one of them fatal.* Gastroenterologie clinique et biologique, 1993. **18**(12): p. 1129-1131.

21. Pierfitte, C., et al., *The link between sunshine and phototoxicity of sparfloxacin.* British journal of clinical pharmacology, 2000. **49**(6): p. 609-612.

22. Andrade, R.J., et al., *Acute liver injury associated with the use of ebrotidine, a new H 2-receptor antagonist.* Journal of hepatology, 1999. **31**(4): p. 641-646.

23. Cook, G. and S. Sherlock, *Jaundice and its relation to therapeutic agents.* The Lancet, 1965. **285**(7378): p. 175-179.

24. Keisu, M. and T. Andersson, *Drug-induced liver injury in humans: the case of ximelagatran*, in *Adverse Drug Reactions*. 2010, Springer. p. 407-418.
