# Supplementary material for: ToxiM: A Toxicity Prediction Tool for Small Molecules Developed Using Machine Learning and Chemoinformatics Approaches
Source: Front Pharmacol. 2017 Nov 30;8:880. doi: 10.3389/fphar.2017.00880 (PMC5714866; doi:10.3389/fphar.2017.00880)
Supplement: Supplementary file 14 [file Table10.DOCX]

**Supplementary Table S10.** Prediction of RF based classification models on second validation set. This dataset contained 15 compounds which are used by us in daily life but their use has been debatable.

| **SN** | **CID** | **Name** | **Model** | | | **NT/T** | **References** |
| --- | --- | --- | --- | --- | --- | --- | --- |
|  |  |  |  | **NT** | **T** |  |  |
| **1** | 174 | ethylene glycol | D | 0.579 | 0.421 | MP | [[1](#_ENREF_1)] |
|  |  |  | F | 0.316 | 0.684 |  |  |
|  |  |  | H | 0.456 | 0.544 |  |  |
| **2** | 5143 | Saccharin | D | 0.259 | 0.741 | T | [[2](#_ENREF_2)] |
|  |  |  | F | 0.044 | 0.956 |  |  |
|  |  |  | H | 0.091 | 0.909 |  |  |
| **3** | 6049 | EDTA | D | 0.091 | 0.909 | T | [[3](#_ENREF_3)] |
|  |  |  | F | 0.044 | 0.956 |  |  |
|  |  |  | H | 0.046 | 0.954 |  |  |
| **4** | 2943 | Dimethyl Tetrachloroterephthalate | D | 0.021 | 0.979 | T | USEPA Office of Pesticide Programs, Health Effects Division, Science Information Management Branch: "Chemicals Evaluated for Carcinogenic Potential" (April 2006) |
|  |  |  | F | 0.019 | 0.981 |  |  |
|  |  |  | H | 0.007 | 0.993 |  |  |
| **5** | 6658 | methyl methacrylate | D | 0.04 | 0.96 | T | [[4](#_ENREF_4)] |
|  |  |  | F | 0.013 | 0.987 |  |  |
|  |  |  | H | 0.269 | 0.731 |  |  |
| **6** | 8478 | Benzethonium Chloride | D | 0.113 | 0.887 | T | [[5](#_ENREF_5)] |
|  |  |  | F | 0.099 | 0.901 |  |  |
|  |  |  | H | 0.118 | 0.882 |  |  |
| **7** | 19665 | Butylhydroxybutylnitrosamine | D | 0.11 | 0.89 | T | [[6](#_ENREF_6)] |
|  |  |  | F | 0.169 | 0.831 |  |  |
|  |  |  |  |  |  |  |  |
| **8** | 38258 | Imidazolidinyl urea | H  D | 0.169  0.514 | 0.831  0.486 | MP |  |
|  |  |  |  |  |  |  |  |
|  |  |  | F | 0.215 | 0.785 |  |  |
|  |  |  | H | 0.221 | 0.779 |  |  |
| **9** | 134601 | Aspartame | D | 0.456 | 0.544 | T | [[7](#_ENREF_7)] |
|  |  |  | F | 0.248 | 0.752 |  |  |
|  |  |  | H | 0.279 | 0.721 |  | [[8](#_ENREF_8)] |
| **10** | 197578 | Polyacrylamide-butylamine polymer | D | 0.461 | 0.539 | MP |  |
|  |  |  | F | 0.648 | 0.352 |  |  |
|  |  |  | H | 0.515 | 0.485 |  |  |
| **11** | 5284448 | Polysorbate 80 (glycol) | D | 0.242 | 0.758 | T | [[9](#_ENREF_9)] |
|  |  |  | F | 0.211 | 0.789 |  |  |
|  |  |  | H | 0.235 | 0.765 |  |  |
| **12** | 23665760 | sodium hypochlorite | D | 0.527 | 0.473 | MP | [[10](#_ENREF_10)] |
|  |  |  | F | 0.457 | 0.543 |  |  |
|  |  |  | H | 0.514 | 0.486 |  |  |
| **13** | 23672308 | Sodium glutamate | D | 0.426 | 0.574 | T | [[11](#_ENREF_11)] |
|  |  |  | F | 0.493 | 0.507 |  |  |
|  |  |  | H | 0.309 | 0.691 |  |  |
| **14** | 23693105 | Sodium 1-tetradecanesulfonate | D | 0.12 | 0.88 | T |  |
|  |  |  | F | 0.178 | 0.822 |  |  |
|  |  |  | H | 0.105 | 0.895 |  |  |
| **15** | 86278622 | Asbestos | D | 0.282 | 0.718 | T | [[12](#_ENREF_12)] |
|  |  |  | F | 0.456 | 0.544 |  |  |
|  |  |  | H | 0.116 | 0.884 |  |  |

Where D: Descriptor, FP: Fingerprint and H: Hybrid model

T: Toxic, NT: Non-Toxic, MP: Mixed Prediction

1. Jacobsen, D. and K.E. McMartin, *Methanol and ethylene glycol poisonings.* Medical toxicology, 1986. **1**(5): p. 309-334.

2. Ellwein, L.B. and S.M. Cohen, *The health risks of saccharin revisited.* Critical reviews in toxicology, 1990. **20**(5): p. 311-326.

3. Fountain, J.S. and D.M. Reith, *Dangers of “EDTA”.* The New Zealand medical journal, 2014. **127**(1398): p. 126-127.

4. Leggat, P.A. and U. Kedjarune, *Toxicity of methyl methacrylate in dentistry.* International dental journal, 2003. **53**(3): p. 126-131.

5. Program, N.T., *NTP Toxicology and Carcinogenesis Studies of Benzethonium Chloride (CAS No. 121-54-0) in F344/N Rats and B6C3F1 Mice (Dermal Studies).* National Toxicology Program technical report series, 1995. **438**: p. 1.

6. Parkinson, D. and E. Lotzova, *Interleukin-2, killer cells and cancer therapy: an overview.* Natural immunity and cell growth regulation, 1989. **9**(4): p. 237-241.

7. Bae, O.-N., et al., *Chemical allergens stimulate human epidermal keratinocytes to produce lymphangiogenic vascular endothelial growth factor.* Toxicology and applied pharmacology, 2015. **283**(2): p. 147-155.

8. Maher, T.J. and R.J. Wurtman, *Possible neurologic effects of aspartame, a widely used food additive.* Environmental health perspectives, 1987. **75**: p. 53.

9. Roberts, C.L., et al., *Translocation of Crohn's disease Escherichia coli across M-cells: contrasting effects of soluble plant fibres and emulsifiers.* Gut, 2010: p. gut. 2009.195370.

10. Nickmilder, M., S. Carbonnelle, and A. Bernard, *House cleaning with chlorine bleach and the risks of allergic and respiratory diseases in children.* Pediatric allergy and immunology, 2007. **18**(1): p. 27-35.

11. Freeman, M., *Reconsidering the effects of monosodium glutamate: a literature review.* Journal of the American Association of Nurse Practitioners, 2006. **18**(10): p. 482-486.

12. Kanarek, M.S., *Mesothelioma from chrysotile asbestos: update.* Annals of epidemiology, 2011. **21**(9): p. 688-697.
