# Supplementary material for: ToxiM: A Toxicity Prediction Tool for Small Molecules Developed Using Machine Learning and Chemoinformatics Approaches
Source: Front Pharmacol. 2017 Nov 30;8:880. doi: 10.3389/fphar.2017.00880 (PMC5714866; doi:10.3389/fphar.2017.00880)
Supplement: Supplementary file 15 [file Table11.DOCX]

**Supplementary Table S11**. This table contains the LogP scores of withdrawn molecule. The permeability (LogPapp) cutoff was taken to be -5.1 from admetSAR.

| **Name** | **Prediction** |
| --- | --- |
| 1-(4-chlorophenyl)-2-methylpropan-2-amine | -4.51802868 |
| N-(4-ethoxyphenyl)-3-hydroxybutanamide | -4.83752026 |
| 2-[4-(2-methylpropyl)phenyl]acetic acid | -4.44253695 |
| 15376.sdf | -4.7273604 |
| phosphoric acid;N-propan-2-ylpyrimidin-2-amine | -5.31714049 |
| 1-[2-(4-chlorophenyl)ethyl]-6,7-dimethoxy-2-methyl-3,4-dihydro-1H-isoquinoline | -4.45068162 |
| N-(4-chloro-3-methyl-1,2-oxazol-5-yl)-2-[2-(6-methyl-1,3-benzodioxol-5-yl)acetyl]thiophene-3-sulfonamide | -4.67276731 |
| 2-(1-benzylindazol-3-yl)oxyacetic acid | -4.62917099 |
| 4-[[1-ethoxy-2-oxo-2-(4-phenylphenyl)ethyl]amino]benzoic acid | -4.82551649 |
| 2,4-dichloro-6-(3,5-dichloro-2-hydroxyphenyl)sulfanylphenol | -4.70769297 |
| 2-butyl-1-(diaminomethylidene)guanidine | -5.20521234 |
| ethyl N-[1-(4-chlorophenyl)-2-methylpropan-2-yl]carbamate | -4.57650498 |
| 2482.sdf | -4.58522998 |
| 5-chloro-7-iodoquinolin-8-ol | -4.47873869 |
| [4-[(4-acetyloxyphenyl)-cyclohexylidenemethyl]phenyl] acetate | -4.31886734 |
| [4-[1-acetyl-3-(4-acetyloxyphenyl)-2-oxoindol-3-yl]phenyl] acetate | -4.40008068 |
| 1,8-dihydroxyanthracene-9,10-dione | -4.68282347 |
| 3,3-bis(4-hydroxyphenyl)-1H-indol-2-one | -5.01933048 |
| [1-(3-methylbutoxy)-3-morpholin-4-ylpropan-2-yl] 3,4,5-trimethoxybenzoate | -4.72131492 |
| 1-[4-(1,3-benzodioxol-5-ylmethyl)piperazin-1-yl]-2-(4-chlorophenoxy)ethanone | -4.63221671 |
| 7-(6,11-dihydro-5H-dibenzo[1,2-a:1',2'-e][7]annulen-11-ylamino)heptanoic acid | -4.76704019 |
| 4-(3-methylbut-2-enyl)-1,2-diphenylpyrazolidine-3,5-dione | -4.39040857 |
| 2-[3-chloro-4-(2,5-dihydropyrrol-1-yl)phenyl]propanoic acid | -4.56593145 |
| 2-[4-(3-oxo-1H-isoindol-2-yl)phenyl]propanoic acid | -4.55306573 |
| 2-[2,3-dichloro-4-(thiophene-2-carbonyl)phenoxy]acetic acid | -4.50067755 |
| (2,3,4-trihydroxyphenyl)-(3,4,5-trihydroxyphenyl)methanone | -5.70657424 |
| N-benzyl-3-[2-(pyridine-4-carbonyl)hydrazinyl]propanamide | -5.13777962 |
| 2-amino-5-phenyl-1,3-oxazol-4-one | -4.60415072 |
| 4-butyl-1,2-diphenylpyrazolidine-3,5-dione | -4.39040857 |
| 2-[(E)-2-cyclohexa-1,5-dien-1-ylethenyl]-4-methoxy-2,3-dihydropyran-6-one | -4.33173306 |
| 7-[(1S,5R)-6-[[(2S)-2-[[(2S)-2-aminopropanoyl]amino]propanoyl]amino]-3-azabicyclo[3.1.0]hexan-3-yl]-1-(2,4-difluorophenyl)-6-fluoro-4-oxo-1,8-naphthyridine-3-carboxylic acid;methanesulfonic acid | -5.81838207 |
| 2-[[6-methoxy-5-(trifluoromethyl)naphthalene-1-carbothioyl]-methylamino]acetic acid | -4.63607582 |
| 2-(octylamino)-1-(4-propan-2-ylsulfanylphenyl)propan-1-ol | -4.77904396 |
| (Z)-3-(4-bromophenyl)-N,N-dimethyl-3-pyridin-3-ylprop-2-en-1-amine | -4.34898095 |
| 2-[6-chloro-2-(4-chlorophenyl)imidazo[1,2-a]pyridin-3-yl]-N,N-dipropylacetamide | -4.44748784 |
| 5-amino-1-cyclopropyl-7-[(3S,5R)-3,5-dimethylpiperazin-1-yl]-6,8-difluoro-4-oxoquinoline-3-carboxylic acid | -5.30021954 |
| N-(4-bromophenyl)sulfonyl-N'-[2-[[2-(diaminomethylideneamino)-1,3-thiazol-4-yl]methylsulfanyl]ethyl]methanimidamide | -5.19461131 |
| 2-[4-[3-(trifluoromethyl)phenyl]piperazin-1-yl]ethyl 2-[[7-(trifluoromethyl)quinolin-4-yl]amino]benzoate | -4.83804333 |
| 1-phenoxypropan-2-ylhydrazine | -4.87574835 |
| 2-methyl-4-nitro-1-(4-nitrophenyl)imidazole | -4.41508759 |
| ethyl 2-[[(1R)-1-cyclohexyl-2-[(2S)-2-[[4-[(Z)-N'-hydroxycarbamimidoyl]phenyl]methylcarbamoyl]azetidin-1-yl]-2-oxoethyl]amino]acetate | -5.58565369 |
