# Supplementary material for: ToxiM: A Toxicity Prediction Tool for Small Molecules Developed Using Machine Learning and Chemoinformatics Approaches
Source: Front Pharmacol. 2017 Nov 30;8:880. doi: 10.3389/fphar.2017.00880 (PMC5714866; doi:10.3389/fphar.2017.00880)
Supplement: Supplementary file 17 [file Table13.DOCX]

**Supplementary Table S13**.LogP values of compounds used in daily life.

The permeability (LogPapp) cutoff was taken to be -5.1 from admetSAR.

| **Name** | **Predicted** |
| --- | --- |
| Aspartame | -5.12843 |
| Ethylene glycol | -4.70769 |
| Butylhydroxybutylnitrosamine | -4.57713 |
| Polyacralaminde-butylamine | -5.13706 |
| Sodium hypochloride | -4.15345 |
| Sodium glutamate | -4.7141 |
| Sodium 1-tetradecanesulfonate | -4.12824 |
| Dimethyl tetrachloroterephthalate | -4.31887 |
| Imidazolidinyl urea | -6.75616 |
| Saccharin | -4.47123 |
| Polysorbate 80(glycol) | -5.37383 |
| EDTA | -5.37642 |
| Methyl methacrylate | -4.26039 |
| Benzethonium Chloride | -4.535 |
| Asbestos | -8.13159 |
