# Supplementary material for: ToxiM: A Toxicity Prediction Tool for Small Molecules Developed Using Machine Learning and Chemoinformatics Approaches
Source: Front Pharmacol. 2017 Nov 30;8:880. doi: 10.3389/fphar.2017.00880 (PMC5714866; doi:10.3389/fphar.2017.00880)
Supplement: Supplementary file 18 [file Table14.DOCX]

**Supplementary  Table S14**.LogS values for compounds used in day to day life.

The solubility (LogS) cutoff was taken to be -5.0. This cutoff was taken from J. Chem. Inf. Model., 2007, 47 (4), pp 1395–1404.

| **Name** | **Predicted** |
| --- | --- |
| Aspartame | -2.60244 |
| Ethylene glycol | 0.699447 |
| Butylhydroxybutylnitrosamine | -1.74554 |
| Polyacralaminde-butylamine | -0.64416 |
| Sodium hypochloride | -0.50977 |
| Sodium glutamate | -0.51211 |
| Sodium 1-tetradecanesulfonate | -4.13825 |
| Dimethyl tetrachloroterephthalate | -4.98654 |
| Imidazolidinyl urea | -3.09576 |
| Saccharin | -1.61527 |
| Polysorbate 80(glycol) | -5.43471 |
| EDTA | -2.32196 |
| Methyl methacrylate | -0.52753 |
| Benzethonium Chloride | -5.02872 |
| Asbestos | -2.90956 |
