# Supplementary material for: ToxiM: A Toxicity Prediction Tool for Small Molecules Developed Using Machine Learning and Chemoinformatics Approaches
Source: Front Pharmacol. 2017 Nov 30;8:880. doi: 10.3389/fphar.2017.00880 (PMC5714866; doi:10.3389/fphar.2017.00880)
Supplement: Supplementary file 19 [file Table15.DOCX]

| Name | NT | T | LogPapp | LogS | Factor | Papp(prediction) | S(prediction) |
| --- | --- | --- | --- | --- | --- | --- | --- |
| Aspartame | 0.456 | 0.544 | -5.12843 | -2.60244 | T | P | S |
| Ethylene glycol | 0.579 | 0.421 | -4.70769 | 0.699447 | NT | P | S |
| Butylhydroxybutylnitrosamine | 0.11 | 0.89 | -4.57713 | -1.74554 | T | P | S |
| Polyacralaminde-butylamine | 0.461 | 0.539 | -5.13706 | -0.64416 | T | P | S |
| Sodium hypochloride | 0.527 | 0.473 | -4.15345 | -0.50977 | NT | P | S |
| Sodium glutamate | 0.426 | 0.574 | -4.7141 | -0.51211 | T | P | S |
| Sodium 1-tetradecanesulfonate | 0.12 | 0.88 | -4.12824 | -4.13825 | T | P | S |
| Dimethyl tetrachloroterephthalate | 0.021 | 0.979 | -4.31887 | -4.98654 | T | P | S |
| Imidazolidinyl urea | 0.514 | 0.486 | -6.75616 | -3.09576 | NT | NP | S |
| Saccharin | 0.259 | 0.741 | -4.47123 | -1.61527 | T | P | S |
| Polysorbate 80(glycol) | 0.242 | 0.758 | -5.37383 | -5.43471 | T | NP | NS |
| EDTA | 0.091 | 0.909 | -5.37642 | -2.32196 | T | NP | S |
| Methyl methacrylate | 0.04 | 0.96 | -4.26039 | -0.52753 | T | P | S |
| Benzethonium Chloride | 0.113 | 0.887 | -4.535 | -5.02872 | T | P | S |
| Asbestos | 0.282 | 0.718 | -8.13159 | -2.90956 | T | NP | S |

**Supplementary Table S15:**This table contains values of classification score done using descriptor module of ToxiM and the regression score for solubility and permeability calculated using respective regression models mentioned in the manuscript.

This table uses results from descriptor model for the prediction of toxicity. We advise readers to go through the Table S10 for the better understanding of the classification results.

References:

1. http://pubs.acs.org/doi/pdf/10.1021/ci00009a013
2. http://lmmd.ecust.edu.cn/admetsar1/predict/

Notes:

- The solubility(LogS) cutoff was taken to be -5.0. This cutoff was taken from J. Chem. Inf. Model., 2007, 47 (4), pp 1395–1404.
- The permeability(LogPapp) cutoff was taken to be -5.1 from admetSAR.
