# Supplementary material for: ToxiM: A Toxicity Prediction Tool for Small Molecules Developed Using Machine Learning and Chemoinformatics Approaches
Source: Front Pharmacol. 2017 Nov 30;8:880. doi: 10.3389/fphar.2017.00880 (PMC5714866; doi:10.3389/fphar.2017.00880)
Supplement: Supplementary file 20 [file DataSheet1.DOCX]

**Supplementary Text S1.** Construction of negative dataset

Using the information of unique identifier (ID) of metabolites available at BIGG database ([King et al., 2016](#_ENREF_6)), 990 metabolites were retrieved from KEGG ([Kanehisa and Goto, 2000](#_ENREF_5)), 2,491 from HMDB ([Wishart et al., 2007](#_ENREF_10)) and 2,766 from MetaNetX ([Ganter et al., 2013](#_ENREF_2)). The MetaNetX information for some metabolites contains the ID for CHEBI database - (<https://www.ebi.ac.uk/chebi/>) ([Degtyarenko et al., 2008](#_ENREF_1)) which is its external resource. We had extracted the CHEBI 'ID' for those metabolites from BIGG (1,804). We removed the common metabolites within the three source databases to prepare the list of source ‘IDs’ for the unique metabolites in each of the three databases. The SDF files for metabolites were retrieved from their respective databases including CHEBI (848), HMDB (408) and KEGG. A total of 1,263 metabolites were used in the negative dataset.

**Supplementary Text S2.** Number of individual fingerprints calculated for all molecules

For the calculation of fingerprints, the software 'PaDEL' was used and ten fingerprints were included. These fingerprints along with their bit sizes are as follows : MACCS Fingerprinter (MACCSFP) – 166 bits, PubChemFingerprinter (PubChemFP) – 881 bits, Sub structure Fingerprint Count (SubFPC) – 307 bits, Fingerprinter – 1024 bits, Graph Only Fingerprinter (GraphFP) – 1024 bits, Estate Fingerprinter (EstateFP) – 79 bits, Klekota Roth Fingerprinter (KRFP) – 4860 bits, Sub structure Fingerprinter (SubFP) - 307 bits, Atom Pairs 2D Fingerprinter (AP2D) – 780 bits and Atom Pairs 2D Fingerprint Count (APC2D) - 780 bits.

**Supplementary Text S3.** Detailed description of calculated descriptors for all molecules

All 196 descriptors were calculated using the RDKit package,andwere classified into the following descriptor families: Connectivity descriptors (12), Constitutional descriptors (106), MOE-type descriptors (58), CPSA descriptors (1), Molecular property descriptors (4) and Topological descriptors (7).

**Supplementary Text S4.** Principal component analysis

The two axes in the principal component plot represent the two principal components. The first principal component is a linear combination of original predictor variables, which captures the maximum variance in the dataset. The second principal component is a linear combination of other predictors, which captures the remaining variance in the dataset and is not correlated with the first component. In the case of fingerprints, the toxins were observed to be closely clustered in the upper half and non-toxins were clustered in the lower half of the plot. The variance between PC-1 and PC-2 for the complete dataset was 10% and 3%, respectively (Supplementary Figure S3a). Also, the variance decreased up to PC-5 after which it remained constant (Supplementary Figure S3b). In the case of descriptors, toxins and non-toxins were clustered in the upper half and showed separate clustering. The variance between PC-1 and PC-2 for the whole dataset was 23.1% and 6.7%, respectively (Supplementary Figure S3c). It decreased rapidly up to PC-4 and after it, the decrease was almost constant (Supplementary Figure S3d). From the results, it is clear that PCA-based model is not expected to give the best performance. Hence, in the later analysis, we compared the performance of other machine learning based models for developing the tool.

**Supplementary Text S5.**Discussion on the terminal cases of real dataset

Asbestos is a combination six different minerals: chrysotile, crocidolite, amosite, anthophyllite, tremolite, and actinolite. Among these, chrysotile and amosite asbestos are common occurring. There have been studies that link asbestos with malignant mesothelioma, lung cancer, cancer of the throat, cancer of the larynx, and gastrointestinal cancer, which makes its exposure to humans toxic ([Gerrits and Landrigan, 1996](#_ENREF_3)). The same toxic potential was reported in our study, where it was found to be toxic by all the three models of ToxiM. The ambiguity arose when the solubility and permeability calculations were performed. Our webserver predicted asbestos to be water soluble, but it is known that asbestos is sparingly soluble in water ([Gronow, 1987](#_ENREF_4)).Asbestos was predicted to be non-permeable by ToxiM, but we could not gather any evidences regarding the cell permeability potential of asbestos.

Polysorbate 80(glycol) is a non-ionic surfactant, which is widely used as an additive in foods and pharmaceutical preparations and cosmetics as an emulsifier or as stabilizer. There have been studies to examine the carcinogenicity of Polysorbate 80(glycol). It was experimentally proven that Polysorbate 80(glycol) can cause cancer with prolonged exposure ([Program, 1995](#_ENREF_9)). This supported our prediction of its toxicity by all the three models of ToxiM. Polysorbate 80(glycol) was predicted to be non-soluble and non-permeable which is not in accordance with known experimental results (<http://jpdb.nihs.go.jp/jp14e/14data/Part-II/Polysorbate_80.pdf>).

**Refrences**

Degtyarenko, K., De Matos, P., Ennis, M., Hastings, J., Zbinden, M., Mcnaught, A., Alcántara, R., Darsow, M., Guedj, M., and Ashburner, M. (2008). ChEBI: a database and ontology for chemical entities of biological interest. *Nucleic acids research* 36**,** D344-D350.

Ganter, M., Bernard, T., Moretti, S., Stelling, J., and Pagni, M. (2013). MetaNetX. org: a website and repository for accessing, analysing and manipulating metabolic networks. *Bioinformatics***,** btt036.

Gerrits, J.F., and Landrigan, P.J. (1996). Asbestos‐related cancer. *CA: a cancer journal for clinicians* 46**,** 254-255.

Gronow, J.R. (1987). Dissolution of asbestos fibers in water. *Clay minerals* 22**,** 21-35.

Kanehisa, M., and Goto, S. (2000). KEGG: kyoto encyclopedia of genes and genomes. *Nucleic acids research* 28**,** 27-30.

King, Z.A., Lu, J., Dräger, A., Miller, P., Federowicz, S., Lerman, J.A., Ebrahim, A., Palsson, B.O., and Lewis, N.E. (2016). BiGG Models: A platform for integrating, standardizing and sharing genome-scale models. *Nucleic acids research* 44**,** D515-D522.

Mayr, A., Klambauer, G., Unterthiner, T., and Hochreiter, S. (2016). DeepTox: toxicity prediction using deep learning. *Frontiers in Environmental Science* 3**,** 80.

Mishra, N.K., Singla, D., Agarwal, S., and Raghava, G.P. (2014). ToxiPred: A server for prediction of aqueous toxicity of small chemical molecules in T. Pyriformis. *Journal of Translational Toxicology* 1**,** 21-27.

Program, N.T. (1995). NTP Toxicology and Carcinogenesis Studies of Benzethonium Chloride (CAS No. 121-54-0) in F344/N Rats and B6C3F1 Mice (Dermal Studies). *National Toxicology Program technical report series* 438**,** 1.

Wishart, D.S., Tzur, D., Knox, C., Eisner, R., Guo, A.C., Young, N., Cheng, D., Jewell, K., Arndt, D., and Sawhney, S. (2007). HMDB: the human metabolome database. *Nucleic acids research* 35**,** D521-D526.
