# Supplementary material for: ToxiM: A Toxicity Prediction Tool for Small Molecules Developed Using Machine Learning and Chemoinformatics Approaches
Source: Front Pharmacol. 2017 Nov 30;8:880. doi: 10.3389/fphar.2017.00880 (PMC5714866; doi:10.3389/fphar.2017.00880)
Supplement: Supplementary file 21 [file DataSheet2.DOCX]

**Abbreviation**

**Table 1.** This table contains symbols and names of all the atoms and atom pairs identified by atomcount function used in compositional analysis.

| **Abbreviations** | **Name** |
| --- | --- |
| C | Carbon |
| O | Oxygen |
| N | Nitrogen |
| P | Phosphorous |
| H | Hydrogen |
| Se | Selenium |
| S | Sulphur |
| R | Alkyl |
| Fe | Iron |
| Co | Cobalt |
| ACP | Atom pair |
| Cu | Copper |
| Cl | Chlorine |
| Sn | Tin |
| Br | Bromine |
| As | Arsenic |
| Hg | Mercury |
| Na | Sodium |
| Pb | Led |
| B | Boron |
| Cd | Cadmium |
| F | Fluorine |
| Ni | Nickel |
| Cr | Chromium |
| K | Potassium |
| Zn | Zinc |
| Al | Aluminium |
| Ti | Titanium |
| Pd | Palladium |
| Ag | Silver |
| V | Vanadium |
| Sb | Antimony |
| I | Iodine |
| Pt | Platinum |
| Si | Silicon |
| Mn | Manganese |
| Ca | Calcium |
| Ba | Barium |
| U | Uranium |
| Mg | Magnesium |

**Table 2:** The list of important fingerprints and their names is shown

| **Fingerprints** | **Name** | **SMARTS** |
| --- | --- | --- |
| APC2D2_C_C | Presence of C-C at topological distance 2 |  |
| APC2D3_C_C | Presence of C-C at topological distance 3 |  |
| SubFPC302 | Rotatable bond |  |
| SubFP296 | Charged |  |
| APC2D4_C_C | Presence of C-C at topological distance 4 |  |
| APC2D7_C_C | Presence of C-C at topological distance 7 |  |
| SubFPC301 | 1,5-tautomerizable |  |
| SubFPC307 | Chiral center specified |  |
| APC2D1_C_C | Presence of C-C at topological distance 1 |  |
| MACCSFP49 | Charge | ('[!+0]',0) |
| SubFPC299 | Salt |  |
| APC2D6_C_C | Presence of C-C at topological distance 6 |  |
| SubFPC2 | Secondary Carbon |  |
| SubFPC296 | Charged |  |
| APC2D5_C_C | Presence of C-C at topological distance 5 |  |
| KRFP1147 |  | [!#1][O-] |
| APC2D8_C_C | Presence of C-C at topological distance 8 |  |
| SubFPC295 | CONS bond |  |
| SubFP299 | Salt |  |
| EStateFP20 | sNH3 | [ND1H3]-* |
| MACCSFP166 | Fragments |  |
| SubFP84 | Carboxylic acid |  |
| SubFP297 | Anion |  |
| SubFPC1 | Primary Carbon |  |
| SubFPC300 | 1,3-tautomerizable |  |
| SubFP298 | Cation |  |
| APC2D3_C_O | Presence of C-O at topological distance 3 |  |
| APC2D1_C_O | Presence of C-O at topological distance 1 |  |
| SubFPC297 | Anion |  |
| APC2D1_C_N | Presence of C-N at topological distance 1 |  |
| SubFPC14 | Tertiary alcohol | [OX2H][CX4D4;!$(C([OX2H])[O,S,#7,#15])] |

**Other abbreviations**

CV = cross-validation is a model validation technique to access the extrapolation of results of statistical analysis on independent data

MCC = Matthews’s correlation coefficient is used as a measure of quality of binary classifications. It produces values between -1 and +1, where, -1= complete disagreement and +1= a perfect prediction

MLR = Multi-linear Regression

PLS = Partial Least Square regression
